# Automated Syntheses of Xylan Oligosaccharides Containing β3-Linkages Enable Substrate Specificity Studies of Xylanases from Marine Bacteria

**DOI:** 10.1021/acs.orglett.5c04433

**Published:** 2025-11-26

**Authors:** Nitish Verma, Nils Rustmeier, Uwe Osswald, Fabian Pfrengle

**Affiliations:** Institute of Organic Chemistry, Department of Natural Sciences and Sustainable Resources, 27270 BOKU University, Muthgasse 18, 1190 Vienna, Austria

## Abstract

Marine polysaccharides, including β3-xylan and mixed-linkage xylan (MLX), play a central role in the ocean’s carbon cycle. Using an optimized xylose donor, automated glycan assembly enabled the synthesis of β3- and MLX-oligosaccharides up to decasaccharides. Interrogation of these structurally defined substrates with the marine xylanases Xyl4, AlXyn26A, and MfXyn26A revealed a surprisingly broad substrate scope of MLXases toward both β3-xylan and MLX motifs, highlighting AGA-derived glycans as powerful probes for studying marine polysaccharide metabolism.

## Graphical abstract

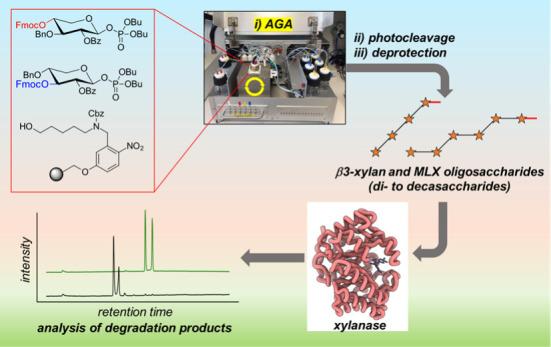

Marine algae fix nearly 50 gigatons of inorganic carbon annually into organic matter, approximately 80% of which consists of polysaccharides.^1^ While a large fraction is metabolized by heterotrophic bacteria, resulting in the release of CO_2_ back into the atmosphere, a small proportion escapes degradation. These polysaccharides are transported to the seafloor through vertical mixing and particle sinking, either because their biosynthesis outpaces bacterial decomposition or because their specific glycan structures render them resistant to enzymatic hydrolysis. This process is referred to as glyco-carbon sequestration.^1,2^

β3-xylan and mixed-linkage xylan (MLX) are algal polysaccharides involved in the marine carbon cycle and serve as an important source of organic carbon in the ocean.^3,4^ β3-xylan is a linear homopolysaccharide composed of d-xylose residues connected via β3-glycosidic bonds. It represents the predominant xylan structure in the cell walls of green and red algae.^4,5^ The β3-xylan of the green alga *Caulerpa lentillifera* has been reported to exhibit diverse bioactivities, including antitumor, apoptotic, and anticoagulant effects.^5,6^ In contrast, MLX is characterized by the presence of both β3- and β4-glycosidic linkages. The ratio of β3 to β4 linkages is approximately 1:4, with β3 linkages distributed irregularly along the β4-xylan backbone. MLX polysaccharides have been isolated from the edible red alga *Palmaria palmata*.^7^ As integral components of marine biomass, xylans represent a valuable renewable resource for biotechnological applications, including the production of functional xylooligosaccharides (XOS) for food and feed, the conversion into sustainable chemical commodities, and the utilization in pharmaceutical agents.^8–10^

Marine bacteria degrade algal xylans into fermentable xylose through the cooperative action of xylanases and xylosidases. While the enzymes that break down terrestrial plant xylans are well characterized, those acting on marine algal xylan remain comparatively understudied. Endo-β3-xylanases (from here on denoted as β3-xylanases) specifically hydrolyze β3-xylan of marine algae but are not active on β4-xylan of terrestrial plants.^11^ These enzymes, which so far have only been reported within the glycoside hydrolase family 26 (GH26; see cazy.org), progressively degrade pure β3-xylan into di- and trisaccharides, with xylose produced as a byproduct.^12–15^

A recently identified class of marine bacterial GH26 xylanases, termed MLXases, specifically hydrolyze the β4-linkages of β3Xylβ4Xyl motifs within MLX. From the native xylan of the red alga *Palmaria palmata*, MLXases release XOS with degrees of polymerization (DP) ranging from 2 to 8, with tri-, penta-, and hexaxylosides as dominant products.^4^ Both β3-xylanases and MLXases are endoacting enzymes and are secreted extracellularly. Within marine ecosystems, these enzymes mediate the degradation of external algal polysaccharides to efficiently utilize xylan as a source of carbon and energy.^16,17^

One of the key challenges in advancing the systematic characterization of xylanases from marine bacteria is the missing availability of regio-defined XOS containing β3-glycosidic linkages. Access to a defined library of tailored oligosaccharides would facilitate the analysis of hydrolysis products, enabling detailed active-site mapping and the precise determination of substrate specificities.^18–21^ Previously, β3-XOS (tri- to hexasaccharides) were synthesized in solution using disarmed xylose trichloroacetimidate donors.^3,22^ Automated glycan assembly (AGA) provides a more efficient and versatile platform for the synthesis of such oligosaccharides, allowing the streamlined preparation of structurally related glycans from a limited set of monosaccharide building blocks (BBs).^23^ We have previously reported the automated synthesis of arabinoxylan oligosaccharides derived from plant cell walls, containing β4-xylosidic linkages.^24,25^ Here, we describe AGA of β3-xylan and MLX oligosaccharides, as found in algal cell walls, which serve as valuable tools for the biochemical characterization of marine bacterial xylanases.

The synthesis of β3-xylan and MLX oligosaccharides requires two distinct xylose building blocks (BBs) for elongation at the C3- or C4-position. While BBs and reaction conditions for AGA of exclusively β4-linked xylans, as present in land plants, are well established,^24^ suitable BBs and conditions for efficient β3-linkage formation had to be identified. Due to their relatively straightforward preparation, BBs **2** and **3** were initially investigated (Scheme 1). These BBs contain benzoyl groups to provide permanent protection at the C2- and C4-positions, and an Fmoc group at the C3-position for chain elongation. As leaving groups, thiotolyl (BB **2**) and dibutyl-phosphate (BB **3**) were selected.

**Scheme 1:**
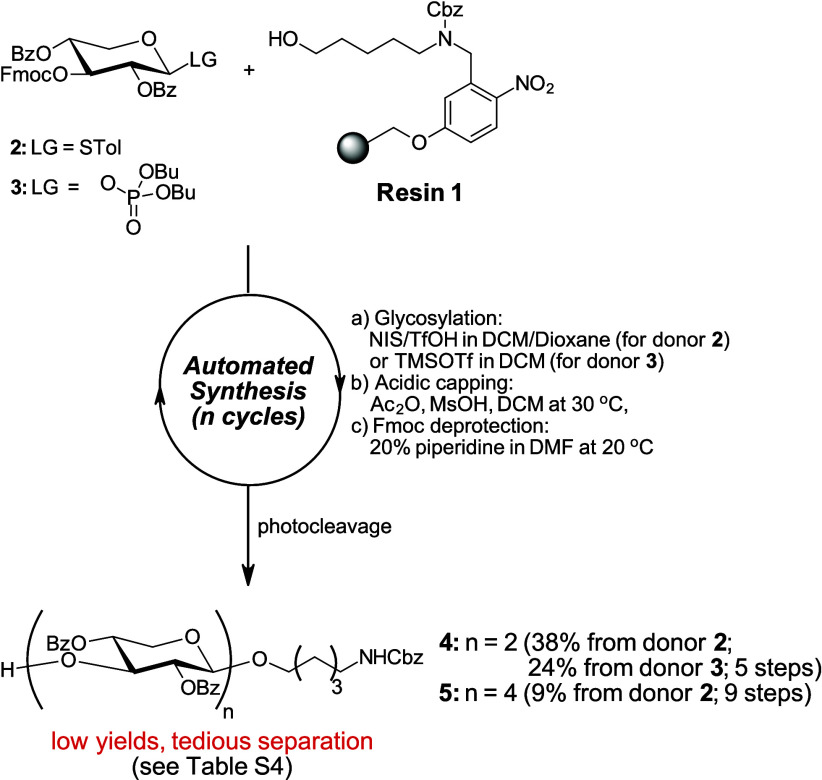
AGA of β3-Xylan Oligosaccharides Using Disarmed Donors **2** and **3**

After synthesizing multigram quantities of donors **2** and **3** (see Scheme S1), we evaluated their potential for the synthesis of β3-XOS (Scheme 1). The oligosaccharides were assembled on aminopentyl linker-functionalized resin **1** to facilitate detection of reducing end fragments in HPLC-MS assays used for evaluating xylanase specificity. First, AGA of a β3-xylan disaccharide was performed under standard reaction conditions for thioglycoside donors: an initial low temperature (T1 = – 20 °C for 5 min), followed by a higher reaction temperature (T2 = 0 °C for 20 min). One glycosylation cycle with 6.5 equiv of BB per linkage yielded disaccharide **4** in 14% (entry 1, Table S4). Extending the low temperature reaction time (T1) to 20 min^26^ improved the yield of disaccharide **4** to 38% (entry 2, Table S4). Omitting the capping step (to prevent potential chain cleavage under acidic conditions), doubling the glycosylation cycles, or lowering the reaction temperature (T1 to – 30 °C and T2 to – 10 °C) did not provide improvements. Applying the optimized conditions (entry 2, Table S4) to the synthesis of tetrasaccharide **5** gave only 9% yield (see Figures S2 and S4). The reduced yields may have resulted from an increasing mismatch between the reactivities of the glycosyl donor and acceptor as the glycan acceptor size increased. We speculate that the decreased reactivity of the acceptor hydroxy group may be attributed to the higher number of electron-withdrawing ester groups.

For the synthesis of β4-XOS, phosphate donors proved superior to thioglycosides.^24^ In contrast, the yield of β3-disaccharide **4** at various temperatures were not improved when using phosphate donor **3**. We attribute this to the 2,4-diBz-protected phosphate donor **3** being too electron-deficient to support efficient glycosylation. We thus prepared the more reactive 4-*O*-benzyl (Bn)-protected xylose phosphate **6** (see Scheme S2) for the synthesis of β3-XOS.

An initial attempt using the corresponding thioglycoside donor afforded disaccharide **7** in 37% yield with 24% monosaccharide as a side product (see Supporting Information). In contrast, a single cycle with 5 equiv of phosphate donor **6** (Scheme 2) delivered **7** in 73% yield. We then performed AGA of β3-xylan tetrasaccharide **8**, hexasaccharide **9**, and decasaccharide **10** (Scheme 2). Notably, decasaccharide **10** was obtained in 24 h with 27% overall yield over 21 steps.

**Scheme 2:**
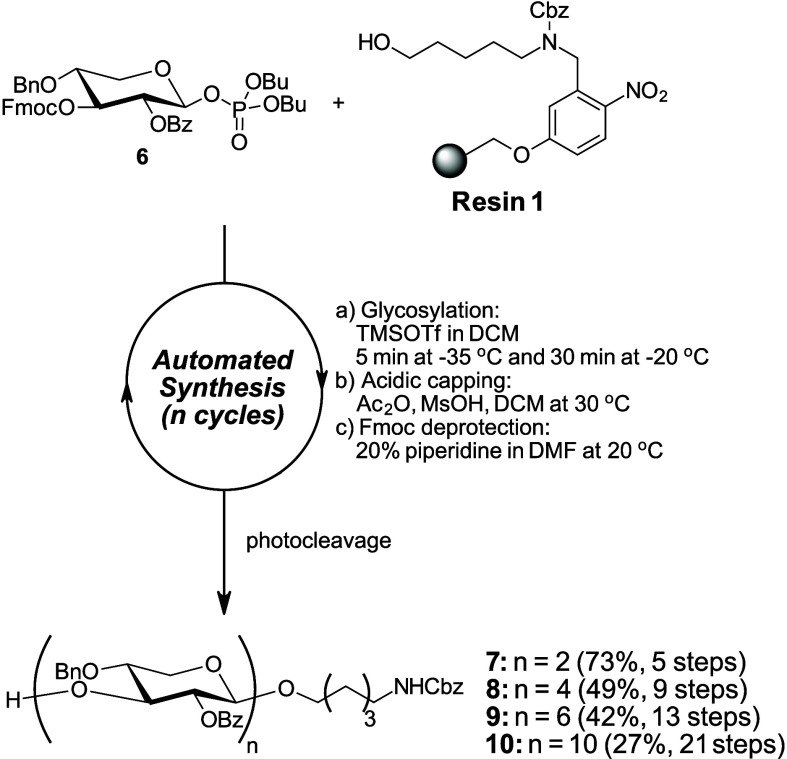
AGA of β3-Xylan Oligosaccharides Using Armed Donor **6**

Following assembly of the β3-XOS, the Bz and Bn protecting groups were removed to furnish the unprotected XOS (Scheme 3). The sequence commenced with a Zemplén deacylation, followed by hydrogenolysis with 10% Pd/C under an H_2_ atmosphere. To reduce reaction times and suppress formation of *N*-alkylated side products, benzyl cleavage was most efficiently performed under 8 bar H_2_ in a *t*-BuOH/H_2_O/AcOH solvent system. Under these conditions, hydrogenolysis of the ether protecting groups in all oligosaccharides proceeded efficiently, affording the desired products (**11**–**14**) in good yields.

**Scheme 3:**
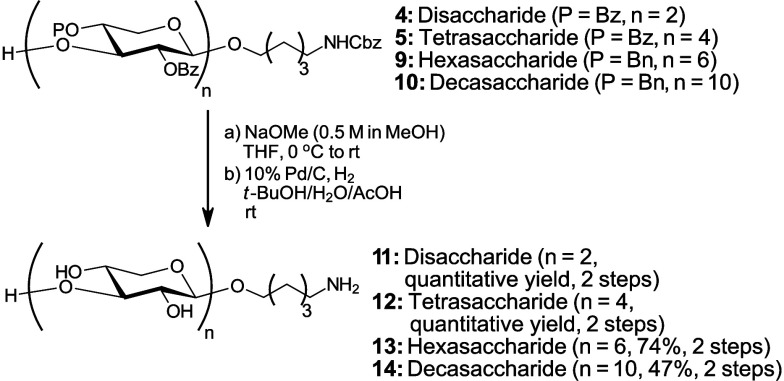
Deprotection of β3-Xylan Oligosaccharides

We then synthesized a series of MLX oligosaccharides (Scheme 4). Besides phosphate donor **6** for the formation of β3-linkages, we employed phosphate donor **15**, previously established as an effective donor for β4-xylan synthesis.^24^ AGA of the protected form of trisaccharide **16**, bearing a β4-linkage at the reducing end, afforded the desired product in 22% yield after photocleavage, together with 16% of the respective disaccharide deletion sequence as a side product. In contrast, the protected trisaccharide form of **17**, carrying a β3-linkage at the reducing end, was obtained in 45% yield. Glycosylations with donor **6** outperformed those with donor **15**, particularly in the initial coupling to the linker. When using donor **15**, two glycosylation cycles were required to achieve satisfactory yields for larger MLX oligosaccharides (e.g., **18**, **19**, and **21**). For MLX oligosaccharides **20**, **22**, and **23**, two cycles were applied in all glycosylation reactions. Global deprotection and C18 column chromatography furnished MLX oligosaccharides **16**–**23** in high purity.

**Scheme 4:**
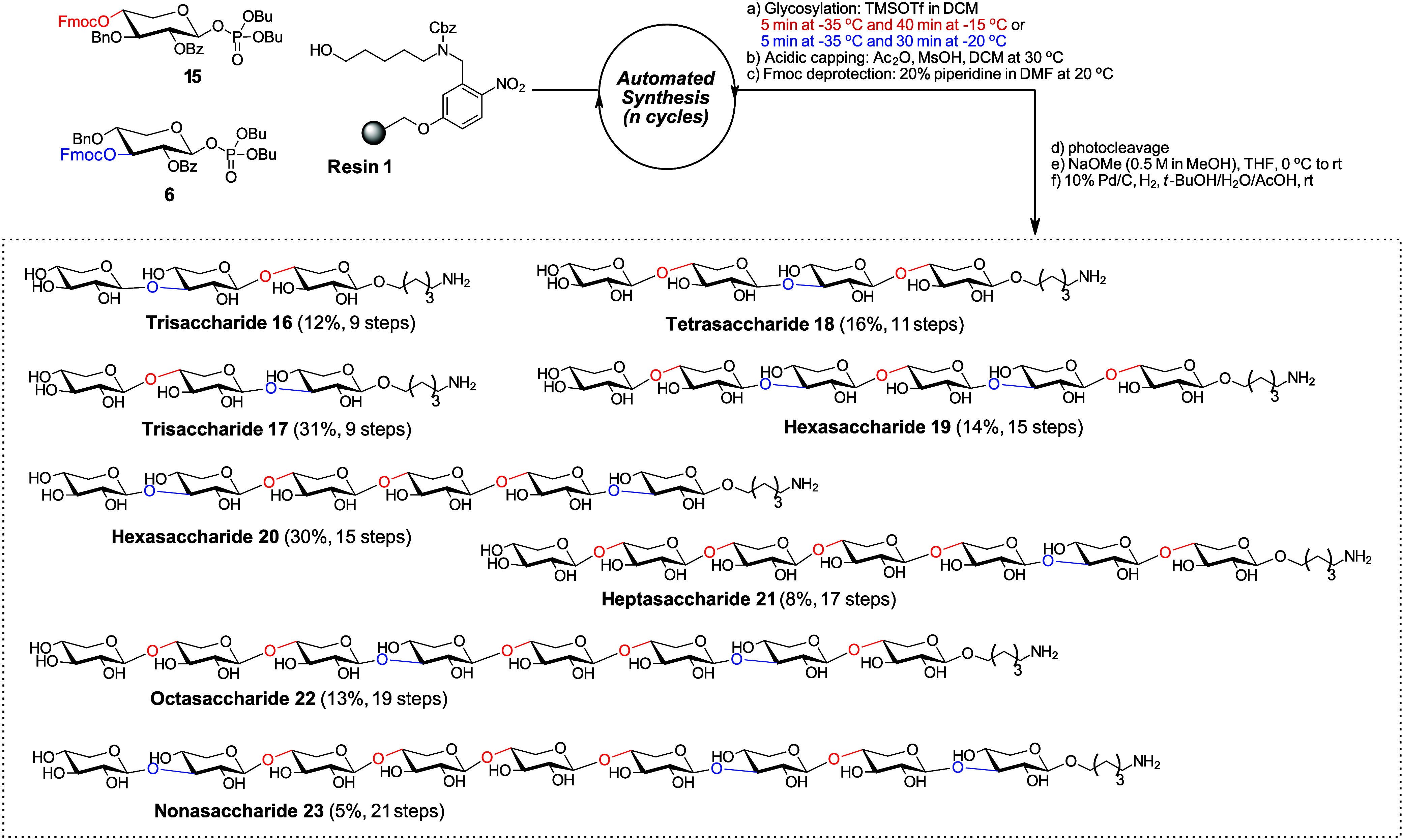
AGA and Deprotection Reactions for the Synthesis of MLX Oligosaccharides

Using our series of synthetic XOS (see Figure 1A), the substrate specificities of two endolytic xylanases were assessed: Xyl4, a homo-β3-xylanase from *Vibrio* spec. AX-4, and MLXase AlXyn26A from *Algibacter* sp. L4_22, which cleaves β4-glycosidic bonds that follow a β3-linkage toward the reducing end side of the xylan polymer. The XOS were incubated with the recombinant catalytic domains of these enzymes at 20 °C for 1 h or 37 °C for 24–26 h to monitor initial and final reaction stages. The hydrolysis products were analyzed by porous graphitic carbon HPLC-MS.

**Figure 1:**
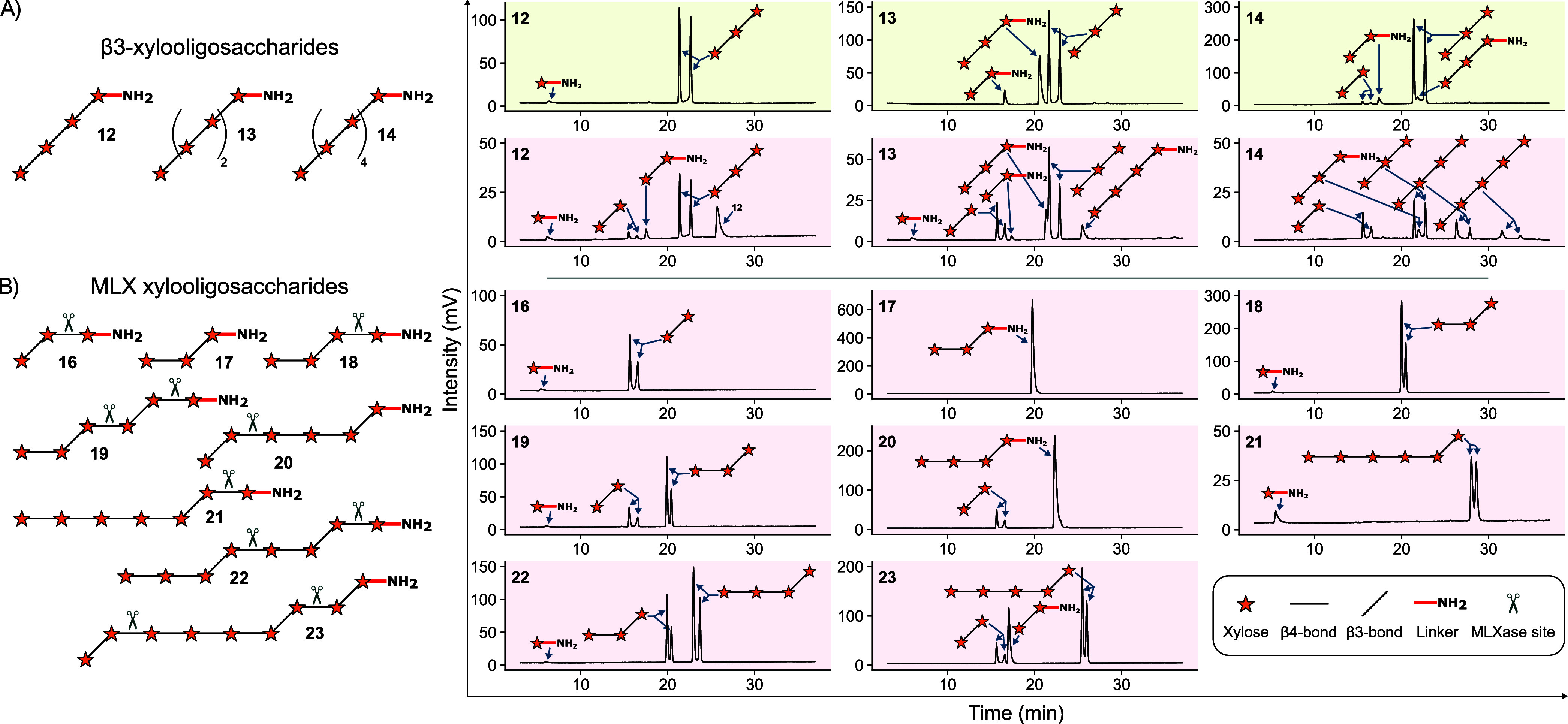
Enzymatic digest of synthetic xylooligosaccharides (XOS). Symbol structures of starting materials with theoretical MLXase cleavage sites indicated by scissors are shown on the left. HPLC-MS analyses of hydrolysis products after 1 h of incubation with β3-xylanase Xyl4 (green shaded plots) and MLXase AlXyn26A (red shaded plots) of β3-XOS **12**–**14** (A) and MLX oligosaccharides **16**–**23** (B).

First, the digest of synthetic β3-XOS by Xyl4 and AlXyn26A was investigated. Xyl4 cleaved tetrasaccharide **12** into trixyloside and linker-functionalized monosaccharide (Xyl-L). Hexasaccharide **13** was converted into trixylosides with and without linker as the major products and dixyloside-L. From decasaccharide **14**, the trixyloside was the dominant hydrolysis product along with trixyloside-L and dixylosides (see Figure 1A, right). Surprisingly, AlXyn26A, previously characterized as MLX-specific, also hydrolyzed β3-XOS into distinct products. AlXyn26A degraded tetrasaccharide **12** into trixyloside, dixylosides with and without linker, and Xyl-L. Hexasaccharide **13** was digested into tetraxyloside-L, trixylosides with and without linker, dixylosides with and without linker, and Xyl-L. From decasaccharide **14**, AlXyn26A released β3-XOS with degrees of polymerization ranging from two to five (see Figure 1A, right).

To further compare the catabolic proficiencies of Xyl4 and AlXyn26A, we monitored the progression of their β3-XOS digests over time. In all cases, Xyl4 primarily produced trixyloside, whereas AlXyn26A generated dixyloside as the dominant product (see Figure S7). These results demonstrate that AlXyn26A not only exhibits β3-xylanase activity but also produces products distinct from those generated by the well-established activity of Xyl4.

MLX oligosaccharides **16**–**23**, which were designed to feature specific MLXase cleavage sites within their structures (β3Xylβ4Xyl motifs, see Figure 1), were unaltered by Xyl4 (see Figure S8), confirming the enzyme’s strict specificity for continuous β3-linked xylose residues. In contrast, AlXyn26A hydrolyzed compounds **16**–**23** into the expected hydrolysis products after 1 h of incubation (see Figure 1B). Additionally, partial hydrolysis of the linker bond was observed in **17** and **20** after incubation for 24 h (see Figure S8). These reactions likely occurred because, in both compounds, the linker bond is next to a β3-xylosidic linkage, making it susceptible to MLXase cleavage.

Previous reports suggested GH26 MLXases have little activity on β3-xylan, based on studies using extracted polysaccharide from the green alga *Caulerpa lentillifera*.^4^ However, AlXyn26A efficiently digested our synthetic β3-XOS, indicating broader substrate specificity. To test if this applies to homologous enzymes, we produced recombinant MfXyn26A, a novel GH26 xylanase from the brown algae-resident bacterium *Mariniflexile fucanivorans*. Using natural MLX from *Palmaria palmata* and β3-decasaccharide **14**, MfXyn26A produced the same XOS as AlXyn26A (see Figure S9). These findings highlight the utility of synthetic oligosaccharides for enzyme characterization and suggest that substrate heterogeneity in natural xylans may lead to discrepant reports.

In conclusion, using AGA with two distinct xylose BBs, we achieved the first synthesis of a series of precision XOS containing β3-linkages, specifically designed for metabolic enzyme characterization. These XOS include specific cleavage sites for β3-xylanases and mixed-linked xylanases (MLXases), which cleave the β4-bonds following β3-xyloses toward the reducing end site. In this study, three purely β3-linked and eight mixed-linked XOS were incubated with the β3-xylanase Xyl4 and the MLXases AlXyn26A and MfXyn26A. While enzymatic digests largely proceeded as anticipated, we observed that the MLXases AlXyn26A and MfXyn26A also hydrolyzed the β3-bonds within our purely β3-linked XOS. In natural environments, bacteria salvage MLX as a resource for energy and carbon.^3,4^ The newly discovered activity of MLXases to target stretches of consecutive β3-bonds within XOS may enhance the efficiency of this process. Whether this activity is universal to all MLXases remains to be determined.

AGA of XOS incorporating chemically modified or nonxylose monosaccharides may provide more structurally complex mimetics of natural xylans in the future. This is particularly important, as the polysaccharide structure varies across algal species, seasons, and habitats.^27^ These synthetic XOS, which feature aminolinker-functionalization for facile fluorophore attachment, represent powerful probes for screening metabolic activities in microbial communities or seawater samples.^28^ Furthermore, our findings may contribute to improving valorization of algal biomass containing xylan.

## Supplementary Material



## Data Availability

The data underlying this study are available in the published article and its Supporting Information.

## Notes

Synthesizer modules and conditions, synthesis of xylose BBs, optimization of AGA reactions using disarmed donors (Table S4), copies of NMR spectra, and additional analyses of enzymatic degradation products (PDF)
