# Supplementary material for: Automated Syntheses of Xylan Oligosaccharides Containing β3-Linkages Enable Substrate Specificity Studies of Xylanases from Marine Bacteria
Source: Org Lett. 2025 Nov 26;27(49):13565–70. doi: 10.1021/acs.orglett.5c04433 (PMC12706835; doi:10.1021/acs.orglett.5c04433)
Supplement: Supplementary file 1 [file ol5c04433_si_001.pdf]

Supporting Information

For

Automated Syntheses of Xylan Oligosaccharides Containing β3-Linkages Enable Substrate Specificity Studies of Xylanases from Marine Bacteria

Nitish Verma, Nils Rustmeier, Uwe Osswald, and Fabian Pfrengle*

Institute of Organic Chemistry, Department of Natural Sciences and Sustainable Resources,
University of Natural Resources and Life Sciences Vienna
Muthgasse 18, 1190 Vienna, Austria

*Corresponding author E-mail: *fabian.pfrengle@boku.ac.at*

Table of Contents

1. General methods 3

1.1 General method for the analytical HPLC of the crude reaction mixture obtained after the photocleavage reaction 4

Table S1. Analytical and semi-prep HPLC columns with their respective specifications. 4

Table S2. Analytical HPLC method (50 min) for YMC-Small and YMC-Diol normal phase (NP) columns. 4

Table S3. Analytical HPLC method (60 min) for YMC-Diol NP column. 4

1.2 Method for calculation of %yield of purified xylans after two deprotection steps 5

2. Synthesizer modules and conditions 6

3. Synthesis of xylose donor building blocks (BBs) 7

3.1 Synthesis of disarmed xylose donors 2 and 3 7

Scheme S1. Synthesis of disarmed Bz-protected xylose BBs **2** and **3**. 7

3.2 Synthesis of armed xylose donors S10 and 6 16

Scheme S2. Synthesis of armed Bn-protected xylose BBs **S10** and **6**. 16

4. Automated Glycan Assembly (AGA) 30

4.1 Optimization of AGA reactions for synthesis of β3-xylan di- and tetrasaccharides using disarmed donors. 30

Table S4. AGA of β3-xylan oligosaccharides using disarmed donors **2** and **3**. 30

Figure S1. HPLC-Chromatograms of crude reaction mixtures obtained after AGA of disaccharide **4** (in entries 1–5 of Table S4) using donor **2**. 31

Figure S2. HPLC-Chromatograms of crude reaction mixtures obtained after AGA of tetrasaccharide **5** (in entries 6–8 of Table S4) using donor **2**. 32

Figure S3. HPLC-Chromatograms of crude reaction mixtures obtained after AGA of disaccharide **4** (in entries 9–13 of Table S4) using donor **3**. 33

Figure S4. HPLC-Chromatogram of the crude reaction mixture after AGA of tetrasaccharide **5** using donor **2** (entry 7, Table S4). 34

Figure S5. Plot of disaccharide **4** absorption peak area (at λ = 280 nm) *vs* μmoles of **4** injected into the analytical HPLC-system. 35

Figure S6. HPLC-Chromatograms of four different concentrations of disaccharide **4** using a YMC-Small NP column with a gradient of EtOAc in hexanes (50 min, flow rate 1 mL/min). 36

4.2 AGA of β3-xylan oligosaccharides using Bn-protected armed xylose donors 55

4.3 AGA of mixed-linkage xylan (MLX) oligosaccharides 86

5. Extraction and purification of recombinant carbohydrate hydrolases 168

6. Xylanase assays and analysis of enzymatic degradation products using HPLC-MS 168

Table S5. LC/MS method using Hypercarb column. 168

Figure S7. Digests of synthesized β3-xylooligosaccharides by recombinant xylanases. 169

Figure S8. Digests of synthesized MLX oligosaccharides by recombinant xylanases. 171

Figure S9. Digests of natural MLX and synthesized β3-xylan decasaccharide (**14**) using recombinant xylanases. 171

7. References 172

1. General methods

All purchased chemicals were used without further purification. Solvents were dried over activated 4 Å molecular sieves. Aqueous solutions of salts were saturated unless stated otherwise. The concentration of organic solutions was performed under reduced pressure at 40 °C, unless stated otherwise. All reactions were monitored through thin layer chromatography (unless stated otherwise), which was performed on Merck pre-coated plates: generally, on 5 × 10 cm, layer thickness 0.25 mm, Silica Gel $60F_{254}$. High performance thin-layer chromatography was performed on silica gel $60F_{254}$ HPTLC precoated glass plates with a 25 mm concentration zone supplied by Merck. Spots were detected by a UV-lamp (254 nm) then by dipping reagent (anisaldehyde-H_2SO_4 or Hanessian´s stain) and heating at 250 °C using hotplate. Filtrations were performed using 25 mm syringe filters (PTFE, 0.45 µm) from Fischer Brand. Silica gel (0.040–0.063 mm, from Macherey-Nagel) was used for direct phase column chromatography, and purifications were performed either by hand or on the automatic system Interchim puriFlash 4125 or Interchim puriFlash 5.250. Liquid chromatography-mass spectrometry (LC-MS) analysis were performed on a Shimadzu LC10 system with Shimadzu 2020 mass spectrometer and Alltech ELSD 3300 (drift tube temperature = 60 °C, receiver gain = 2). NMR spectra were recorded on a Brucker 600'54 Ascend Evo with Prodigy CPP 1.1 BBO 600 S3 (600 MHz for ^{1}H, 151 MHz for decoupled ^{13}C), Brucker 300'54 Ascend ULH (300 MHz for ^{1}H, 76 MHz for decoupled ^{13}C), Bruker Avance III 600 instrument (600 MHz for ^{1}H, 151 MHz for decoupled ^{13}C), and a Bruker AVIII-HD (300 MHz for ^{1}H, 76 MHz for ^{13}C) using standard software provided by the manufacturer. ^{1}H spectra were referenced to 0 (external calibration to TMS) or 7.26 ppm for solutions in $CDCl_3$, 2.05 ppm for solutions in $(CD_3)_2CO$, 0 ppm (external calibration to DSS) for solutions in D_2O; ^{13}C spectra were referenced to 77.23 ppm for solutions in $CDCl_3$, 29.84 ppm for solutions in $(CD_3)_2CO$ and 67.19 ppm (external calibration to 1,4-dioxane) for solutions in D_2O. Assignments are based on 2D ^{1}H-^{1}H COSY, ^{1}H-^{13}C HSQC, ^{1}H-^{13}C CLIP-HSQC, ^{1}H-^{13}C HMBC, and ^{1}H-^{1}H TOCSY spectra. Peaks of the respective xylose residues in the ^{1}H NMR spectrum are labelled alphabetically (A, B, C, D, etc.) starting from the reducing end. ESI-HRMS data was obtained using samples dissolved in ACN/H_2O on an Agilent Technologies 6230B LCMS-TOF or a Waters Xevo G2-XS QTof instrument. Datasets were analyzed by Mass Hunter Qualitative Navigator B.08.00 software or mass-adducts were calculated with Mass Hunter Isotope Distribution Calculator v. 8.0.8208.0 software. The automated syntheses were performed on a Glyconeer 3.1 with software provided by the manufacturer. Linker-functionalized resin was purchased from GlycoUniverse.

1.1 General method for the analytical HPLC of the crude reaction mixture obtained after the photocleavage reaction

The obtained crude reaction mixture in DCM after the photocleavage reaction was concentrated under reduced pressure, redissolved in EtOAc, and passed through a pre-packed 0.5 g normal phase silica column (SI-S-500/6). Eluted EtOAc-fractions were concentrated under reduced pressure, and the crude mixture was re-dissolved in 3 mL toluene (unless stated otherwise) under sonication. The crude reaction mixture in toluene (70 µL) was then injected into the analytical HPLC-column.

Table S1. Analytical and semi-prep HPLC columns with their respective specifications.

	**Column type**	**Column specifications**
**Analytical HPLC**		
1	YMC-Small	YMC-Pack SIL-06 (250 × 4.6 mmI.D., S-5 µm, 6 nm)
2	YMC-Diol	YMC-Pack Diol-120-NP (150 × 4.6 mmI.D., S-5 µm, 12 nm)
**Semi-prep HPLC (for purification)**		
3	YMC-Small	YMC-Pack SIL-06 (250 × 10.0 mmI.D., S-5 µm, 6 nm)
4	YMC-Diol	YMC-Pack Diol-120-NP (250 × 10.0 mmI.D., S-5 µm, 12 nm)

Table S2. Analytical HPLC method (50 min) for YMC-Small and YMC-Diol normal phase (NP) columns.

**Time [min]**	**A (EtOAc%)**	**B (hexanes%)**	**Flow (mL/min)**	**Max. Pressure Limit [bar]**
0	20	80	1	400.00
5	20	80	1	…..
35	50	50	1	…..
40	55	45	1	…..
45	55	45	1	…..
50	20	80	1	…..

Table S3. Analytical HPLC method (60 min) for YMC-Diol NP column.

**Time [min]**	**A (EtOAc%)**	**B (hexanes%)**	**Flow (mL/min)**	**Max. Pressure Limit [bar]**
0	20	80	1	400.00
5	20	80	1	…..
35	50	50	1	…..
40	55	45	1	…..
55	55	45	1	…..
60	20	80	1	…..

This method was used for analytical HPLC of crude reaction mixtures obtained after AGA of MLX hexasaccharide **S21**, octasaccharide **S23**, and nonasaccharide **S24**.

1.2 Method for calculation of %yield of purified xylans after two deprotection steps

Unprotected xylan oligosaccharides were isolated in the salt form with acetate as a counter ion. Yield of unprotected xylans were precisely calculated by determining the amount of acetate present in the NMR sample. Please see the following standard equations for calculating the yield.

$Wt._A + Wt._B = x$ mg (known weight after the lyophilization)

$n_A/n_B = 1/(I_B/3) = y$ (known number from the ^{1}H NMR spectrum)

$n_B = n_A/y$

In general, $n = Wt./M.Wt.$

therefore, $Wt. = n \times M.Wt.$

$n_A \times M.Wt._A + n_B \times M.Wt._B = x$ mg

$n_A \times M.Wt._A + (n_A/y) \times M.Wt._B = x$ mg

By solving the above equation, the defined value of n_A was calculated.

$$\% \text{ yield} = \frac{nA}{nSM} \text{ X } 100$$

$Wt._A$ = weight of the desired xylan with linker in free NH_2 form

$M.Wt._A$ = molecular weight of the desired xylan with linker in free NH_2 form

n_A = moles of the desired xylan with linker in free NH_2 form

$Wt._B$ = weight of the AcOH

$M.Wt._B$ = molecular weight of the AcOH = 60.052 g/mol

n_B = moles of the AcOH

I_B = integration value of the CH_3 of the AcOH at ~1.89 ppm in the ^{1}H NMR spectrum

nSM = no. of moles of the starting material (protected xylan)

2. Synthesizer modules and conditions

Linker-functionalized resin **1** (12.5 µmol of hydroxyl groups) was placed in the reaction vessel of the automated oligosaccharide synthesizer and swollen for at least 30 min in DCM. Before every reaction step, the resin was washed with DMF and DCM. Subsequently, the glycosylation (Module A), capping (Module B) and Fmoc deprotection (Module C) steps were performed. Mixing of the components was accomplished by bubbling Argon through the reaction mixture. The settings for the different modules in the `Glycomerge´ program were used as provided by GlycoUniverse.

Module A1: Glycosylation with glycosyl phosphates

Resin **1** (12.5 µmol of hydroxyl groups) was swollen in DCM and the temperature of the reaction vessel was adjusted according to each AGA reaction (please see experimental procedure). Prior to the glycosylation reaction, the resin was washed with 62 mM TMSOTf in DCM and then DCM only. For the glycosylation reaction, the DCM was drained, and a solution of phosphate BB (5 equiv., 60 mM DCM) was delivered to the reaction vessel at low temperature (see individual reaction). The reaction was initiated by the addition of 62 mM TMSOTf in DCM (1 mL). The glycosylation was performed for t1 (min) at T1 °C and then for t2 (min) at T2 (°C). Subsequently, the solution was drained, and the resin was washed three times with DCM at 25 °C.

Module A2: Glycosylation with thioglycosides

The resin **1** (12.5 µmol of hydroxyl groups) was swollen in DCM and the temperature of the reaction vessel was adjusted to −20 °C. Prior to the glycosylation reaction, the resin was washed with 62 mM TMSOTf in DCM and then DCM only. For the glycosylation reaction, the DCM was drained and a solution of thioglycoside BB (1 mL; 6.5 equiv., 80.0 mM in DCM) was delivered to the reaction vessel. After the set temperature was reached, the reaction was started by the dropwise addition of the activator solution (1 mL; 0.15 M NIS/17 mM TfOH in DCM/dioxane = 2/1). The glycosylation was performed for t1 (min) at T1 (°C) and then for t2 (min) at T2 (°C). Subsequently, the solution was drained and the resin was washed with DCM (3 mL), DCM:dioxane (1:1, 3 mL) and DCM (two times, each with 3 mL). The temperature of the reaction vessel was increased to 25 °C for the next module.

Module B: Capping (two cycles per glycosylation)

The temperature of the reaction vessel was adjusted to 30 °C. 10% pyridine in dry DMF (2 mL) was delivered. After 3 min, the reaction solution was drained, and the resin was washed with DCM (three times with 3 mL). Then a solution of 10% acetic anhydride and 2% methanesulfonic acid in DCM (2 mL) was delivered to the reaction vessel. After 10 min, the solution was drained, and the resin was washed with DCM (three times with 3 mL).

Module C: Fmoc deprotection (one cycle per glycosylation)

The resin was washed with DMF, swollen in DMF, and the temperature of the reaction vessel was adjusted to 20 °C. Prior to the deprotection reaction, DMF was drained, and the resin was washed with DMF three times. For Fmoc deprotection, 2 mL of a solution of 20% piperidine in DMF was delivered to the reaction vessel. After 10 min, the reaction solution was drained, and the resin was washed with DMF (three times with 3 mL) and DCM (three times each with 3 mL). The temperature of the reaction vessel was decreased to −20 °C for the next step.

Cleavage from solid support

After assembly of the oligosaccharides, cleavage from solid support was accomplished by UV irradiation at 305 nm in a continuous flow photoreactor (photocleavage device: Easy-PhotoChem V3 UV-150 from Vapourtec E-series with attached syringe pump) as previously described.[1]

3. Synthesis of xylose donor building blocks (BBs)

3.1 Synthesis of disarmed xylose donors 2 and 3

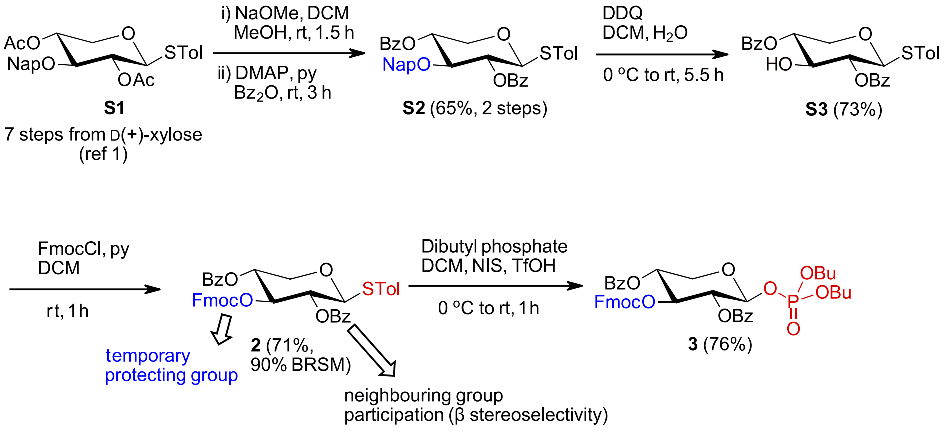

Scheme S1. Synthesis of disarmed Bz-protected xylose BBs **2** and **3**.

4-Methylphenyl 2,4-di-*O*-benzoyl-3-*O*-(2-methyl)naphtyl-1-thio-β-D-xylopyranoside (S2)

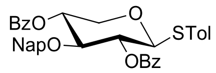

S2

Experimental procedure: To a solution of starting material **S1**[1] (235 mg, 0.49 mmol) in a mixture of DCM (850 µL, 0.58 M) and MeOH (850 µL, 0.58 M) was added NaOMe (0.5 M in MeOH, 176 µL, 88 µmol) at rt, and the reaction mixture was allowed to stir for 1.5 h. After confirmation of reaction completion by TLC, the reaction mixture was neutralized by the addition of IR-120 H^+ resin while slowly stirring the mixture. The reaction mixture was filtered using filter paper, and the filtrate was concentrated under reduced pressure to yield a viscous pale-yellow crude, which was used in the next step without further purification. To a solution of the crude intermediate in anhydrous pyridine (2.44 mL, 0.2 M), DMAP (17.9 mg, 146 µmol) and Bz_2O (442 mg, 1.95 mmol) were added at rt under Ar-atmosphere. After 3 h of stirring, the reaction mixture was quenched by slow addition of MeOH (5 mL) under cooling with an ice bath and concentrated under reduced pressure to yield a viscous residue. The residue was dissolved in DCM (7 mL) and washed sequentially with 2M HCl (5 mL) and saturated aqueous $NaHCO_3$ solution (5 mL, two times). The DCM layer was collected, dried over anhydrous Na_2SO_4, filtered, and concentrated under reduced pressure to yield a crude product. The crude was purified by silica gel column chromatography (EtOAc/hexanes = 1/4, v/v) to yield product **S2** (192 mg, 65% yield over 2 steps) as a white amorphous solid.

***R_f*:** 0.74 (EtOAc/hexanes = 1/2, v/v).

^{1}H NMR (300 MHz, $CDCl_3$): δ 8.03-7.94 (m, 4H, Ar-H), 7.81-7.69 (m, 4H, Ar-H), 7.57-7.50 (m, 2H, Ar-H), 7.48-7.41 (m, 5H, Ar-H), 7.36-7.27 (m, 4H, Ar-H), 7.16-7.10 (m, 2H, Ar-H), 5.47 (t, J = 4.7 Hz, 1H, H-2), 5.30 (d, J = 4.4 Hz, 1H, H-1), 5.26-5.20 (m, 1H, H-4), 5.0 (d, J = 11.9 Hz, 1H, CH_2-Nap), 4.92 (d, J = 12.0 Hz, 1H, CH_2-Nap), 4.74 (dd, J = 12.5, 3.3 Hz, 1H, H-5a), 4.12 (t, J = 5.0 Hz, 1H, H-3), 3.78 (dd, J = 12.4, 4.8 Hz, 1H, H-5b), 2.33 (s, 3H, Ph-CH_3) ppm.

^{13}C NMR (76 MHz, $CDCl_3$): δ 165.85, 165.49, 138.1, 135.0, 133.46, 133.45, 133.36, 133.22, 132.7, 131.0, 130.1, 130.0, 129.94, 129.74, 129.67, 128.53, 128.51, 128.46, 128.16, 127.9, 126.91, 126.24, 126.10, 125.9, 86.7, 75.0, 73.5, 70.7, 69.7, 62.0, 21.3 ppm.

ESI-HRMS: m/z $[M + NH_4]^+$ calcd. for $C_{37}H_{36}NO_6S$: 622.2258; found 622.2275.

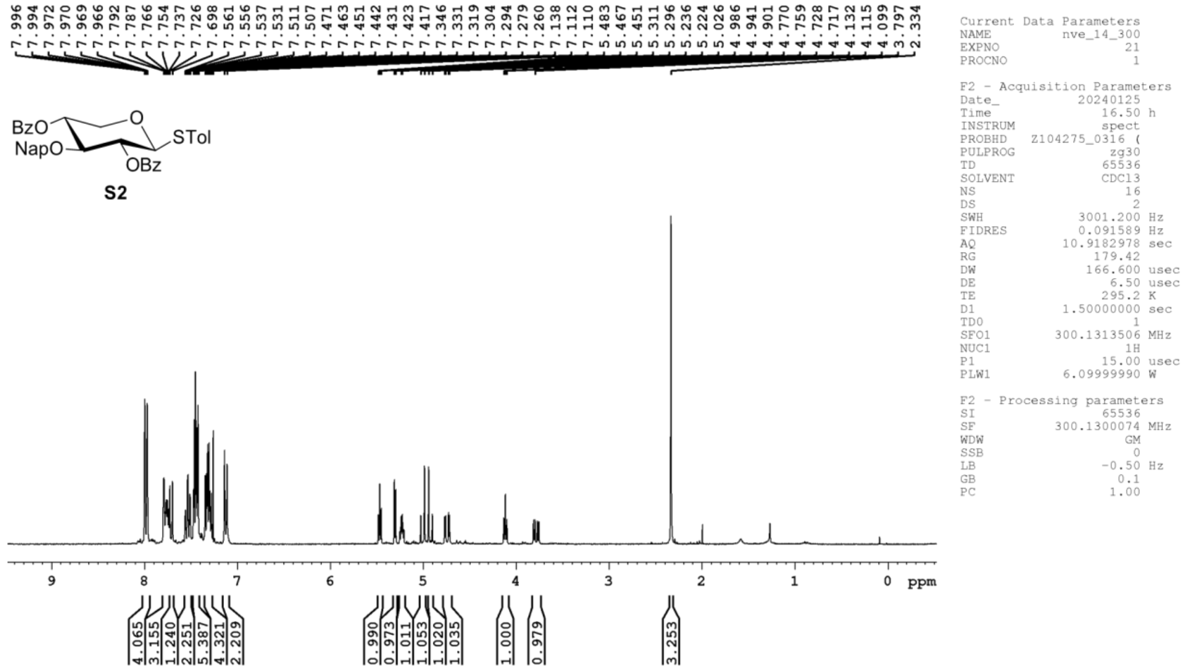

^{1}H NMR spectrum of compound **S2** (300 MHz, $CDCl_3$)

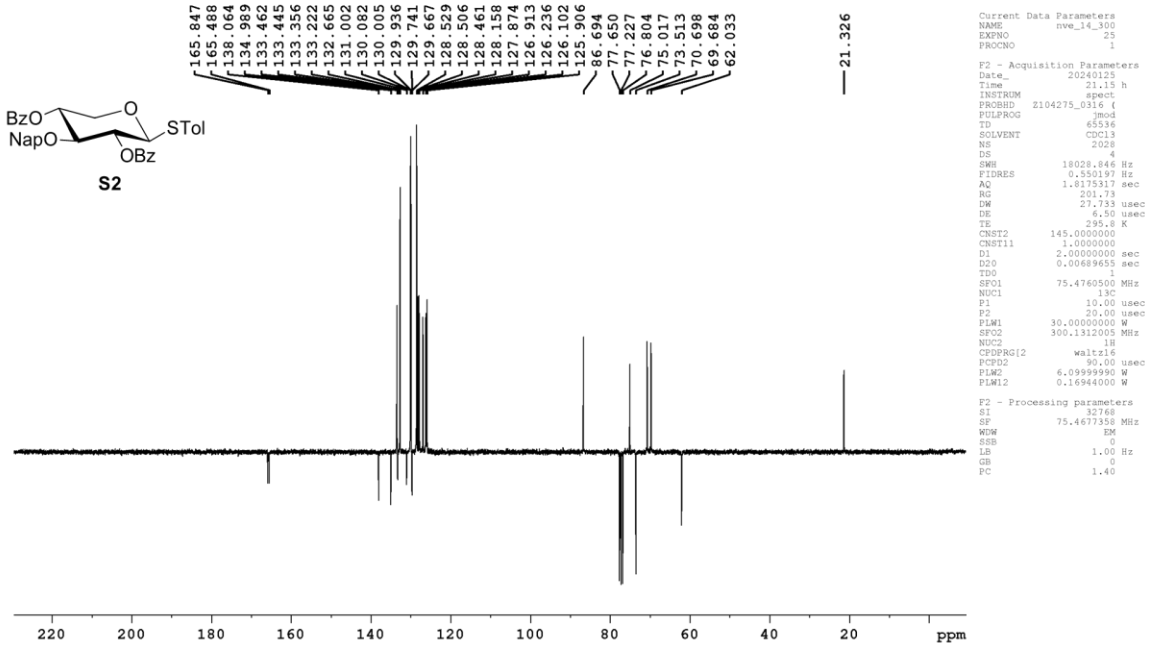

^{13}C APT NMR spectrum of compound **S2** (76 MHz, $CDCl_3$)

4-Methylphenyl 2,4-di-*O*-benzoyl-1-thio-β-D-xylopyranoside (S3)

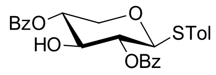

S3

Experimental procedure: To a solution of **S2** (166 mg, 275 μmol) in a 10:1 v/v mixture of DCM (25 mL) and H_2O (2.5 mL), DDQ (187 mg, 834 μmol) was added at 0 ºC under Ar-atmosphere. The reaction mixture was continuously stirred and gradually allowed to reach rt without removing the ice bath. After confirmation of reaction completion (5.5 h) by TLC, the reaction mixture was quenched by slow addition of saturated aqueous $NaHCO_3$ solution (10 mL) under cooling with the ice bath. The DCM layer was collected, washed with saturated aqueous $NaHCO_3$ solution (20 mL), dried over anhydrous Na_2SO_4, filtered, and concentrated under reduced pressure to yield a yellow viscous crude. The crude was subjected to purification by silica gel column chromatography (EtOAc/hexanes = 1/5 to 1/4, v/v) to afford product **S3** (93 mg, 73% yield) as a white solid.

***R_f*:** 0.39 (EtOAc/hexanes = 1/3, v/v).

^{1}H NMR (600 MHz, $CDCl_3$): *δ* 8.07-7.99 (m, 4H, Ar-H), 7.6-7.53 (m, 2H, Ar-H), 7.42-7.36 (m, 6H, Ar-H), 7.11 (d, *J* = 8.2 Hz, 2H, Ar-H), 5.15-5.09 (m, 2H, H-2, H-4), 4.99 (d, *J* = 7.4 Hz, 1H, H-1), 4.48 (dd, *J* = 11.9, 4.5 Hz, 1H, H-5a), 4.16 (td, *J* = 7.4, 5.3 Hz, 1H, H-3), 3.60 (dd, *J* = 11.9, 7.9 Hz, 1H, H-5b), 3.01 (s, 1H, O-H), 2.33 (s, 3H, Ph-CH_3) ppm.

^{13}C NMR (151 MHz, $CDCl_3$): *δ* 166.3, 138.69, 138.64, 133.64, 133.59, 133.56, 130.23, 130.07, 129.99, 129.64, 129.61, 128.63, 128.61, 86.5, 73.2, 72.71, 72.44, 71.80, 71.75, 64.83, 64.64, 21.4 ppm.

ESI-HRMS: m/z $[M + HCOO]^-$ calcd. for $C_{27}H_{25}O_8S$: 509.1276; found 509.1270.

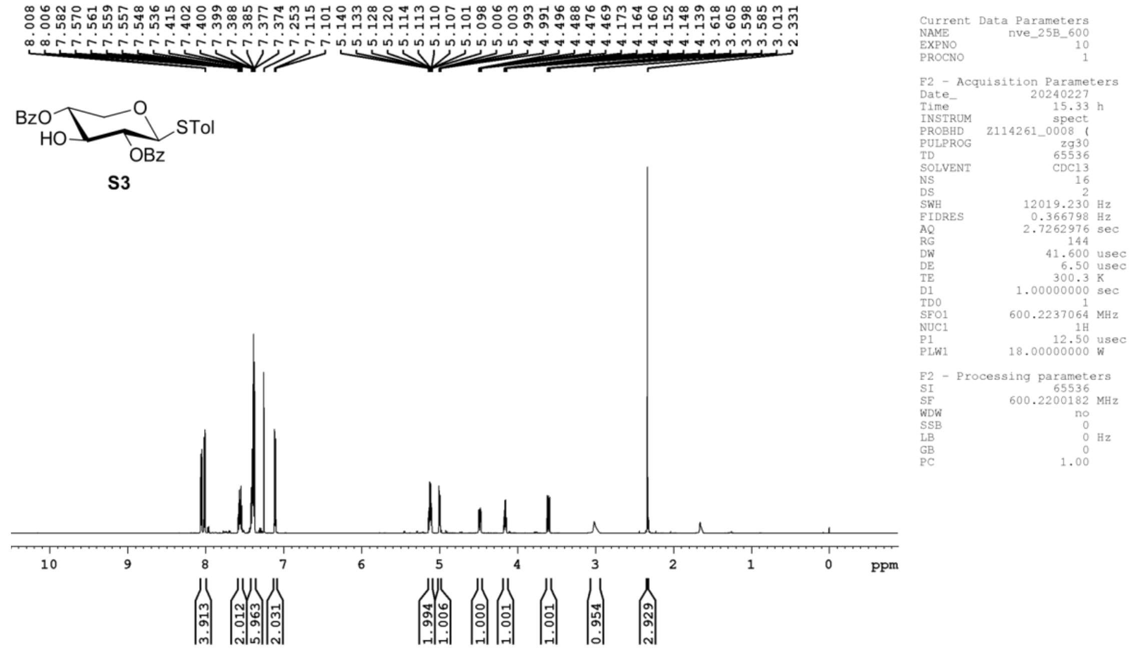

^{1}H NMR spectrum of compound **S3** (600 MHz, $CDCl_3$)

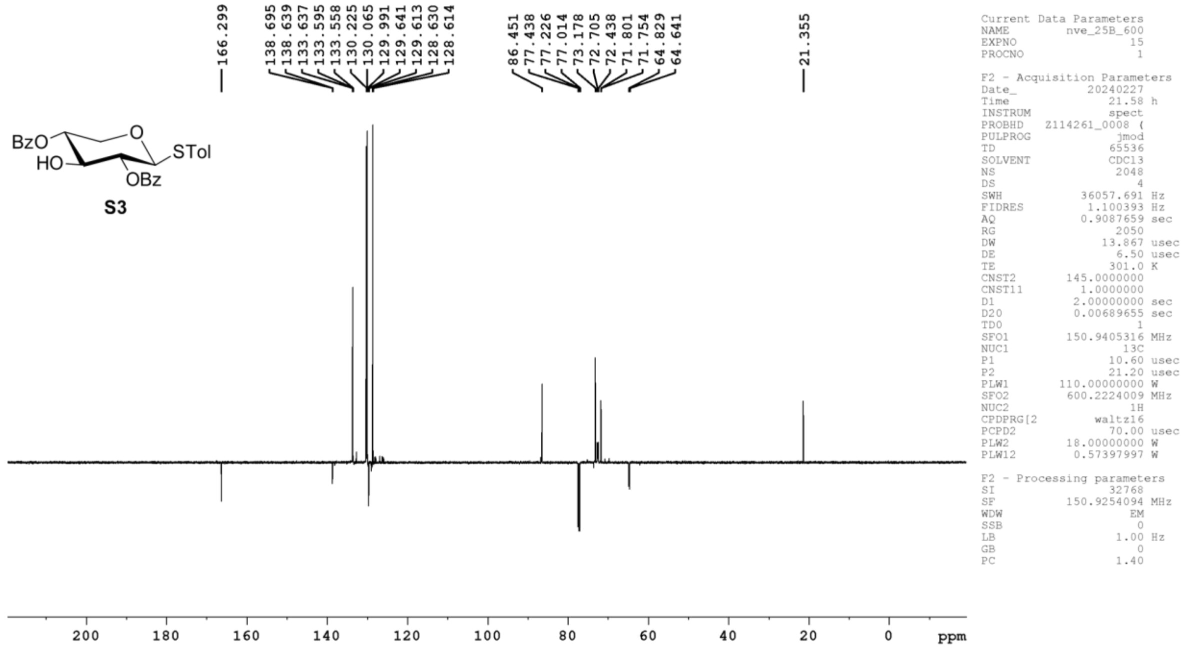

^{13}C APT NMR spectrum of compound **S3** (151 MHz, $CDCl_3$)

4-Methylphenyl 2,4-di-*O*-benzoyl-3-*O*-fluorenylcarboxymethyl-1-thio-β-D-xylopyranoside (2)

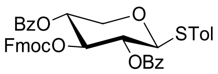

2

Experimental procedure: To a solution of starting **S3** (4.62 g, 9.95 mmol) in a 1:1 v/v mixture of DCM (33 mL) and pyridine (33 mL) was added FmocCl (3.35 g, 12.9 mmol) at 0 °C under Ar-atmosphere. After 10 min, the ice bath was removed. After confirmation of reaction completion (1 h 15 min) by TLC, the reaction mixture was diluted with DCM (20 mL) and quenched by slow addition of 2M-HCl solution (40 mL) under cooling with the ice bath. Then, the DCM layer was collected and sequentially washed with 2M-aqueous HCl solution (40 mL) and saturated aqueous $NaHCO_3$ solution (40 mL). The DCM layer was collected, dried over anhydrous Na_2SO_4, filtered, and concentrated under reduced pressure to yield a viscous pale-yellow crude. The crude was subjected to purification by silica gel column chromatography (EtOAc/hexanes = 1/5 to 1/4, v/v) to afford product **2** (4.82 g, 71% isolated yield, 90% brsm yield) as a white amorphous solid.

***R_f*:** 0.47 (EtOAc/hexanes = 1/3, v/v).

^{1}H NMR (300 MHz, $CDCl_3$): δ 8.09-7.97 (m, 4H, Ar-H), 7.72-7.66 (m, 2H, Ar-H), 7.58-7.50 (m, 2H, Ar-H), 7.48-7.43 (m, 3H, Ar-H), 7.42-7.39 (m, 2H, Ar-H), 7.38-7.29 (m, 5H, Ar-H), 7.20-7.12 (m, 4H, Ar-H), 5.5-5.36 (m, 2H, H-2, H-3), 5.31 (td, J = 8.0, 4.7 Hz, 1H, H-4), 5.05 (d, J = 7.6 Hz, 1H, H-1), 4.59 (dd, J = 11.8, 4.7 Hz, 1H, H-5a), 4.32-4.17 (m, 2H, CH_2-Fmoc), 4.03 (t, J = 7.6 Hz, 1H, CH-Fmoc), 3.66 (dd, J = 11.8, 8.2 Hz, 1H, H-5b), 2.36 (s, 3H, Ph-CH_3) ppm.

^{13}C NMR (76 MHz, $CDCl_3$): δ 165.55, 165.16, 154.6, 143.24, 143.16, 141.28, 141.24, 138.8, 133.71, 133.63, 130.19, 130.07, 130.01, 129.36, 129.18, 128.65, 128.61, 127.96, 127.31, 125.29, 125.28, 120.1, 86.9, 75.9, 70.75, 70.32, 69.3, 65.2, 46.6, 21.4 ppm.

ESI-HRMS: m/z $[M + Na]^+$ calcd. for $C_{41}H_{34}O_8SNa$: 709.1867; found 709.1882.

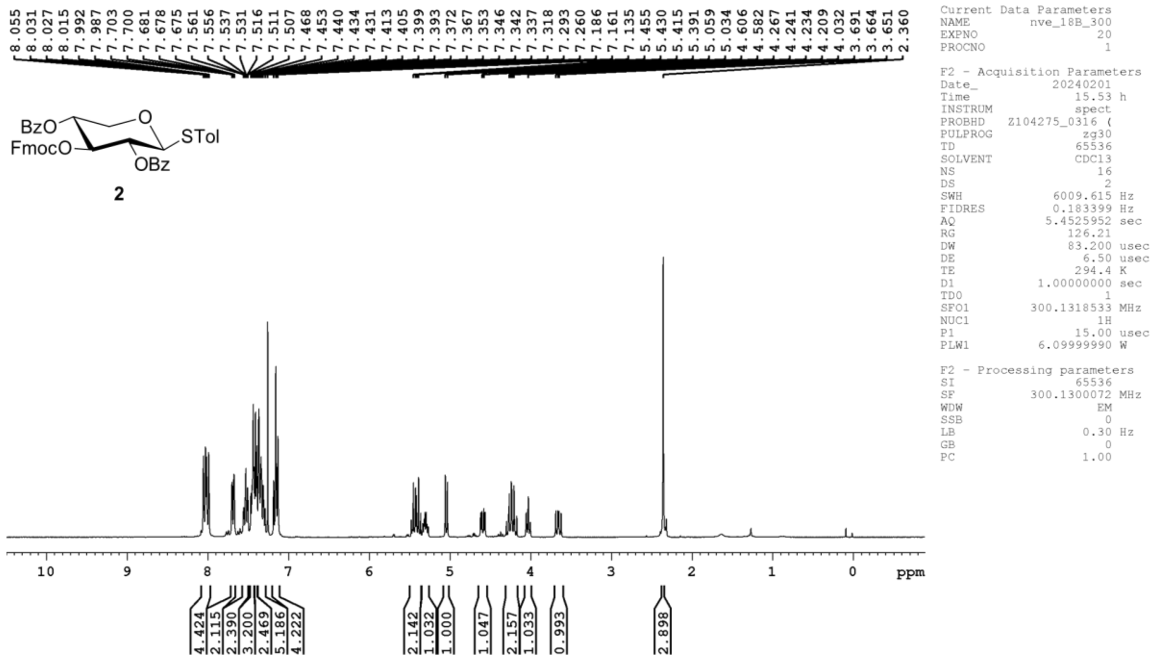

^{1}H NMR spectrum of compound **2** (300 MHz, $CDCl_3$)

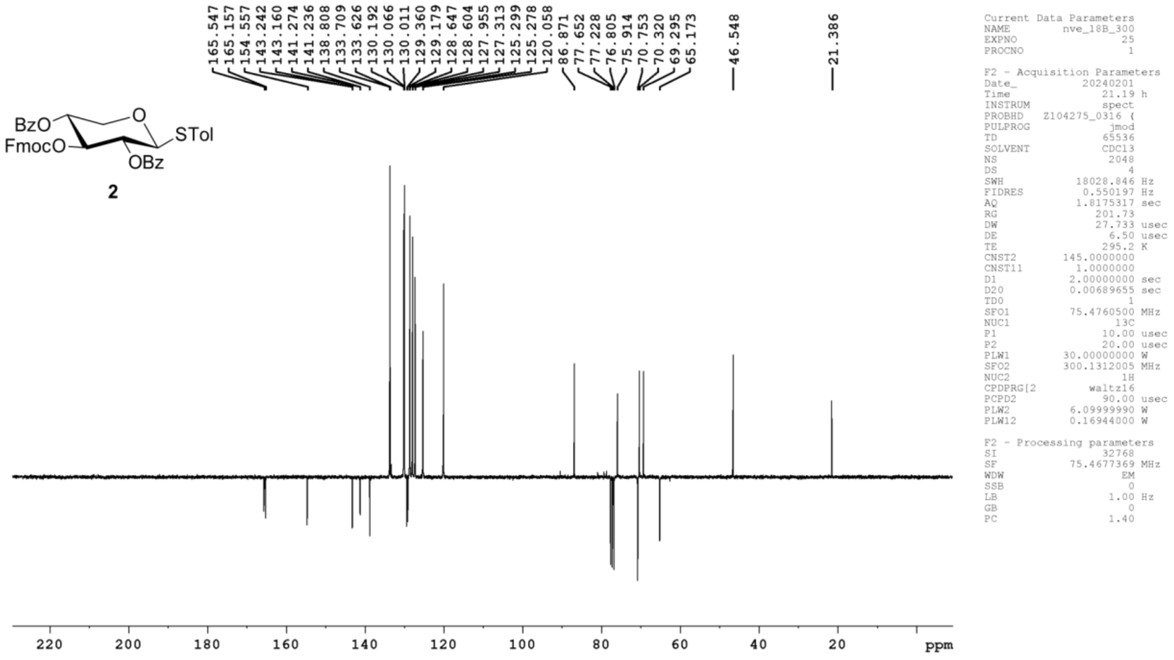

^{13}C APT NMR spectrum of compound **2** (76 MHz, $CDCl_3$)

Dibutoxyphosphoryloxy 2,4-di-*O*-benzoyl-3-*O*-fluorenylcarboxymethyl-β-D-xylopyranoside (3)

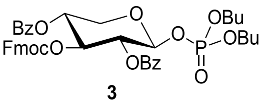

3

Experimental procedure: A solution of thioxyloside **2** (912 mg, 1.33 mmol) and dibutyl hydrogen phosphate (669 µL, 3.37 mmol) in anhydrous DCM (6.64 mL) was stirred with 4 Å pulverized molecular sieves (1.1 g) at rt for 45 min under Ar-atmosphere. After cooling to 0 °C, NIS (recrystallized, 359 mg, 1.59 mmol) and TfOH (35.2 µL, 0.4 mmol) were added to the reaction mixture. After 1 h, the reaction was quenched by dropwise addition of Et_3N (111 µL, 0.6 equiv.) to neutralize the pH of reaction solution, diluted with DCM (10 mL), and filtered using suction filtration over a sintered funnel. The filtrate was sequentially washed with 25% (w/v) aqueous $Na_2S_2O_3$ solution (15 mL) and saturated aqueous $NaHCO_3$ solution (15 mL), dried over Na_2SO_4, filtered, and concentrated under reduced pressure to afford a yellow viscous residue. The crude was subjected to purification by silica gel column chromatography (EtOAc/hexanes = 1/3 to 1/2, v/v) to afford product **3** (781 mg, 76% yield) as a white sticky foam.

***R_f*:** 0.29 (EtOAc/hexanes = 1/3, v/v, run two times).

^{1}H NMR (300 MHz, $CDCl_3$, β-anomer): δ 8.05-7.97 (m, 4H, Ar-H), 7.72-7.66 (m, 2H, Ar-H), 7.57-7.49 (m, 2H, Ar-H), 7.47-7.38 (m, 3H, Ar-H), 7.37-7.29 (m, 5H, Ar-H), 7.20-7.12 (m, 2H, Ar-H), 5.61 (dd, J = 6.9, 5.8 Hz, 1H, H-1), 5.51-5.29 (m, 3H, H-2, H-3, H-4), 4.48 (dd, J = 12.2, 4.4 Hz, 1H, H-5a), 4.30-4.23 (m, 2H, CH_2-Fmoc), 4.13-4.01 (m, 3H, OBu, CH-Fmoc), 3.93-3.81 (m, 2H, OBu), 3.76 (dd, J = 12.2, 7.3 Hz, 1H, H-5b), 1.73-1.61 (m, 2H, Bu), 1.47-1.35 (m, 4H, Bu), 1.21-1.10 (m, 2H, Bu), 0.93 (t, J = 7.4 Hz, 3H, CH_3), 0.76 (t, J = 7.3 Hz, 3H, CH_3) ppm.

^{13}C NMR (76 MHz, $CDCl_3$): δ 165.60, 165.04, 154.5, 143.24, 143.16, 141.32, 141.29, 133.81, 133.79, 130.22, 130.13, 129.1, 128.98, 128.68, 128.00, 127.3, 125.24, 125.23, 120.1, 96.6 (d, J = 4.92 Hz, C-1), 74.2, 70.80, 70.51, 70.38, 68.87, 68.38, 68.29, 68.20, 62.4, 46.6, 32.34, 32.25, 32.15, 32.06, 18.79, 18.56, 13.75, 13.61 ppm.

ESI-HRMS: m/z $[M + Na]^+$ calcd. for $C_{42}H_{45}O_{12}PNa$: 795.2541; found 795.2541.

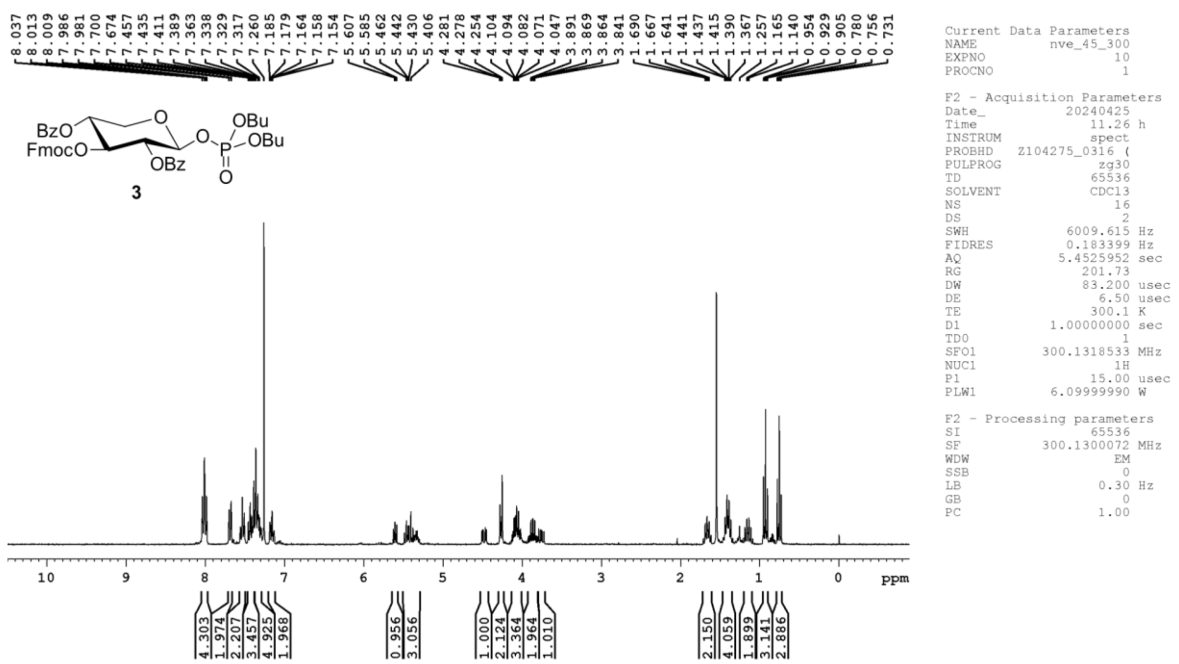

^{1}H NMR spectrum of compound **3** (300 MHz, $CDCl_3$)

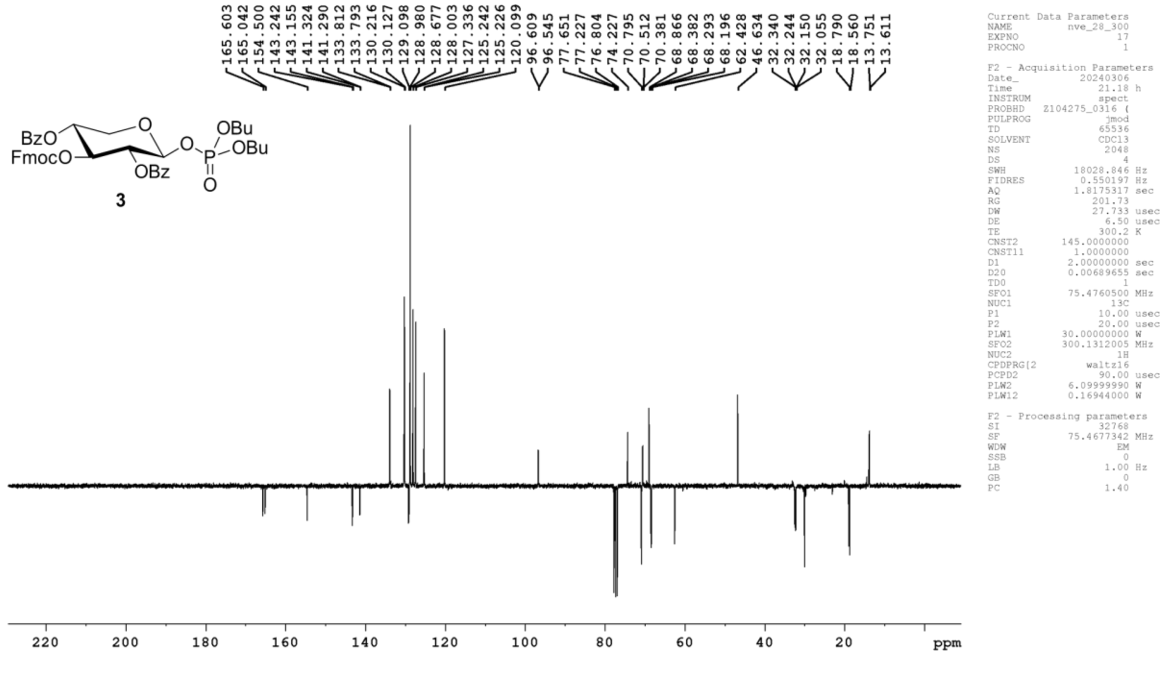

^{13}C APT NMR spectrum of compound **3** (76 MHz, $CDCl_3$)

3.2 Synthesis of armed xylose donors S10 and 6

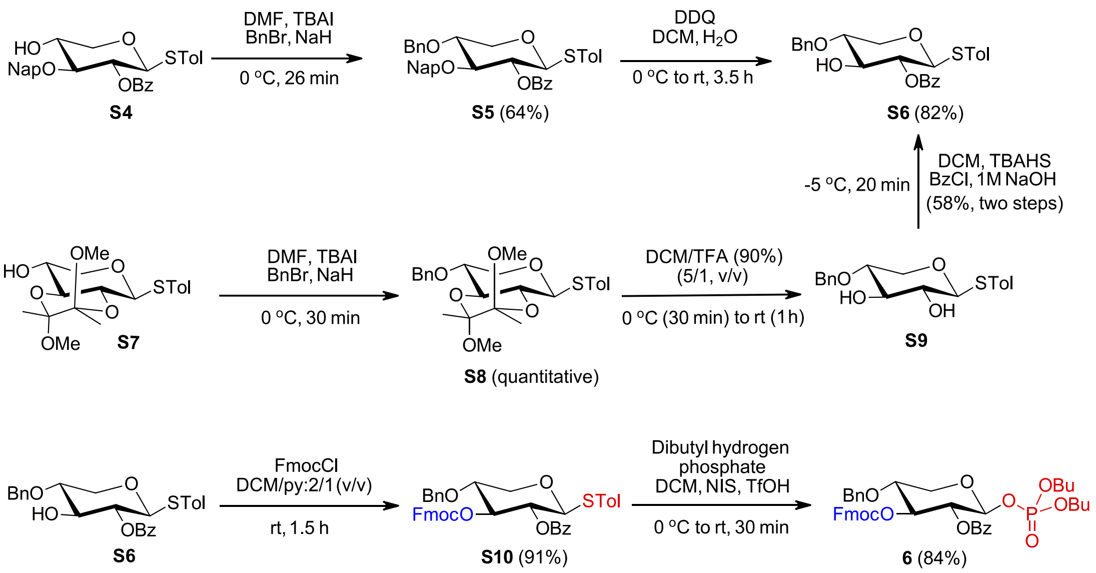

Scheme S2. Synthesis of armed Bn-protected xylose BBs **S10** and **6**.

Route A: synthesis of compound **S6** via compound **S4** from commercially available D(+)-xylose: 10% total yield in 12 steps.[1]

Route B: synthesis of compound **S6** via compound **S7** from commercially available D(+)-xylose: 12% total yield in 7 steps.[2]

Route B for chemical synthesis of compound **S6** is relatively more efficient than route A in terms of total reaction time and total yield of the desired product.

4-Methylphenyl 2-*O*-benzoyl-3-*O*-(2-methyl)naphtyl-4-*O*-benzyl-1-thio-β-D-xylopyranoside (S5)

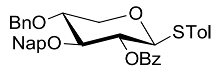

S5

Etherification of S4 using NAH and BnBr:

Experimental procedure: To a solution of **S4**[1] (20 mg, 40 µmol) in anhydrous DMF (0.45 mL) were added TBAI (3 mg, 8 µmol), BnBr (5.2 µL, 44 µmol), and NaH (2.6 mg, 64 µmol) at 0 ºC. The reaction mixture was vigorously stirred at 0 °C. After 26 min, the reaction was quenched by dropwise addition of water under cooling with an ice bath, diluted with EtOAc (5 mL), and washed with saturated aqueous $NaHCO_3$ solution (5 mL). The EtOAc layer was collected, dried over anhydrous Na_2SO_4, filtered, and concentrated under reduced pressure to yield a viscous pale-yellow crude. The crude was subjected to purification by silica gel column chromatography (EtOAc/hexanes = 1/7, v/v) to afford product **S5** (15 mg, 64% yield) as a white amorphous solid.

Etherification of S4 using Ag_2O and BnBr:

Experimental procedure: To a solution of **S4** (20 mg, 40 µmol) in DMF (0.1 mL) was added Ag_2O (21.3 mg, 92 µmol) and BnBr (15 µL, 0.13 mmol) at rt under Ar-atmosphere. The reaction mixture was vigorously stirred. After 24 h, the reaction mixture was diluted with EtOAc (5 mL) and washed with saturated aqueous $NaHCO_3$ solution (5 mL, two times). The EtOAc layer was collected, dried over anhydrous Na_2SO_4, filtered, and concentrated under reduced pressure to yield a viscous pale-yellow crude. The crude was subjected to purification by silica gel column chromatography (EtOAc/hexanes = 1/7, v/v) to afford product **S5** (15 mg, 64% yield) as a white amorphous solid.

***R_f*:** 0.56 (EtOAc/hexanes = 1/3, v/v).

^{1}H NMR (300 MHz, $CDCl_3$): δ 7.99-7.92 (m, 2H, Ar-H), 7.73-7.67 (m, 1H, Ar-H), 7.66-7.59 (m, 2H, Ar-H), 7.57-7.50 (m, 2H, Ar-H), 7.44-7.26 (m, 12H, Ar-H), 7.10-7.04 (m, 2H, Ar-H), 5.26 (q, J = 7.0 Hz, 1H, H-2), 4.92 (d, J = 11.7 Hz, 1H, CH_2), 4.85 (d, J = 11.7 Hz, 1H, CH_2), 4.80 (d, J = 8.5 Hz, 1H, H-1), 4.71 (d, J = 11.6 Hz, 1H, CH_2), 4.63 (d, J = 11.6 Hz, 1H, CH_2), 4.21 (dd, J = 11.7, 4.5 Hz, 1H, H-5a), 3.81 (t, J = 7.8 Hz, 1H, H-3), 3.77-3.69 (m, 1H, H-4), 3.41 (dd, J = 11.7, 8.7 Hz, 1H, H-5b), 2.30 (s, 3H, Ph-CH_3) ppm.

^{13}C NMR (76 MHz, $CDCl_3$): δ 165.5, 138.24, 138.12, 135.5, 133.30, 133.17, 133.12, 130.03, 129.85, 129.64, 128.70, 128.51, 128.30, 128.13, 128.08, 128.06, 127.84, 127.06, 126.28, 126.12, 125.98, 87.4, 81.0, 77.3, 75.0, 73.3, 71.8, 66.7, 21.3 ppm.

ESI-HRMS: m/z $[M + H]^+$ calcd. for $C_{37}H_{35}O_5S$: 591.2200; found 591.2203.

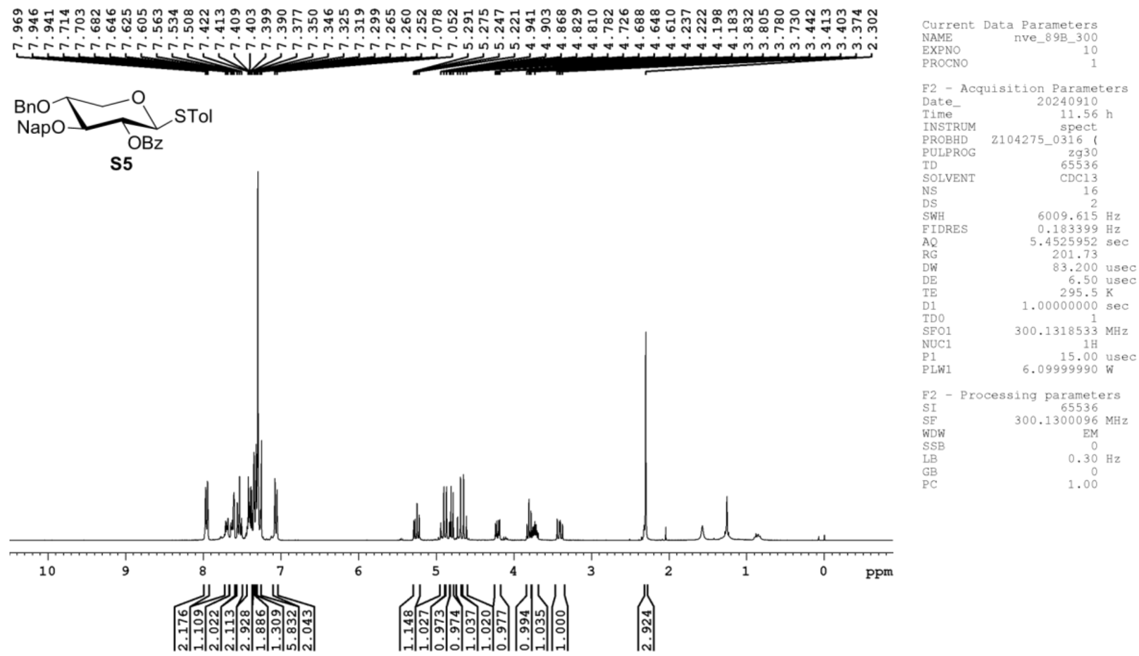

^{1}H NMR spectrum of compound **S5** (300 MHz, $CDCl_3$)

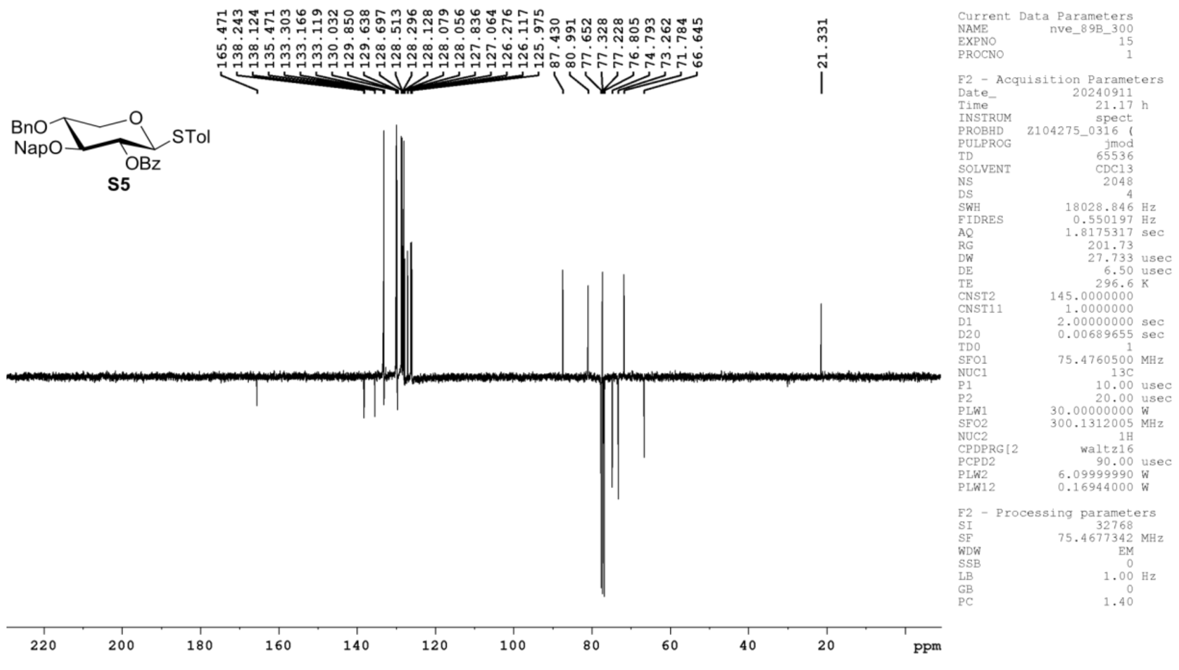

^{13}C APT NMR spectrum of compound **S5** (76 MHz, $CDCl_3$)

4-Methylphenyl 2,3-*O*-(2,3-dimethoxybutane-2,3-diyl)-1-thio-β-D-xylopyranoside (S7)

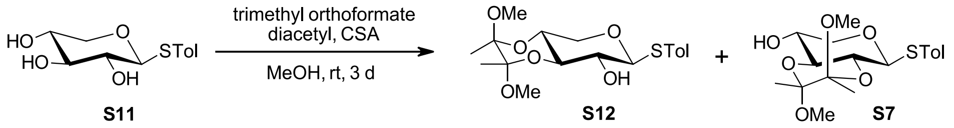

To a solution of starting material **S11**[2] (10.0 g, 39.0 mmol) in anhydrous MeOH (136 mL), trimethyl orthoformate (21 mL, 195 mmol) and diacetyl (6.8 mL, 78.0 mmol) were added. Then a catalytic amount of CSA (0.92 g, 3.96 mmol) was added in one portion. The mixture was stirred for 3 d at rt. After quenching the reaction with Et_3N (3 mL), the solution was concentrated under reduced pressure to a deep red crude which was purified by silica gel column chromatography (toluene/EtOAc = 6/1 to 3/1) to yield literature known[2] 2-alcohol **S12** (10.9 g, 75%) as pale yellow solid with small amounts of diacetyl related impurities and 4-alcohol **S7** (4.0 g, 28%) as pale yellow solid (used in the next step without further purification). A small amount of 4-alcohol **S7** was further purified by silica gel column chromatography (EtOAc/hexanes = 1/4) to yield the main BBA-diastereomer pure for characterization.

R**_f_:** 0.40 (toluene/EtOAc = 2/1, v/v, ran in HPTLC).

^{1}H NMR (600 MHz, $CDCl_3$): δ 7.47-7.36 (m, 2 H, Ar-H), 7.15-7.03 (m, 2 H, Ar-H), 4.68 (d, J = 9.8 Hz, 1 H, H-1), 4.08 (dd, J = 11.5, 5.3 Hz, 1 H, H-5a), 3.93-3.83 (m, 1 H, H-4), 3.67 (t, J = 9.5 Hz, 1 H, H-3), 3.57 (t, J = 9.7 Hz, 1 H, H-2), 3.30-3.24 (m, 4 H, OCH_3^{BBA}, H-5b), 3.23 (s, 3 H, OCH_3^{BBA}), 2.41-2.34 (m, 1 H, O-H), 2.32 (s, 3 H, Ph-CH_3), 1.34 (s, 3 H, CH_3), 1.33 (s, 3 H, CH_3’).

^{13}C NMR (151 MHz, $CDCl_3$): δ 137.9, 132.7, 129.76, 129.42, 100.3, 99.7, 86.5, 75.0, 69.8, 68.2, 67.4, 48.35, 48.09, 21.2, 17.8, 17.7.

ESI-HRMS: m/z [M - OMe]$^+$ calcd. for $C_{17}H_{23}O_5S$: 339.1261; found 339.1268.

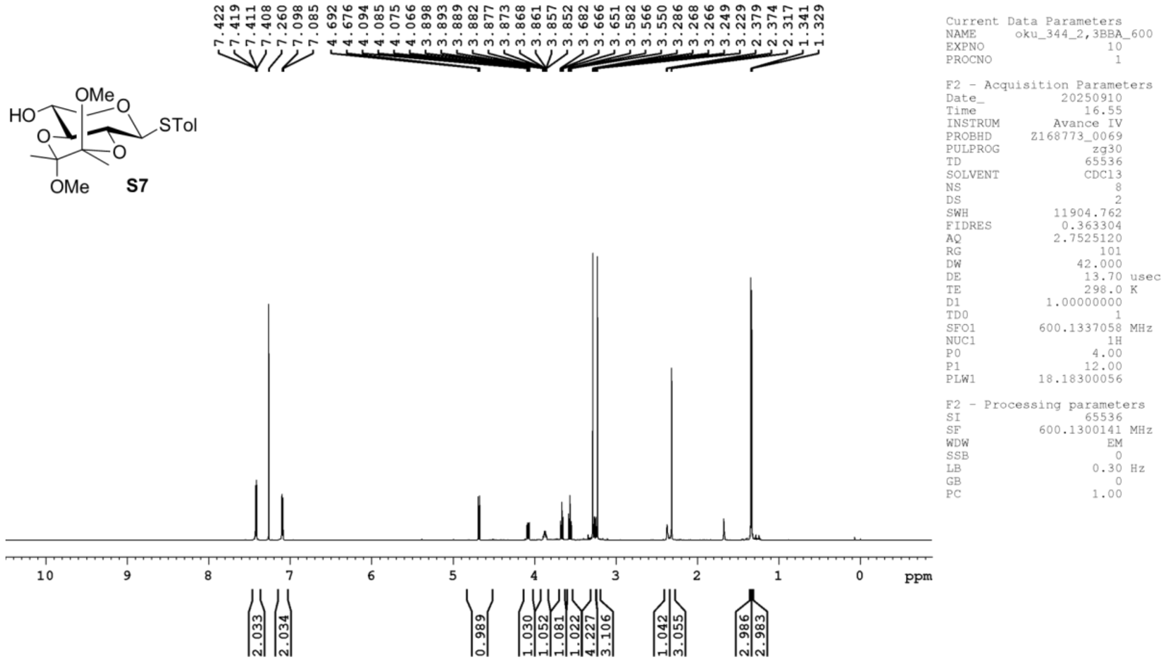

^{1}H NMR spectrum of compound **S7** (600 MHz, $CDCl_3$)

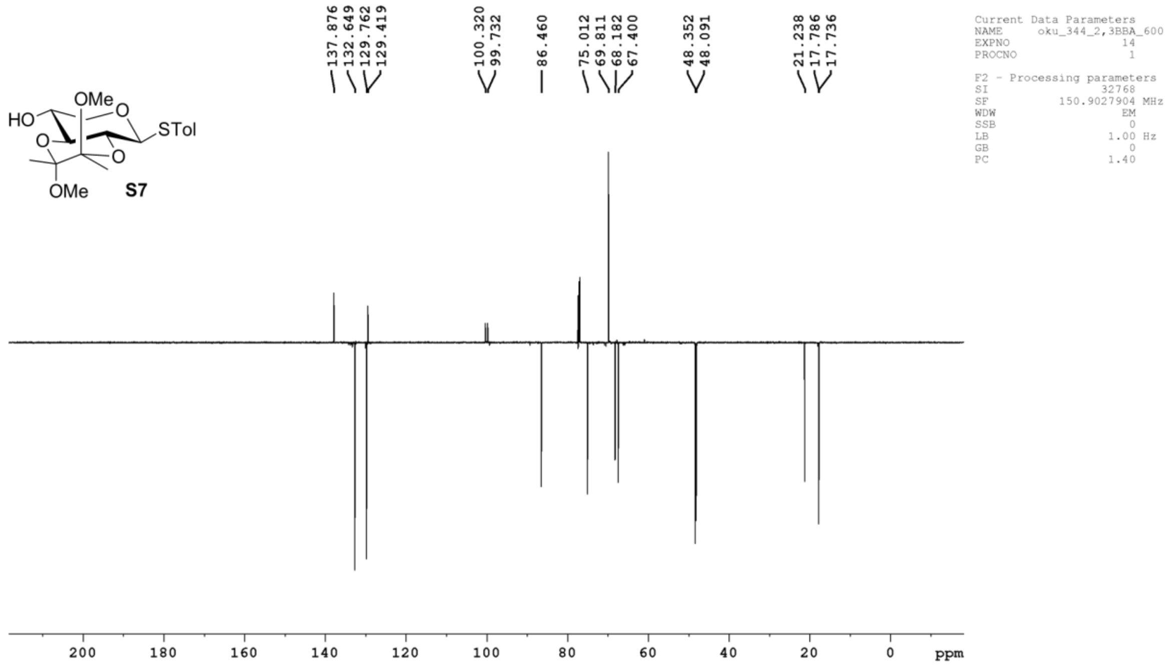

^{13}C NMR spectrum of compound **S7** (151 MHz, $CDCl_3$)

4-Methylphenyl 2,3-*O*-(2,3-dimethoxybutane-2,3-diyl)-4-*O*-benzyl-1-thio-β-D-xylopyranoside (S8)

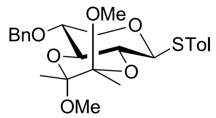

S8

Experimental procedure: Synthesis of compound **S8** was performed in two batches (small scale with 92 mg starting material **S7** and large scale with 4.00 g starting material **S7**) and purified together by silica gel column chromatography. To a solution of starting material **S7** (92 mg, 0.25 mmol and 4.00 g, 10.8 mmol) in DMF (2.48 mL and 100 mL), TBAI (18 mg, 0.05 mmol and 797 mg, 2.16 mmol), BnBr (35 µL, 0.3 mmol and 1.54 mL, 12.94 mmol), and NaH (20 mg, 0.5 mmol and 862 mg, 21.6 mmol) were added sequentially at 0 °C under Ar-atmosphere. Each reaction mixture was vigorously stirred at 0 °C. After 30 min, the reaction was quenched by dropwise addition of water (10 mL) using an ice bath, diluted with EtOAc (100 mL), and washed with saturated aqueous $NaHCO_3$ solution (100 mL) and H_2O (100 mL, two times). The EtOAc layer was collected, dried over anhydrous Na_2SO_4, filtered, and concentrated under reduced pressure to yield a viscous pale-yellow crude. The crude was subjected to purification by silica gel column chromatography (EtOAc/hexanes = 1/7, v/v) to afford product **S8** (5.00 g, quantitative yield) as a viscous syrup.

R

R_f: 0.7 (EtOAc/hexanes = 1/3, v/v).

^{1}H NMR (300 MHz, $CDCl_3$, major stereoisomer): δ 7.45-7.38 (m, 2H, Ar-H), 7.36-7.30 (m, 5H, Ar-H), 7.13-7.05 (m, 2H, Ar-H), 4.87 (d, J = 11.8 Hz, 1H, CH_2-Ph), 4.67 (d, J = 8.3 Hz, 1H, H-1), 4.63 (d, J = 10.3 Hz, 1H, CH_2-Ph), 4.01 (dd, J = 5.3, 11.5 Hz, 1H, H-5a), 3.86 (t, J = 9.4 Hz, 1H, H-3), 3.74-3.66 (m, 1H, H-4), 3.61 (t, J = 9.7 Hz, 1H, H-2), 3.32 (s, 3H, OCH_3), 3.26 (s, 3H, OCH_3), 3.24-3.21 (m, 1H, H-5b), 2.32 (s, 3H, Ph-CH_3), 1.36 (s, 3H, CH_3), 1.35 (s, 3H, CH_3) ppm.

^{13}C NMR (76 MHz, $CDCl_3$): δ 132.7, 129.8, 128.6, 127.94, 127.82, 86.4, 75.2, 74.5, 73.9, 68.81, 68.67, 48.44, 48.18, 21.3, 18.0, 17.9 ppm.

ESI-HRMS: m/z $[M + NH_4]^+$ calcd. for $C_{25}H_{36}NO_6S$: 478.2258; found 478.2224.

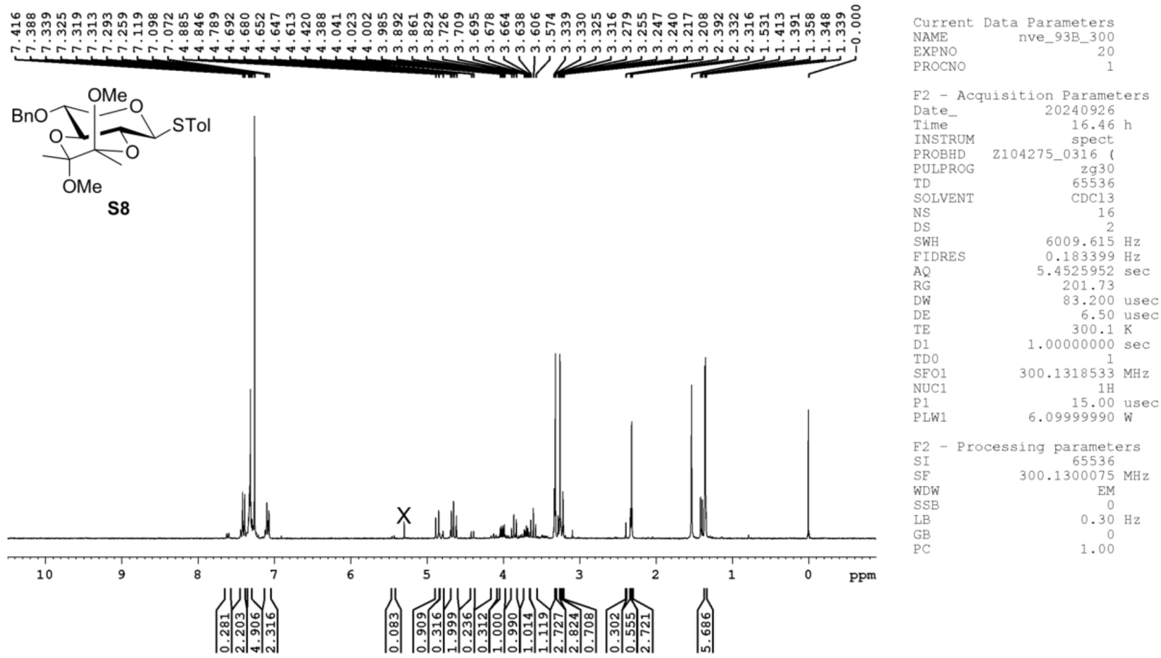

^{1}H NMR spectrum of compound **S8** (300 MHz, $CDCl_3$)

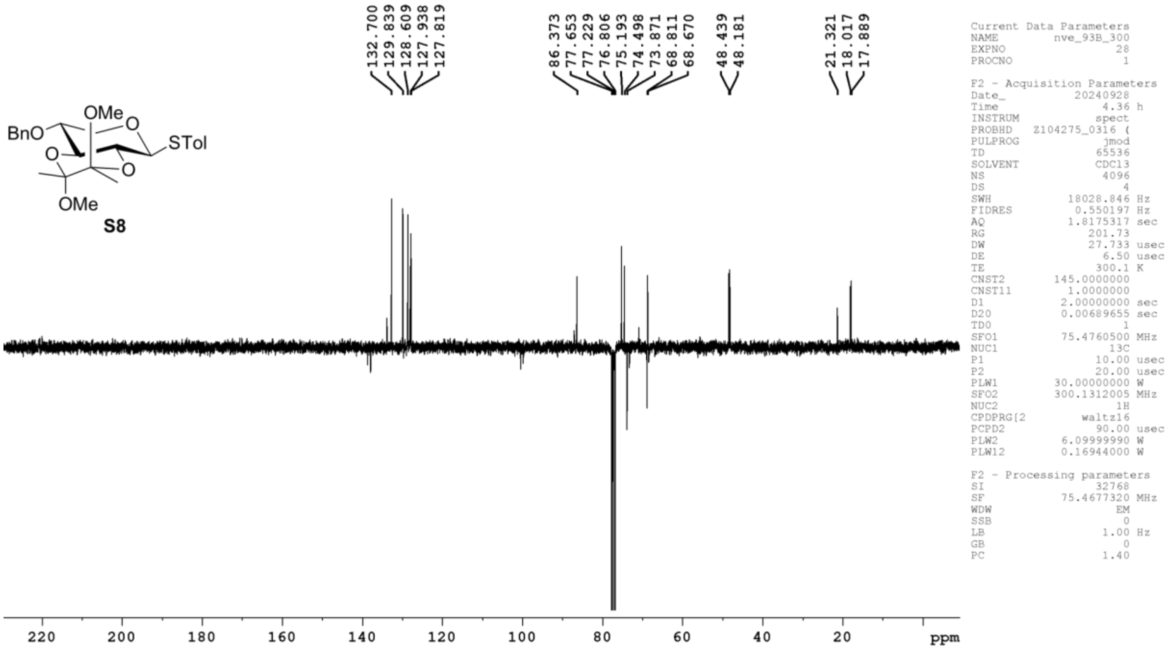

^{13}C APT NMR spectrum of compound **S8** (76 MHz, $CDCl_3$)

4-Methylphenyl 2-*O*-benzoyl-4-*O*-benzyl-1-thio-β-D-xylopyranoside (S6)

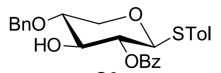

S6

Synthesis through Nap deprotection of compound S5 (Scheme S2):

Experimental procedure: To a solution of starting material **S5** (162 mg, 0.274 mmol) in a 10:1 v/v mixture of DCM (25 mL) and H_2O (2.5 mL), DDQ (186.4 mg, 0.821 mmol) was added at 0 °C under Ar-atmosphere. The reaction mixture was continuously stirred and gradually allowed to reach rt without removing the ice bath. After 1 h 15 min, the water bath was removed. After confirmation of reaction completion (3.5 h) by TLC, the reaction was quenched by slow addition of saturated aqueous $NaHCO_3$ solution (20 mL) at 0 °C. The DCM layer was collected, washed one more time with saturated aqueous $NaHCO_3$ solution (20 mL), dried over anhydrous Na_2SO_4, filtered, and concentrated under reduced pressure to yield a viscous yellow crude. The crude was subjected to purification by silica gel column chromatography (EtOAc/hexanes = 1/6 to 1/4, v/v) to afford product **S6** (101 mg, 82% yield) as a sticky foam.

Synthesis through regioselective benzoylation of compound S9 (Scheme S2)[3]:

Experimental procedure: To a solution of **S8** (4.22 g, 9.17 mmol) in DCM (92 mL) was added H_2O (1.65 mL) and TFA (100%, 16.5 mL) at rt. After 35 min, the reaction mixture was washed with saturated aqueous $NaHCO_3$-solution (80 mL, two times). The DCM layer was collected, dried over anhydrous Na_2SO_4, filtered, and concentrated under reduced pressure to yield a viscous pale-yellow crude **S9**, which was directly used for the next step without further purification. ***R_f*:** 0.13 (EtOAc/hexanes = 1/3, v/v). **^{1}H NMR (300 MHz, $CDCl_3$) of S9:** δ 7.45-7.39 (m, 2H, Ar-H), 7.38-7.28 (m, 5H, Ar-H), 7.15-7.09 (m, 2H, Ar-H), 4.69 (d, J = 11.8 Hz, 1H, CH_2-Ph), 4.63 (d, J = 11.8 Hz, 1H, CH_2-Ph), 4.47 (d, J = 9.0 Hz, 1H, H-1), 4.07 (dd, J = 4.8, 11.5 Hz, 1H, H-5a), 3.67 (t, J = 8.4 Hz, 1H, H-3), 3.52-3.42 (m, 1H, H-4), 3.35 (t, J = 8.7 Hz, 1H, H-2), 3.26 (dd, J = 9.8, 11.4 Hz, 1H, H-5b), 2.78-2.62 (br, 2H, O-H), 2.34 (s, 3H, Ph-CH_3) ppm. **^{13}C NMR (76 MHz, $CDCl_3$) of S9:** δ 138.7, 138.1, 133.6, 130.0, 128.8, 128.3, 128.0, 89.1, 77.0, 76.74, 73.21, 72.0, 67.3, 21.3 ppm. To a solution of crude starting material **S9** (9.17 mmol) in DCM (165.1 mL) was added TBAHS (623 mg, 1.84 mmol), BzCl (1.44 mL, 12.4 mmol) and 1M aqueous NaOH solution (24 mL, 24 mmol) at −5 °C using a cooling bath (ice + acetone). The reaction mixture was vigorously stirred. After 20 min, the reaction mixture was washed with H_2O (100 mL) and saturated aqueous $NaHCO_3$ solution (100 mL) using an ice bath. The DCM layer was collected, dried over anhydrous Na_2SO_4, filtered, and concentrated under reduced pressure to yield a viscous crude. The crude was subjected to purification by silica gel column chromatography (EtOAc/hexanes = 1/7 to 1/3, v/v) to afford product **S6** (2.47 g, 58% yield over 2 steps) as a sticky foam.

***R_f*:** 0.40 (EtOAc/hexanes = 1/3, v/v, run two times).

^{1}H NMR (300 MHz, $CDCl_3$): δ 8.12-8.05 (m, 2H, Ar-H), 7.64-7.55 (m, 1H, Ar-H), 7.50-7.42 (m, 2H, Ar-H), 7.38-7.28 (m, 7H, Ar-H), 7.12-7.04 (m, 2H, Ar-H), 5.0 (t, J = 9.2 Hz, 1H, H-2), 4.76-4.62 (m, 3H, H-1, CH_2-Ph), 4.12 (dd, J = 5.1, 11.5 Hz, 1H, H-5a), 3.87 (t, J = 8.7 Hz, 1H, H-3), 3.58 (ddd, J = 5.0, 8.7, 10.0 Hz, 1H, H-4), 3.30 (dd, J = 10.1, 11.4 Hz, 1H, H-5b), 2.7 (br, 1H, O-H), 2.32 (s, 3H, Ph-CH_3) ppm.

^{13}C NMR (76 MHz, $CDCl_3$): δ 166.3, 138.55, 138.10, 133.58, 133.52, 130.2, 129.9, 128.77, 128.68, 128.61, 128.24, 128.04, 87.0, 77.6, 76.2, 73.44, 73.18, 67.6, 21.3 ppm.

ESI-HRMS: m/z $[M + NH_4]^+$ calcd. for $C_{26}H_{30}NO_5S$: 468.1839; found 468.1841.

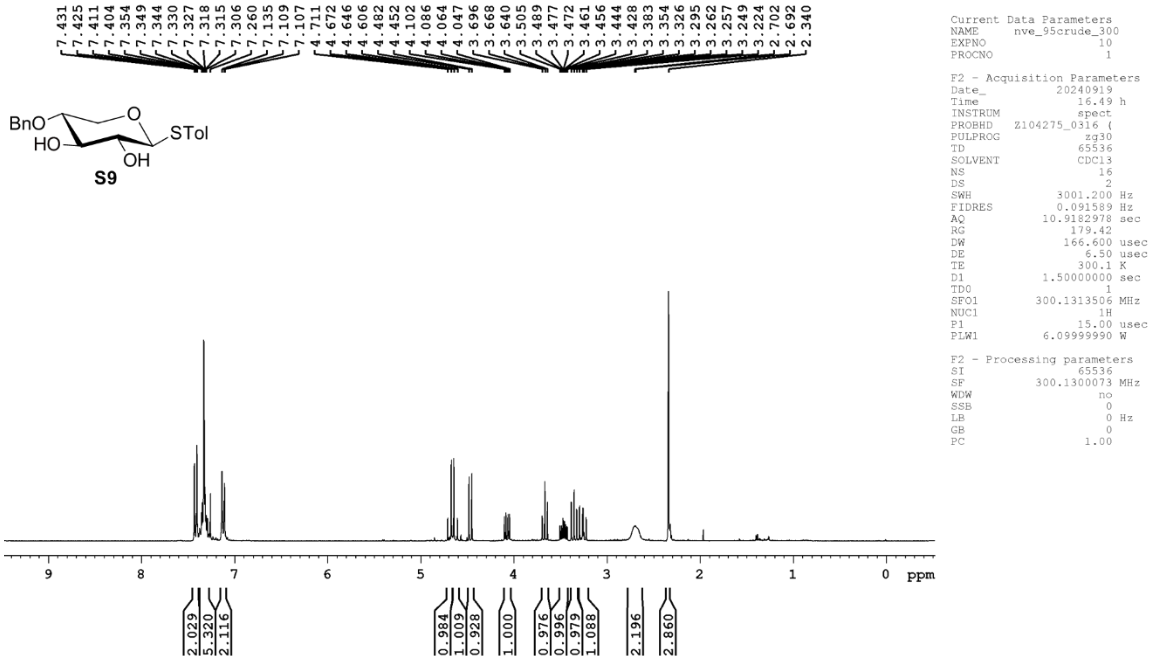

^{1}H NMR spectrum of compound **S9** (300 MHz, $CDCl_3$)

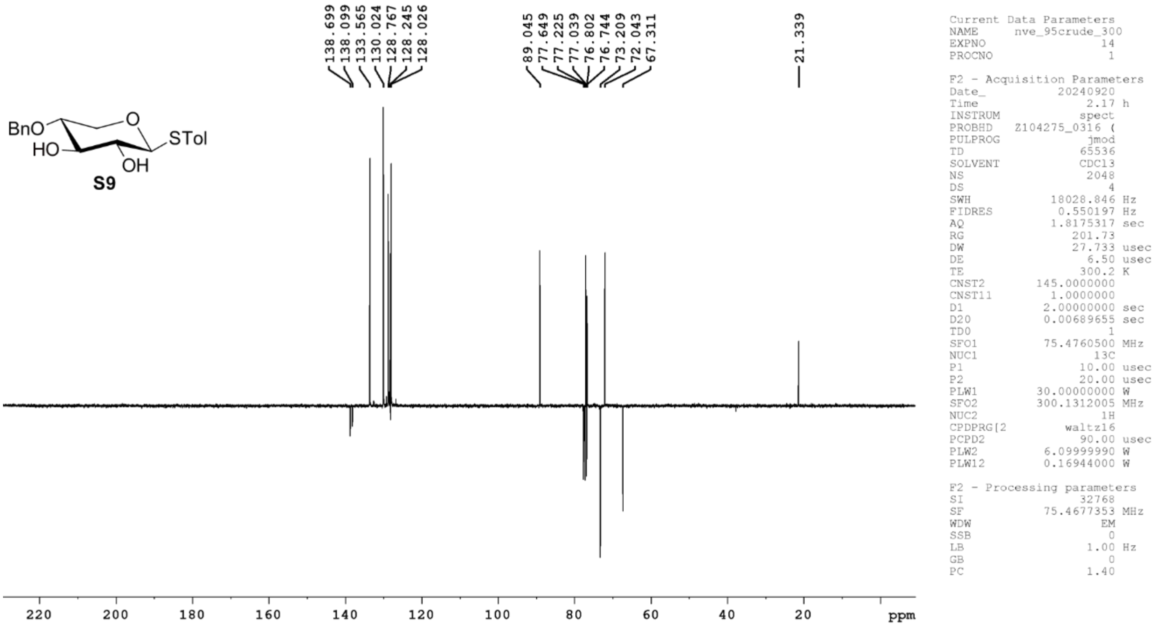

^{13}C APT NMR spectrum of compound **S9** (76 MHz, $CDCl_3$)

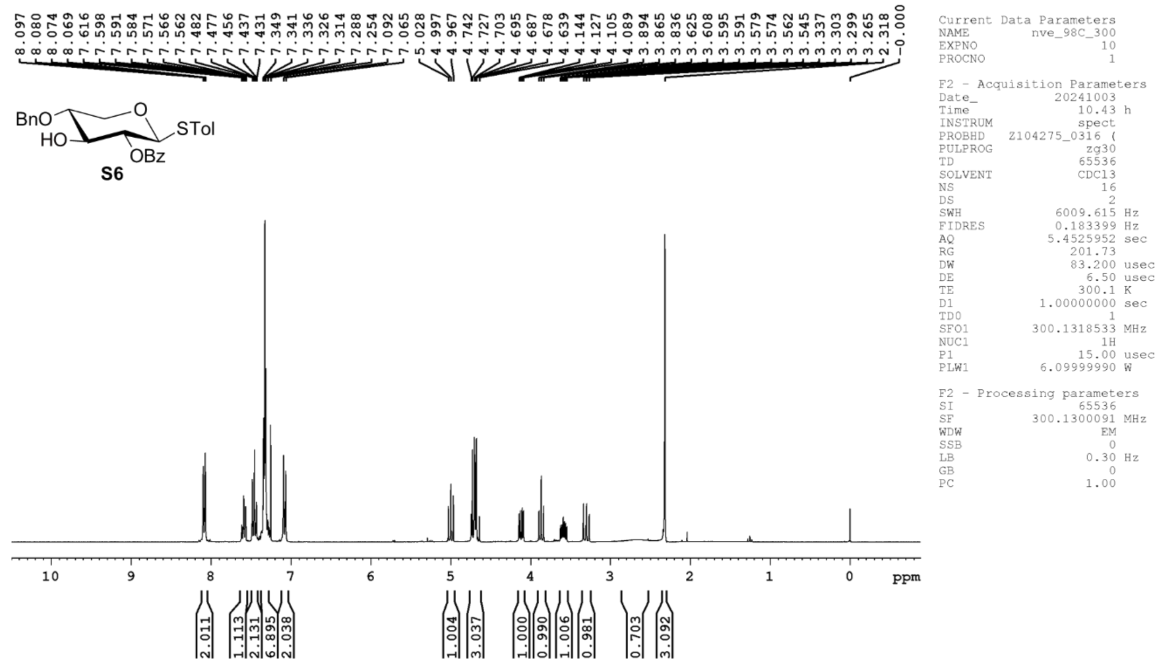

^{1}H NMR spectrum of compound **S6** (300 MHz, $CDCl_3$)

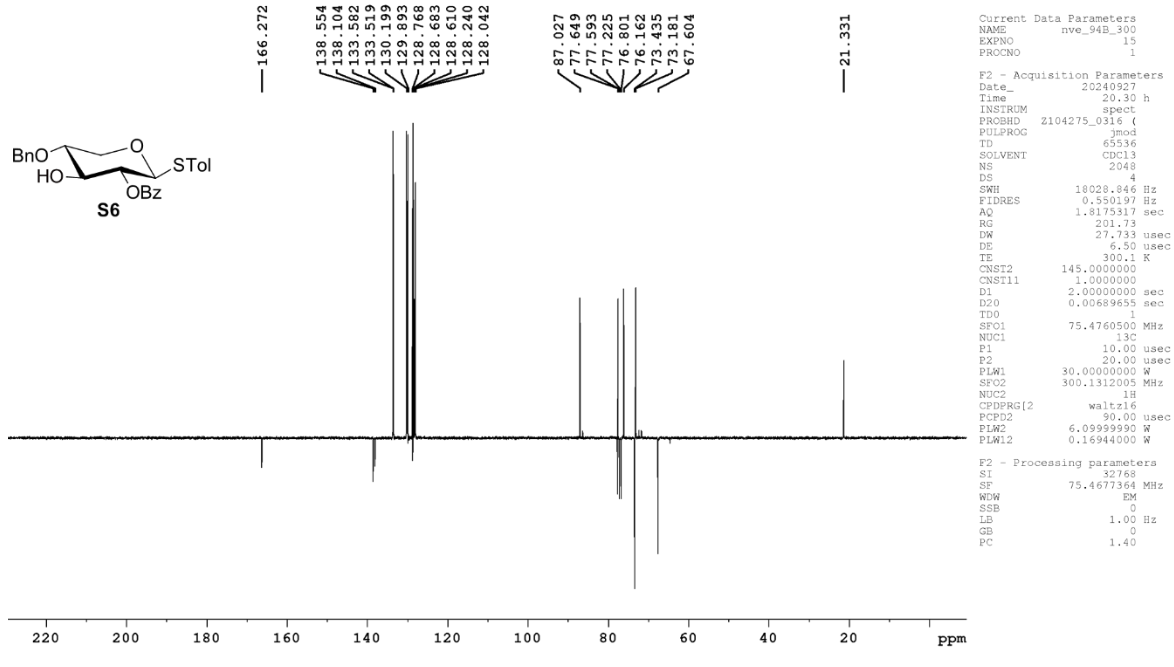

^{13}C APT NMR spectrum of compound **S6** (76 MHz, $CDCl_3$)

4-Methylphenyl 2-*O*-benzoyl-3-*O*-fluorenylcarboxymethyl-4-*O*-benzyl-1-thio-β-D-xylopyranoside (S10)

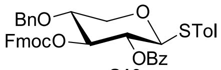

S10

Experimental procedure: Synthesis of compound **S10** was performed in two batches (small scale with 100.0 mg starting material **S6** and large scale with 2.12 g starting material **S6**), and purified together by silica gel column chromatography. To a solution of starting material **S6** (100 mg, 0.22 mmol and 2.12 g, 4.70 mmol) in 2:1 v/v mixture of DCM (1 mL and 21.4 mL) and pyridine (0.5 mL and 10.7 mL), FmocCl (85.4 mg, 0.33 mmol and 1.83 g, 7.056 mmol) was added at rt under Ar-atmosphere. After confirmation of reaction completion (1.5 h) by TLC, the reaction mixture was diluted with DCM (20 mL). Then, the reaction mixture was sequentially washed with 2M-aqueous HCl solution (25 mL), saturated aqueous $NaHCO_3$ solution (25 mL), 2M-aqueous HCl solution (25 mL), and saturated aqueous $NaHCO_3$ solution (25 mL). The DCM layer was collected, dried over anhydrous Na_2SO_4, filtered, and concentrated under reduced pressure to yield a viscous crude. The crude was subjected to purification by silica gel column chromatography (EtOAc/hexanes = 1/7 to 1/3, v/v) to afford product **S10** (3.01 g, 91% yield) as a white amorphous solid.

***R$_f$*:** 0.49 (EtOAc/hexanes = 1/3, v/v).

^{1}H NMR (600 MHz, $CDCl_3$): δ 8.04-7.99 (m, 2H, Ar-H), 7.73-7.68 (m, 2H, Ar-H), 7.53-7.45 (m, 3H, Ar-H), 7.38-7.31 (m, 6H, Ar-H), 7.29-7.23 (m, 5H, Ar-H), 7.23-7.17 (m, 2H, Ar-H), 7.10-7.06 (m, 2H, Ar-H), 5.26-5.17 (m, 2H, H-2, H-3), 4.80 (d, J = 8.9 Hz, 1H, H-1), 4.65 (d, J = 12.0 Hz, 1H, CH_2-Ph), 4.58 (d, J = 11.9 Hz, 1H, CH_2-Ph), 4.28 (dd, J = 7.2, 10.5 Hz, 1H, CH_2-Fmoc), 4.19 (dd, J = 7.9, 10.5 Hz, 1H, CH_2-Fmoc), 4.15 (dd, J = 5.2, 11.8 Hz, 1H, H-5a), 4.03 (t, J = 7.5 Hz, 1H, CH-Fmoc), 3.77 (ddd, J = 5.2, 8.6, 9.5 Hz, 1H, H-4), 3.41 (dd, J = 9.8, 11.8 Hz, 1H, H-5b), 2.31 (s, 3H, Ph-CH_3) ppm.

^{13}C NMR (151 MHz, $CDCl_3$): δ 165.4, 154.8, 143.58, 143.22, 141.39, 141.31, 138.6, 137.8, 133.55, 133.49, 130.2, 129.93, 129.53, 128.71, 128.67, 128.55, 128.18, 127.97, 127.90, 127.32, 125.42, 125.21, 120.1, 87.3, 79.3, 75.0, 73.4, 70.87, 70.43, 67.6, 46.7, 21.3 ppm.

ESI-HRMS: m/z $[M + K]^+$ calcd. for $C_{41}H_{36}O_7SK$: 711.1813; found 711.1817.

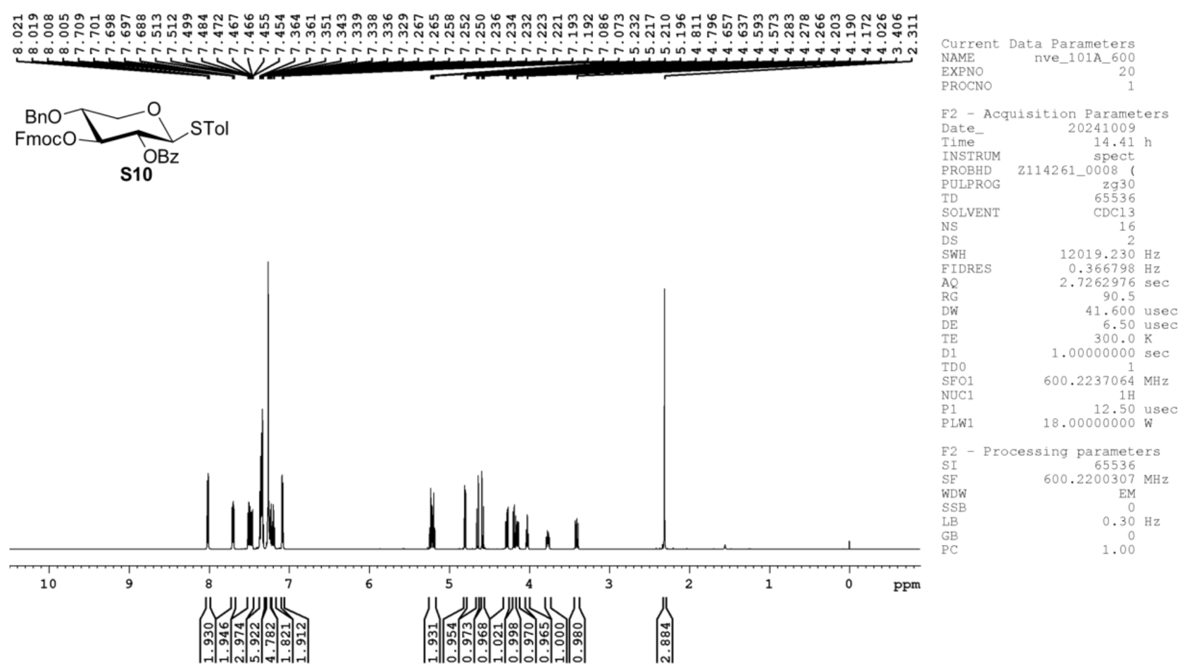

^{1}H NMR spectrum of compound **S10** (600 MHz, $CDCl_3$)

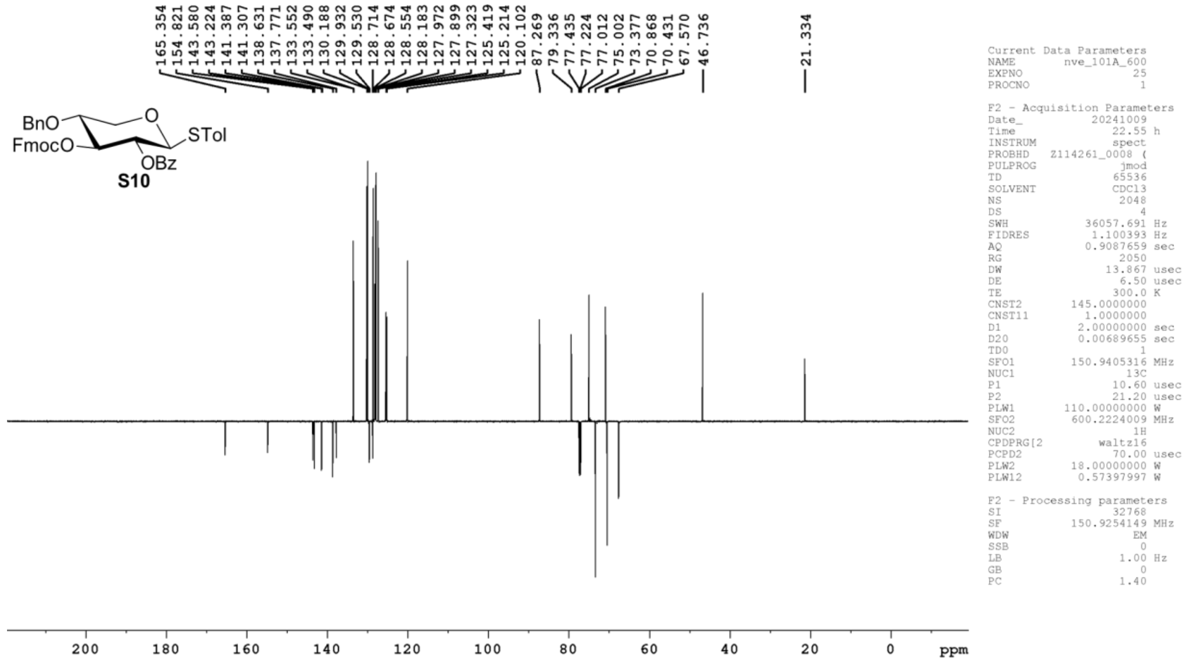

^{13}C APT NMR spectrum of compound **S10** (151 MHz, $CDCl_3$)

Dibutoxyphosphoryloxy 2-*O*-benzoyl-3-*O*-fluorenylcarboxymethyl-4-*O*-benzyl-β-D-xylopyranoside (6)

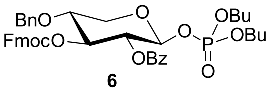

6

Experimental procedure: A solution of thioxyloside **S10** (1.00 g, 1.49 mmol) and dibutyl hydrogen phosphate (750 µL, 3.77 mmol) in anhydrous DCM (7.5 mL) was stirred with 4 Å pulverized molecular sieves (1.22 g) at rt for 40 min under Ar-atmosphere. After cooling to 0 °C, NIS (recrystallized, 401 mg, 1.78 mmol) and TfOH (40 µL, 0.446 mmol) were added to the reaction mixture. After 35 min, the reaction mixture was quenched by dropwise addition of Et_3N (100 µL) to neutralize, diluted with DCM (10 mL), and filtered. The filtrate was washed with saturated aqueous $NaHCO_3$ solution (15 mL) and 25% (w/v) aqueous $Na_2S_2O_3$ solution (15 mL), dried over Na_2SO_4, filtered, and concentrated under reduced pressure to afford a yellow viscous residue. The residue was subjected to purification by silica gel column chromatography (EtOAc/hexanes = 1/4 to 1/2, v/v) to afford product **6** (944 mg, 84% yield) as a viscous syrup.

***R_f*:** 0.36 (EtOAc/hexanes = 1/2, v/v).

^{1}H NMR (600 MHz, $CDCl_3$, β-anomer): δ 8.03-7.99 (m, 2H, Ar-H), 7.74-7.69 (m, 2H, Ar-H), 7.54-7.46 (m, 3H, Ar-H), 7.38-7.33 (m, 4H, Ar-H), 7.31-7.27 (m, 5H, Ar-H), 7.25-7.18 (m, 2H, Ar-H), 5.42 (t, *J* = 7.1 Hz, 1H, H-1), 5.33 (dd, *J* = 7.1, 9.0 Hz, 1H, H-2), 5.20 (t, *J* = 8.7 Hz, 1H, H-3), 4.67 (d, *J* = 12.0 Hz, 1H, CH_2-Ph), 4.61 (d, *J* = 11.9 Hz, 1H, CH_2-Ph), 4.32 (dd, *J* = 7.2, 10.5 Hz, 1H, CH_2-Fmoc), 4.25 (dd, *J* = 7.7, 10.5 Hz, 1H, CH_2-Fmoc), 4.11 (dd, *J* = 5.0, 12.0 Hz, 1H, H-5a), 4.08-3.99 (m, 3H, CH-Fmoc, OBu), 3.86-3.78 (m, 2H, H-4, OBu), 3.77-3.71 (m, 1H, OBu), 3.55 (dd, *J* = 9.3, 12.1 Hz, 1H, H-5b), 1.65-1.59 (m, 2H, Bu), 1.40-1.31 (m, 4H, Bu), 1.13-1.05 (m, 2H, Bu), 0.90 (t, *J* = 7.41 Hz, 3H, CH_3), 0.71 (t, *J* = 7.38 Hz, 3H, CH_3) ppm.

^{13}C NMR (151 MHz, $CDCl_3$): δ 165.2, 154.7, 143.54, 143.21, 141.43, 141.36, 137.6, 133.7, 130.2, 129.1, 128.74, 128.63, 128.29, 128.02, 127.99, 127.35, 125.38, 125.21, 120.2, 97.0 (d, *J* = 4.86 Hz, C-1), 77.4, 74.5, 73.4, 71.56, 71.49, 70.5, 68.23, 68.19, 68.12, 68.08, 64.3, 46.8, 32.28, 32.23, 32.07, 32.02, 18.75, 18.50, 13.74, 13.58 ppm.

ESI-HRMS: m/z $[M + Na]^+$ calcd. for $C_{42}H_{47}O_{11}PNa$: 781.2748; found 781.2746.

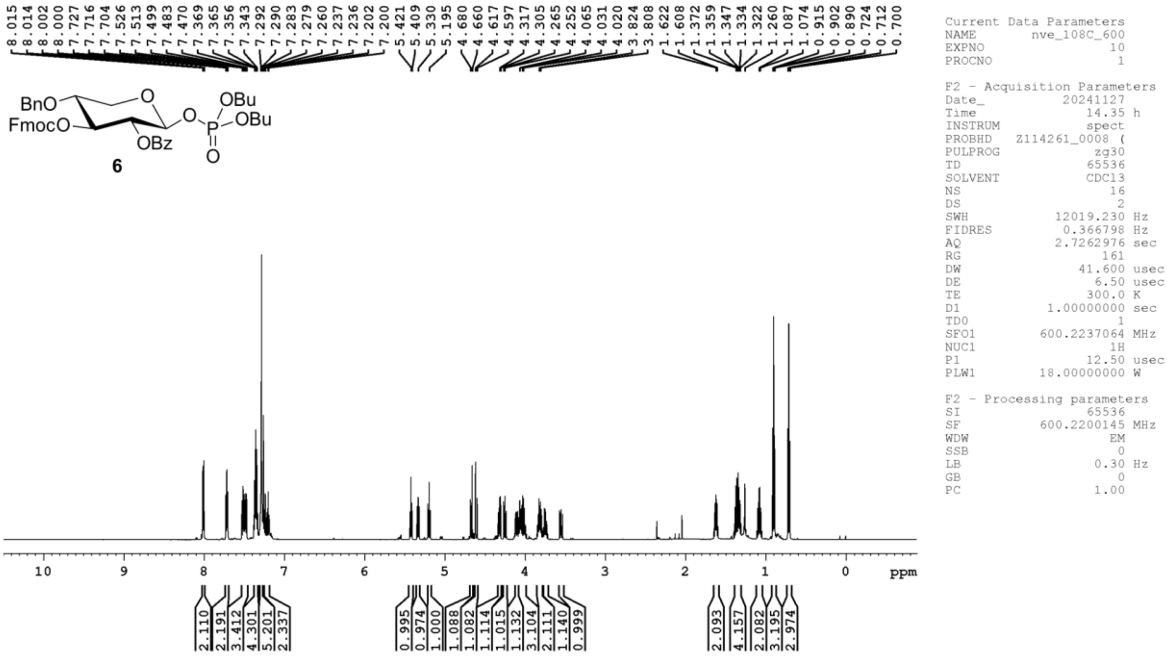

^{1}H NMR spectrum of compound **6** (600 MHz, $CDCl_3$)

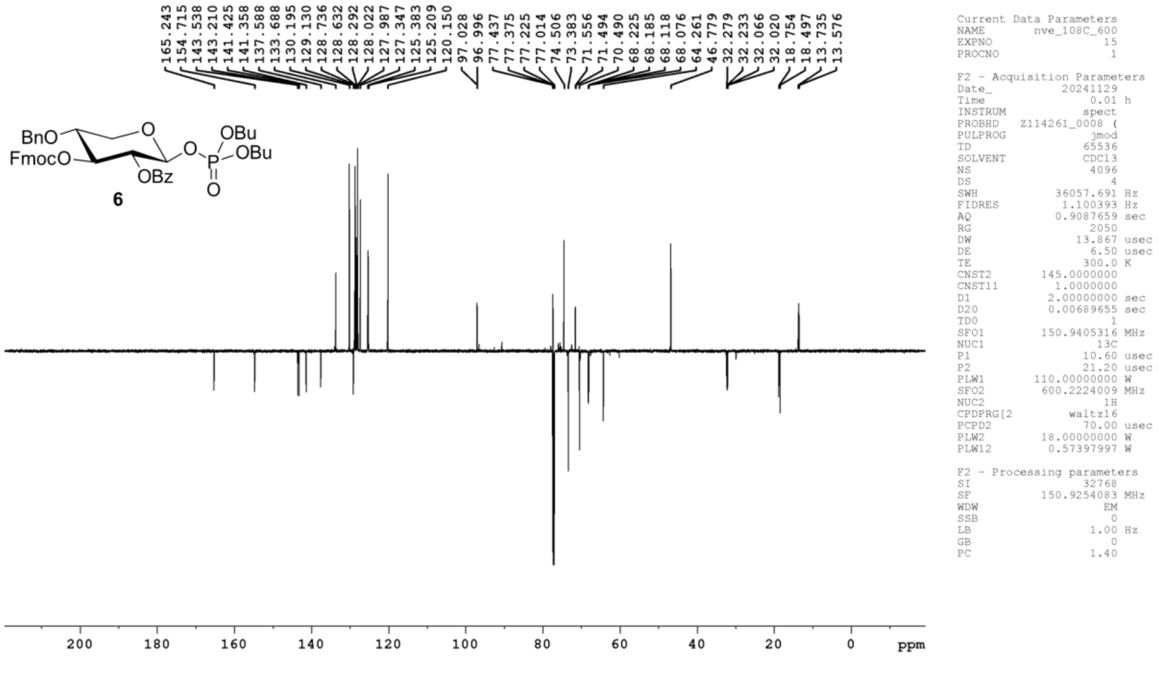

^{13}C APT NMR spectrum of compound **6** (151 MHz, $CDCl_3$)

4. Automated Glycan Assembly (AGA)

4.1 Optimization of AGA reactions for synthesis of β3-xylan di- and tetrasaccharides using disarmed donors.

Table S4. AGA of β3-xylan oligosaccharides using disarmed donors **2** and **3**.

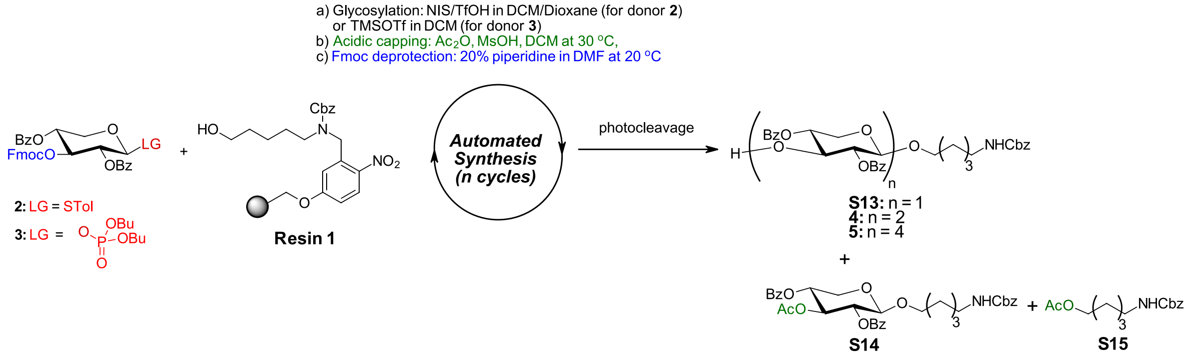

Entry	Reaction conditions	Loop (*n* cycles)	β3-Xylan (yield)[a]
	reactions with donor **2**		
1	–20 °C (5 min) to 0 °C (20 min)	2 ×	**4** (14%[b])
2	–20 °C (25 min) to 0 °C (20 min)	2 ×	**4** (38%, 5 steps)
3	–20 °C (25 min) to 0 °C (20 min), no capping	2 ×	**4** (27%) + **S13** (36%)
4	–20 °C (25 min) to 0 °C (20 min), no capping, 2 cycles/glycosylation	2 ×	**4** (37%[b])
5	–30 °C (25 min) to –10 °C (20 min), no capping	2 ×	**4** (5%) + **S13** (53%)
6	–20 °C (25 min) to 0 °C (20 min)	4 ×	**5** (9%, 9 steps)
7	–30 °C (25 min) to –10 °C (20 min)	4 ×	**5** (7%)
8	–20 °C (25 min) to 0 °C (40 min)	4 ×	complex mixture
	reactions with donor **3**		
9	–25 °C (5 min) to 0 °C (30 min)	2 ×	**4** (11%) + **S14** (22%)
10[c]	–35 °C (5 min) to –12.5 °C (30 min)	2 ×	**4** (17%) + **S14** (17%)
11	–35 °C (5 min) to –23 °C (30 min)	2 ×	**4** (22%) + **S14** (18%)
12	–35 °C (5 min) to –15 °C (30 min), no capping	2 ×	**4** (8%) + **S13** (33%)
13	–35 °C (5 min) to –15 °C (30 min), 2 cycles/glycosylation	2 ×	**4** (24%) + **S14** (12%) + **S15** (26%)

[a]Isolated yield. [b]Yield was determined using a HPLC-based calibration curve. [c]50% UV power was used for the photocleavage reaction.

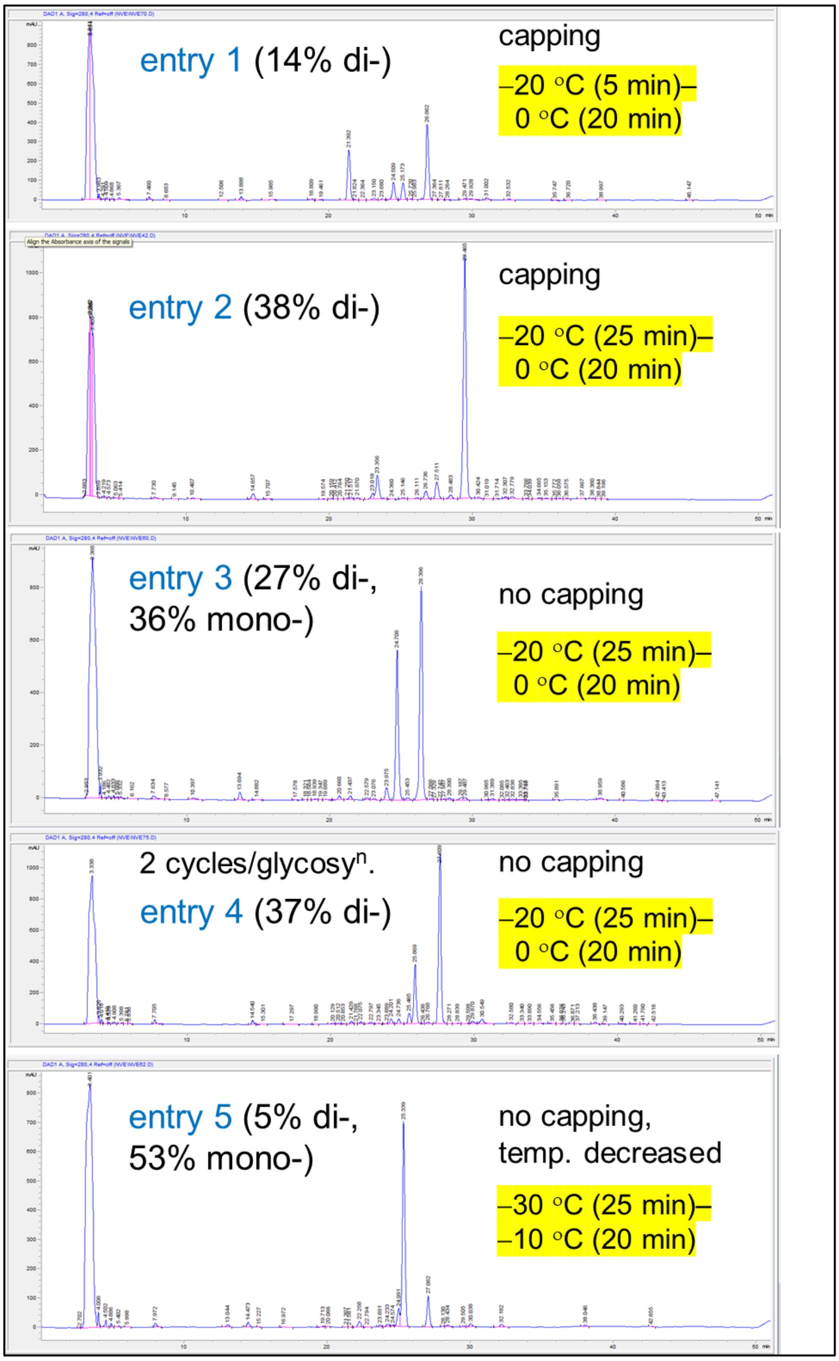

Figure S1. HPLC-Chromatograms of crude reaction mixtures obtained after AGA of disaccharide **4** (in entries 1–5 of Table S4) using donor **2**. YMC-Small NP column was used with a gradient of EtOAc in hexanes (50 min, flow rate 1 mL/min).

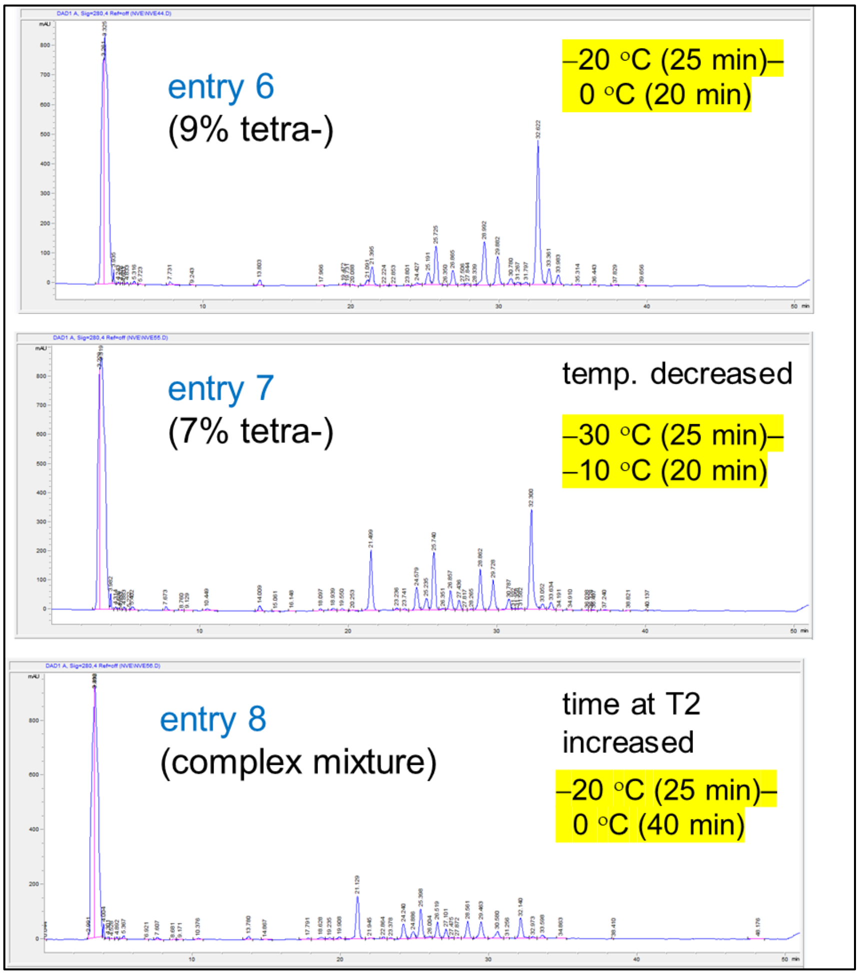

Figure S2. HPLC-Chromatograms of crude reaction mixtures obtained after AGA of tetrasaccharide **5** (in entries 6–8 of Table S4) using donor **2**. YMC-Small NP column was used with a gradient of EtOAc in hexanes (50 min, flow rate 1 mL/min).

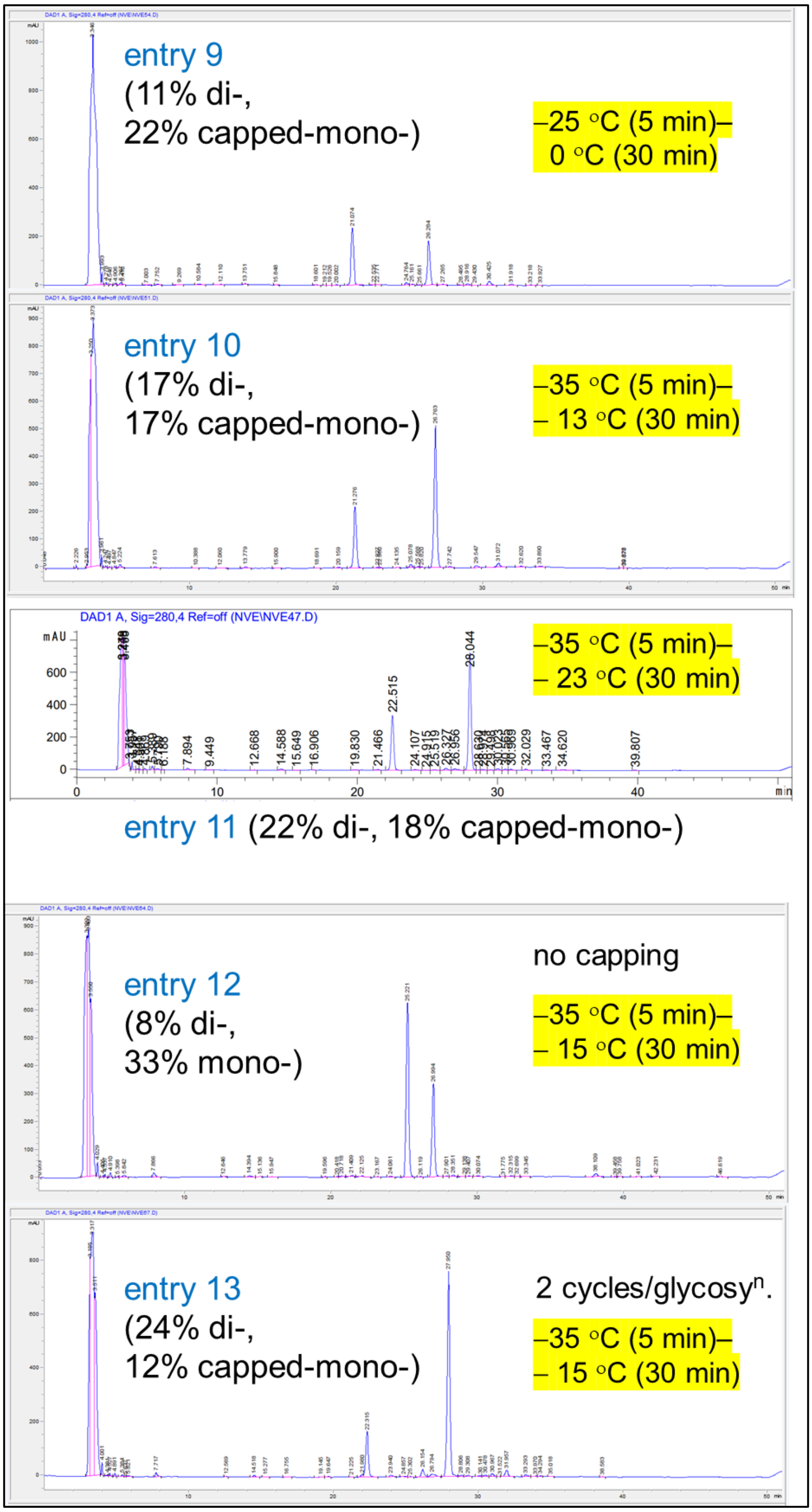

Figure S3. HPLC-Chromatograms of crude reaction mixtures obtained after AGA of disaccharide **4** (in entries 9–13 of Table S4) using donor **3**. YMC-Small NP column was used with a gradient of EtOAc in hexanes (50 min, flow rate 1 mL/min).

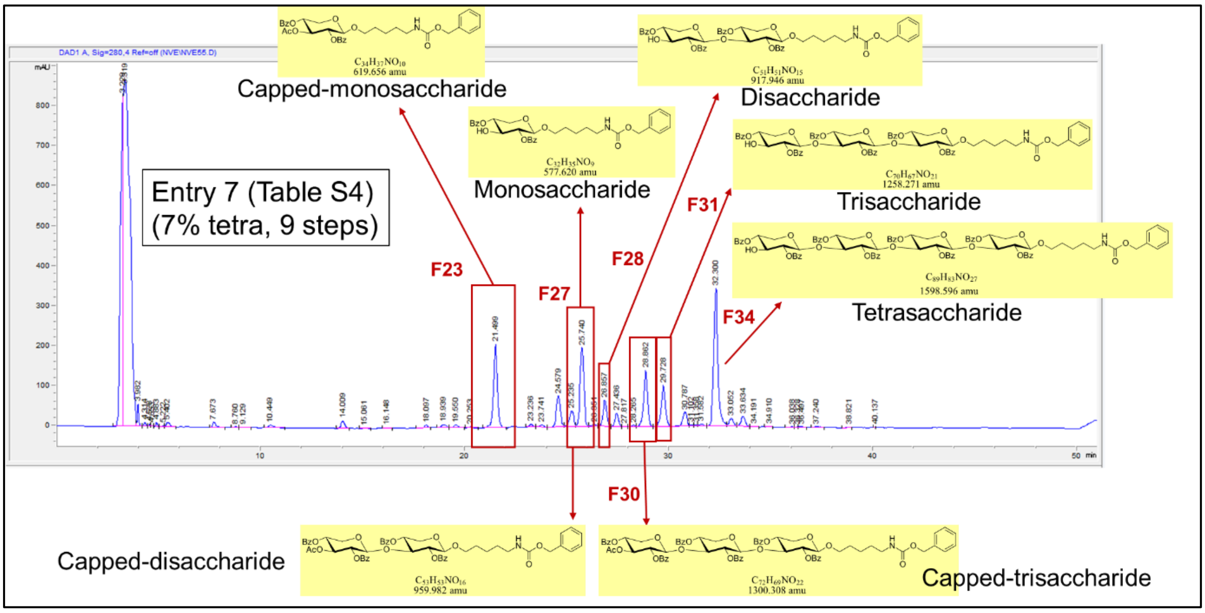

Figure S4. HPLC-Chromatogram of the crude reaction mixture after AGA of tetrasaccharide **5** using donor **2** (entry 7, Table S4). The structures behind the individual peaks were identified using LC/MS-analysis.

Calibration curve for yield determination of disaccharide **4** (entries 1 and 4, Table S4)

The calibration curve for disaccharide **4** was plotted from data obtained from the absorption peak area generated by injecting the same volume of four solutions (1–4, with different concentrations) of pure disaccharide **4** in toluene into the analytical HPLC-system (**Figures S5** and **S6**).

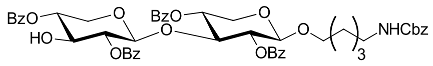

4

	µmoles injected	peak area (λ = 280 nm)
1	0.0267	3806
2	0.0533	7560
3	0.1067	14802
4	0.2135	28846

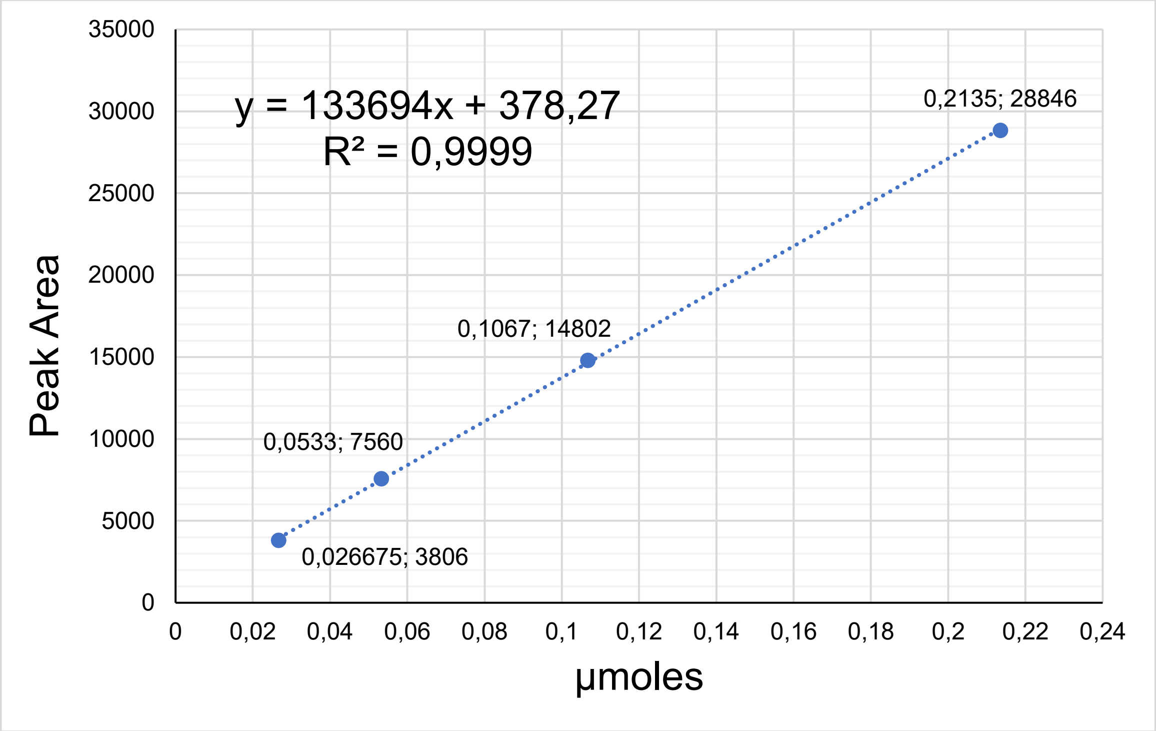

Figure S5. Plot of disaccharide **4** absorption peak area (at λ = 280 nm) *vs* µmoles of **4** injected into the analytical HPLC-system.

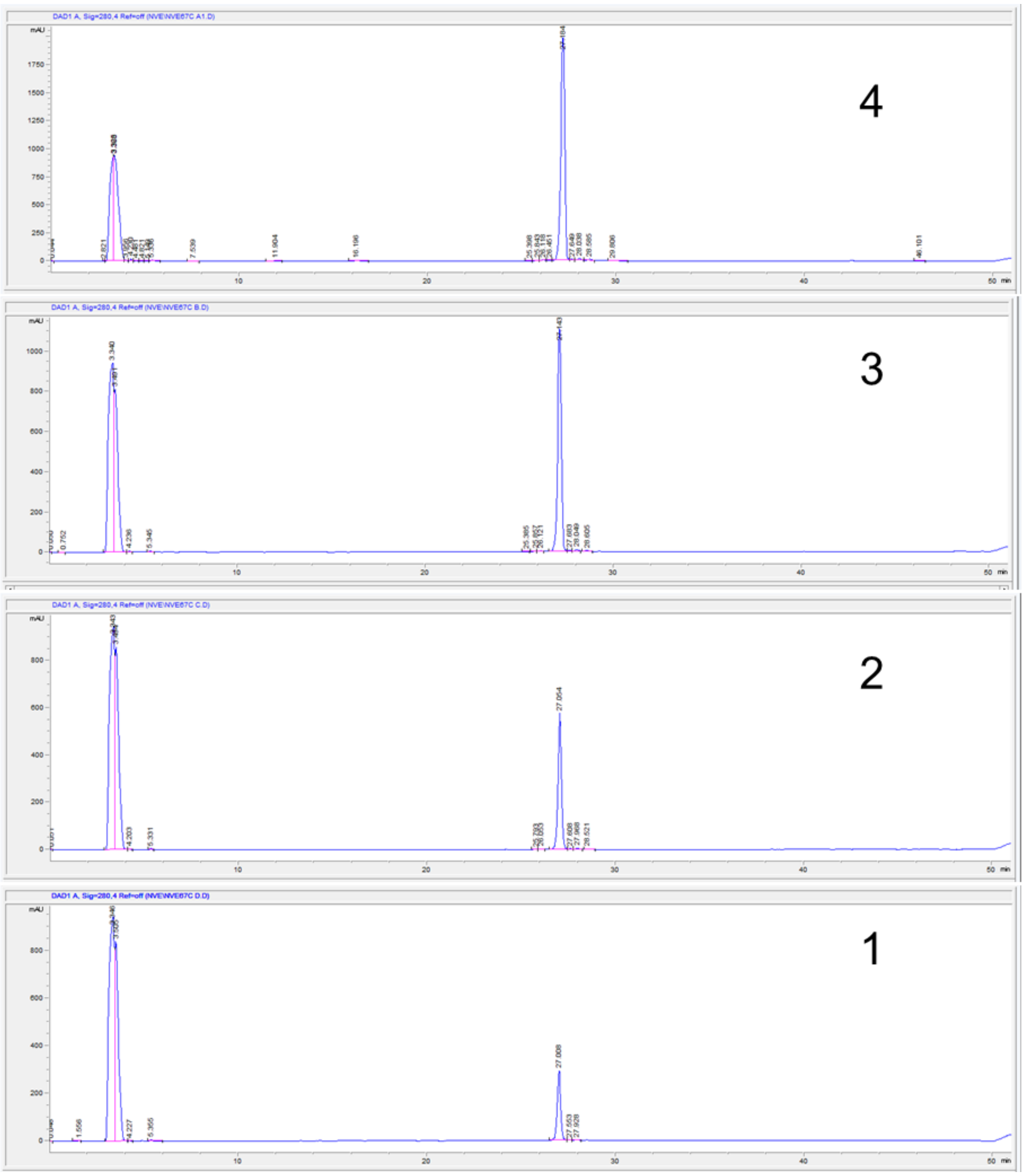

Figure S6. HPLC-Chromatograms of four different concentrations of disaccharide **4** using a YMC-Small NP column with a gradient of EtOAc in hexanes (50 min, flow rate 1 mL/min).

AGA of Bz-protected β3-xylan disaccharide 4 (entry 2, Table S4)

Benzyloxycarbonylaminopentyl 2,4-di-*O*-benzoyl-β-D-xylopyranosyl-(1→3)-2,4-di-*O*-benzoyl-β-D-xylopyranoside (**4**)

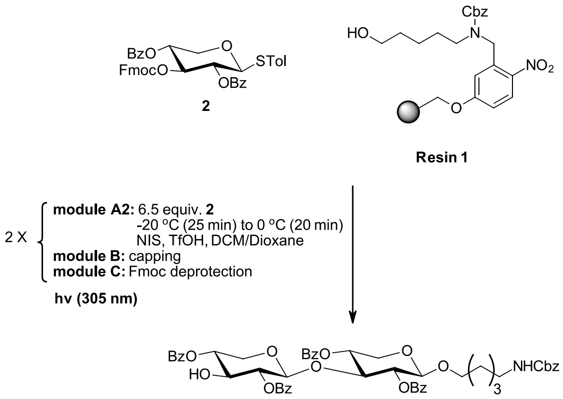

Experimental procedure: Linker-functionalized resin **1** (40 mg, 13.2 μmol) was placed in the synthesizer and synthesizer modules were applied as follows: 2 × [module A2 (BB **2**; total amount used = 118 mg, 0.17 mmol, 13 equiv.) at –20 °C (25 min) to 0 °C (20 min), module B, and module C]. Cleavage from the resin using UV irradiation at 305 nm in a continuous flow photoreactor afforded the crude product. Purification of the crude by normal phase HPLC using a preparative YMC-Small column (EtOAc/hexanes = 1/9 to 1/1.5, v/v) gave protected β3-xylan disaccharide **4** (4.6 mg, 38% yield over 5 steps) as a glassy solid.

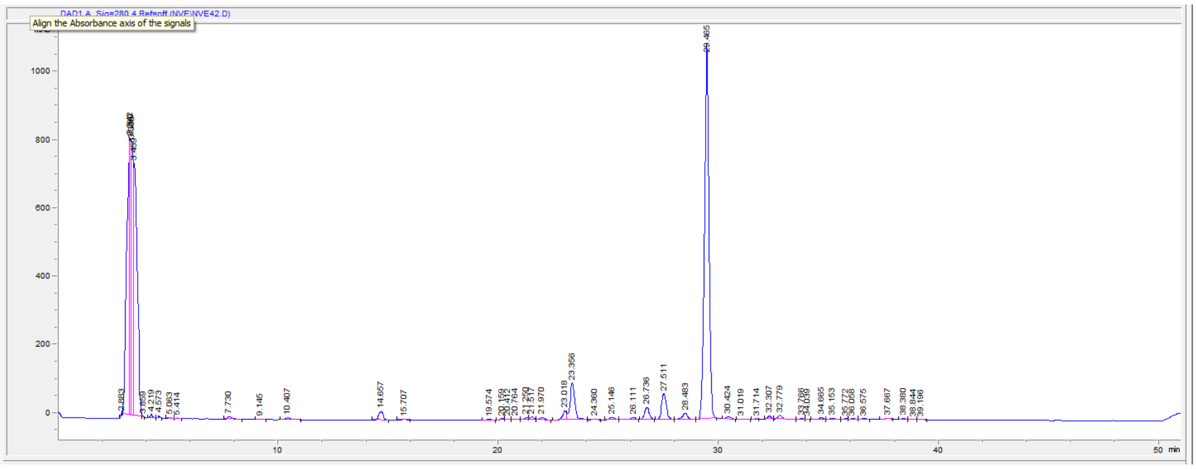

Analytical HPLC of the crude reaction mixture was performed on a YMC-Small NP column using a gradient of EtOAc in hexanes (50 min, flow rate 1 mL/min).

^{1}H NMR (300 MHz, $(CD_3)_2CO$): δ 8.16-8.09 (m, 2H, Ar-H), 8.01-7.90 (m, 4H, Ar-H), 7.81-7.74 (m, 2H, Ar-H), 7.68-7.40 (m, 10H, Ar-H), 7.39-7.26 (m, 7H, Ar-H), 6.14 (br, 1H, N-H), 5.25-5.12 (m, 2H, H-4A, H-2A), 5.12-5.0 (m, 4H, CH_2-Cbz, H-1B, H-2B), 4.99-4.90 (m, 1H, H-4B), 4.82 (d, J = 6.0 Hz, 1H, O-H), 4.75 (d, J = 6.0 Hz, 1H, H-1A), 4.46 (t, J = 7.5 Hz, 1H, H-3A), 4.26 (dd, J = 4.7, 12.0 Hz, 1H, H-5A), 4.14 (dd, J = 4.6, 11.9 Hz, 1H, H-5B), 4.09-4.0 (m, 1H, H-3B), 3.74-3.58 (m, 2H, OCH_2, H-5A), 3.51 (dd, J = 7.8, 11.9 Hz, 1H, H-5B), 3.45-3.32 (m, 1H, OCH_2), 2.96 (q, J = 6.6 Hz, 2H, CH_2-NHCbz), 1.44-1.30 (m, 4H, CH_2), 1.25-1.13 (m, 2H, CH_2) ppm.

^{13}C NMR (151 MHz, $(CD_3)_2CO$): δ 166.18, 166.12, 165.57, 165.40, 134.17, 134.11, 134.01, 133.7, 130.98, 130.85, 130.77, 130.57, 130.42, 129.40, 129.34, 129.20, 129.05, 128.65, 128.56, 101.85, 101.21, 77.5, 74.1, 73.1, 72.8, 71.31, 71.05, 69.5, 66.3, 62.27, 62.02, 41.4, 30.4, 23.8 ppm.

ESI-HRMS: m/z $[M + K]^+$ calcd. for $C_{51}H_{51}NO_{15}K$: 956.2890; found 956.2905.

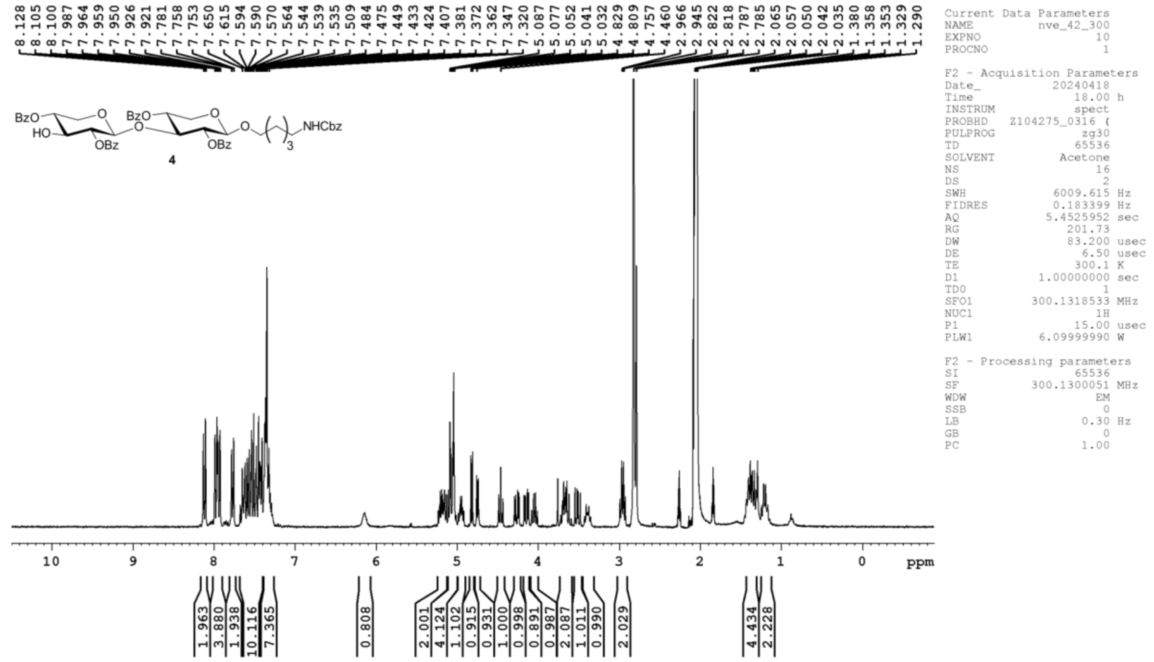

^{1}H NMR spectrum of compound **4** (300 MHz, $(CD_3)_2CO$)

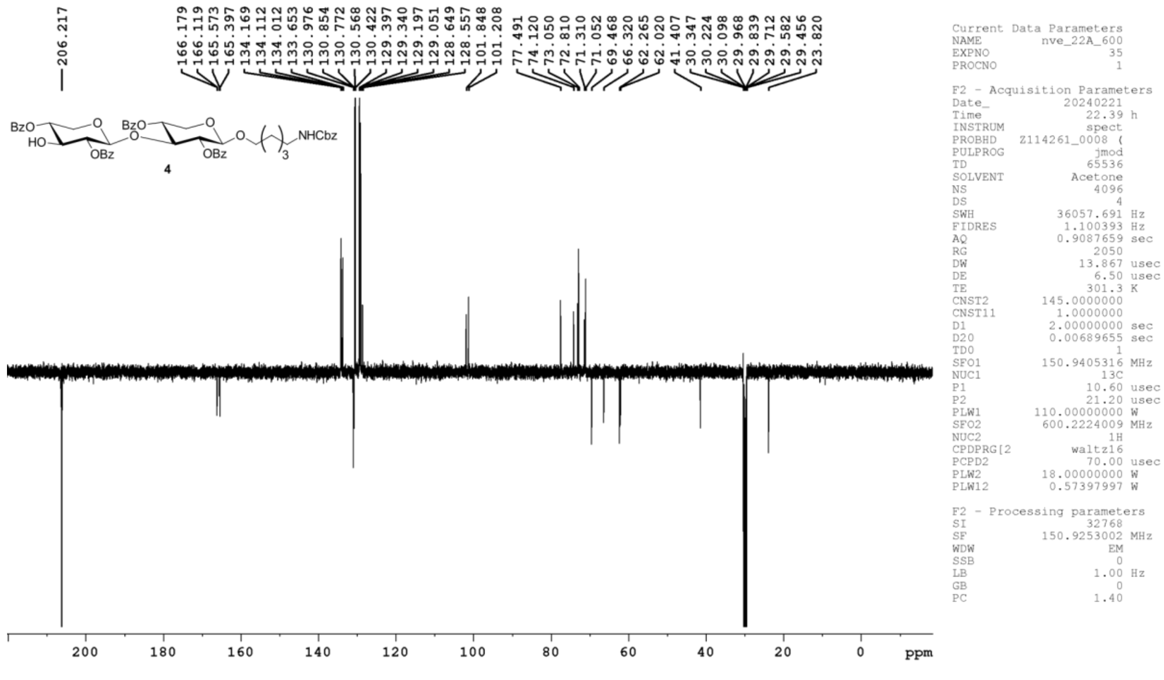

^{13}C APT NMR spectrum of compound **4** (151 MHz, $(CD_3)_2CO$)

Analytical data for linker-attached uncapped monosaccharide (S13), capped monosaccharide (S14), and capped linker (S15) as obtained as side products during various AGA reactions performed in Table S4

Benzyloxycarbonylaminopentyl 2,4-di-*O*-benzoyl-β-D-xylopyranoside (**S13**)

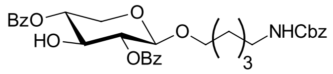

S13

The linker-attached Bz-protected monosaccharide **S13** was obtained as a side product during AGA reaction trials [entry 3 (2.6 mg, 36%), entry 5 (3.8 mg, 53%), and entry 12 (2.4 mg, 33%) of Table S4].

Physical description: Glassy solid.

^{1}H NMR (600 MHz, $CDCl_3$): δ 8.04-7.98 (m, 4H, Ar-H), 7.57-7.51 (m, 2H, Ar-H), 7.37-7.30 (m, 9H, Ar-H), 5.13-5.06 (m, 4H, CH_2-Cbz, H-2, H-4), 4.88 (d, J = 3.4 Hz, 1H, H-1), 4.74 (br, 1H, N-H), 4.31 (dd, J = 3.2, 12.8 Hz, 1H, H-5a), 4.17 (t, J = 4.7 Hz, 1H, H-3), 3.90-3.82 (m, 1H, OCH_2), 3.76 (dd, J = 4.4, 12.8 Hz, 1H, H-5b), 3.57-3.47 (m, 1H, OCH_2), 3.27 (br, 1H, O-H), 3.19-3.10 (m, 2H, $C\mathit{H}_2$-NHCbz), 1.69-1.61 (m, 2H, CH_2), 1.52-1.48 (m, 2H, CH_2), 1.43-1.33 (m, 2H, CH_2) ppm.

^{13}C NMR (151 MHz, $CDCl_3$): δ 166.24, 166.02, 133.57, 133.46, 130.17, 130.13, 129.95, 129.70, 128.74, 128.59, 128.58, 128.30, 99.1, 71.1, 69.2, 68.8, 66.9, 59.7, 41.1, 29.87, 29.15, 23.5 ppm.

ESI-HRMS: m/z $[M + Na]^+$ calcd. for $C_{32}H_{35}NO_9Na$: 600.2204; found 600.2215.

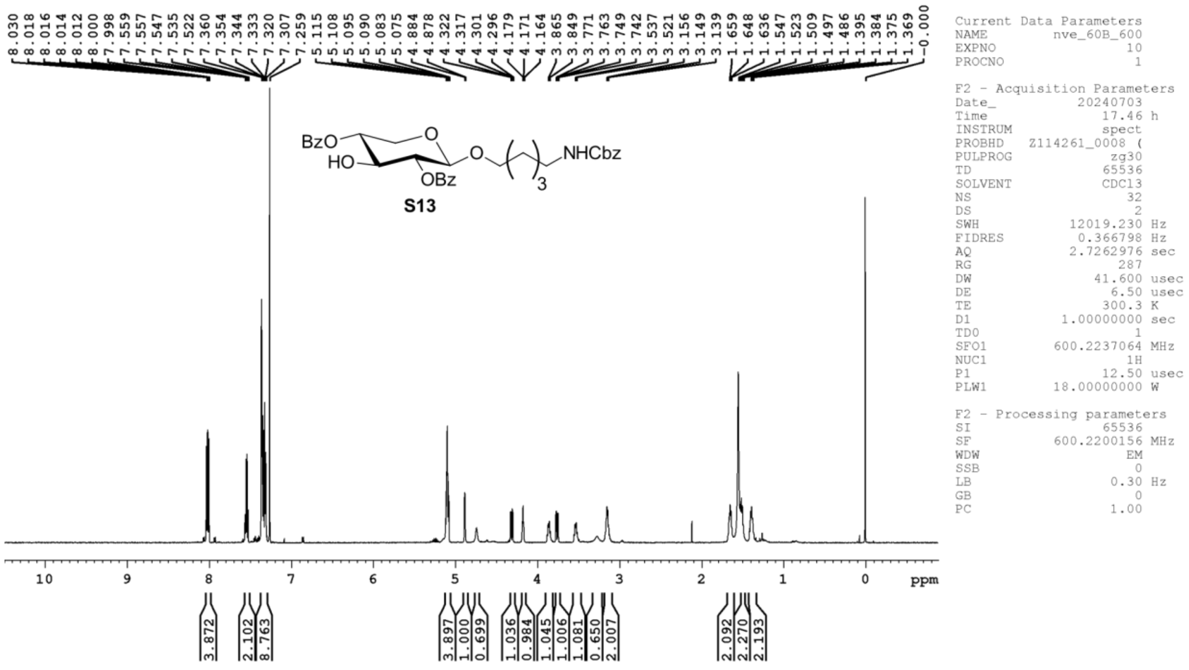

^1H NMR spectrum of compound **S13** (600 MHz, $CDCl_3$)

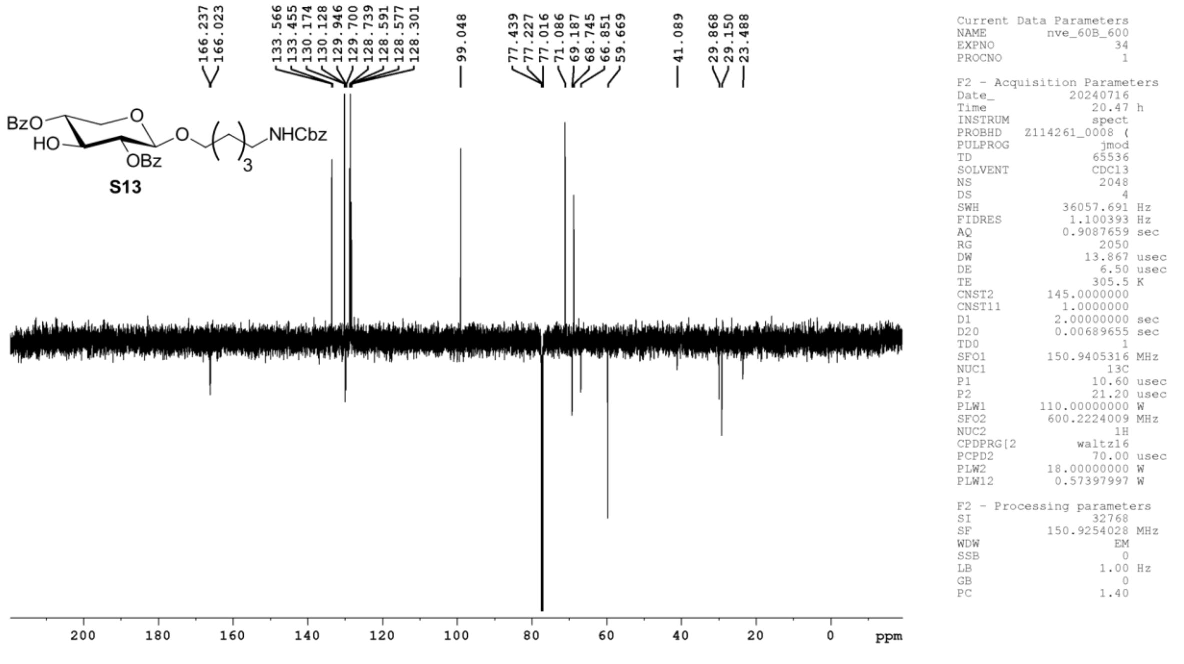

^{13}C APT NMR spectrum of compound **S13** (151 MHz, $CDCl_3$)

Benzyloxycarbonylaminopentyl 2,4-di-*O*-benzoyl-3-*O*-acetyl-β-D-xylopyranoside (**S14**)

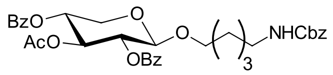

S14

The linker attached capped Bz-protected monosaccharide **S14** was obtained as a side product during AGA reaction trials [entry 9 (1.7 mg, 22%), entry 10 (1.3 mg, 17%), entry 11 (1.4 mg, 18%), entry 13 (0.9 mg, 12%) of Table S4].

Physical description: Glassy solid.

^{1}H NMR (600 MHz, $(CD_3)_2CO$): δ 8.04-7.97 (m, 4H, Ar-H), 7.69-7.62 (m, 2H, Ar-H), 7.54-7.48 (m, 4H, Ar-H), 7.40-7.33 (m, 4H, Ar-H), 7.32-7.27 (m, 1H, Ar-H), 6.17 (br, 1H, N-H), 5.58 (t, *J* = 8.6 Hz, 1H, H-3), 5.24-5.16 (m, 2H, H-2, H-4), 5.04 (s, 2H, CH_2-Cbz), 4.89 (d, *J* = 6.8 Hz, 1H, H-1), 4.31 (dd, *J* = 5.1, 11.8 Hz, 1H, H-5a), 3.83 (ddd, *J* = 6.3, 6.3, 9.9 Hz, 1H, OCH_2), 3.78-3.71 (m, 1H, H-5b), 3.60-3.53 (m, 1H, OCH_2), 3.02-2.95 (m, 2H, CH_2-NHCbz), 1.89 (s, 3H, $COCH_3$) 1.58-1.51 (m, 2H, CH_2), 1.45-1.38 (m, 2H, CH_2), 1.33-1.29 (m, 2H, CH_2) ppm.

^{13}C NMR (151 MHz, $(CD_3)_2CO$): δ 170.4, 166.1, 165.7, 134.47, 134.35, 130.50, 130.47, 129.59, 129.57, 129.25, 128.71, 128.62, 101.6, 72.36, 72.17, 70.86, 69.95, 66.4, 62.7, 41.5, 30.41, 30.28, 30.15, 23.9, 20.7 ppm.

ESI-HRMS: m/z $[M + K]^+$ calcd. for $C_{34}H_{37}NO_{10}K$: 658.2049; found 658.2059.

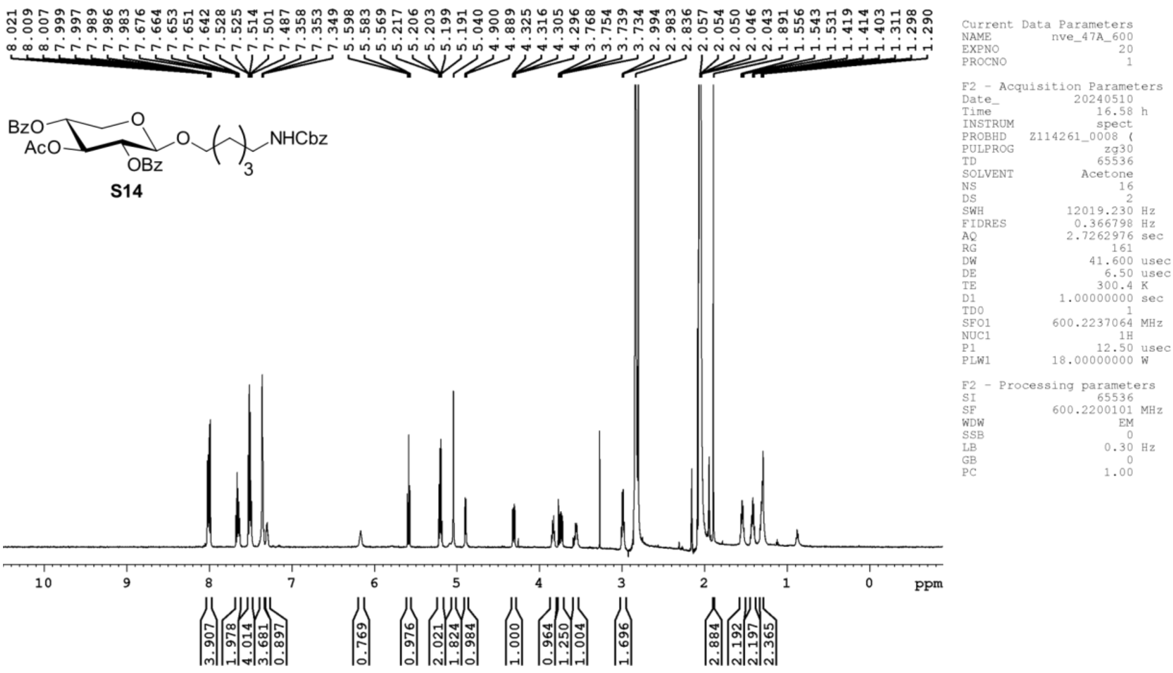

^{1}H NMR spectrum of compound **S14** (600 MHz, $(CD_3)_2CO$)

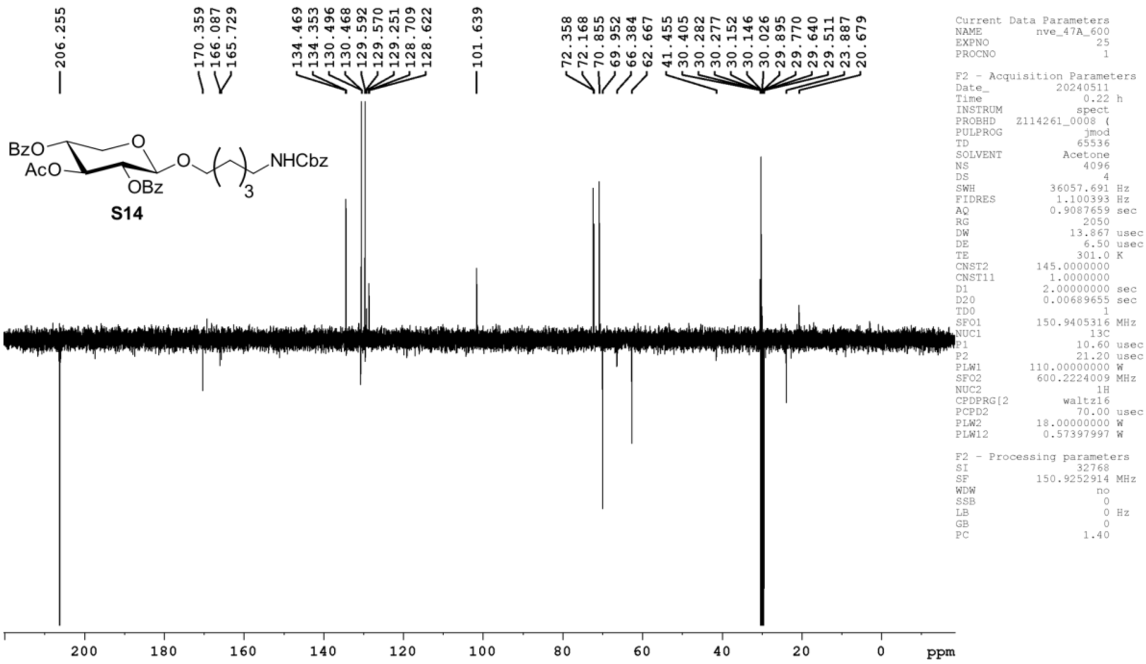

^{13}C APT NMR spectrum of compound **S14** (151 MHz, $(CD_3)_2CO$)

5-[(benzyl)(oxycarbonylamino)]Pentyl acetate (capped linker, **S15**)

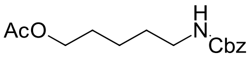

S15

The compound **S15** (0.9 mg, 26% yield, glassy solid) was obtained as a side product during AGA reaction trial in entry 13 of Table S4.

***Rf*:** 0.69 (EtOAc/hexanes = 1/2, v/v).

^{1}H NMR (300 MHz, $(CD_3)_2CO$): δ 7.40-7.26 (m, 5H, Ar-H), 6.33 (s, 1H, N-H), 5.05 (s, 2H, CH_2-Cbz), 4.01 (t, J = 6.6 Hz, 2H, OCH_2), 3.15 (q, J = 6.6 Hz, 2H, C*H*$_2$-NHCbz), 1.97 (s, 3H, $COCH_3$), 1.68-1.48 (m, 4H, CH_2), 1.44-1.35 (m, 2H, CH_2) ppm.

^{13}C NMR (76 MHz, $(CD_3)_2CO$): δ 129.2, 128.62, 128.55, 66.3, 64.6, 41.4, 30.4, 29.1 (merged with solvent peak), 23.8, 20.8 ppm.

^{13}C NMR (151 MHz, $CDCl_3$): δ 128.74, 128.33, 66.9, 64.5, 41.1, 29.9, 28.5, 23.4, 21.2 ppm.

ESI-HRMS: m/z $[M + NH_4]^+$ calcd. for $C_{15}H_{25}N_2O_4$: 297.1809; found 297.1814.

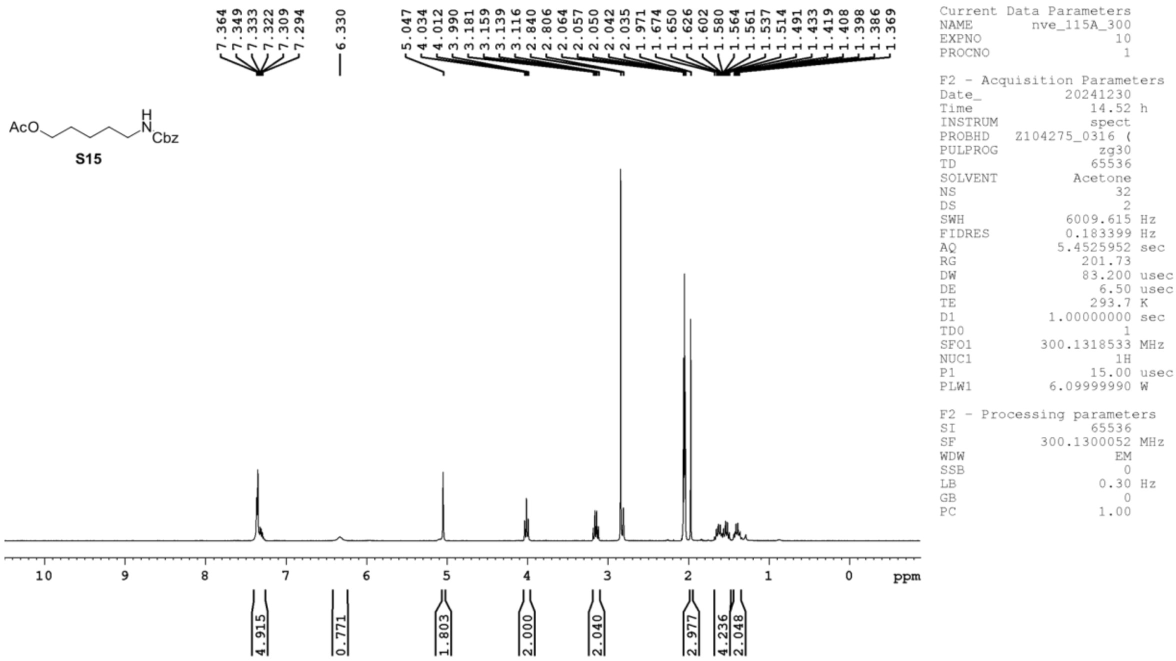

^{1}H NMR spectrum of compound **S15** (300 MHz, $(CD_3)_2CO$)

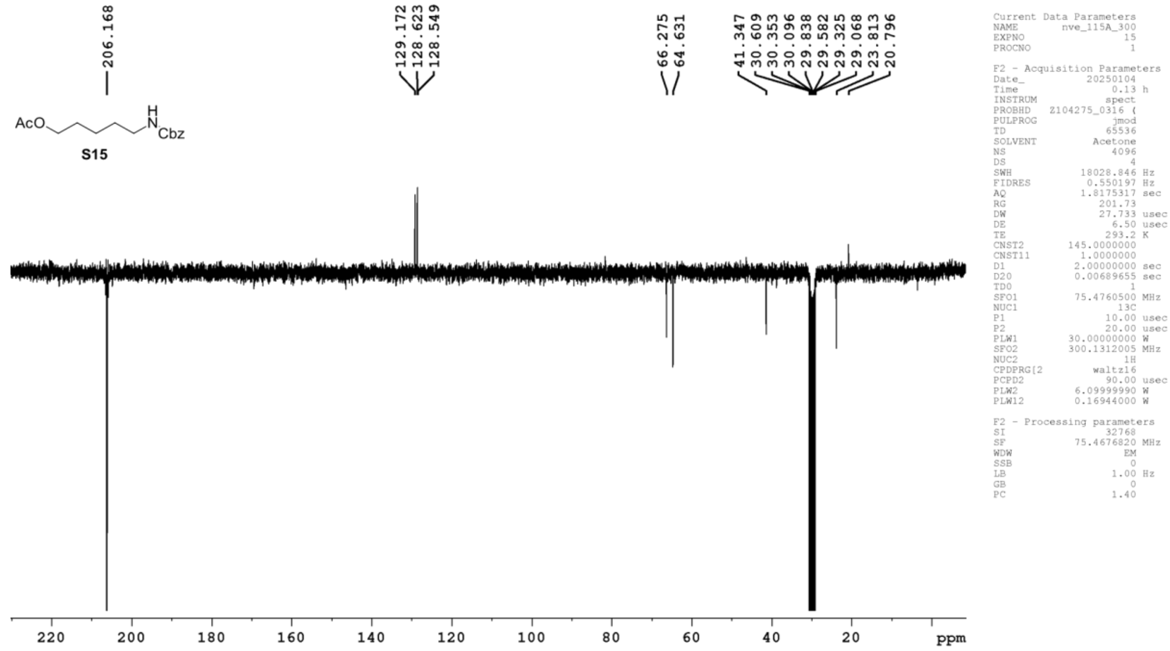

^{13}C APT NMR spectrum of compound **S15** (76 MHz, $(CD_3)_2CO$)

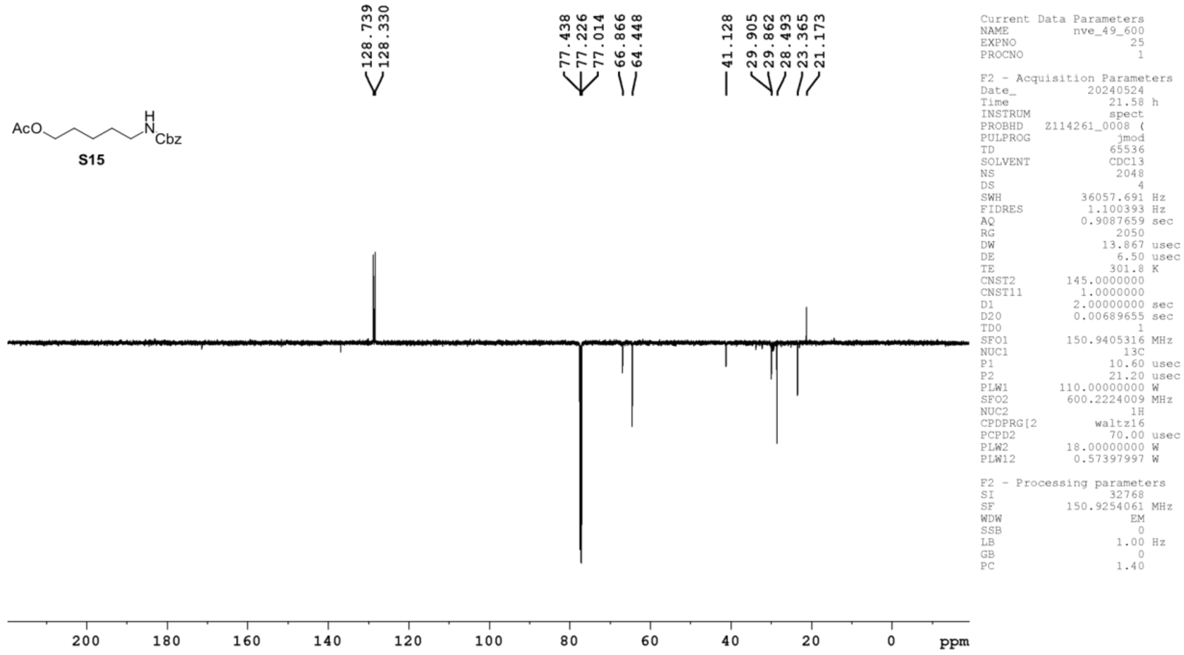

^{13}C APT NMR spectrum of compound **S15** (151 MHz, $CDCl_3$)

Aminopentyl β-D-xylopyranosyl-(1→3)-β-D-xylopyranoside (11)

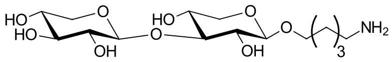

11

Experimental procedure: To a solution of β3-xylan protected disaccharide **4** (3.7 mg, 4.03 µmol) in THF (81 µL, 0.05 M), a solution of NaOMe in MeOH (0.5 M, 81 µL, 40 µmol) was added at rt, and the reaction mixture was stirred. After confirmation of reaction completion (43 h) by TLC (R_f: 0.44 (MeOH/$CHCl_3$ = 1/5, v/v)), the reaction mixture was neutralized by the addition of IR-120 H^+ resin while slowly stirring the mixture. Then, the reaction mixture was filtered, and the filtrate was concentrated under reduced pressure to yield a partially-deprotected glassy crude, which was kept under high vacuum until usage in the next step without any further purification. To a solution of the partially deprotected crude (4.03 µmol) in *t*-BuOH (0.53 mL), H_2O (0.27 mL) and AcOH (5 µL) was added unreduced 10% Pd/C (6 mg), and the reaction mixture was stirred under H_2-atmosphere (using atmospheric pressure). After 3 h, the reaction mixture was filtered using a PTFE syringe filter (0.45 µm) and concentrated under reduced pressure to yield a crude product, which was purified using a HILIC-HPLC column chromatography (13 mM NH_4OAc/ACN = 1/4 to 1/1, v/v). The purified product was lyophilized to give β3-xylan disaccharide **11** (2.0 mg, quantitative yield over 2 steps) as a white amorphous solid.

^{1}H NMR (600 MHz, D_2O): δ 4.68 (d, J = 7.8 Hz, 1H, H-1B), 4.45 (d, J = 7.9 Hz, 1H, H-1A), 4.03-3.97 (m, 2H, H-5A, H-5B), 3.92-3.87 (m, 1H, OCH_2), 3.74-3.61 (m, 4H, H-3A, H-4A, OCH_2), 3.50-3.43 (m, 2H, H-2A), 3.38-3.30 (m, 3H, H-2B, H-5A, H-5B), 2.97 (t, J = 7.5 Hz, 2H, CH_2-NH_2), 1.72-1.63 (m, 4H, CH_2), 1.49-1.42 (m, 2H, CH_2) ppm.

^{13}C NMR (151 MHz, D_2O): δ 104.1, 103.3, 84.6, 76.3, 74.0, 73.3, 70.84, 69.84, 68.3, 65.82, 65.40, 40.1, 28.9, 27.7, 22.8 ppm.

ESI-HRMS: m/z [M + K]$^+$ calcd. for $C_{15}H_{29}NO_9K$: 406.1474; found 406.1472.

NMR chemical shifts of selected ^{1}H and ^{13}C atoms in compound **11**:

xylose ring	proton	δ (ppm)	multiplicity	J (Hz)	carbon	δ (ppm)
A (reducing end)	H-1A	4.45	d	7.9	C-1A	103.3
	H-2A	3.50-3.43	m		C-2A	73.3
	H-3A	3.74-3.61	m			
	H-4A	3.74-3.61	m			
	H-5Aa	3.38-3.30	m			
	H-5Ab	4.03-3.97	m			
B (non-reducing end)	H-1B	4.68	d	7.8	C-1B	104.1
	H-2B	3.38-3.30	m			
	H-5Ba	3.38-3.30	m			
	H-5Bb	4.03-3.97	m			

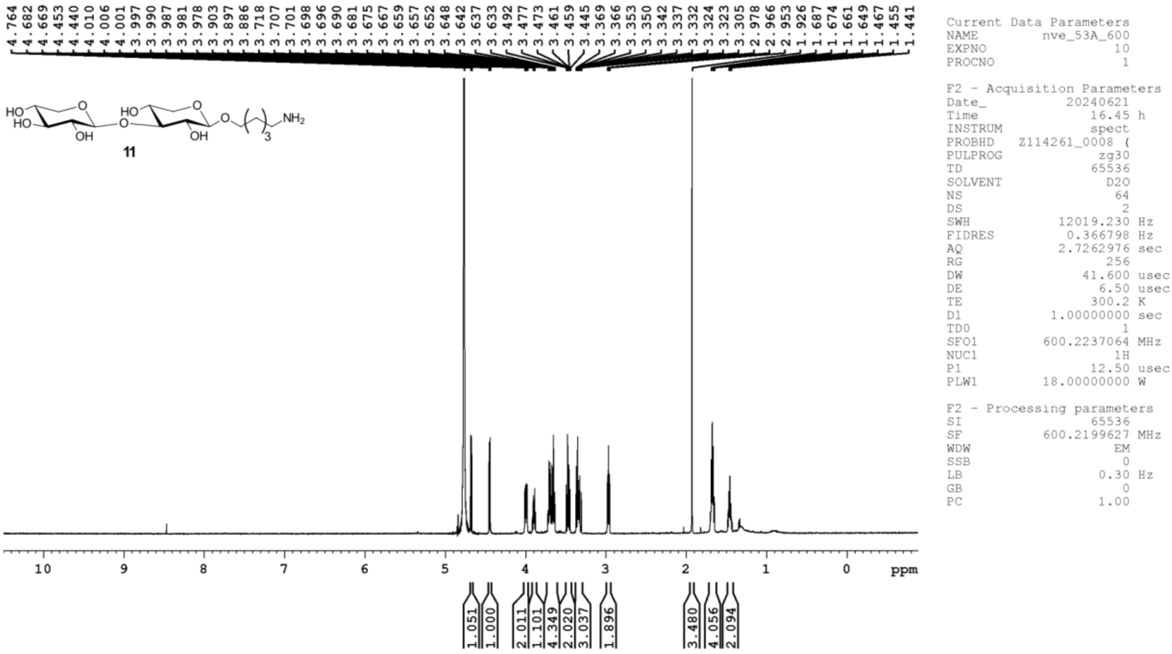

[1] H NMR spectrum of compound **11** (600 MHz, D_2O)

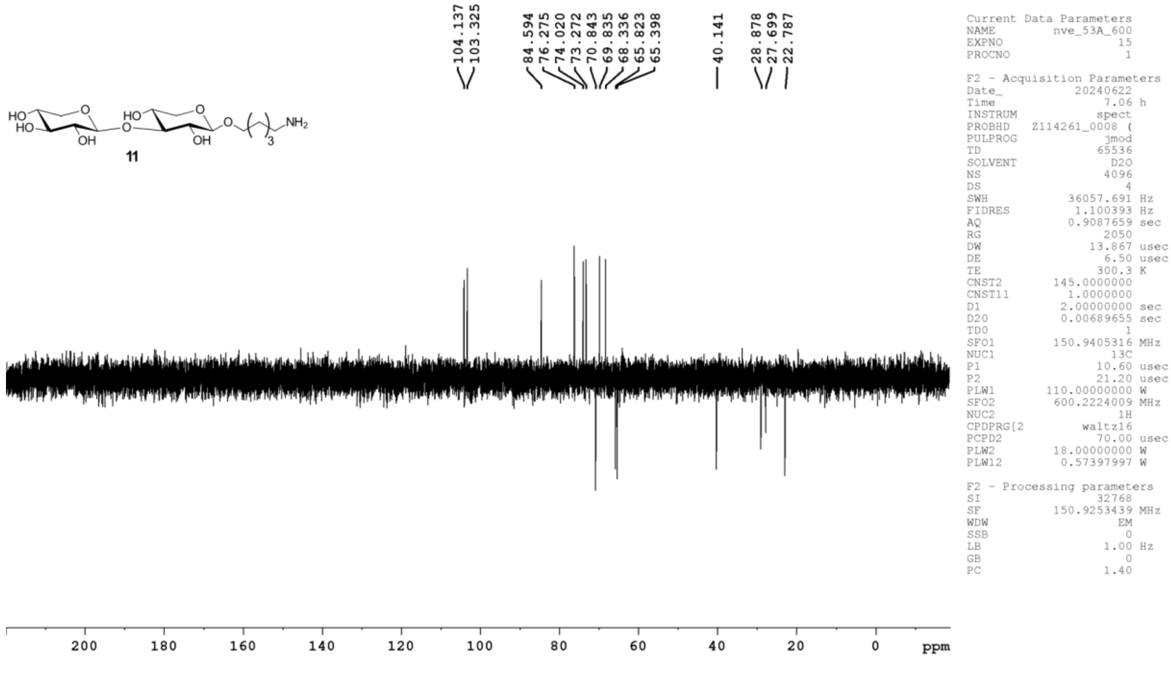

[13] C APT NMR spectrum of compound **11** (151 MHz, D_2O)

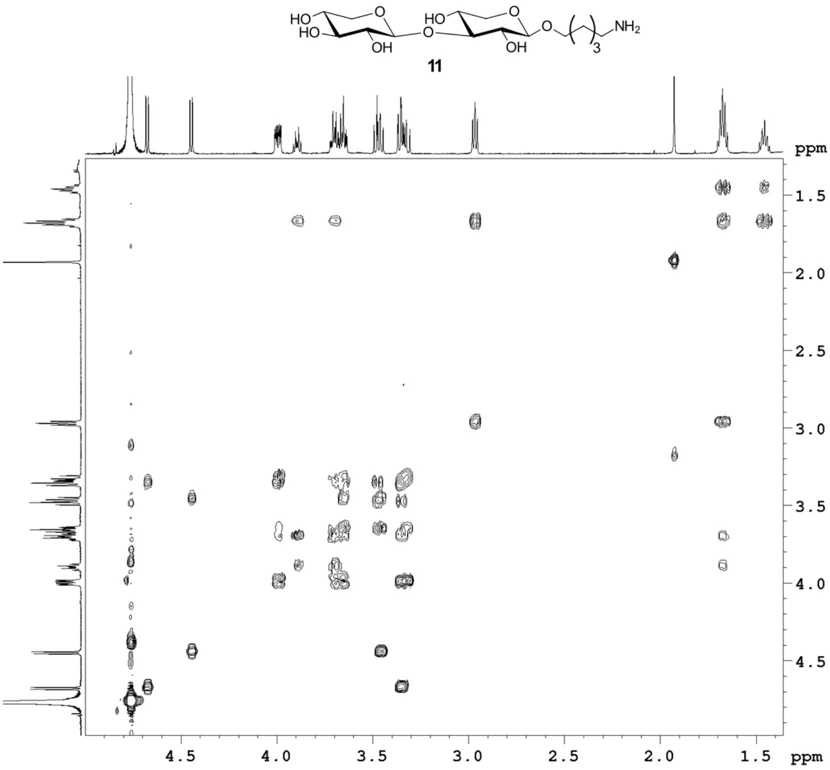

^{1}H-^{1}H COSY NMR spectrum of compound **11** (600 MHz, D_2O)

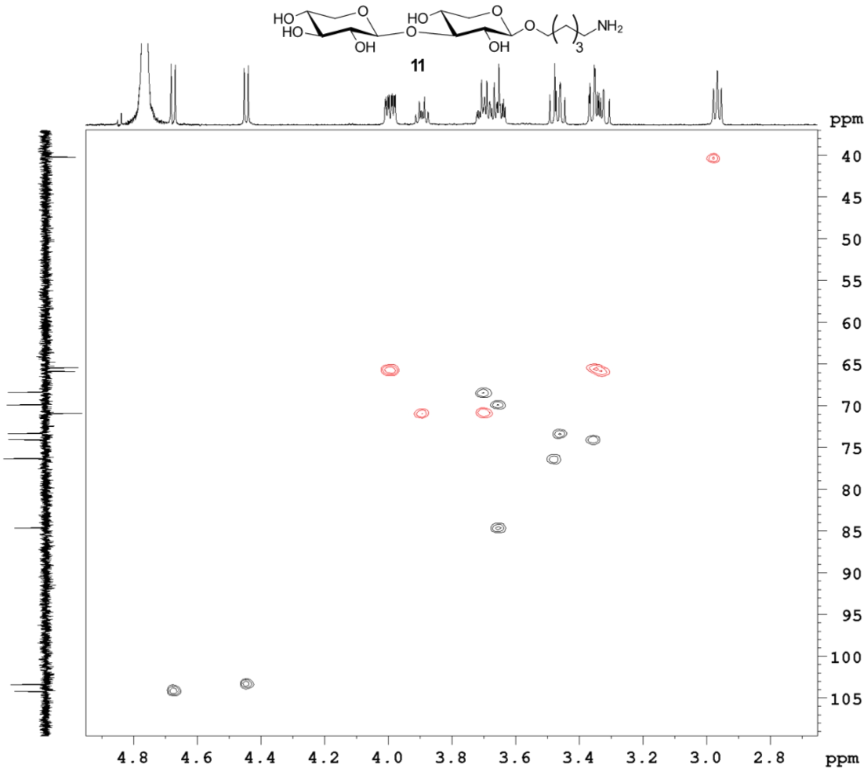

^{1}H-^{1}H HSQC NMR spectrum of compound **11** (600/151 MHz, D_2O)

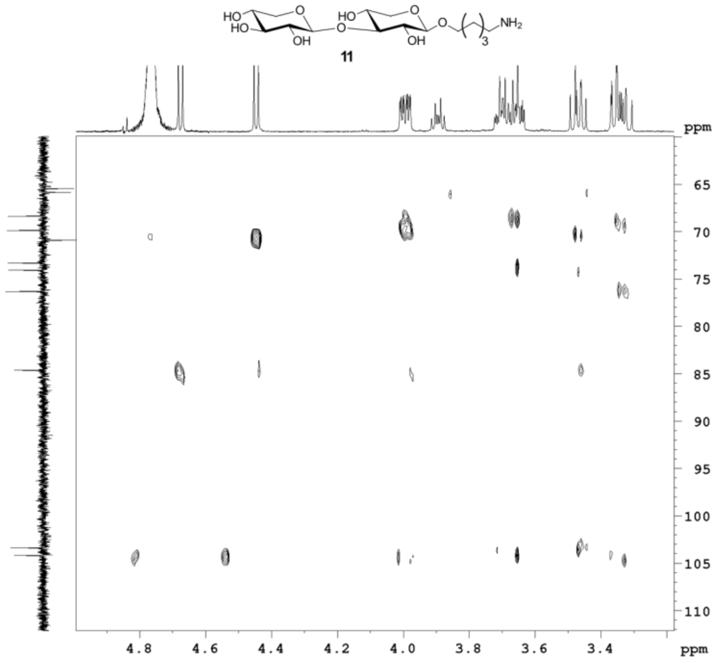

^{1}H-^{13}C HMBC NMR spectrum of compound **11** (600/151 MHz, D_2O)

AGA of Bz-protected β3-xylan tetrasaccharide 5 (entry 6, Table S4)

Benzyloxycarbonylaminopentyl 2,4-di-*O*-benzoyl-β-D-xylopyranosyl-(1→3)-2,4-di-*O*-benzoyl-β-D-xylopyranosyl-(1→3)-2,4-di-*O*-benzoyl-β-D-xylopyranosyl-(1→3)-2,4-di-*O*-benzoyl-β-D-xylopyranoside (**5**)

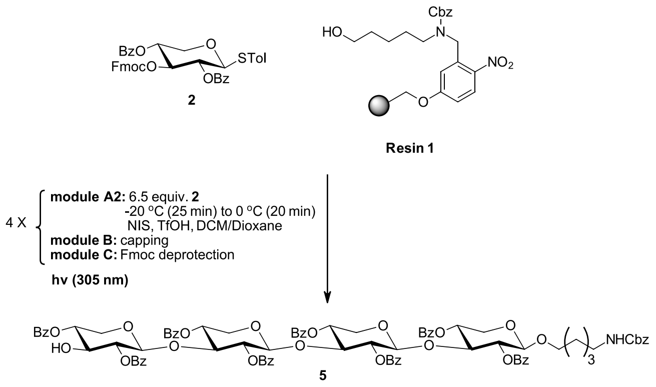

Experimental procedure: Linker-functionalized resin **1** (40 mg, 13.2 μmol) was placed in the synthesizer and synthesizer modules were applied as follows: 4 × [module A2 (BB **2**; total amount used = 236 mg, 0.34 mmol, 26 equiv.) at –20 °C (25 min) to 0 °C (20 min), module B, and module C]. Cleavage from the resin using UV irradiation at 305 nm in a continuous flow photoreactor afforded the crude product. Purification of the crude by normal phase HPLC (EtOAc/hexanes = 1/2.3 to 1/1, v/v) using a preparative YMC-Small column gave protected β3-xylan tetrasaccharide **5** (1.8 mg, 9% yield over 9 steps) as a glassy solid.

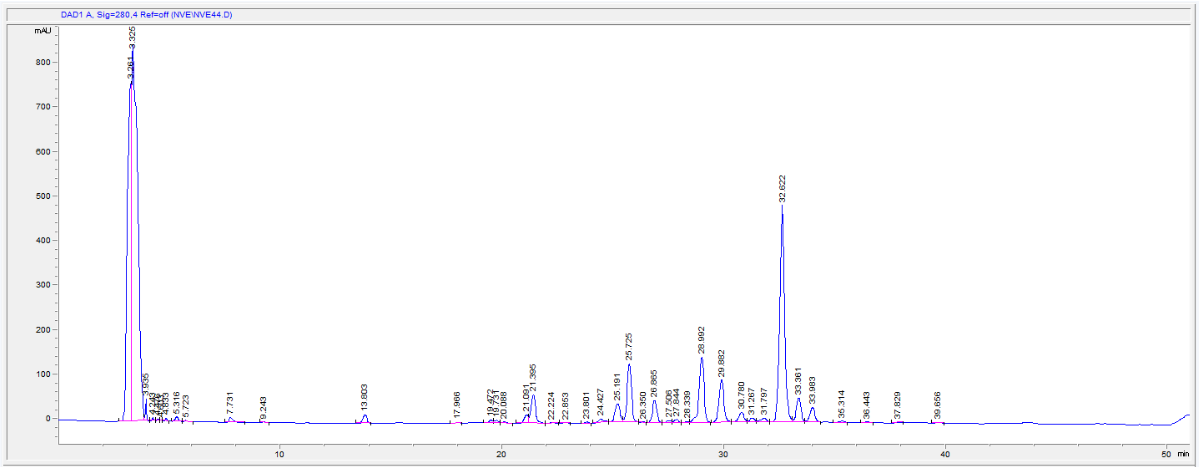

Analytical HPLC of the crude reaction mixture was performed using a YMC-Small NP column and a gradient of EtOAc in hexanes (50 min, flow rate 1 mL/min). β3-xylan tetrasaccharide **5** eluted at 32.6 min.

^{1}H NMR (600 MHz, $(CD_3)_2CO$): δ 8.15-8.11 (m, 2H, Ar-H), 8.03-7.99 (m, 4H, Ar-H), 7.97-7.93 (m, 2H, Ar-H), 7.92-7.88 (m, 2H, Ar-H), 7.87-7.83 (m, 2H, Ar-H), 7.74-7.71 (m, 2H, Ar-H), 7.69-7.63 (m, 3H, Ar-H), 7.63-7.57 (m, 3H, Ar-H), 7.56-7.50 (m, 5H, Ar-H), 7.49-7.45 (m, 4H, Ar-H), 7.41-7.37 (m, 3H, Ar-H), 7.37-7.26 (m, 11H, Ar-H), 7.16-7.12 (m, 2H, Ar-H), 6.13 (br, 1H, N-H), 5.11-4.91 (m, 12H, H-2A, H-2B, H-2C, H-2D, H-4A, H-4C, H-4D, CH_2-Cbz, H-1B, H-1C, H-1D), 4.88 (d, J = 5.8 Hz, 1H, O-H), 4.70-4.66 (m, 2H, H-1A, H-4B), 4.35 (t, J = 7.6 Hz, 1H, H-3A), 4.24 (t, J = 5.3 Hz, 1H, H-3C), 4.22-4.17 (m, 2H, H-3B), 4.16-4.10 (m, 2H, H-3D), 4.09-4.06 (m, 1H), 3.96 (dd, J = 4.4, 12.5 Hz, 1H), 3.67-3.62 (m, 1H, OCH_2), 3.58 (dd, J = 7.9, 12.0 Hz, 1H), 3.48-3.39 (m, 3H), 3.38-3.32 (m, 1H, OCH_2), 2.96-2.89 (m, 2H, CH_2-NHCbz), 1.42-1.32 (m, 4H, CH_2), 1.18-1.12 (m, 2H, CH_2) ppm.

^{13}C NMR (151 MHz, $(CD_3)_2CO$): δ 165.11, 165.03, 134.24, 134.15, 134.10, 133.94, 133.86, 133.69, 130.93, 130.89, 130.86, 130.79, 130.76, 130.66, 130.59, 130.57, 130.46, 130.41, 130.16, 130.13, 129.46, 129.37, 129.35, 129.29, 129.25, 129.20, 129.15, 129.03, 128.64, 128.56, 101.23, 101.20, 100.3, 99.9, 77.4, 76.2, 73.8, 72.92, 72.69, 72.13, 71.35, 71.20, 71.05, 70.42, 70.2, 69.4, 66.3, 63.0, 62.22, 62.07, 60.5, 41.4, 30.3, 23.8 ppm.

ESI-HRMS: m/z $[M + H]^+$ calcd. for $C_{89}H_{84}NO_{27}$: 1598.5225; found 1598.5283.

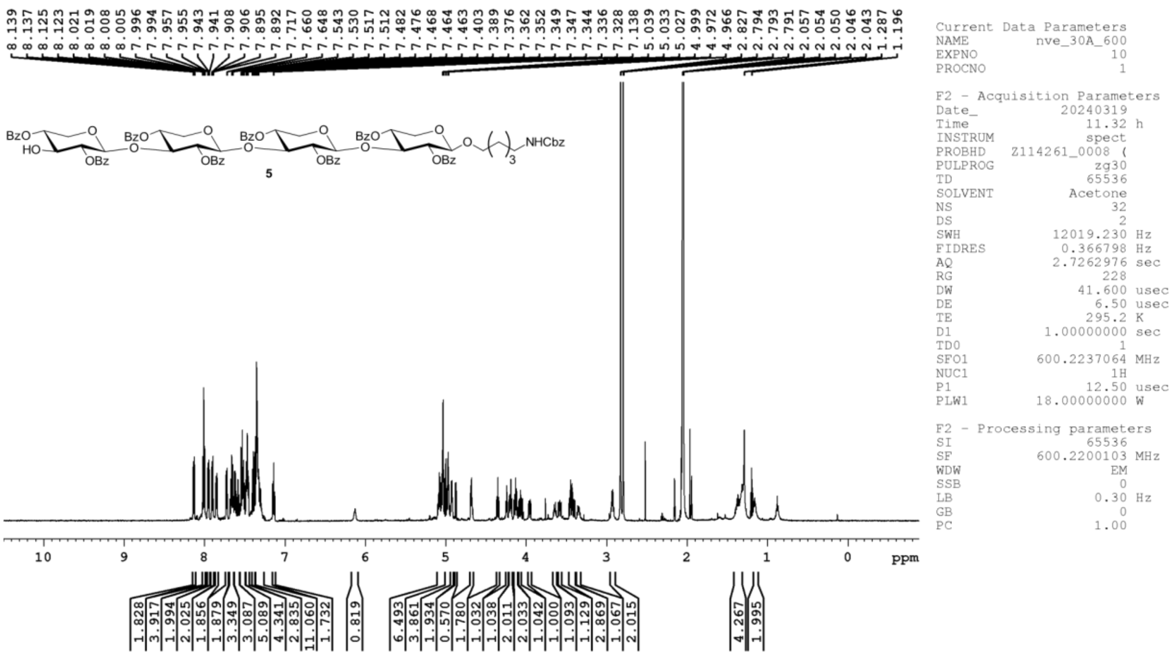

^{1}H NMR spectrum of compound **5** (600 MHz, $(CD_3)_2CO$)

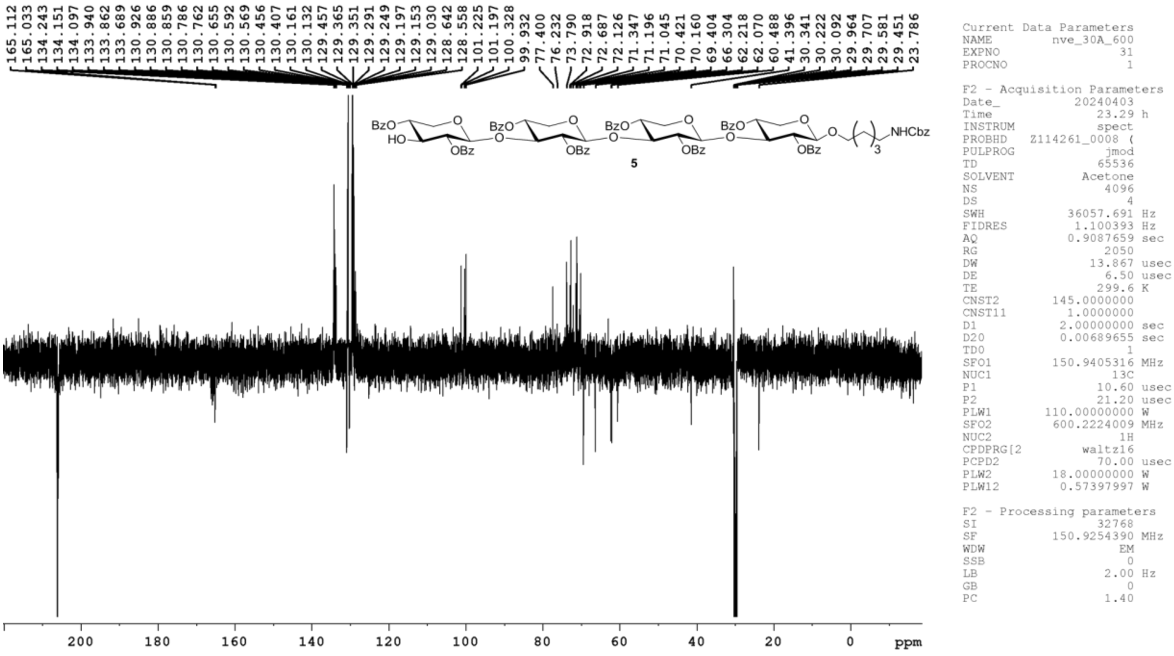

^{13}C APT NMR spectrum of compound **5** (151 MHz, $(CD_3)_2CO$)

Aminopentyl β-D-xylopyranosyl-(1→3)-β-D-xylopyranosyl-(1→3)-β-D-xylopyranosyl-(1→3)-β-D-xylopyranoside (12)

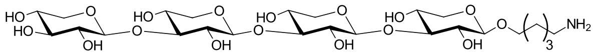

12

Experimental procedure: To a solution of β3-xylan protected tetrasaccharide **5** (2.0 mg, 1.25 μmol) in THF (80 μL, 0.016 M), solution of NaOMe in MeOH (0.5 M, 80 μL, 40 μmol) was added at rt, and reaction mixture was allowed to stir. After confirmation of reaction completion (28 h) by TLC (R_f: 0.27 (MeOH/$CHCl_3$ = 1/5, v/v)), reaction mixture pH was neutralized by the addition of IR-120 H^+ resin while slowly stirring the mixture. Then, the reaction mixture was filtered, and the filtrate was concentrated under reduced pressure to yield a partially deprotected glassy crude, which was kept under high vacuum until usage in the next step without any further purification. To a solution of the partially deprotected crude (1.25 μmol) in *t*-BuOH (0.17 mL), H_2O (0.08 mL), and AcOH (one drop) was added unreduced 10% Pd/C (1.3 mg) and the reaction mixture was stirred under H_2-atmosphere (using atmospheric pressure). After 4 h, the reaction mixture was filtered using a PTFE syringe filter (0.45 μm) and concentrated under reduced pressure to yield a crude product, which was purified using HILIC-HPLC column chromatography (13 mM NH_4OAc/ACN = 1/4 to 1/1, v/v). The purified product was lyophilized to give β3-xylan tetrasaccharide **12** as a white amorphous solid (1.39 mg, quantitative yield over 2 steps).

^{1}H NMR (600 MHz, D_2O): δ 4.74-4.69 (m, 3H, H-1B, H-1C, H-1D), 4.44 (d, J = 7.9 Hz, 1H, H-1A), 4.05-3.97 (m, 4H, H-5A, H-5B, H-5C, H-5D), 3.92-3.87 (m, 1H, OCH_2), 3.76-3.63 (m, 10H, OCH_2), 3.58-3.52 (m, 2H), 3.50-3.43 (m, 2H, H-2A), 3.38-3.30 (m, 5H), 3.00 (t, J = 7.5 Hz, 2H, CH_2-NH_2), 1.73-1.65 (m, 4H, CH_2), 1.50-1.43 (m, 2H, CH_2) ppm.

^{13}C NMR (151 MHz, D_2O): δ 104.1, 103.87, 103.82, 103.34, 84.47, 84.34, 84.22, 76.3, 74.0, 73.73, 73.67, 73.35, 70.8, 69.9, 68.3, 65.82, 65.48, 65.47, 65.45, 65.43, 40.0, 28.8, 27.0, 22.7 ppm.

ESI-HRMS: m/z $[M + H]^+$ calcd. for $C_{25}H_{46}NO_{17}$: 632.2760; found 632.2774.

NMR chemical shifts of selected ^{1}H and ^{13}C atoms in compound **12**:

**xylose ring**	**proton**	**δ (ppm)**	**multiplicity**	**J (Hz)**	**carbon**	**δ (ppm)**
A (reducing end)	H-1A	4.44	d	7.9	C-1A	103.34
	H-2A	3.50-3.43	m			
	H-5Ab	4.05-3.97	m			
B	H-1B	4.74-4.69	m			
	H-5Bb	4.05-3.97	m			
C	H-1C	4.74-4.69	m			
	H-5Cb	4.05-3.97	m			
D	H-1D	4.74-4.69	m			
	H-5Db	4.05-3.97	m			

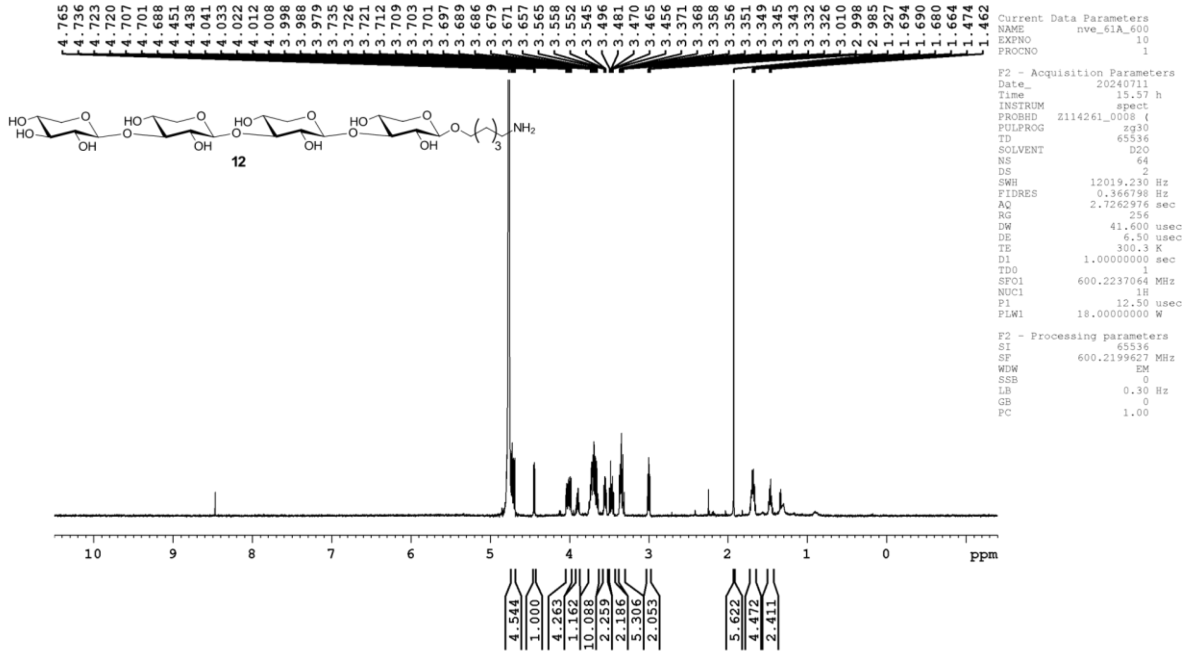

^{1}H NMR spectrum of compound **12** (600 MHz, D_2O)

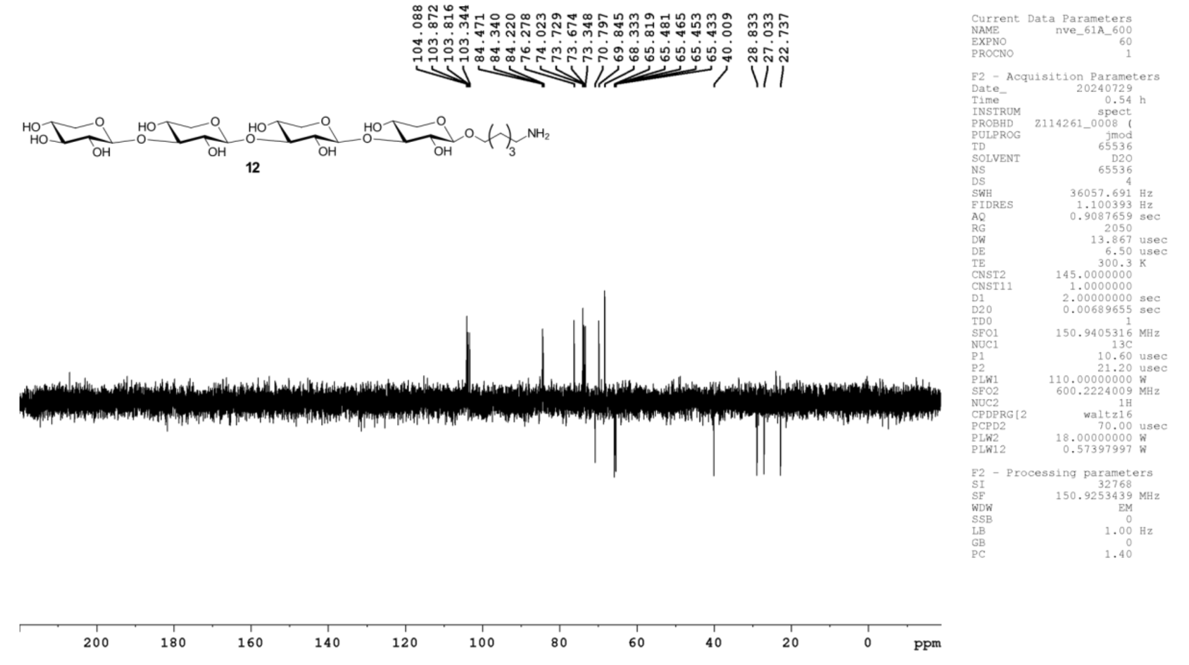

^{13}C APT NMR spectrum of compound **12** (151 MHz, D_2O)

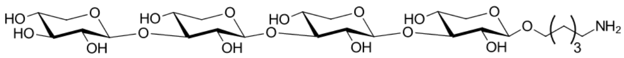

12

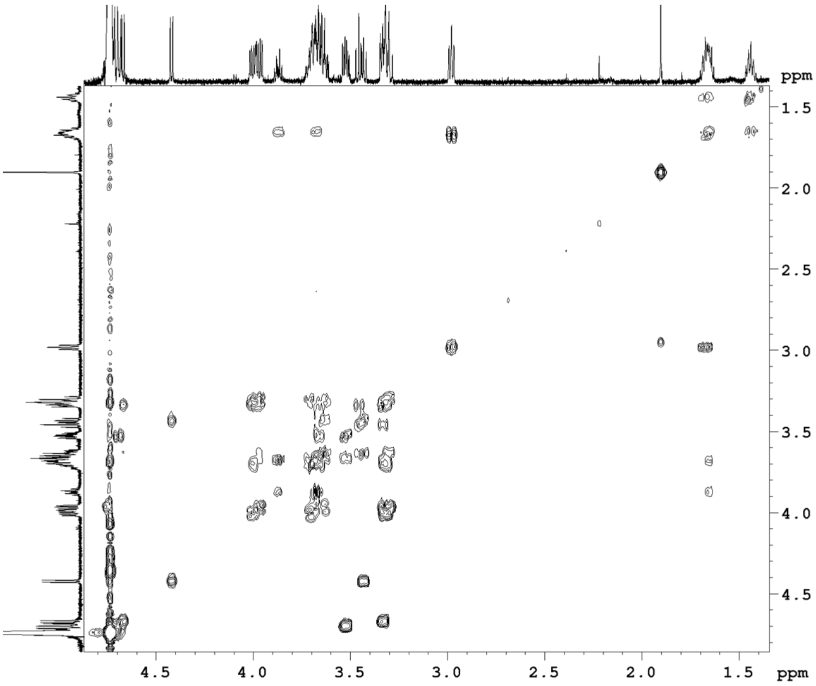

^{1}H-^{1}H COSY NMR spectrum of compound **12** (600 MHz, D_2O)

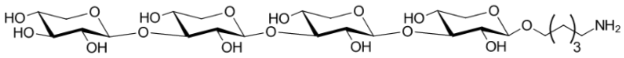

12

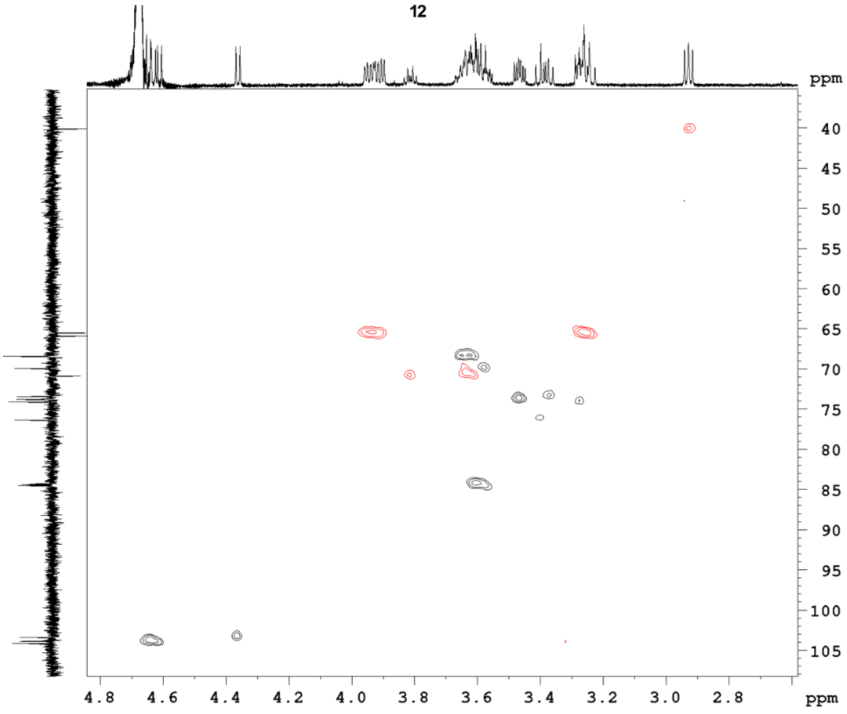

^{1}H-^{13}C HSQC NMR spectrum of compound **12** (600/151 MHz, D_2O)

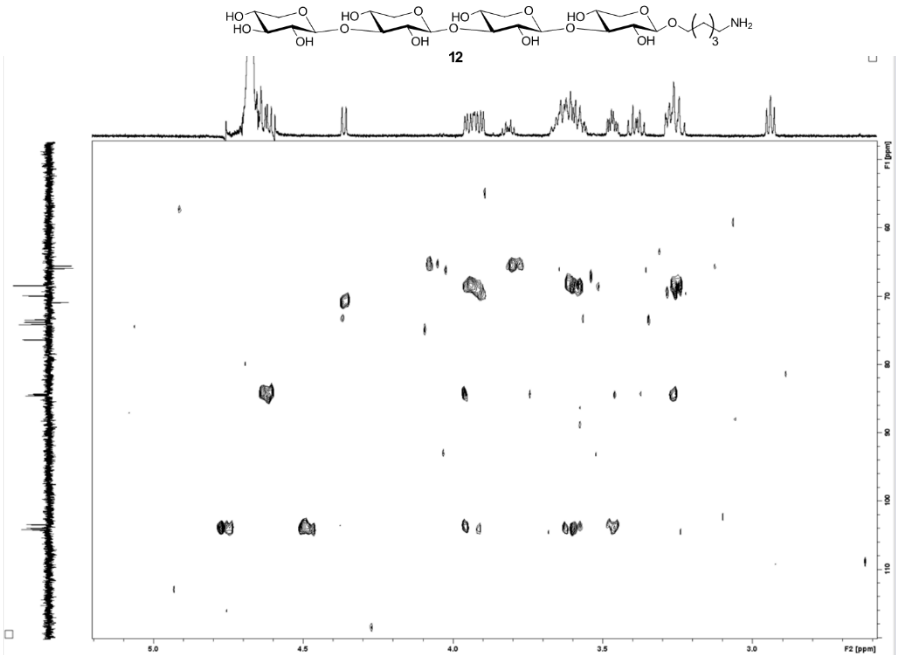

^{1}H-^{13}C HMBC NMR spectrum of compound **12** (600/151 MHz, D_2O)

4.2 AGA of β3-xylan oligosaccharides using Bn-protected armed xylose donors

AGA of Bn-protected β3-xylan disaccharide 7 using thioxyloside donor S10

Benzyloxycarbonylaminopentyl 2-*O*-benzoyl-4-*O*-benzyl-β-D-xylopyranosyl-(1→3)-2-*O*-benzoyl-4-*O*-benzyl-β-D-xylopyranoside (**7**)

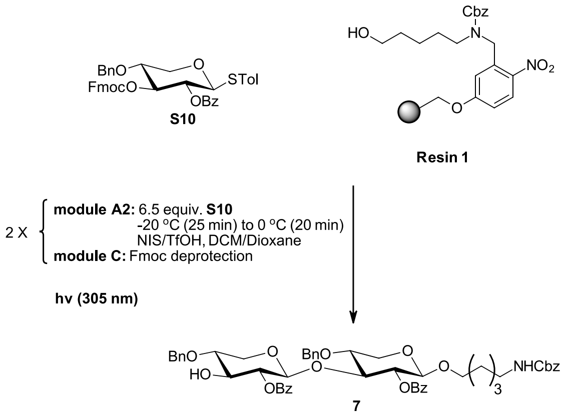

Experimental procedure: Linker-functionalized resin **1** (38.0 mg, 12.5 µmol) was placed in the synthesizer and synthesizer modules were applied as follows: 2 × [module A2 (BB **S10**; total amount used = 109 mg, 0.1625 mmol, 13 equiv.) at –20 °C (25 min) to 0 °C (20 min) and module C]. Cleavage from the resin using UV irradiation at 305 nm in a continuous flow photoreactor afforded the crude product. Purification of the crude by normal phase HPLC using a preparative YMC-Small column (EtOAc/hexanes = 1/9 to 1/1.5, v/v) gave protected β3-xylan disaccharide **7** (4.1 mg, 37% yield over 5 steps) as a glassy solid and linker-attached monosaccharide **S16** (1.7 mg, 24% yield) as a side product.

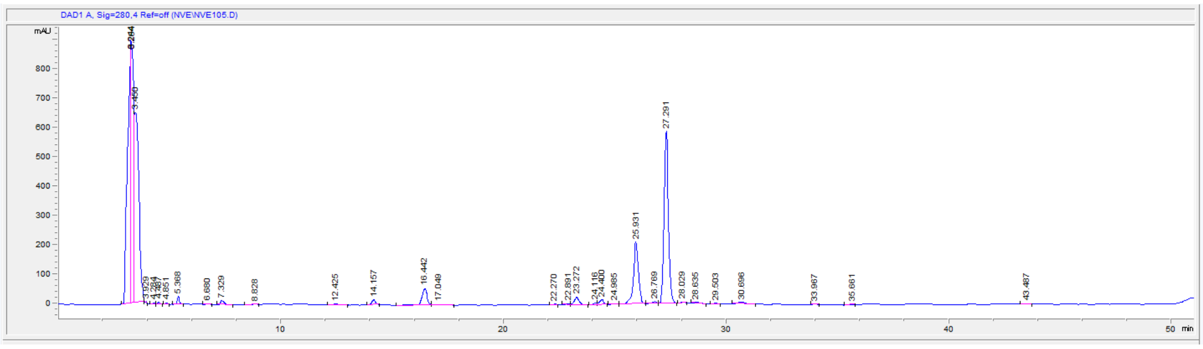

Analytical HPLC of the crude reaction mixture was performed on a YMC-Small NP column using a gradient of EtOAc in hexanes (50 min, flow rate 1 mL/min). Linker-attached monosaccharide **S16** as a side product was eluted at 25.9 min. Desired disaccharide **7** was eluted at 27.3 min.

Benzyloxycarbonylaminopentyl 2-*O*-benzoyl-4-*O*-benzyl-β-D-xylopyranoside (**S16**)

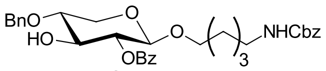

S16

The linker-attached Bn-protected monosaccharide **S16** (1.7 mg as a glassy solid, 24% yield with respect to mmoles of linker hydroxyl on resin) was obtained as a side product during AGA reaction trial for the synthesis of disaccharide **7** using Bn-protected xylose-thiotolyl donor **S10**.

^{1}H NMR (300 MHz, $(CD_3)_2CO$): δ 8.1-8.0 (m, 2H, Ar-H), 7.67-7.58 (m, 1H, Ar-H), 7.55-7.47 (m, 2H, Ar-H), 7.43-7.23 (m, 10H, Ar-H), 6.13 (br, 1H, N-H), 5.06-4.94 (m, 3H, CH_2-Cbz, H-2), 4.86-4.69 (m, 3H, CH_2-Ph, O-H), 4.57 (d, J = 7.7 Hz, 1H, H-1), 4.04 (dd, J = 5.2, 11.4 Hz, 1H, H-5a), 3.95-3.84 (m, 1H, H-3), 3.82-3.70 (m, 1H, OCH_2), 3.66-3.54 (m, 1H, H-4), 3.5-3.41 (m, 1H, OCH_2), 3.39-3.28 (m, 1H, H-5b), 2.99-2.85 (m, 2H, $C\mathit{H}_2$-NHCbz), 1.53-1.40 (m, 2H, CH_2), 1.39-1.30 (m, 2H, CH_2), 1.29-1.16 (m, 2H, CH_2) ppm.

^{13}C NMR (76 MHz, $(CD_3)_2CO$): δ 133.8, 130.4, 129.32, 129.17, 129.03, 128.62, 128.54, 128.51, 128.26, 102.3, 78.8, 75.30, 75.10, 73.6, 69.7, 66.2, 64.5, 41.3, 30.25, 29.6 (merged with acetone peak), 23.8 ppm.

ESI-HRMS: m/z [M + HCOO]$^-$ calcd. for $C_{33}H_{38}NO_{10}$: 608.2501; found 608.2503.

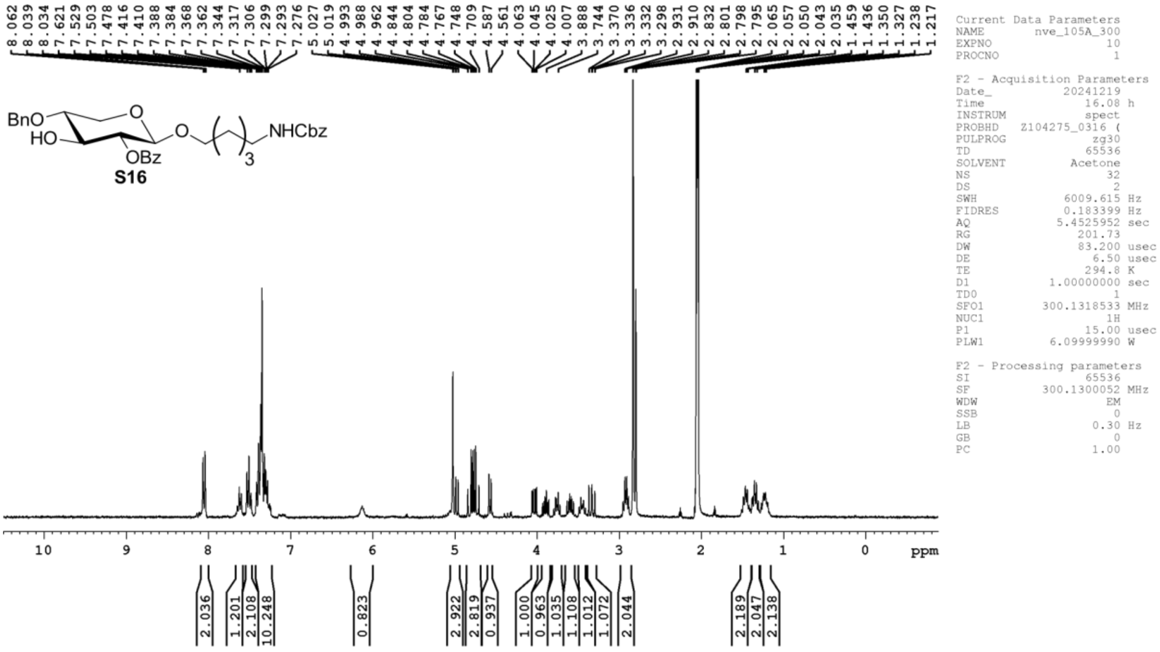

^{1}H NMR spectrum of compound **S16** (300 MHz, $(CD_3)_2CO$)

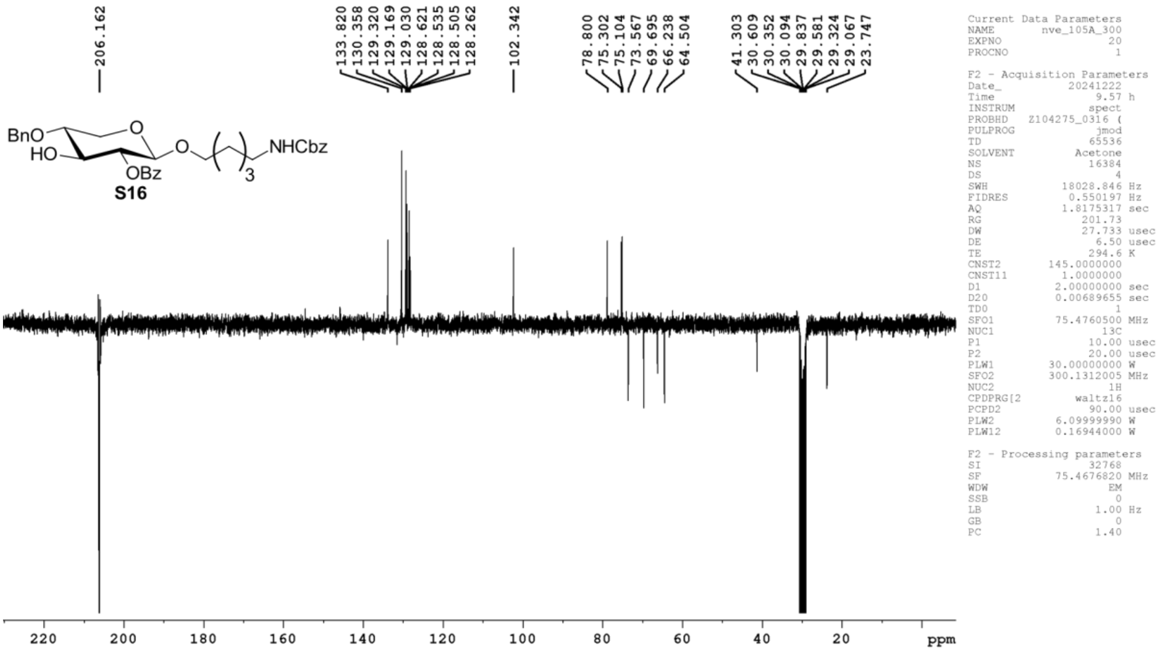

^{13}C APT NMR spectrum of compound **S16** (76 MHz, $(CD_3)_2CO$)

AGA of Bn-protected β3-xylan disaccharide 7 using xylose-phosphate donor 6

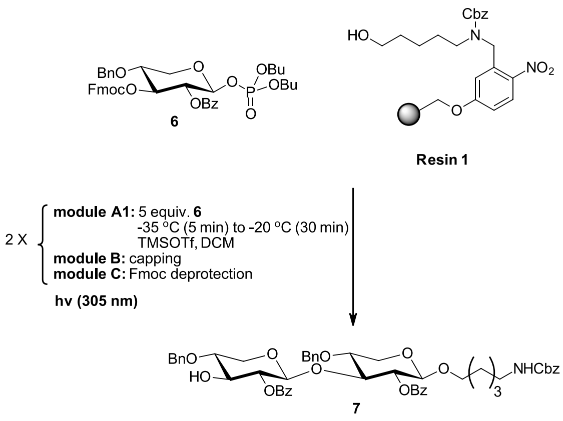

Experimental procedure: Linker-functionalized resin **1** (38.0 mg, 12.5 μmol) was placed in the synthesizer and synthesizer modules were applied as follows: 2 × [module A1 (BB **6**; total amount used = 95 mg, 125 μmol, 10 equiv.) at –35 °C (5 min) to –20 °C (30 min), module B, and module C]. Cleavage from the resin using UV irradiation at 305 nm in a continuous flow photoreactor afforded the crude product. Purification of the crude by normal phase HPLC using a preparative YMC-Small column (EtOAc/hexanes = 1/9 to 1/1.5, v/v) gave protected β3-xylan disaccharide **7** (8.1 mg, 73% yield over 5 steps) as a glassy solid.

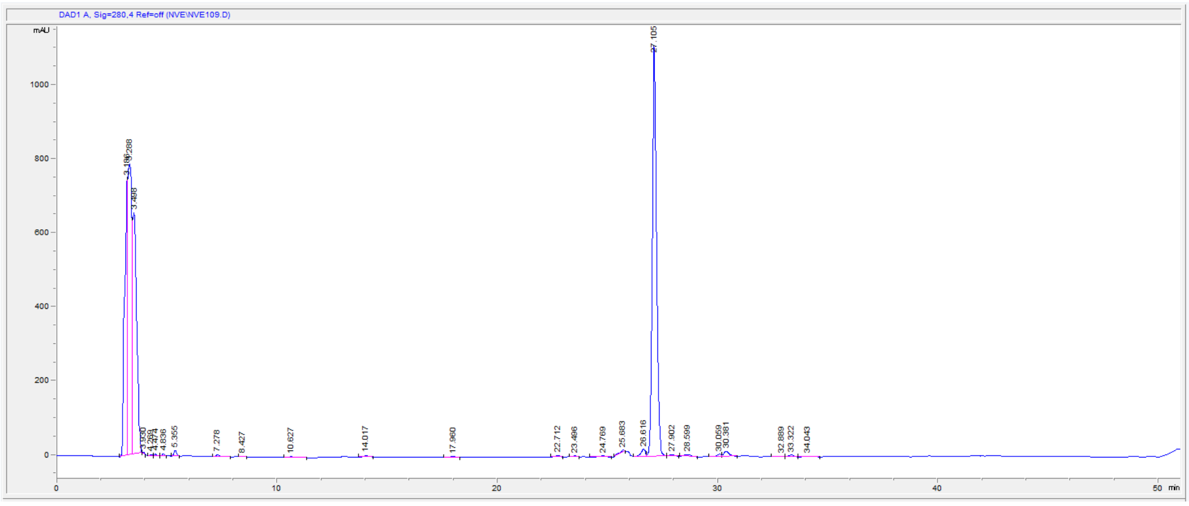

Analytical HPLC of the crude reaction mixture was performed on a YMC-Small NP column using a gradient of EtOAc in hexanes (50 min, flow rate 1 mL/min).

^{1}H NMR (300 MHz, $(CD_3)_2CO$): δ 8.01-7.94 (m, 2H, Ar-H), 7.82-7.75 (m, 2H, Ar-H), 7.66-7.54 (m, 2H, Ar-H), 7.51-7.39 (m, 6H, Ar-H), 7.38-7.23 (m, 13H, Ar-H), 6.12 (br, 1H, N-H), 5.08-4.94 (m, 4H, CH_2-Cbz, H-2B, H-2A), 4.93-4.82 (m, 2H, H-1B, CH_2-Ph), 4.79-4.63 (m, 4H, CH_2-Ph, O-H), 4.53 (d, J = 6.9 Hz, 1H, H-1A), 4.18 (t, J = 8.1 Hz, 1H, H-3A), 4.1-4.04 (m, 1H, H-5B), 4.03-3.96 (m, 1H, H-5A), 3.72-3.52 (m, 4H, H-3B, H-4A, OCH_2, H-4B), 3.44-3.35 (m, 1H, H-5A), 3.34-3.23 (m, 2H, OCH_2, H-5B), 2.94-2.79 (m, 2H, CH_2-NHCbz, merged with H_2O peak in acetone), 1.39-1.23 (m, 4H, CH_2), 1.17-1.05 (m, 2H, CH_2) ppm.

^{13}C NMR (76 MHz, $(CD_3)_2CO$): δ 165.79, 165.25, 139.9, 133.96, 133.50, 131.23, 131.01, 130.52, 130.46, 129.39, 129.17, 129.01, 129.00, 128.99, 128.71, 128.62, 128.53, 128.49, 128.26, 128.23, 101.77, 101.61, 78.84, 78.59, 76.6, 75.1, 74.79, 73.99, 73.44, 73.36, 69.4, 66.2, 64.3, 63.7, 41.3, 30.20, 29.71, 23.7 ppm.

ESI-HRMS: m/z $[M + H]^+$ calcd. for $C_{51}H_{56}NO_{13}$: 890.3746; found 890.3762.

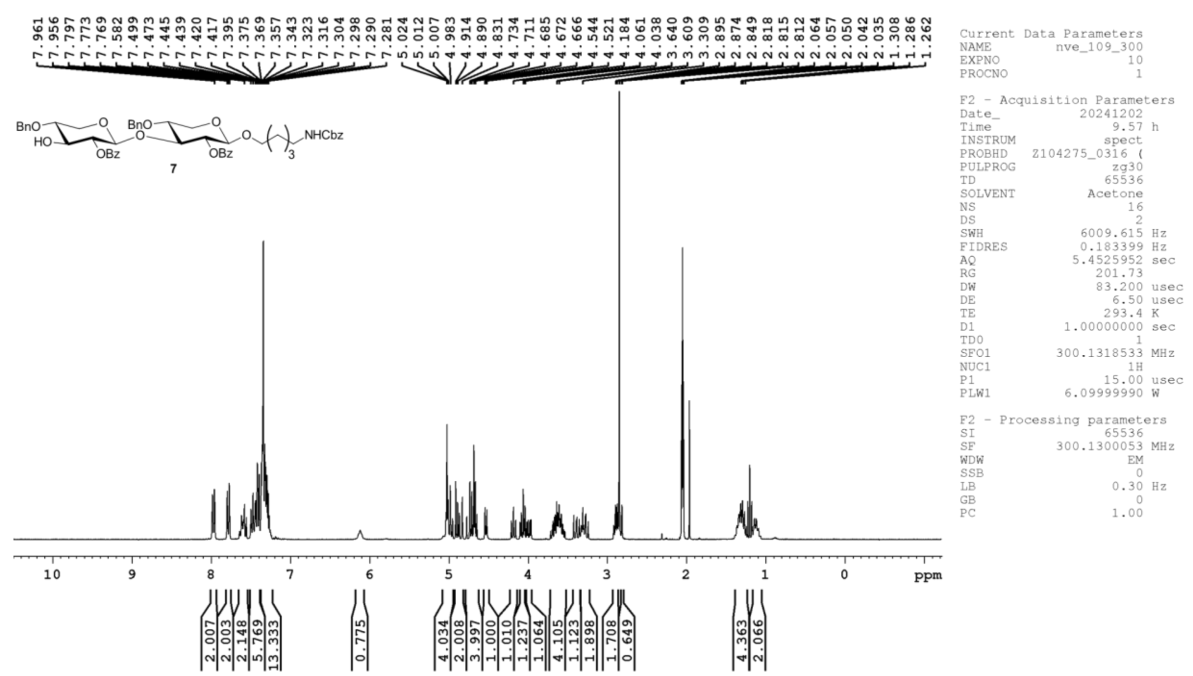

^{1}H NMR spectrum of compound **7** (300 MHz, $(CD_3)_2CO$)

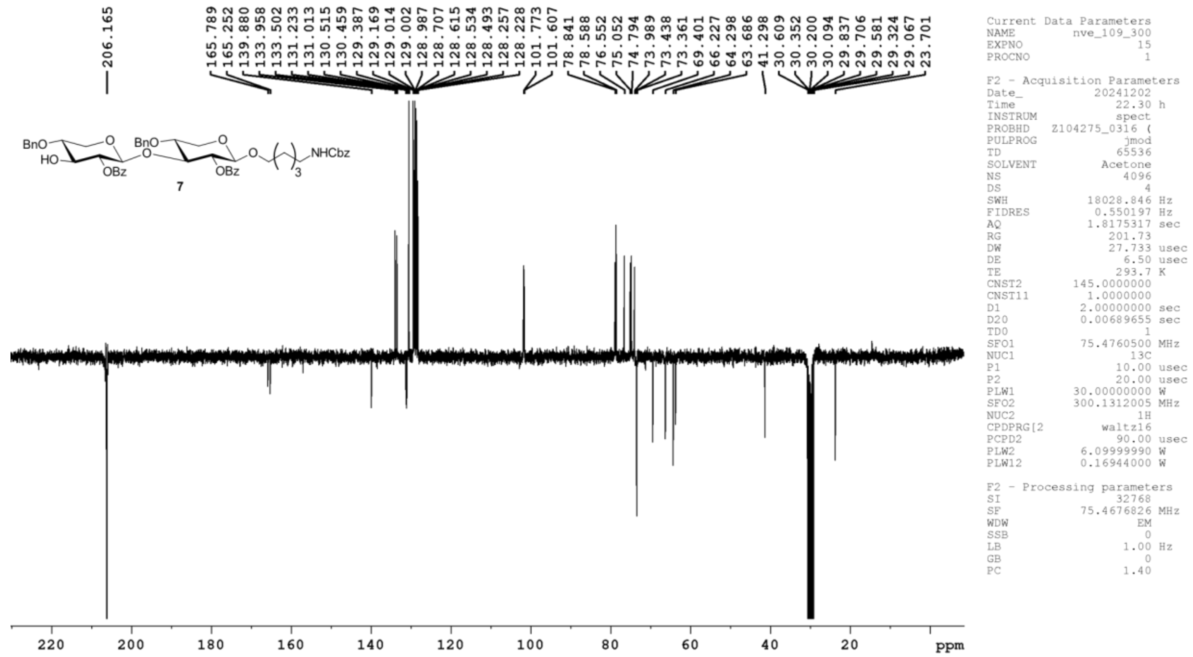

^{13}C APT NMR spectrum of compound **7** (76 MHz, $(CD_3)_2CO$)

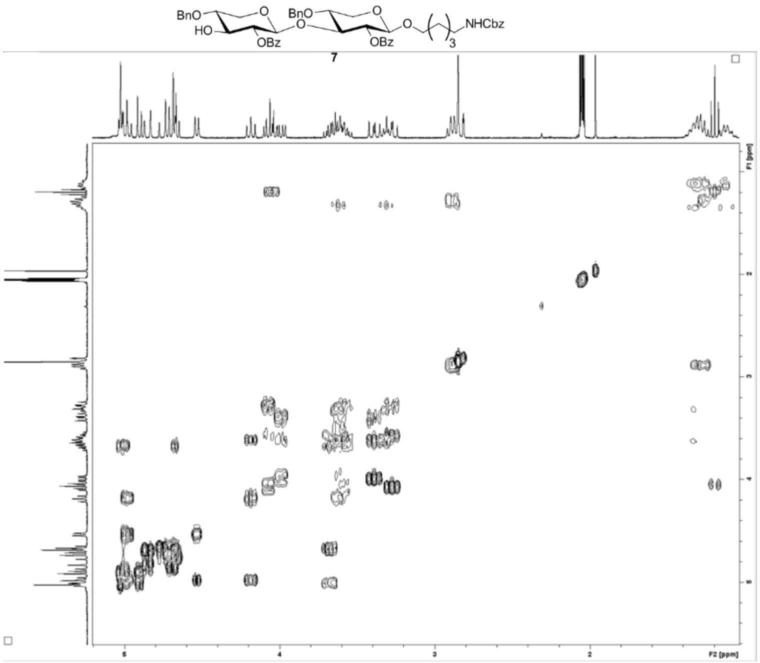

^{1}H-^{1}H COSY NMR spectrum of compound **7** (300 MHz, $(CD_3)_2CO$)

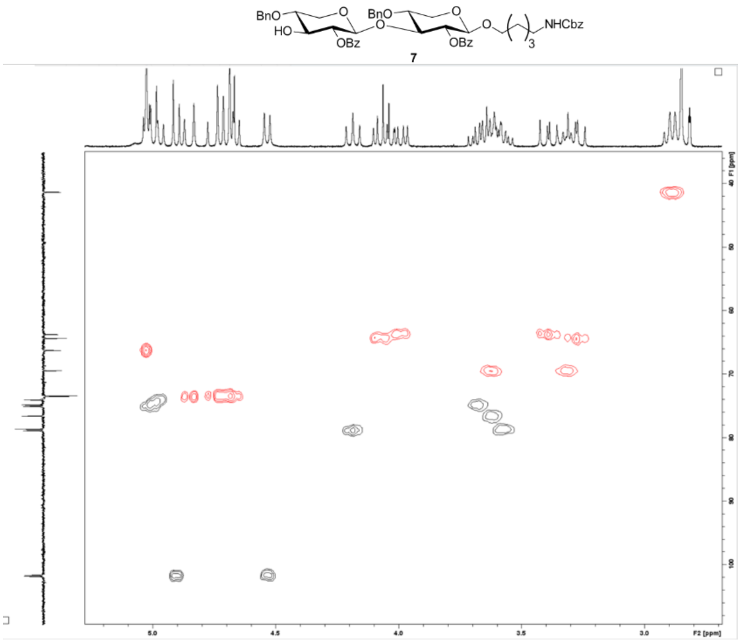

^{1}H-^{13}C HSQC NMR spectrum of compound **7** (300/76 MHz, $(CD_3)_2CO$)

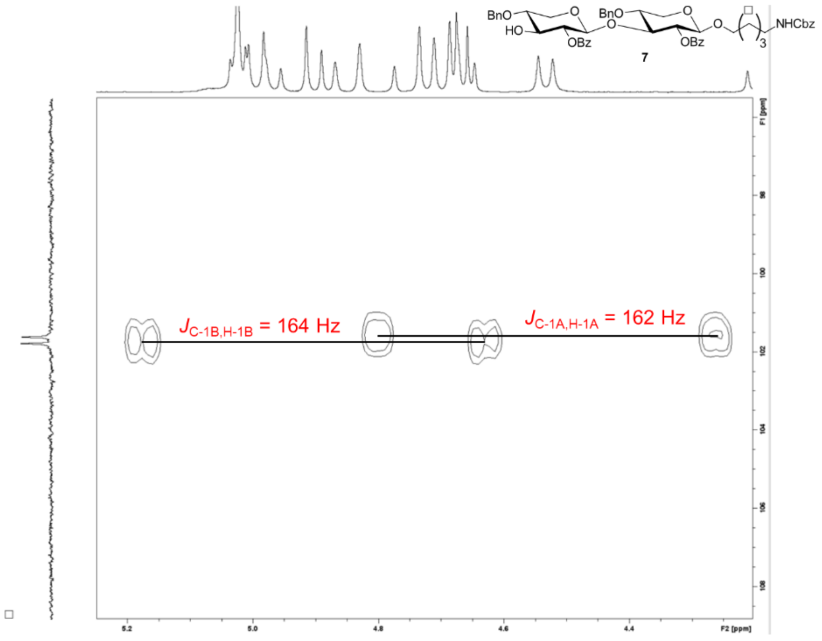

^{1}H-^{13}C CLIP HSQC NMR spectrum of compound **7** (300/76 MHz, $(CD_3)_2CO$)

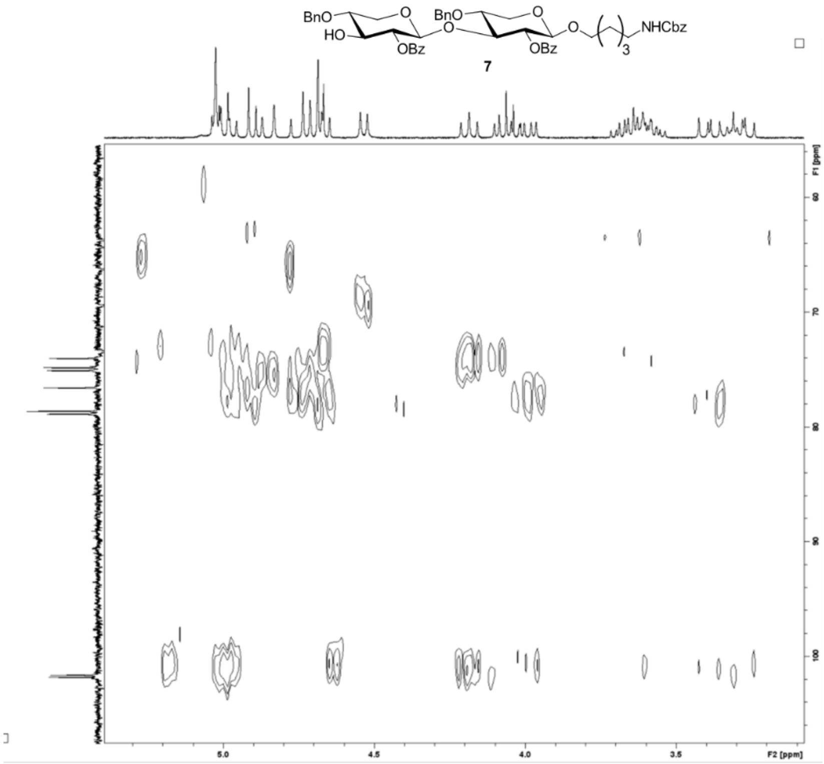

^{1}H-^{13}C HMBC NMR spectrum of compound **7** (300/76 MHz, $(CD_3)_2CO$)

AGA of Bn-protected β3-xylan tetrasaccharide 8

Benzyloxycarbonylaminopentyl 2-*O*-benzoyl-4-*O*-benzyl-β-D-xylopyranosyl-(1→3)-2-*O*-benzoyl-4-*O*-benzyl-β-D-xylopyranosyl-(1→3)-2-*O*-benzoyl-4-*O*-benzyl-β-D-xylopyranosyl-(1→3)-2-*O*-benzoyl-4-*O*-benzyl-β-D-xylopyranoside (**8**)

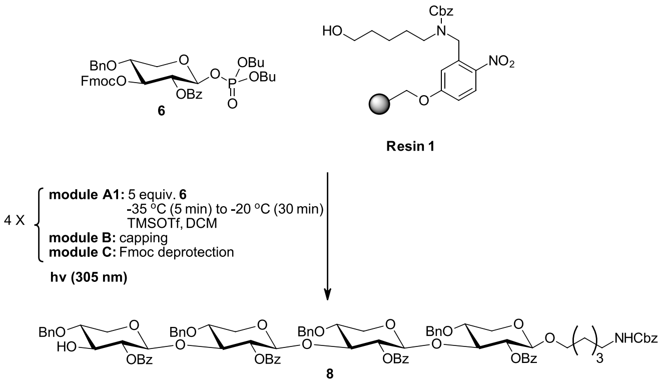

Experimental procedure: Linker-functionalized resin **1** (38.0 mg, 12.5 μmol) was placed in the synthesizer and synthesizer modules were applied as follows: 4 × [module A1 (BB **6**; total amount used = 190 mg, 0.25 mmol, 20 equiv.) at –35 ºC (5 min) to –20 ºC (30 min), module B, and module C]. Cleavage from the resin using UV irradiation at 305 nm in a continuous flow photoreactor afforded the crude product. Purification of the crude by normal phase HPLC using a preparative YMC-Small column (EtOAc/hexanes = 1/2.3 to 1/1, v/v) gave protected β3-xylan tetrasaccharide **8** (9.5 mg, 49% yield over 9 steps) as a glassy solid.

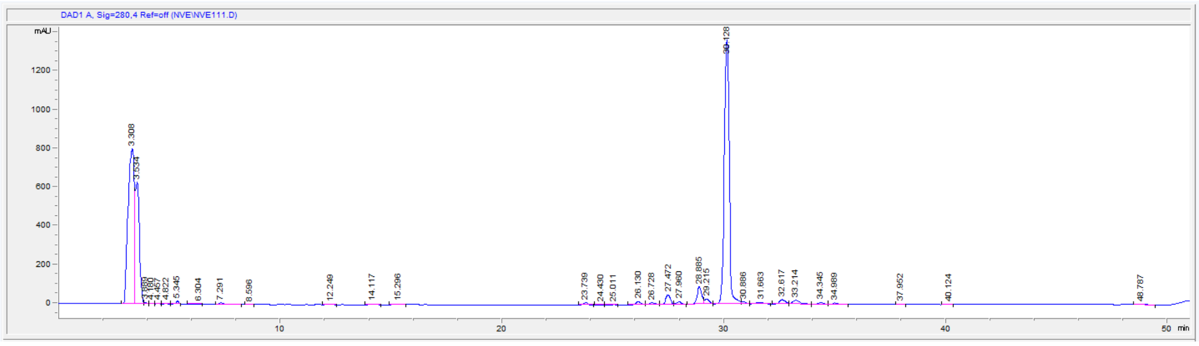

Analytical HPLC of the crude reaction mixture was performed on a YMC-Small NP column using a gradient of EtOAc in hexanes (50 min, flow rate 1 mL/min).

^{1}H NMR (600 MHz, $(CD_3)_2CO$): δ 7.94-7.86 (m, 4H, Ar-H), 7.76-7.72 (m, 2H, Ar-H), 7.64-7.55 (m, 4H, Ar-H), 7.52-7.48 (m, 1H, Ar-H), 7.47-7.43 (m, 2H, Ar-H), 7.42-7.19 (m, 32H, Ar-H), 6.06 (br, 1H, N-H), 5.05-4.98 (m, 3H, CH_2-Cbz, H-2D), 4.87 (dd, J = 5.6, 5.6 Hz, 1H, H-2C), 4.84-4.74 (m, 6H, CH_2-Ph, H-2A, H-1D, H-1B, H-1C), 4.74-4.71 (m, 1H, H-2B), 4.70-4.66 (m, 2H, CH_2-Ph, O-H), 4.65-4.56 (m, 4H, CH_2-Ph), 4.49 (d, J = 11.6 Hz, 1H, CH_2-Ph), 4.45 (d, J = 7.0 Hz, 1H, H-1A), 4.08-3.96 (m, 4H, H-5D, H-3C, H-5C, H-3A), 3.92-3.86 (m, 2H, H-5A, H-5B), 3.82 (t, J = 7.0 Hz, 1H, H-3B), 3.74-3.69 (m, 1H, H-3D), 3.63-3.54 (m, 2H, OCH_2, H-4D), 3.51-3.47 (m, 1H, H-4C), 3.44-3.39 (m, 1H, H-4A), 3.33-3.23 (m, 4H, OCH_2, H-5A, H-5C, H-5D), 3.22-3.17 (m, 2H, H-4B, H-5B), 2.93-2.86 (m, 2H, CH_2-NHCbz), 1.38-1.25 (m, 4H, CH_2), 1.16-1.10 (m, 2H, CH_2) ppm.

^{13}C NMR (151 MHz, $(CD_3)_2CO$): δ 166.1, 165.26, 165.25, 165.10, 139.96, 139.94, 139.79, 139.70, 133.90, 133.82, 133.55, 131.34, 131.00, 130.66, 130.63, 130.62, 130.56, 130.41, 130.38, 129.29, 129.28, 129.22, 129.19, 129.05, 129.03, 128.96, 128.80, 128.73, 128.63, 128.59, 128.55, 128.53, 128.27, 128.22, 128.18, 101.65, 101.41, 100.76, 100.08, 78.67, 78.63, 76.74, 76.62, 76.34, 75.99, 75.68, 74.96, 74.94, 74.83, 74.11, 73.55, 73.49, 73.46, 73.09, 72.57, 72.33, 69.4, 66.3, 64.5, 63.8, 62.8, 61.8, 41.4, 29.8, 23.8 ppm.

ESI-HRMS: m/z $[M + H]^+$ calcd. for $C_{89}H_{92}NO_{23}$: 1542.6055; found 1542.6065.

NMR chemical shifts of selected ^{1}H and ^{13}C atoms in compound **8**:

xylose ring	proton	δ (ppm)	multiplicity	J (Hz)	carbon	δ (ppm)
A (reducing end)	H-1A	4.45	d	7.0	C-1A	101.65
	H-2A	4.80				
	H-3A	3.99			C-3A	78.63
	H-4A	3.44-3.39	m			
	H-5Aa	3.32				
	H-5Ab	3.92				
B	H-1B	4.78			C-1B	100.76
	H-2B	4.74-4.71	m			
	H-3B	3.82	t	7.0	C-3B	76.62
	H-4B	3.19				
	H-5Ba	3.18			C-5B	62.83
	H-5Bb	3.88				
C	H-1C	4.74			C-1C	100.08
	H-2C	4.87	dd	5.6, 5.6		
	H-3C	4.01			C-3C	75.99
	H-4C	3.51-3.47	m			
	H-5Ca	3.25			C-5C	64.5
	H-5Cb	4.01				
D (non-reducing end)	H-1D	4.82			C-1D	101.41
	H-2D	5.0				
	H-3D	3.74-3.69	m			
	H-4D	3.54				
	H-5Da	3.22				
	H-5Db	4.02				

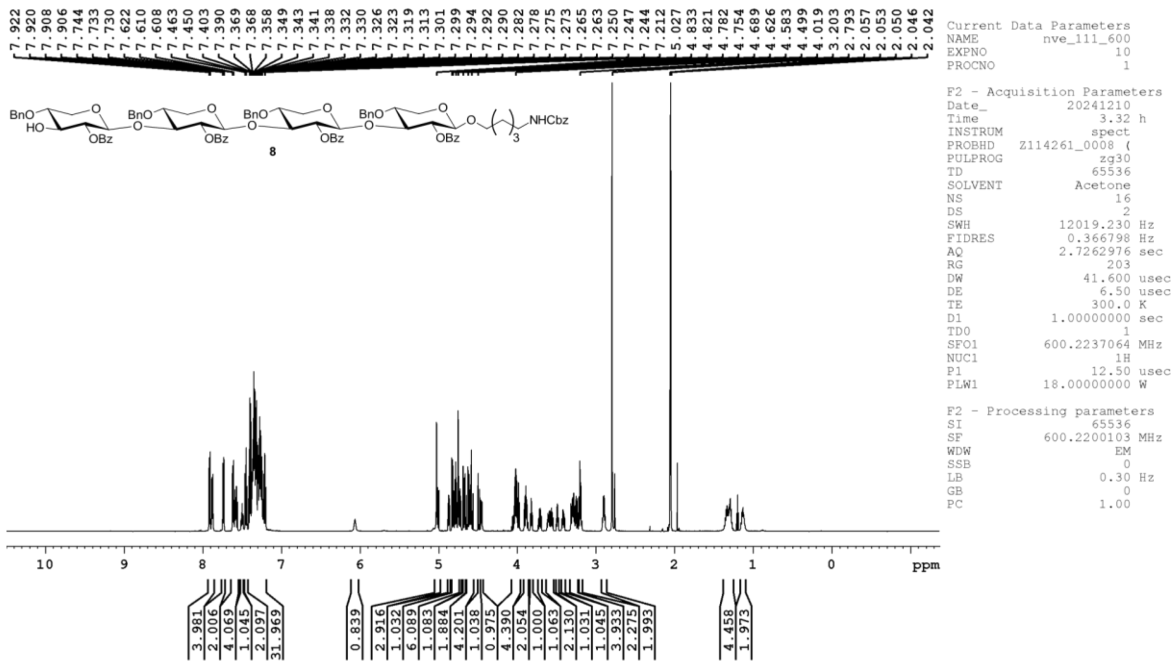

^{1}H NMR spectrum of compound **8** (600 MHz, $(CD_3)_2CO$)

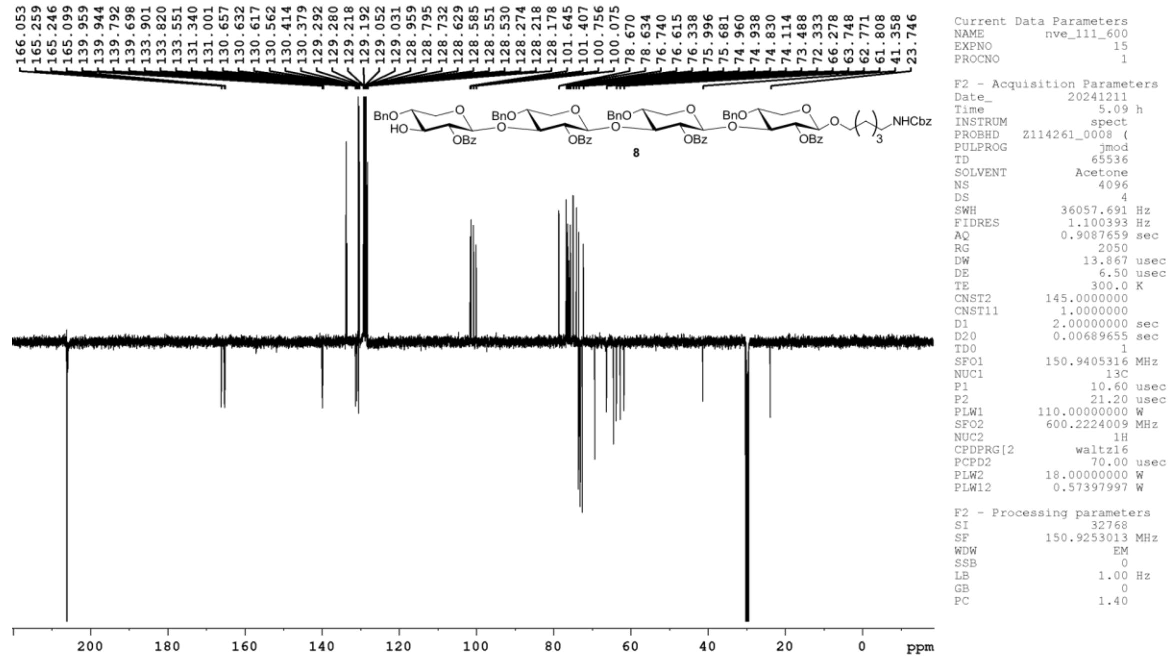

^{13}C APT NMR spectrum of compound **8** (151 MHz, $(CD_3)_2CO$)

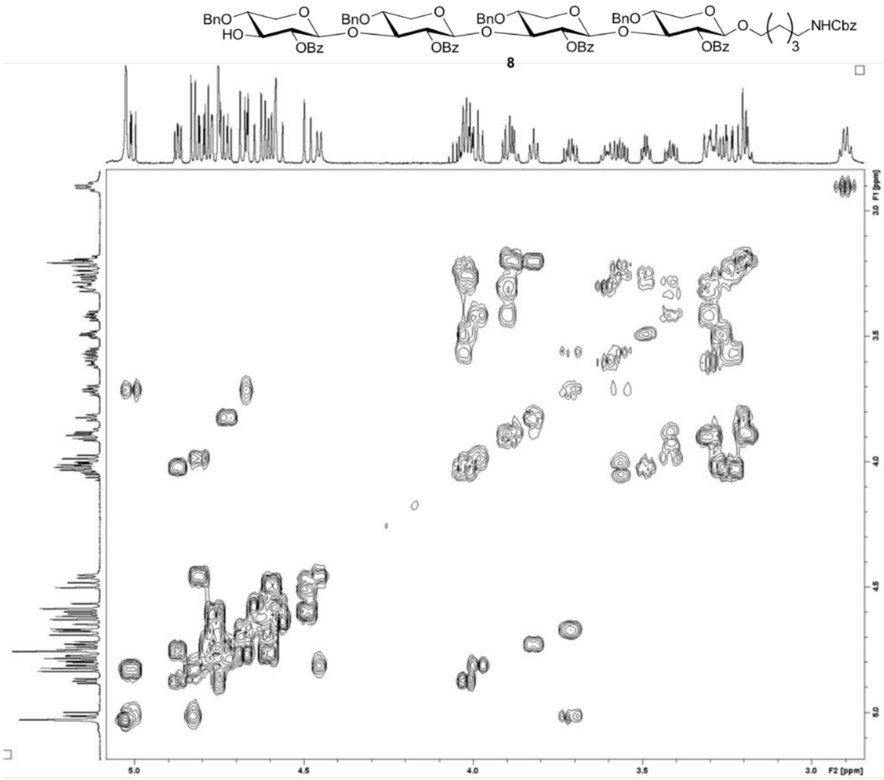

^{1}H-^{1}H COSY NMR spectrum of compound **8** (600 MHz, $(CD_3)_2CO$)

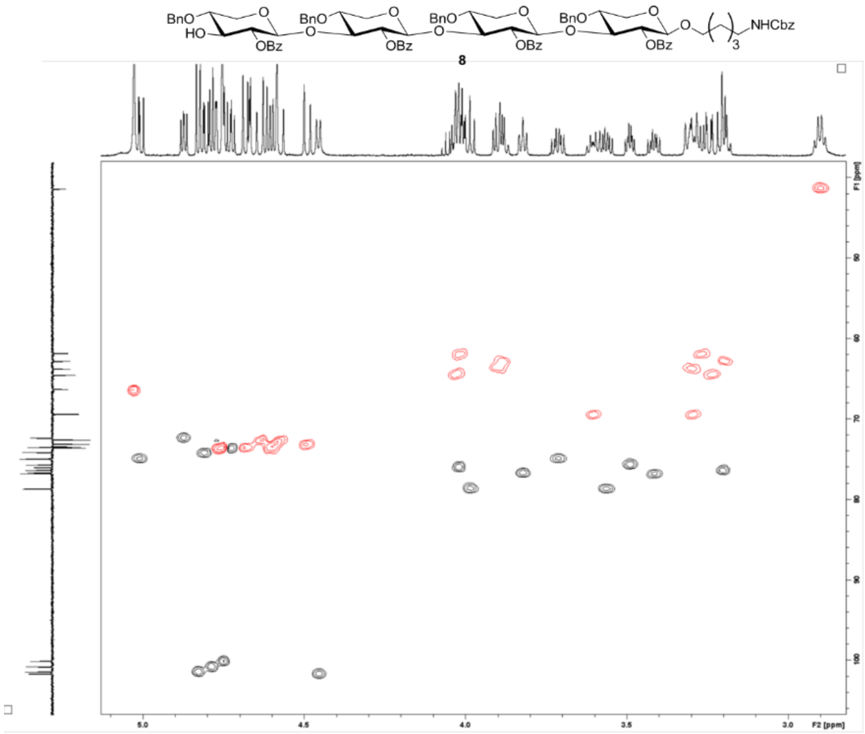

^{1}H-^{13}C HSQC NMR spectrum of compound **8** (600/151 MHz, $(CD_3)_2CO$)

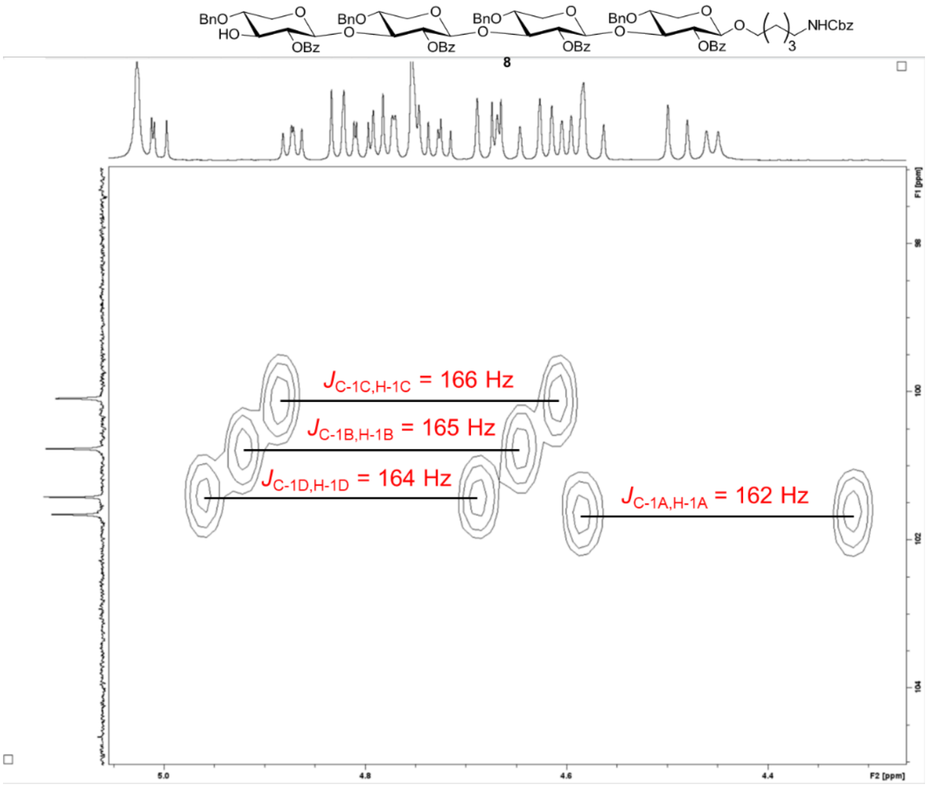

^{1}H-^{13}C CLIP-HSQC NMR spectrum of compound **8** (600/151 MHz, $(CD_3)_2CO$)

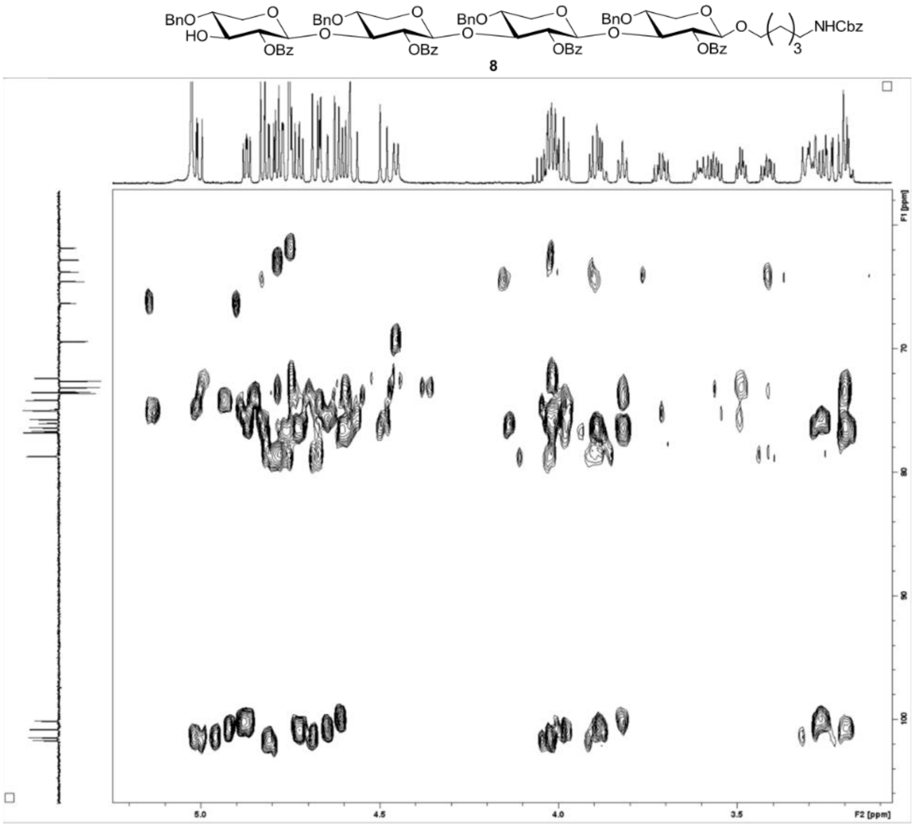

^{1}H-^{13}C HMBC NMR spectrum of compound **8** (600/151 MHz, $(CD_3)_2CO$)

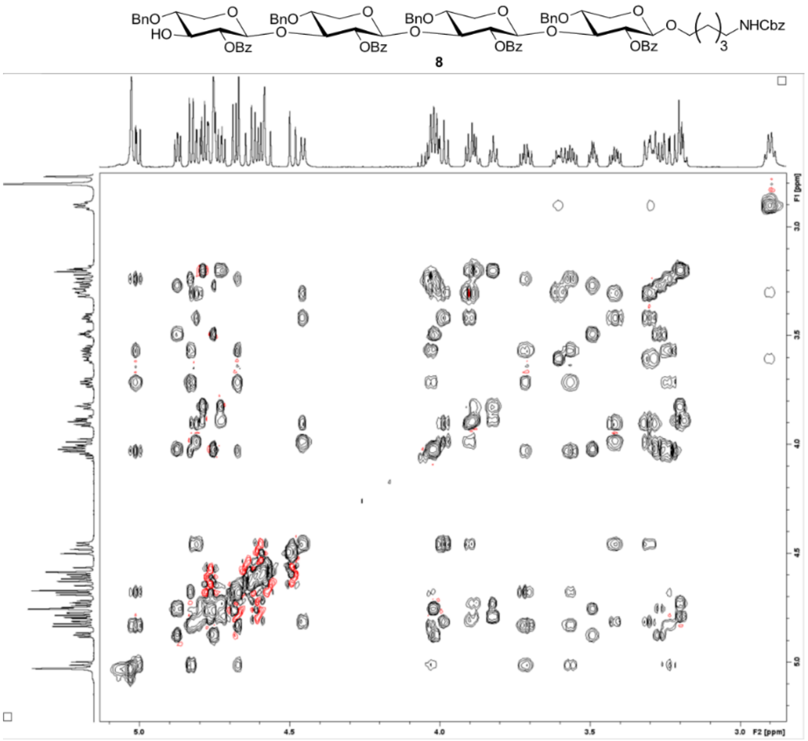

^{1}H-^{1}H TOCSY NMR spectrum of compound **8** (600 MHz, $(CD_3)_2CO$)

AGA of Bn-protected β3-xylan hexasaccharide 9

Benzyloxycarbonylaminopentyl 2-*O*-benzoyl-4-*O*-benzyl-β-D-xylopyranosyl-(1→3)-2-*O*-benzoyl-4-*O*-benzyl-β-D-xylopyranosyl-(1→3)-2-*O*-benzoyl-4-*O*-benzyl-β-D-xylopyranosyl-(1→3)-2-*O*-benzoyl-4-*O*-benzyl-β-D-xylopyranosyl-(1→3)-2-*O*-benzoyl-4-*O*-benzyl-β-D-xylopyranosyl-(1→3)-2-*O*-benzoyl-4-*O*-benzyl-β-D-xylopyranoside (**9**)

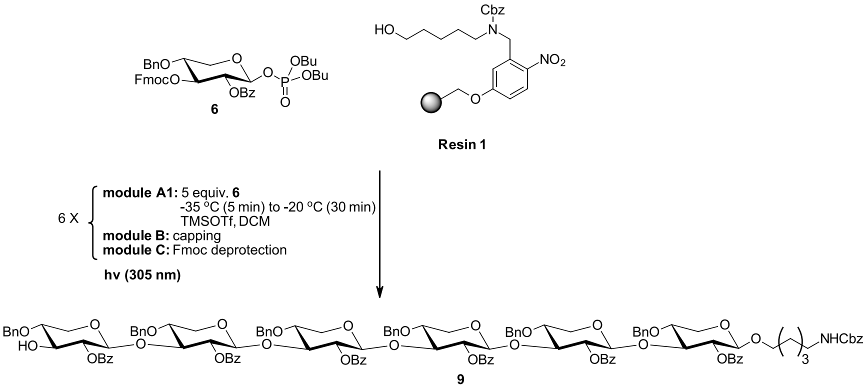

Experimental procedure: Linker-functionalized resin **1** (38.0 mg, 12.5 μmol) was placed in the synthesizer and synthesizer modules were applied as follows: 6 × [module A1 (BB **6**; total amount used = 285 mg, 0.375 mmol, 30 equiv.) at –35 °C (5 min) to –20 °C (30 min), module B, and module C]. Cleavage from the resin using UV irradiation at 305 nm in a continuous flow photoreactor afforded the crude product. Purification of the crude by normal phase HPLC using a preparative YMC-Small column (EtOAc/hexanes = 1/2.3 to 1/1, v/v) gave protected β3-xylan hexasaccharide **9** (11.4 mg, 42% yield over 13 steps) as a glassy solid.

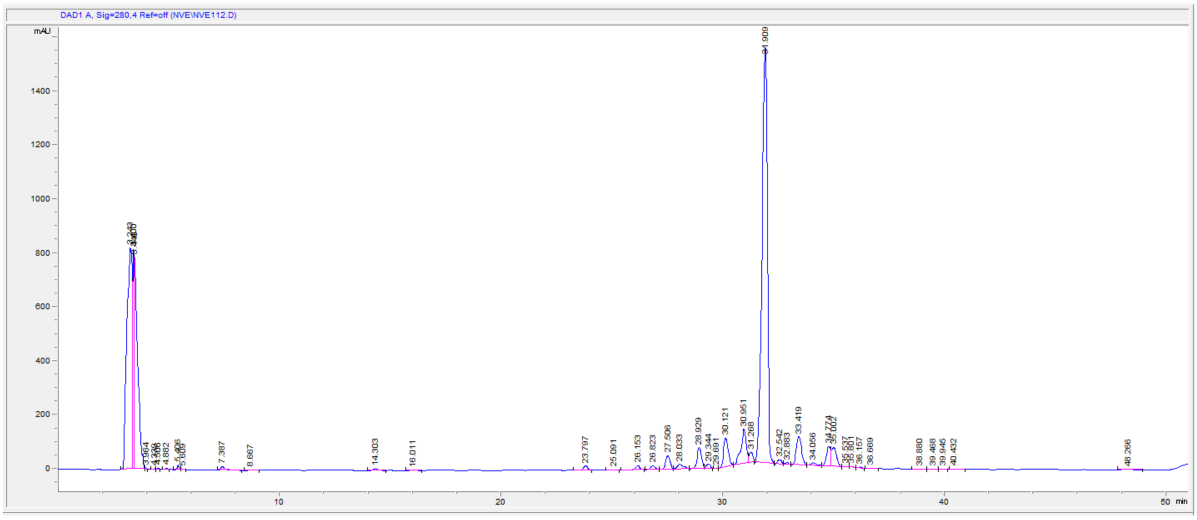

Analytical HPLC of the crude reaction mixture was performed on a YMC-Small NP column using a gradient of EtOAc in hexanes (50 min, flow rate 1 mL/min).

^{1}H NMR (600 MHz, $(CD_3)_2CO$): 7.91-7.88 (m, 2H, Ar-H), 7.88-7.85 (m, 2H, Ar-H), 7.74-7.68 (m, 6H, Ar-H), 7.61-7.58 (m, 2H, Ar-H), 7.55-7.45 (m, 4H, Ar-H), 7.43-7.38 (m, 5H, Ar-H), 7.38-7.23 (m, 42H, Ar-H), 7.23-7.19 (m, 2H, Ar-H), 6.06 (br, 1H, N-H), 5.05-4.98 (m, 3H, CH_2Cbz, H-2F), 4.88 (dd, *J* = 5.6, 5.6 Hz, 1H, H-2E), 4.83 (d, *J* = 7.4 Hz, 1H, H-1F), 4.79-4.74 (m, 5H, H-1E, H-1B, H-2A, CH_2), 4.73-4.68 (m, 6H, H-1C, H-1D, CH_2, H-2B, H-2C, H-2D), 4.67-4.63 (m, 2H, CH_2, O-H), 4.61-4.57 (m, 5H, all CH_2), 4.52-4.48 (m, 3H, all CH_2), 4.43 (d, *J* = 7.0 Hz, 1H, H-1A), 4.05-4.00 (m, 3H, H-3E, H-5E, H-5F), 3.95 (t, *J* = 8.2 Hz, 1H, H-3A), 3.92-3.88 (m, 2H), 3.88-3.79 (m, 5H, H-5A), 3.73-3.68 (m, 1H, H-3F), 3.61-3.54 (m, 2H, OCH_2, H-4F), 3.49 (ddd, *J* = 4.0, 6.2, 6.2 Hz, 1H, H-4E),

3.37-3.32 (m, 1H, H-4A), 3.31-3.26 (m, 4H, H-5E, H-5A, OCH_2), 3.26-3.19 (m, 4H, H-5F), 3.19-3.12 (m, 2H), 2.93-2.87 (m, 2H, $C\mathit{H}_2$-NHCbz), 1.37-1.29 (m, 4H, CH_2), 1.16-1.09 (m, 2H, CH_2) ppm.

^{13}C NMR (151 MHz, $(CD_3)_2CO$): δ 166.0, 165.31, 165.29, 165.22, 165.08, 139.96, 139.80, 139.75, 139.71, 133.85, 133.81, 133.76, 133.71, 131.32, 130.98, 130.66, 130.62, 130.57, 130.53, 130.47, 130.36, 129.21, 129.17, 129.05, 128.99, 128.97, 128.83, 128.77, 128.72, 128.67, 128.63, 128.59, 128.54, 128.29, 128.25, 128.22, 101.61, 101.38, 100.57, 100.43, 100.25, 100.08, 78.67, 78.51, 76.84, 76.72, 76.41, 76.35, 76.32, 76.13, 76.01, 75.98, 75.71, 74.95, 74.92, 74.12, 73.53, 73.45, 73.44, 73.17, 73.05, 72.83, 72.79, 72.71, 72.56, 72.38, 69.3, 66.3, 64.5, 63.74, 62.97, 62.43, 62.34, 61.8, 41.4, 29.8, 23.8 ppm.

ESI-HRMS: m/z $[M + HCOO]^-$ calcd. for $C_{128}H_{128}NO_{35}$: 2238.8272; found 2238.8255.

NMR chemical shifts of selected ^{1}H and ^{13}C atoms in compound **9**:

**xylose ring**	**proton**	***δ* (ppm)**	**multiplicity**	***J* (Hz)**	**carbon**	***δ* (ppm)**
A (reducing end)	H-1A	4.43	d	7.0	C-1A	101.61
	H-2A	4.76				
	H-3A	3.95	t	8.2	C-3A	78.51
	H-4A	3.37-3.32	m			
	H-5Aa	3.27				
	H-5Ab	3.85				
B	H-1B	4.79-4.74	m			
	H-2B	4.73-4.68	m			
C	H-1C	4.73-4.68	m			
	H-2C	4.73-4.68	m			
D	H-1D	4.73-4.68	m			
	H-2D	4.73-4.68	m			
E	H-1E	4.79-4.74	m			
	H-2E	4.88	dd	5.6, 5.6		
	H-3E	4.05-4.00	m			
	H-4E	3.49	ddd	4.0, 6.2, 6.2		
	H-5Ea	3.31-3.26	m			
	H-5Eb	4.05-4.00	m			
F (non-reducing end)	H-1F	4.83	d	7.4	C-1F	101.38
	H-2F	5.00				
	H-3F	3.73-3.68	m			
	H-4F	3.56				
	H-5Fa	3.22				
	H-5Fb	4.02				

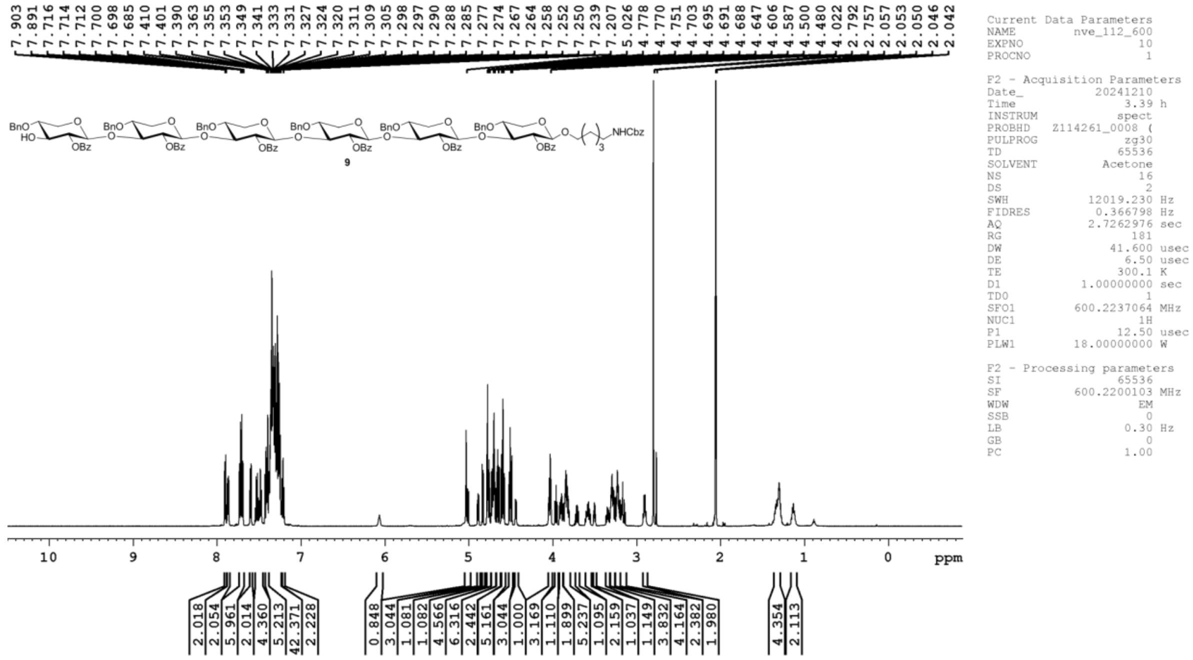

^{1}H NMR spectrum of compound **9** (600 MHz, $(CD_3)_2CO$)

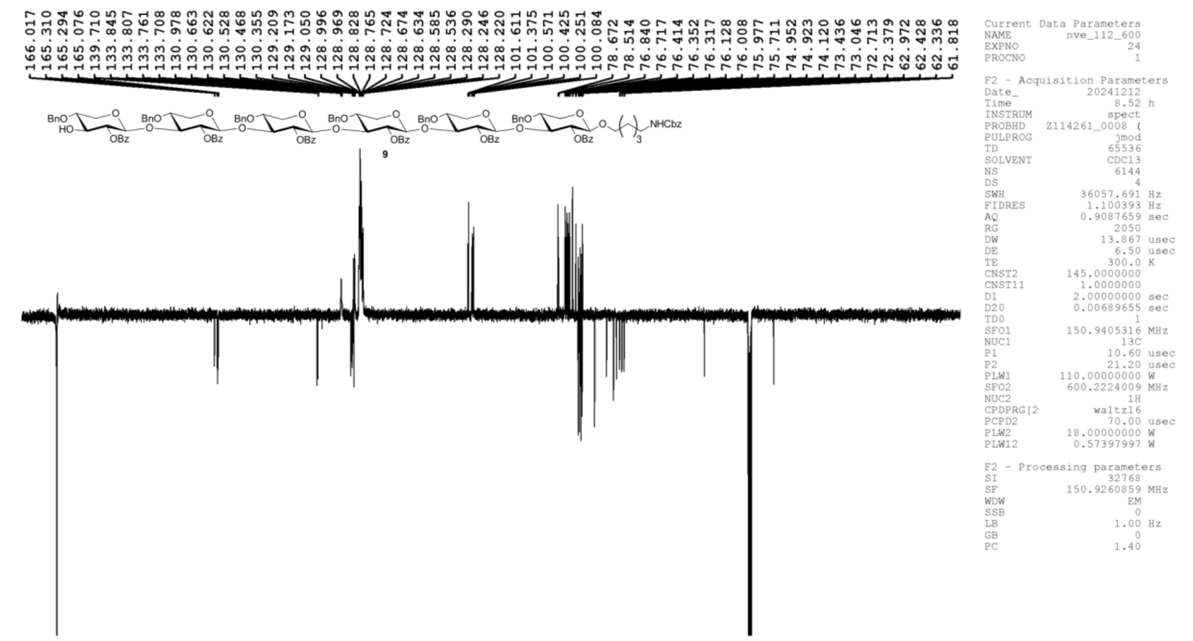

^{13}C APT NMR spectrum of compound **9** (151 MHz, $(CD_3)_2CO$)

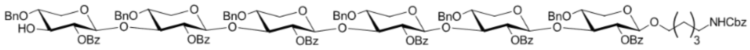

9

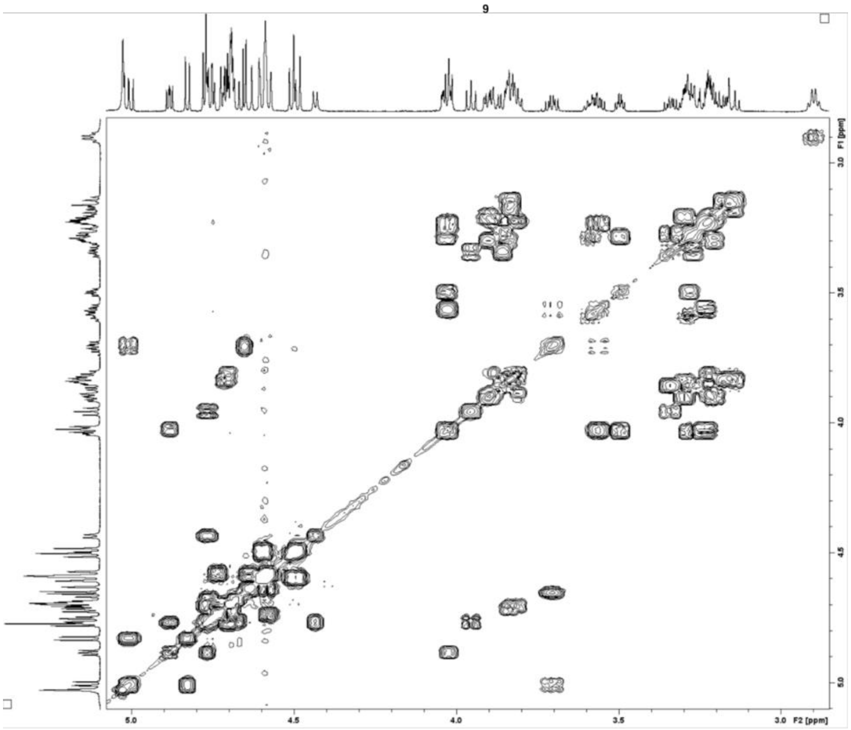

^{1}H-^{1}H COSY NMR spectrum of compound **9** (600 MHz, $(CD_3)_2CO$)

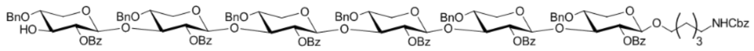

9

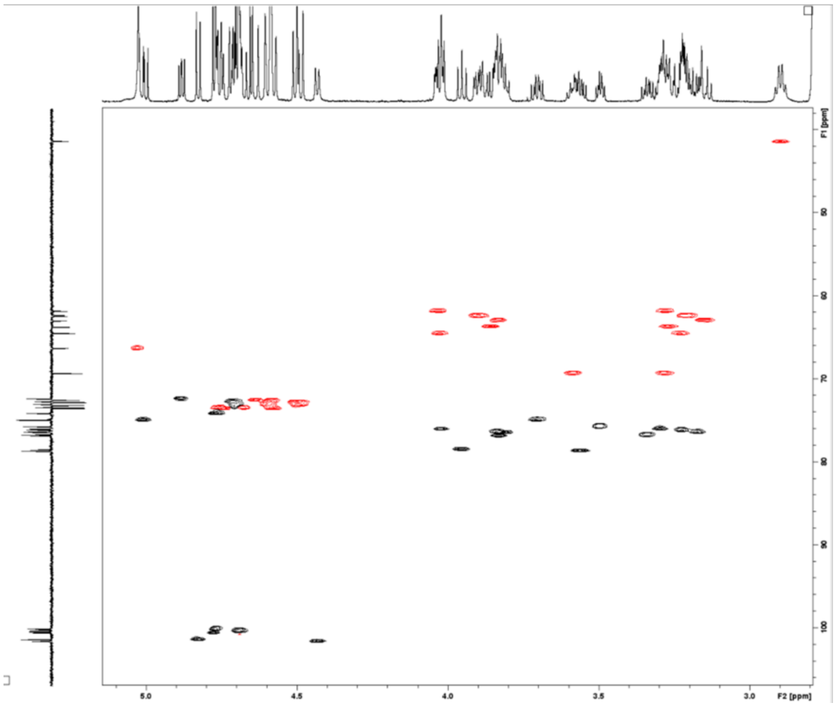

^{1}H-^{13}C HSQC NMR spectrum of compound **9** (600/151 MHz, $(CD_3)_2CO$)

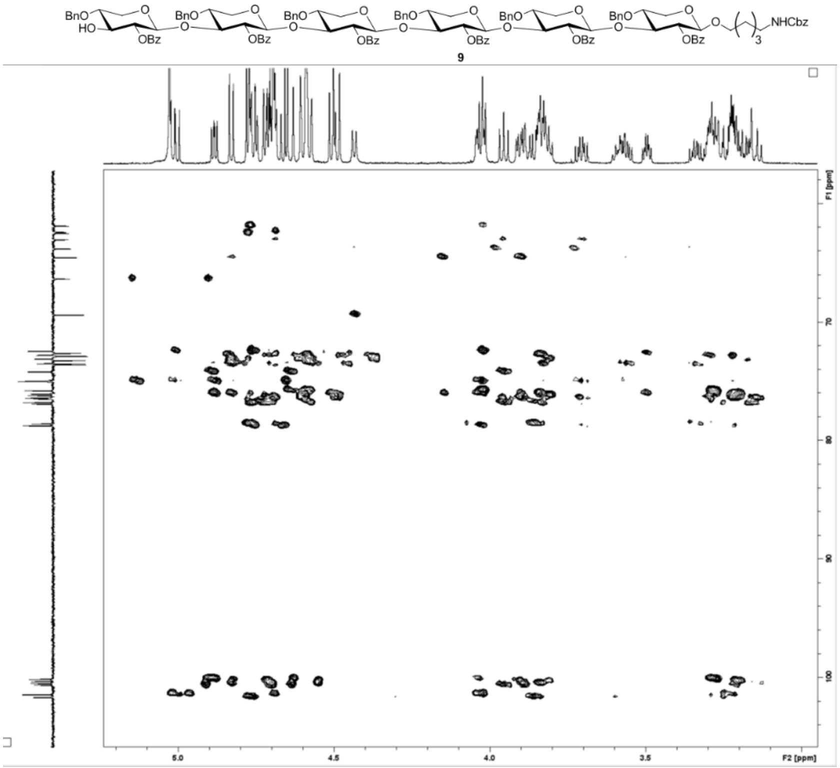

^{1}H-^{13}C HMBC NMR spectrum of compound **9** (600/151 MHz, $(CD_3)_2CO$)

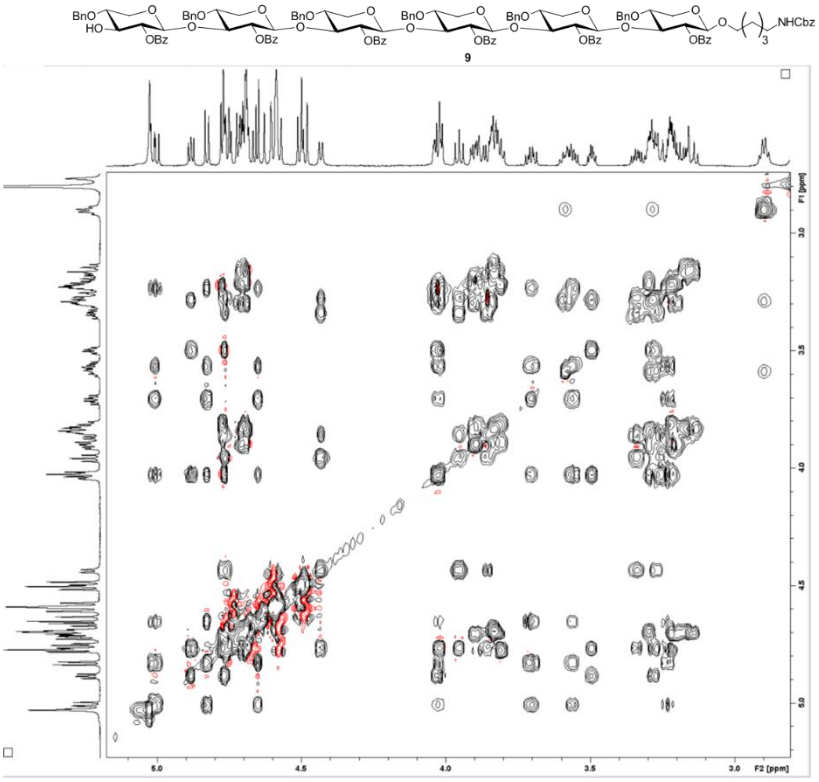

^{1}H-^{1}H TOCSY NMR spectrum of compound **9** (600 MHz, $(CD_3)_2CO$)

Aminopentyl β-D-xylopyranosyl-(1→3)-β-D-xylopyranosyl-(1→3)-β-D-xylopyranosyl-(1→3)-β-D-xylopyranosyl-(1→3)-β-D-xylopyranosyl-(1→3)-β-D-xylopyranoside (13)

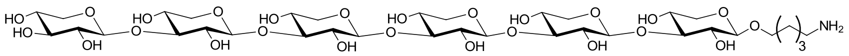

13

Experimental procedure: To a solution of protected hexasaccharide **9** (11.4 mg, 5.2 µmol) in THF (156 µL, 33 mM), a solution of NaOMe in MeOH (0.5 M, 156 µL, 78 µmol) was added at rt, and the reaction mixture was allowed to stir overnight. After confirmation of reaction completion (22 h) by TLC (R_f: 0.13 (toluene/ACN = 2/1, v/v)), the reaction mixture was neutralized by the addition of IR-120 H^+ resin while slowly stirring the mixture. Then, the reaction mixture was filtered, and the filtrate was concentrated under reduced pressure to yield a partially-deprotected glassy crude, which was kept under high vacuum until usage in the next step without any further purification. To a solution of partially deprotected crude (5.2 µmol) in *t*-BuOH (1 mL), H_2O (0.35 mL), and AcOH (3 drops), unreduced 10% Pd/C (9.0 mg) was added, and the reaction mixture was stirred in the H_2 reactor under a pressure of 8 bar H_2. After 48 h, the reaction progress was checked by MALDI/TOF-MS analysis, and more unreduced 10% Pd/C (9.0 mg) was added. The reaction mixture was allowed to stir for two more days at 8 bar H_2 at rt. Then, the reaction mixture was filtered using a PTFE syringe filter (0.45 µm) and concentrated under reduced pressure to yield a crude product, which was purified using HILIC-HPLC column chromatography (13 mM NH_4OAc/ACN = 1/4 to 1/1, v/v). The purified product was lyophilized to give β3-xylan hexasaccharide **13** (4.12 mg, 74% yield over 2 steps) as a white amorphous solid.

^{1}H NMR (600 MHz, D_2O): *δ* 4.72-4.64 (m, 5H, H-1B, H-1C, H-1D, H-1E, H-1F), 4.41 (d, *J* = 7.9 Hz, 1H, H-1A), 4.03-3.93 (m, 6H, H-5A, H-5B, H-5C, H-5D, H-5E, H-5F), 3.90-3.83 (m, 1H, OCH_2), 3.73-3.59 (m, 13H, OCH_2, H-3A), 3.55-3.49 (m, 4H), 3.47-3.40 (m, 2H, H-2A), 3.35-3.26 (m, 7H, H-5A, H-5B, H-5C, H-5D, H-5E, H-5F), 3.00 (t, *J* = 7.5 Hz, 2H, CH_2-NH_2), 1.71-1.61 (m, 4H, CH_2), 1.47-1.40 (m, 2H, CH_2) ppm.

^{13}C NMR (151 MHz, D_2O): *δ* 104.04, 103.83, 103.79, 103.30, 84.39, 84.25, 84.14, 84.13, 76.2, 73.97, 73.70, 73.65, 73.32, 70.76, 69.80, 68.28, 68.26, 65.76, 65.43, 65.41, 65.40, 40.0, 28.8, 27.0, 23.9, 22.7 ppm.

ESI-HRMS: m/z $[M + H]^+$ calcd. for $C_{35}H_{62}NO_{25}$: 896.3605; found 896.3602.

NMR chemical shifts of selected ^{1}H and ^{13}C atoms in compound **13**:

xylose ring	proton	*δ* (ppm)	multiplicity	*J* (Hz)	carbon	*δ* (ppm)
A (reducing end)	H-1A	4.41	d	7.9	C-1A	103.30
	H-2A	3.47-3.40	m			
	H-3A	3.73-3.59	m			
	H-5Aa	3.35-3.26	m			
	H-5Ab	4.03-3.93	m			
B	H-1B	4.72-4.64	m			
	H-5Ba	3.35-3.26	m			
	H-5Bb	4.03-3.93	m			

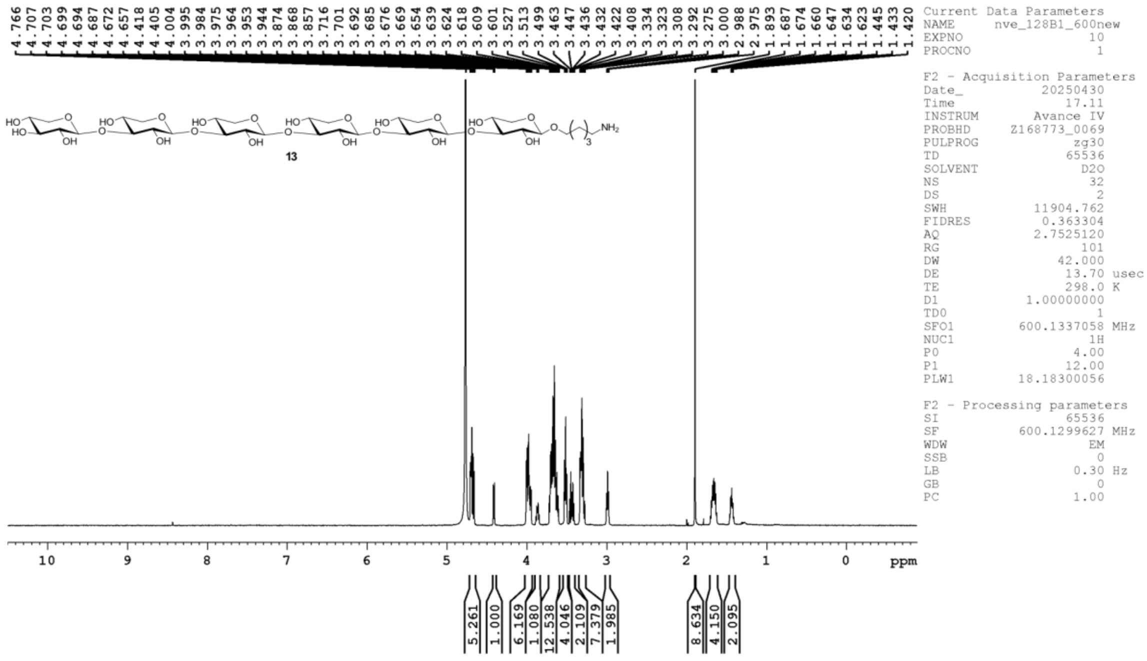

^{1}H NMR spectrum of compound **13** (600 MHz, D_2O)

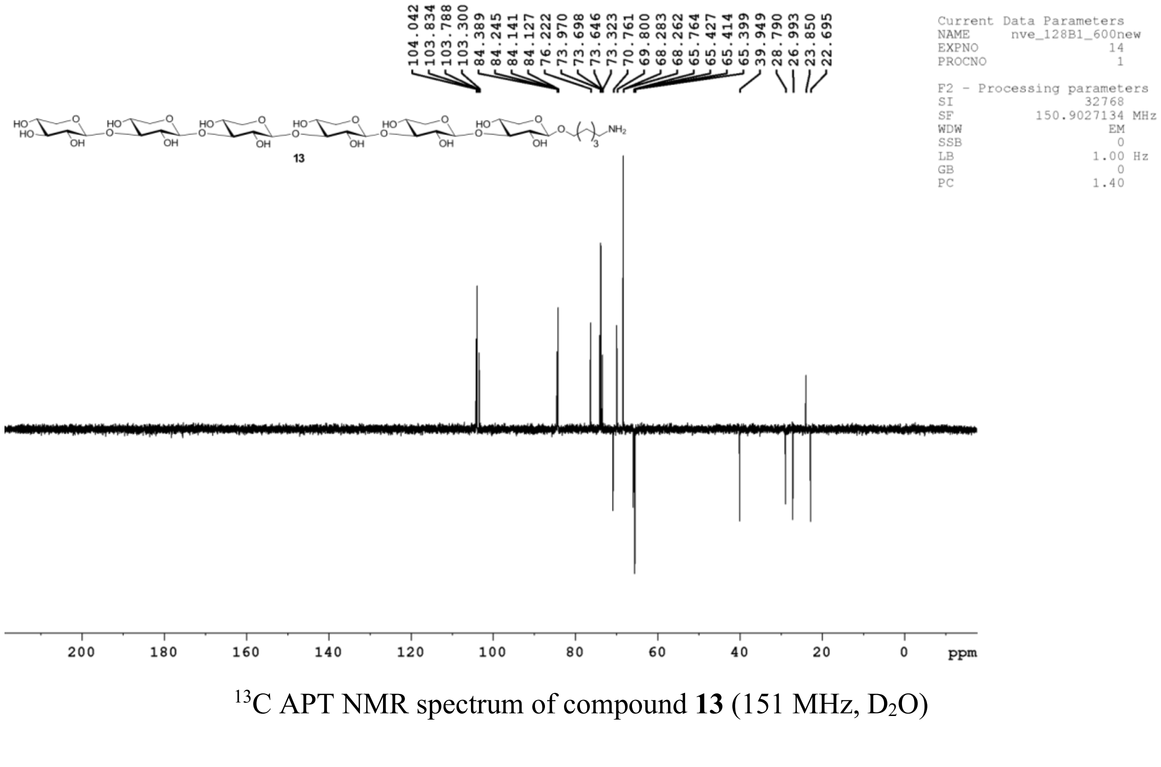

^{13}C APT NMR spectrum of compound **13** (151 MHz, D_2O)

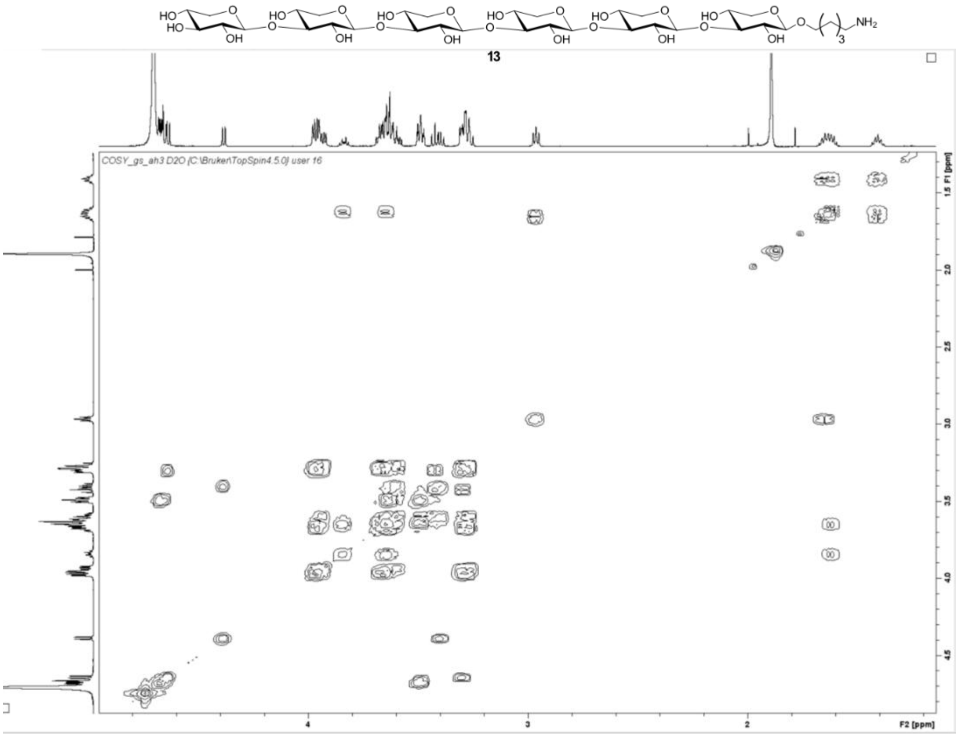

^{1}H-^{1}H COSY NMR spectrum of compound **13** (600 MHz, D_2O)

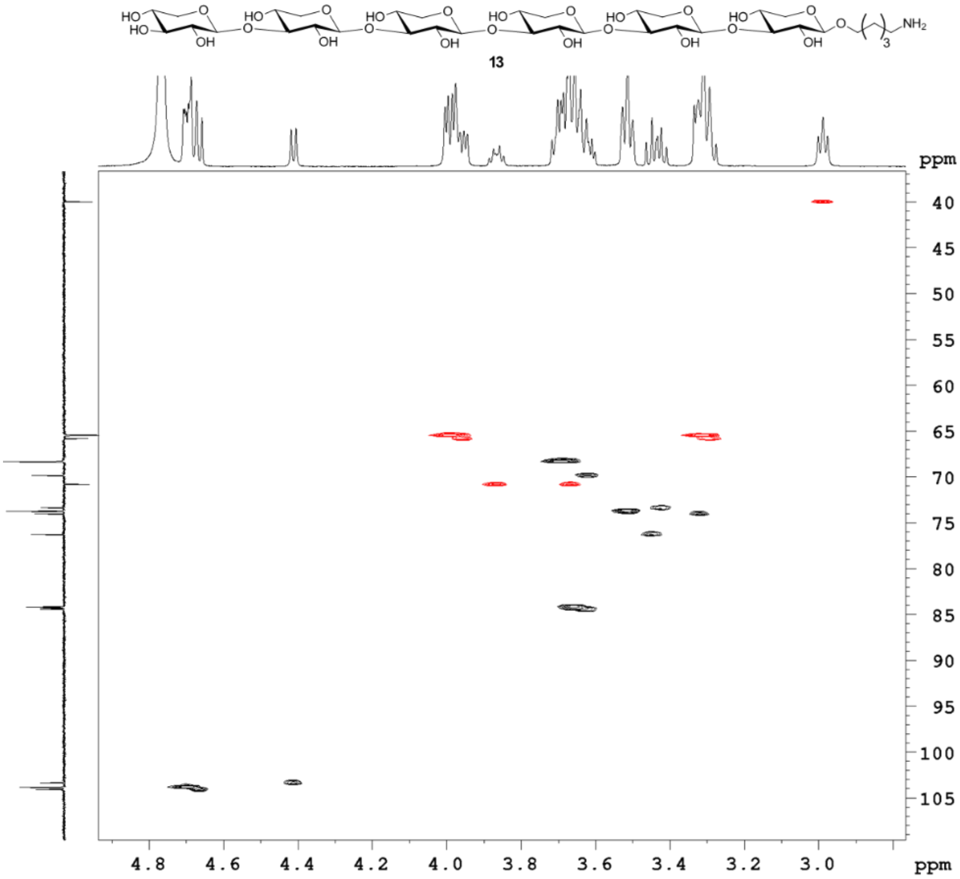

^{1}H-^{13}C HSQC NMR spectrum of compound **13** (600/151 MHz, D_2O)

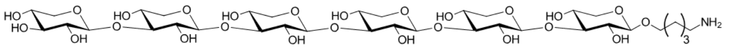

13

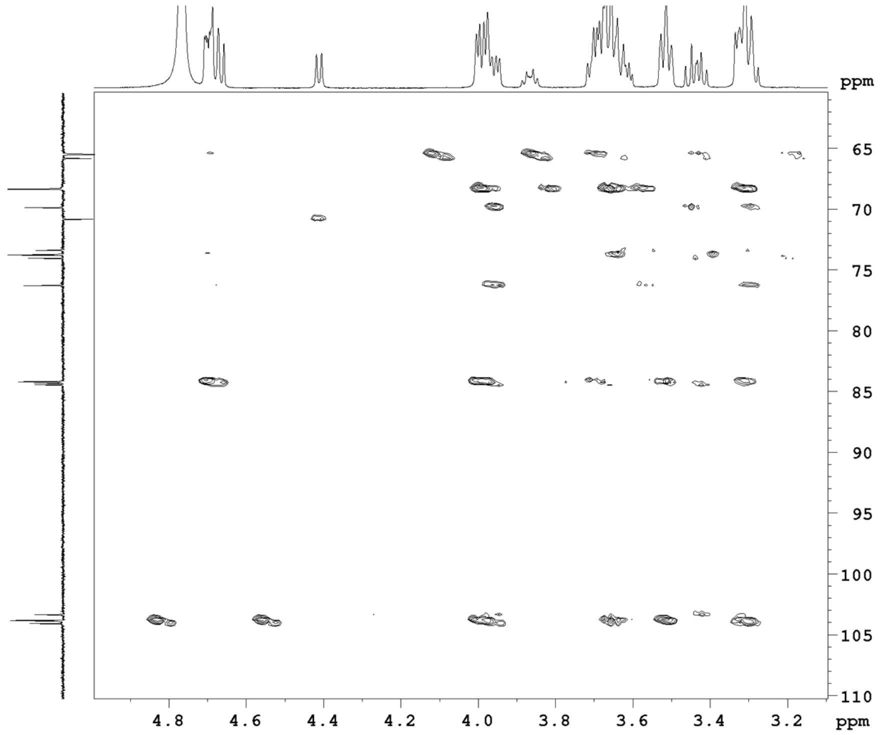

^{1}H-^{13}C HMBC NMR spectrum of compound **13** (600/151 MHz, D_2O)

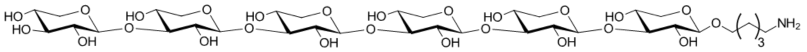

13

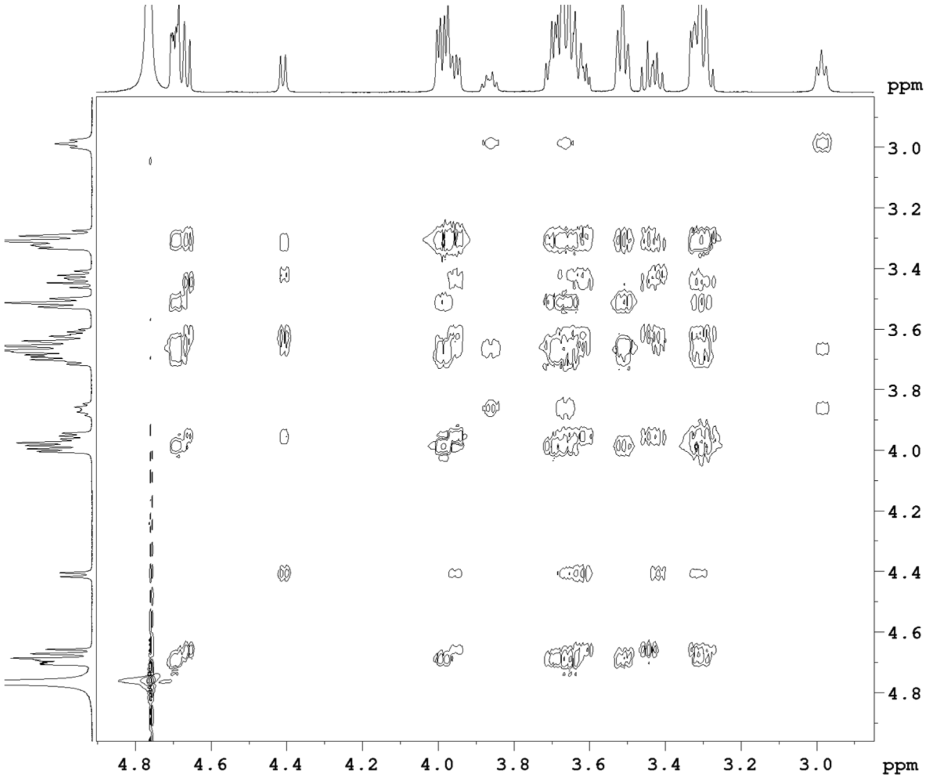

^{1}H-^{1}H TOCSY NMR spectrum of compound **13** (600 MHz, D_2O)

AGA of Bn-protected β3-xylan decasaccharide 10

Benzyloxycarbonylaminopentyl 2-*O*-benzoyl-4-*O*-benzyl-β-D-xylopyranosyl-(1→3)-2-*O*-benzoyl-4-*O*-benzyl-β-D-xylopyranosyl-(1→3)-2-*O*-benzoyl-4-*O*-benzyl-β-D-xylopyranosyl-(1→3)-2-*O*-benzoyl-4-*O*-benzyl-β-D-xylopyranosyl-(1→3)-2-*O*-benzoyl-4-*O*-benzyl-β-D-xylopyranosyl-(1→3)-2-*O*-benzoyl-4-*O*-benzyl-β-D-xylopyranosyl-(1→3)-2-*O*-benzoyl-4-*O*-benzyl-β-D-xylopyranosyl-(1→3)-2-*O*-benzoyl-4-*O*-benzyl-β-D-xylopyranosyl-(1→3)-2-*O*-benzoyl-4-*O*-benzyl-β-D-xylopyranosyl-(1→3)-2-*O*-benzoyl-4-*O*-benzyl-β-D-xylopyranoside (**10**)

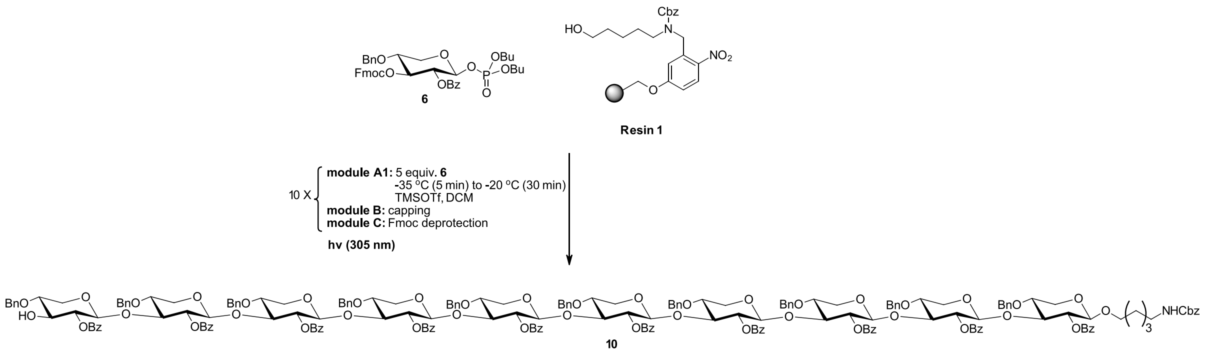

Experimental procedure: Linker-functionalized resin **1** (38.0 mg, 12.5 µmol) was placed in the synthesizer and synthesizer modules were applied as follows: 10 × [module A1 (BB **6**; total amount used = 474 mg, 0.625 mmol, 5 × 10 = 50 equiv.) at –35 ºC (5 min) to –20 ºC (30 min), module B, and module C]. Cleavage from the resin using UV irradiation at 305 nm in a continuous flow photoreactor afforded the crude product. Purification of the crude by normal phase HPLC using a preparative YMC-Small column (EtOAc/hexanes = 1/2.3 to 1/1, v/v) gave protected β3-xylan decasaccharide **10** (11.8 mg, 27% yield over 21 steps) as a glassy solid.

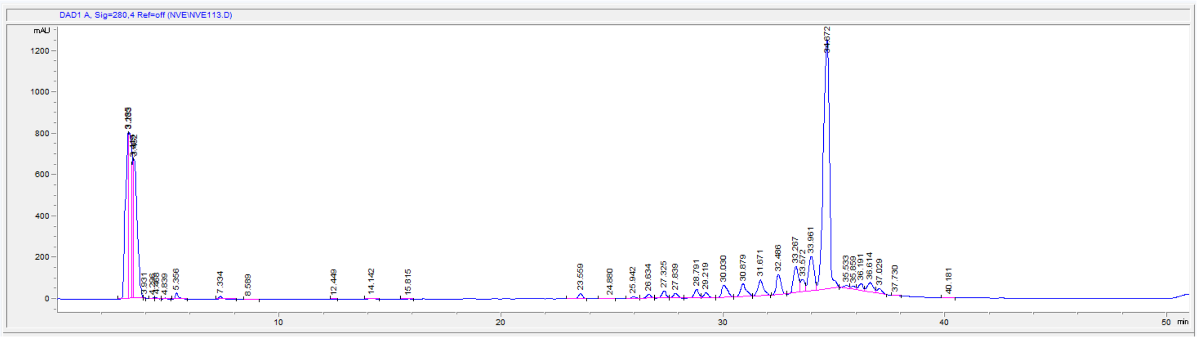

Analytical HPLC of the crude reaction mixture was performed on a YMC-Small NP column using a gradient of EtOAc in hexanes (50 min, flow rate 1 mL/min).

^{1}H NMR (600 MHz, (CD$_3$)$_2$CO): δ 7.93-7.88 (m, 2H, Ar-H), 7.88-7.84 (m, 2H, Ar-H), 7.74-7.64 (m, 14H, Ar-H), 7.61-7.57 (m, 2H, Ar-H), 7.54-7.50 (m, 2H, Ar-H), 7.49-7.17 (m, 83H, Ar-H), 6.06 (br, 1H, N-H), 5.05-4.98 (m, 3H, CH$_2$Cbz, H-2J), 4.88 (dd, J = 5.0, 6.2 Hz, 1H, H-2I), 4.82 (d, J = 7.4 Hz, 1H, H-1J), 4.78-4.73 (m, 5H, H-2A, H-1I), 4.73-4.64 (m, 16H, O-H), 4.64-4.55 (m, 10H), 4.52-4.46 (m, 7H), 4.43 (d, J = 7.0 Hz, 1H, H-1A), 4.05-4.0 (m, 3H, H-5J, H-3I, H-5I), 3.94 (t, J = 8.2 Hz, 1H, H-3A), 3.93-3.88 (m, 2H), 3.88-3.82 (m, 8H, H-5A), 3.81-3.78 (m, 4H), 3.73-3.67 (m, 1H, H-3J), 3.61-3.54 (m, 2H, H-4J), 3.51-3.47 (m, 1H, H-4I), 3.34-3.24 (m, 6H, H-4A, H-5A, H-5I), 3.23-3.17 (m, 10H, H-5J), 3.17-3.15 (m, 2H), 3.15-3.12 (m, 1H), 2.93-2.86 (m, 2H, CH_2-NHCbz), 1.36-1.28 (m, 4H, CH$_2$), 1.15-1.09 (m, 2H, CH$_2$) ppm.

^{13}C NMR (151 MHz, (CD$_3$)$_2$CO): δ 166.0, 165.28, 165.26, 165.24, 165.23, 165.21, 165.07, 139.95, 139.80, 139.79, 139.77, 139.70, 133.87, 133.77, 133.73, 133.56, 131.31, 130.97, 130.65, 130.62,

130.60, 130.54, 130.48, 130.41, 130.35, 129.27, 129.25, 129.19, 129.14, 129.12, 129.05, 129.01, 128.97, 128.96, 128.94, 128.74, 128.73, 128.67, 128.65, 128.58, 128.56, 128.53, 128.29, 128.24, 128.22, 128.18, 101.60, 101.37, 100.53, 100.38, 100.25, 100.22, 100.08, 78.67, 78.50, 76.80, 76.71, 76.56, 76.47, 76.37, 76.34, 76.28, 76.11, 76.06, 76.00, 75.71, 74.95, 74.92, 74.12, 73.52, 73.45, 73.40, 73.15, 72.96, 72.94, 72.92, 72.87, 72.84, 72.79, 72.74, 72.56, 72.36, 69.3, 66.3, 64.5, 63.7, 62.93, 62.51, 62.41, 62.35, 61.8, 41.4, 29.8, 23.7 ppm.

ESI-HRMS: m/z $[M + 2Na]^{2+}$ calcd. for $C_{203}H_{199}NO_{53}Na_2$: 1772.1350; found 1772.1385.

NMR chemical shifts of selected ^{1}H and ^{13}C atoms in compound **10**:

**xylose ring**	**proton**	***δ* (ppm)**	**multiplicity**	***J* (Hz)**
A (reducing end)	H-1A	4.43	d	7.0
	H-2A	4.78-4.73	m	
	H-3A	3.94	t	8.2
	H-4A	3.34-3.24	m	
	H-5Aa	3.34-3.24	m	
	H-5Ab	3.88-3.82	m	
B				
C				
D				
E				
F				
G				
H				
I	H-1I	4.78-4.73	m	
	H-2I	4.88	dd	5.0, 6.2
	H-3I	4.05-4.0	m	
	H-4I	3.51-3.47	m	
	H-5Ia	3.34-3.24	m	
	H-5Ib	4.05-4.0	m	
J (non-reducing end)	H-1J	4.82	d	7.4
	H-2J	5.05-4.98	m	
	H-3J	3.73-3.67	m	
	H-4J	3.61-3.54	m	
	H-5Ja	3.23-3.17	m	
	H-5Jb	4.05-4.0	m	

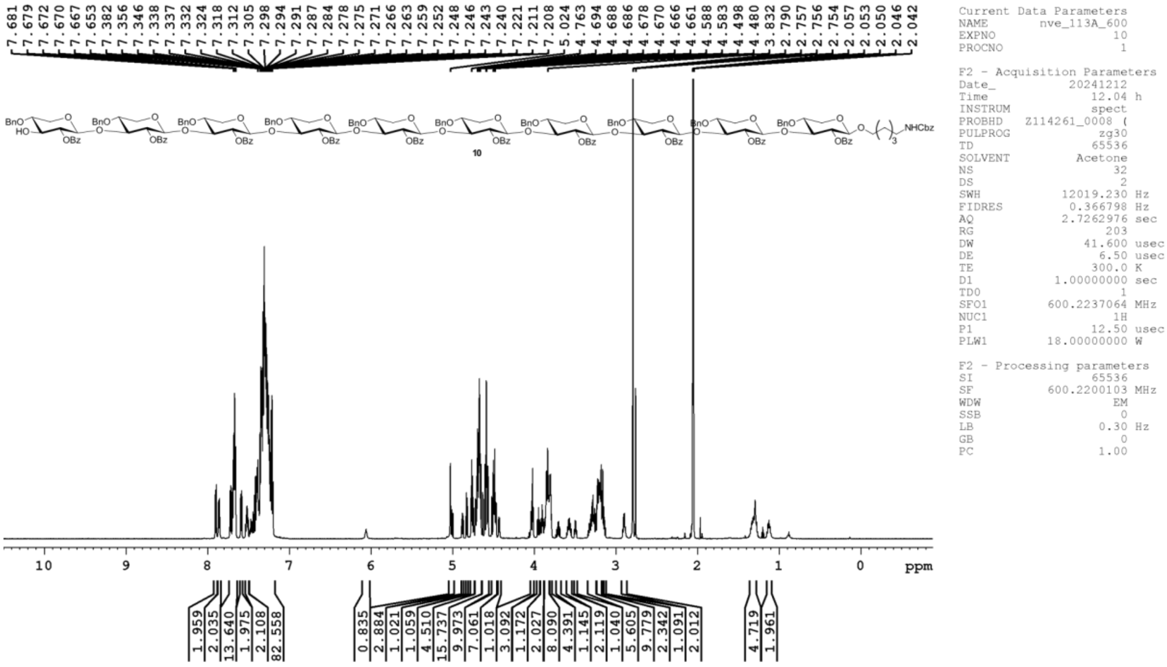

^{1}H NMR spectrum of compound **10** (600 MHz, $(CD_3)_2CO$)

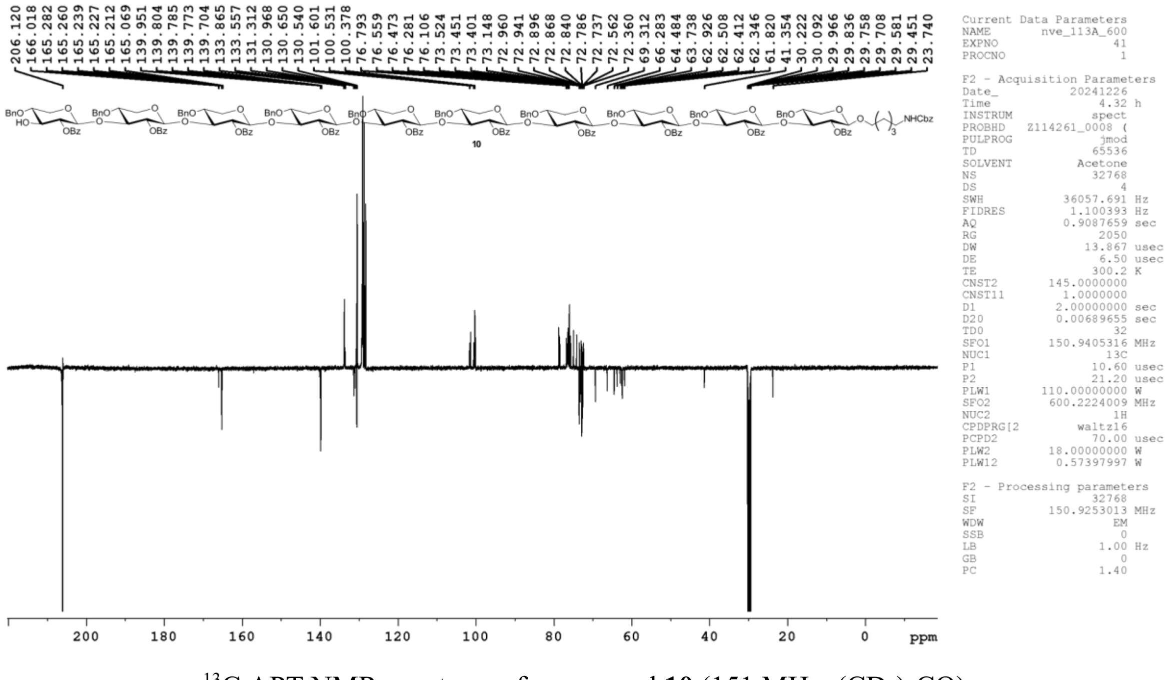

^{13}C APT NMR spectrum of compound **10** (151 MHz, $(CD_3)_2CO$)

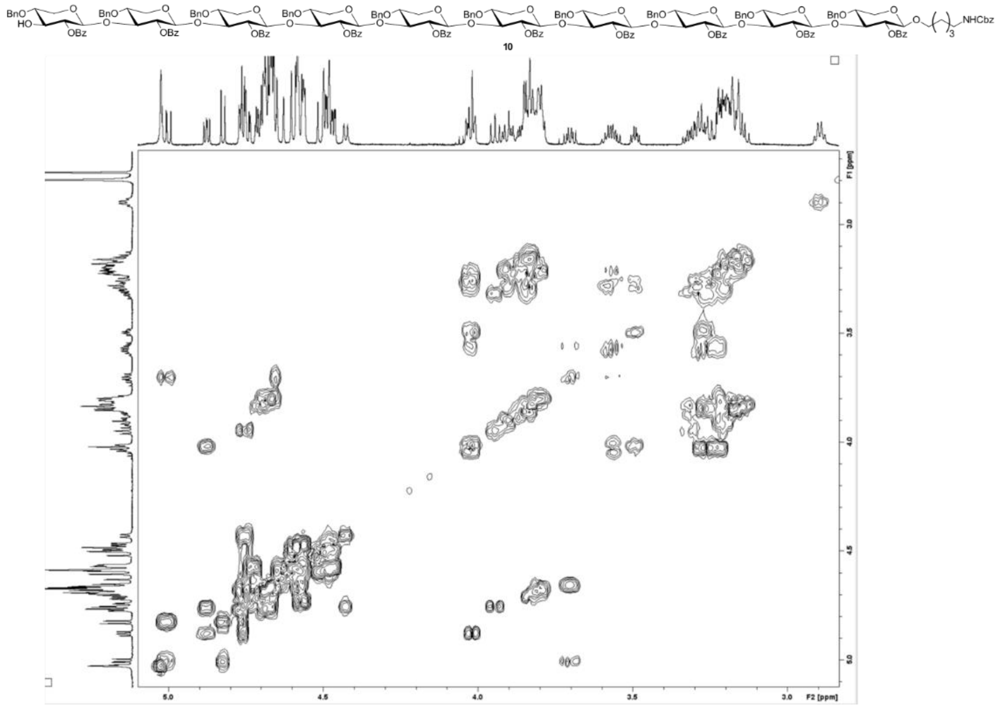

^1H-^1H COSY NMR spectrum of compound **10** (600 MHz, $(CD_3)_2CO$)

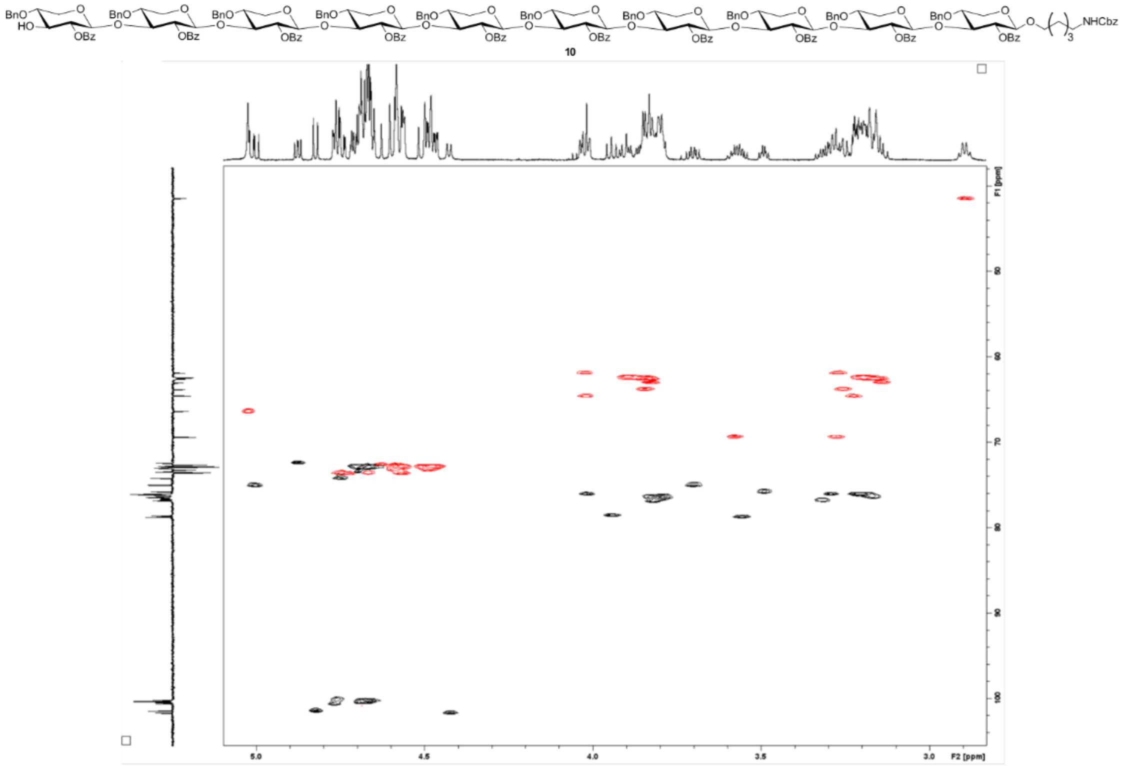

^1H-^{13}C HSQC NMR spectrum of compound **10** (600/151 MHz, $(CD_3)_2CO$)

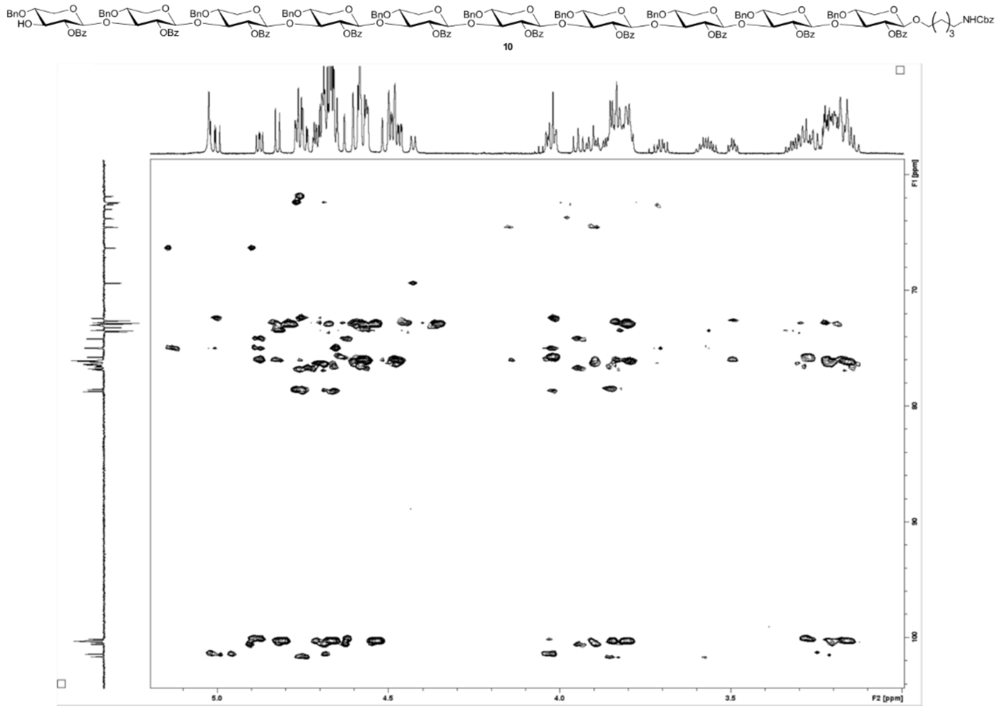

^{1}H-^{13}C HMBC NMR spectrum of compound **10** (600/151 MHz, $(CD_3)_2CO$)

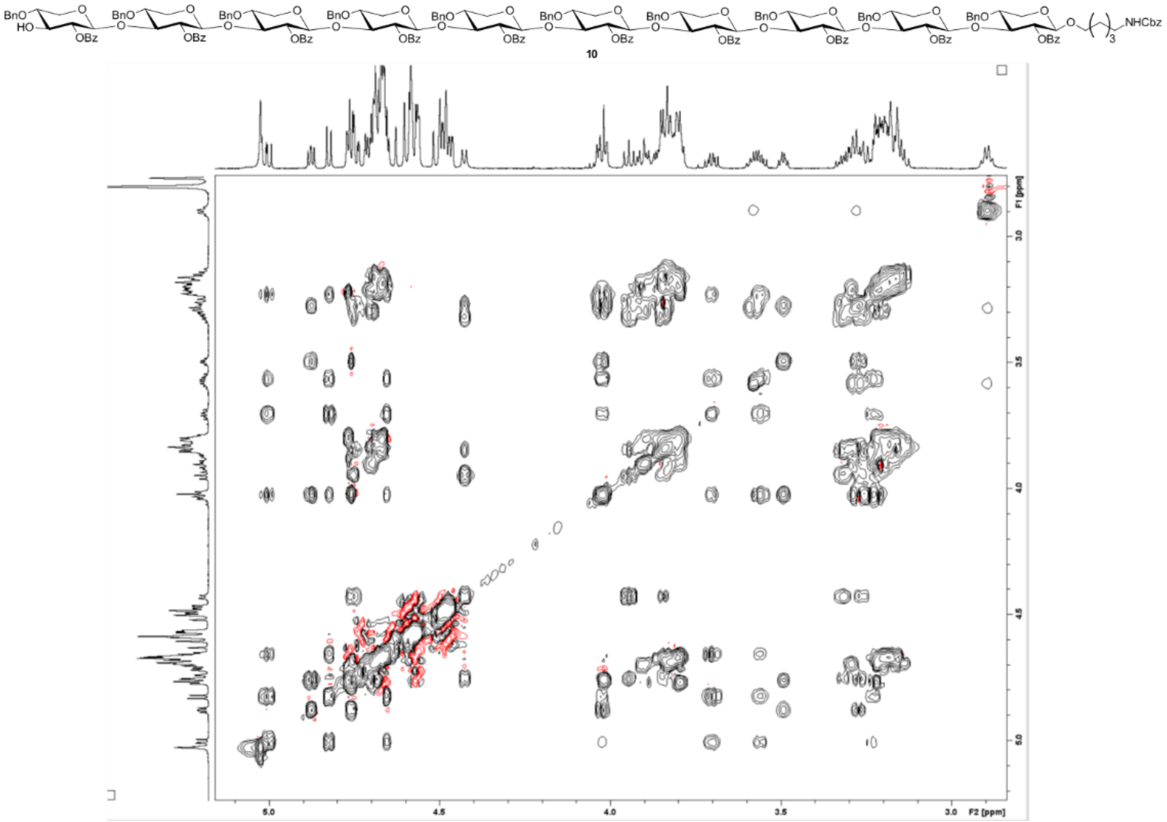

^{1}H-^{1}H TOCSY NMR spectrum of compound **10** (600 MHz, $(CD_3)_2CO$)

Aminopentyl β-D-xylopyranosyl-(1→3)-β-D-xylopyranosyl-(1→3)-β-D-xylopyranosyl-(1→3)-β-D-xylopyranosyl-(1→3)-β-D-xylopyranosyl-(1→3)-β-D-xylopyranosyl-(1→3)-β-D-xylopyranosyl-(1→3)-β-D-xylopyranosyl-(1→3)-β-D-xylopyranosyl-(1→3)-β-D-xylopyranoside (14)

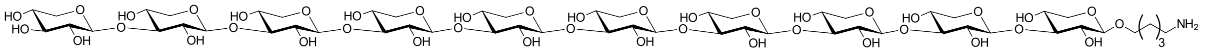

14

Experimental procedure: To a solution of protected decasaccharide **10** (8.3 mg, 2.4 μmol) in THF (237 μL, 10 mM), a solution of NaOMe in MeOH (0.5 M, 237 μL, 0.12 mmol) was added at rt, and the reaction mixture was allowed to stir overnight. After confirmation of reaction completion (24 h) by TLC (R_f: 0.34 (MeOH/$CHCl_3$ = 1/15, v/v)), the reaction mixture was neutralized by the addition of IR-120 H^+ resin while slowly stirring the mixture. Then, the reaction mixture was filtered, and the filtrate was concentrated under reduced pressure to yield a partially-deprotected glassy crude, which was kept under high vacuum until usage in the next step without any further purification. To a solution of partially deprotected crude (2.4 μmol) in *t*-BuOH (0.47 mL), H_2O (0.24 mL), and AcOH (0.25 mL), unreduced 10% Pd/C (12.5 mg) was added and the reaction mixture was stirred in the H_2 reactor under a pressure of 8 bar H_2. After 43 h, the reaction progress was checked by MALDI/TOF-MS analysis. After 46 h, the reaction mixture was filtered using a PTFE syringe filter (0.45 μm) and concentrated under reduced pressure to yield a crude product, which was purified using pre-packed C18 (500 mg, 3 mL) column chromatography with H_2O/ACN (100% to 80%, v/v, + 0.1% AcOH). The purified product was lyophilized to give β3-xylan decasaccharide **14** (1.7 mg, 47% yield over 2 steps) as a white amorphous foam.

^{1}H NMR (600 MHz, D_2O): δ 4.72-4.65 (m, 9H, H-1B, H-1C, H-1D, H-1E, H-1F, H-1G, H-1H, H-1I, H-1J), 4.41 (d, J = 7.9 Hz, 1H, H-1A), 4.02-3.94 (m, 10H, H-5A, H-5B, H-5C, H-5D, H-5E, H-5F, H-5G, H-5H, H-5I, H-5J), 3.89-3.84 (m, 1H, OCH_2), 3.73-3.60 (m, 21H, OCH_2, H-3A), 3.54-3.49 (m, 8H), 3.47-3.39 (m, 2H, H-2A), 3.34-3.27 (m, 11H, H-5A, H-5B, H-5C, H-5D, H-5E, H-5F, H-5G, H-5H, H-5I, H-5J), 3.00 (t, J = 7.6 Hz, 2H, CH_2-NH_2), 1.71-1.62 (m, 4H, CH_2), 1.47-1.40 (m, 2H, CH_2) ppm.

^{13}C NMR (151 MHz, D_2O): δ 104.04, 103.83, 103.79, 103.30, 84.39, 84.25, 84.14, 76.2, 73.98, 73.71, 73.65, 73.33, 70.76, 69.80, 68.3, 65.77, 65.43, 40.0, 28.8, 27.0, 22.7 ppm.

ESI-HRMS: m/z [M - H]$^-$ calcd. for $C_{55}H_{92}NO_{41}$: 1422.5150; found 1422.5130.

NMR chemical shifts of selected ^{1}H and ^{13}C atoms in compound **14**:

xylose ring	proton	δ (ppm)	multiplicity	J (Hz)	carbon	δ (ppm)
A (reducing end)	H-1A	4.41	d	7.9	C-1A	103.30
	H-2A	3.47-3.39	m			
	H-3A	3.73-3.60	m			
	H-5Aa	3.34-3.27	m			
	H-5Ab	4.02-3.94	m			
B	H-1B	4.72-4.65	m			
	H-5Ba	3.34-3.27	m			
	H-5Bb	4.02-3.94	m			

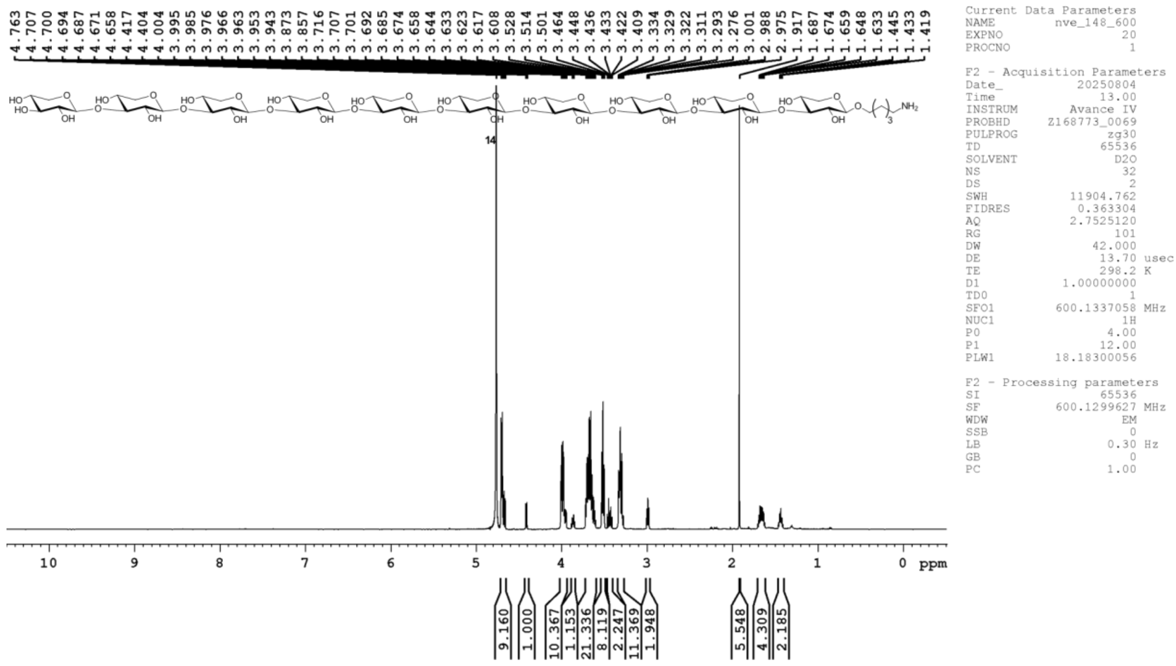

^{1}H NMR spectrum of compound **14** (600 MHz, D_2O)

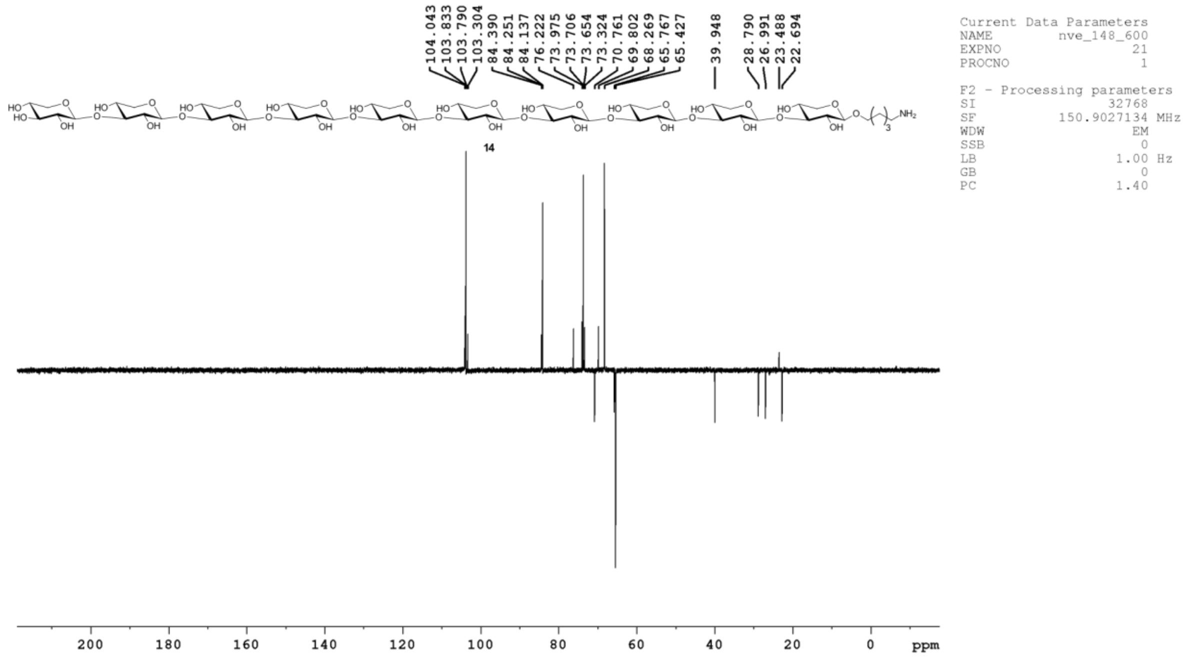

^{13}C DEPTQ135 NMR spectrum of compound **14** (151 MHz, D_2O)

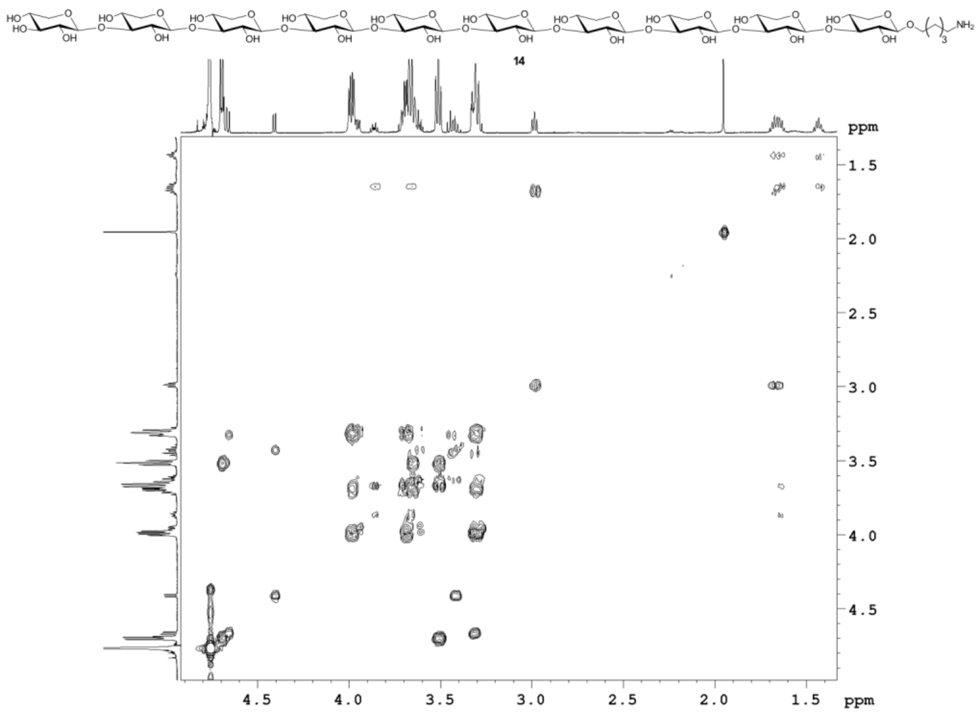

^{1}H-^{1}H COSY NMR spectrum of compound **14** (600 MHz, D_2O)

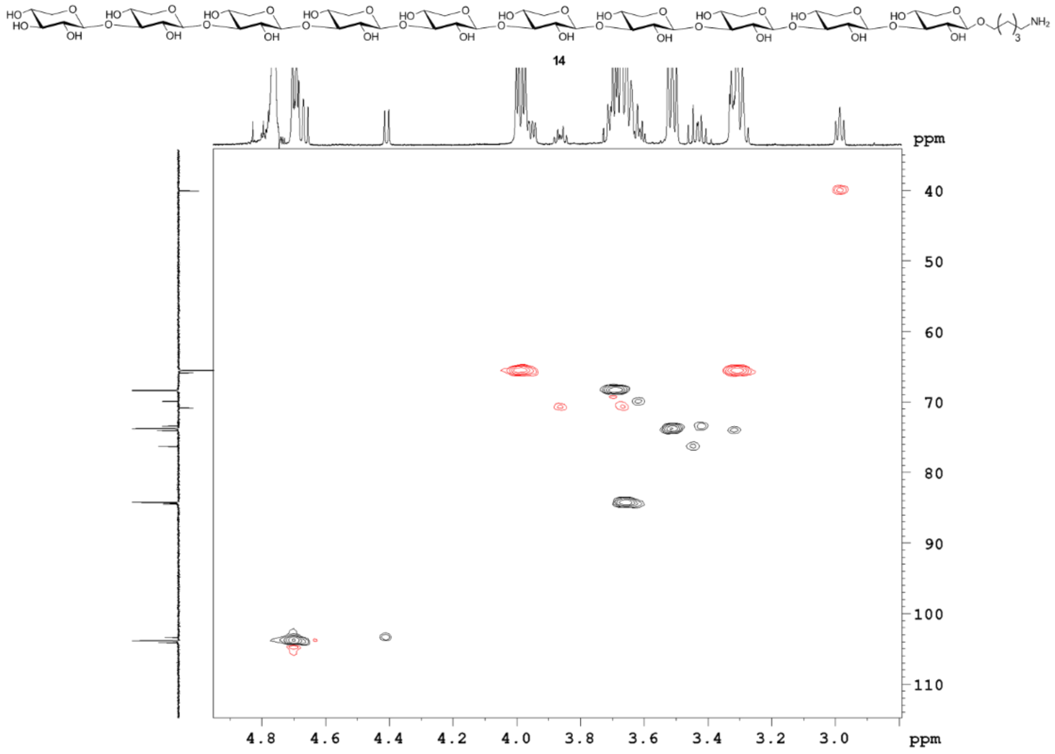

^{1}H-^{13}C HSQC NMR spectrum of compound **14** (600/151 MHz, D_2O)

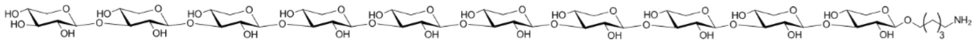

14

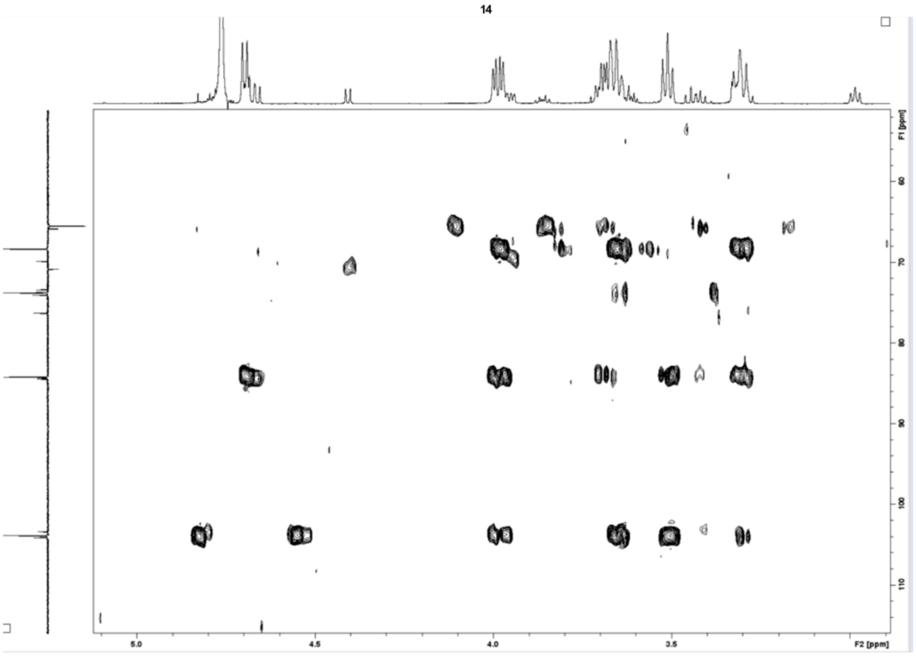

^1H-^{13}C HMBC NMR spectrum of compound **14** (600/151 MHz, D_2O)

4.3 AGA of mixed-linkage xylan (MLX) oligosaccharides

AGA of MLX trisaccharide S17

Benzyloxycarbonylaminopentyl 2-*O*-benzoyl-3-*O*-benzyl-β-D-xylopyranosyl-(1→3)-2-*O*-benzoyl-4-*O*-benzyl-β-D-xylopyranosyl-(1→4)-2-*O*-benzoyl-3-*O*-benzyl-β-D-xylopyranoside (**S17**)

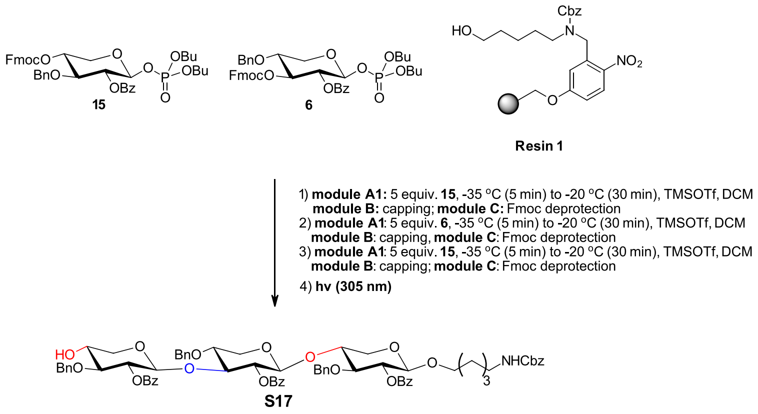

Experimental procedure: Linker-functionalized resin **1** (38 mg, 12.5 μmol) was placed in the synthesizer and synthesizer modules were applied as follows:

1) module A1 (BB **15**[1], 47 mg, 0.0625 mmol, 5 equiv.) at –35 °C (5 min) to –20 °C (30 min), module B, and module C.
2) module A1 (BB **6**, 47 mg, 0.0625 mmol, 5 equiv.) at –35 °C (5 min) to –20 °C (30 min), module B, and module C.
3) module A1 (BB **15**, 47 mg, 0.0625 mmol, 5 equiv.) at –35 °C (5 min) to –20 °C (30 min), module B, and module C.

Cleavage from the resin using UV irradiation at 305 nm in a continuous flow photoreactor afforded the crude product. Purification of the crude by normal phase HPLC using a preparative YMC-Small column (EtOAc/hexanes = 1/2.3 to 1/1, v/v) gave protected MLX trisaccharide **S17** (3.3 mg, 22% yield over 7 steps) as a glassy solid, capped linker (1.8 mg, 52%), and linker-attached disaccharide as a deletion sequence (1.8 mg, 16%).

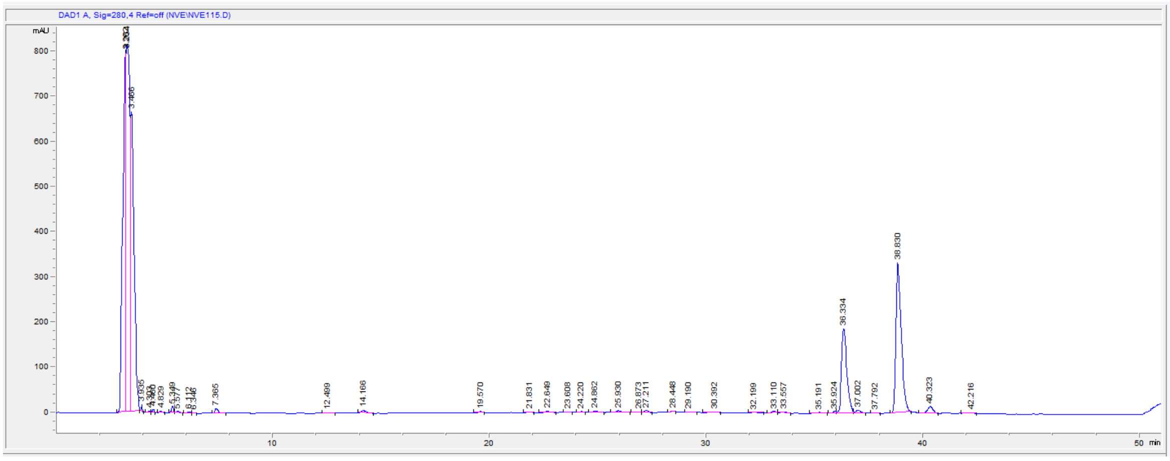

Analytical HPLC of the crude reaction mixture was performed on a YMC-Small NP column using a gradient of EtOAc in hexanes (50 min, flow rate 1 mL/min). MLX trisaccharide **S17** was eluted at 38.8 min.

^{1}H NMR (600 MHz, $(CD_3)_2CO$): δ 8.08-8.03 (m, 4H, Ar-H), 8.03-7.99 (m, 2H, Ar-H), 7.73-7.69 (m, 1H, Ar-H), 7.66-7.61 (m, 2H, Ar-H), 7.60-7.56 (m, 2H, Ar-H), 7.55-7.50 (m, 2H, Ar-H), 7.49-7.45 (m, 2H, Ar-H), 7.39-7.33 (m, 6H, Ar-H), 7.32-7.26 (m, 4H, Ar-H), 7.21-7.18 (m, 2H, Ar-H), 7.15-7.08 (m, 8H, Ar-H), 6.09 (br, 1H, N-H), 5.17 (dd, J = 7.6, 9.2 Hz, 1H, H-2C), 5.05-4.99 (m, 4H, H-2B, H-1C, CH_2Cbz), 4.96 (dd, J = 7.8, 9.3 Hz, 1H, H-2A), 4.88-4.83 (m, 2H, H-1B, CH_2-Ph), 4.79 (d, J = 11.6 Hz, 1H, CH_2-Ph), 4.70 (d, J = 11.7 Hz, 1H, CH_2-Ph), 4.64 (d, J = 11.7 Hz, 1H, CH_2-Ph), 4.63-4.57 (m, 3H, O-H, CH_2-Ph), 4.25-4.19 (m, 2H, H-1A, H-3B), 4.11 (dd, J = 3.7, 12.3 Hz, 1H, H-5B), 3.99 (dd, J = 5.3, 11.6 Hz, 1H, H-5C), 3.89-3.84 (m, 2H, H-5A, H-4C), 3.83-3.78 (m, 1H, H-4A), 3.70-3.64 (m, 1H, OCH_2), 3.63-3.57 (m, 2H, H-4B, H-3C), 3.49-3.41 (m, 2H, H-3A, H-5B), 3.40-3.32 (m, 2H, H-5C, OCH_2), 2.96-2.87 (m, 2H, CH_2-NHCbz), 2.63 (dd, J = 10.1, 11.5 Hz, 1H, H-5A), 1.47-1.39 (m, 2H, CH_2), 1.37-1.32 (m, 2H, CH_2), 1.24-1.16 (m, 2H, CH_2) ppm.

^{13}C NMR (151 MHz, $CD_3)_2CO$): δ 164.86, 164.74, 164.73, 138.85, 138.83, 138.75, 133.22, 133.09, 133.05, 130.60, 130.39, 130.00, 129.90, 129.68, 129.46, 128.60, 128.55, 128.51, 128.27, 128.08, 127.86, 127.83, 127.72, 127.64, 127.52, 127.30, 127.10, 127.07, 101.53, 101.31, 97.5, 82.5, 79.9, 76.0, 74.86, 74.62, 74.43, 73.82, 73.21, 73.04, 71.63, 71.38, 70.3, 68.8, 65.93, 65.37, 62.3, 60.4, 40.4, 22.8 ppm.

ESI-HRMS: m/z $[M + HCOO]^-$ calcd. for $C_{71}H_{74}NO_{20}$: 1260.4810; found 1260.4802.

NMR chemical shifts of selected ^{1}H and ^{13}C atoms in compound **S17**:

xylose ring	proton	δ (ppm)	multiplicity	J (Hz)	carbon	δ (ppm)
A (reducing end)	H-1A	4.22			C-1A	101.31
	H-2A	4.96	dd	7.8, 9.3	C-2A	73.21
	H-3A	3.47			C-3A	79.9
	H-4A	3.83-3.78	m		C-4A	74.86
	H-5Aa	2.63	dd	10.1, 11.5		
	H-5Ab	3.87				
B	H-1B	4.85			C-1B	97.5
	H-2B	5.01			C-2B	71.38
	H-3B	4.20			C-3B	76.0
	H-4B	3.61			C-4B	74.62
	H-5Ba	3.44			C-5B	60.4
	H-5Bb	4.11	dd	3.7, 12.3		
C (non-reducing end)	H-1C	5.01			C-1C	101.53
	H-2C	5.17	dd	7.6, 9.2	C-2C	73.04
	H-3C	3.60				
	H-4C	3.85			C-4C	70.3
	H-5Ca	3.34				
	H-5Cb	3.99	dd	5.3, 11.6		

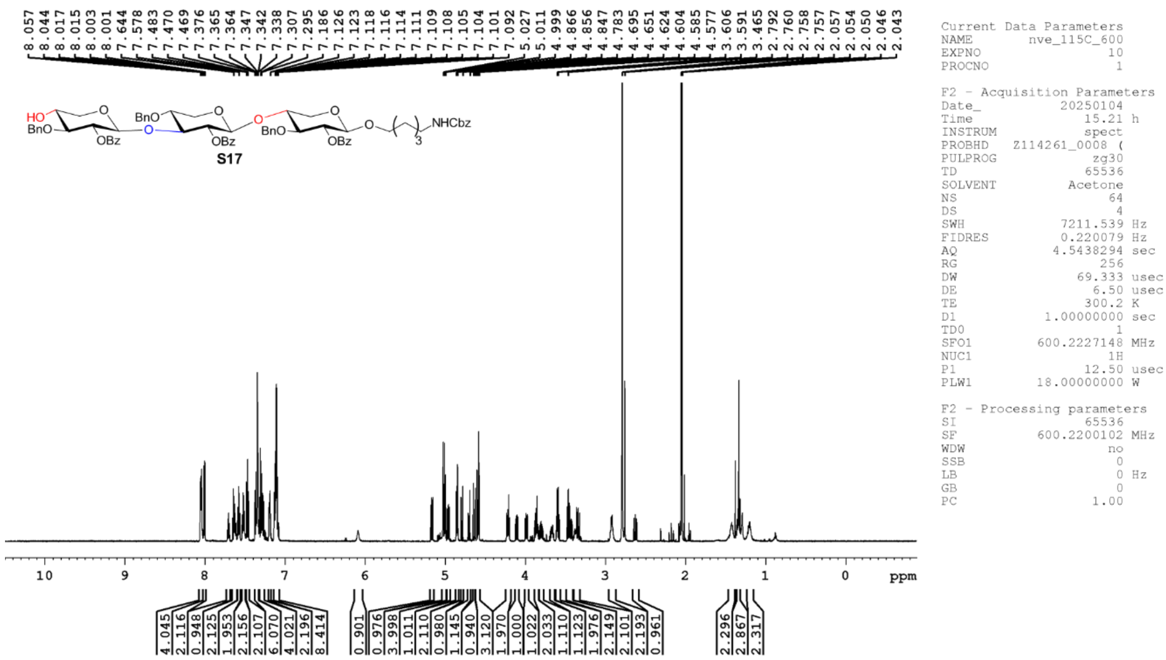

^{1}H NMR spectrum of compound **S17** (600 MHz, $(CD_3)_2CO$)

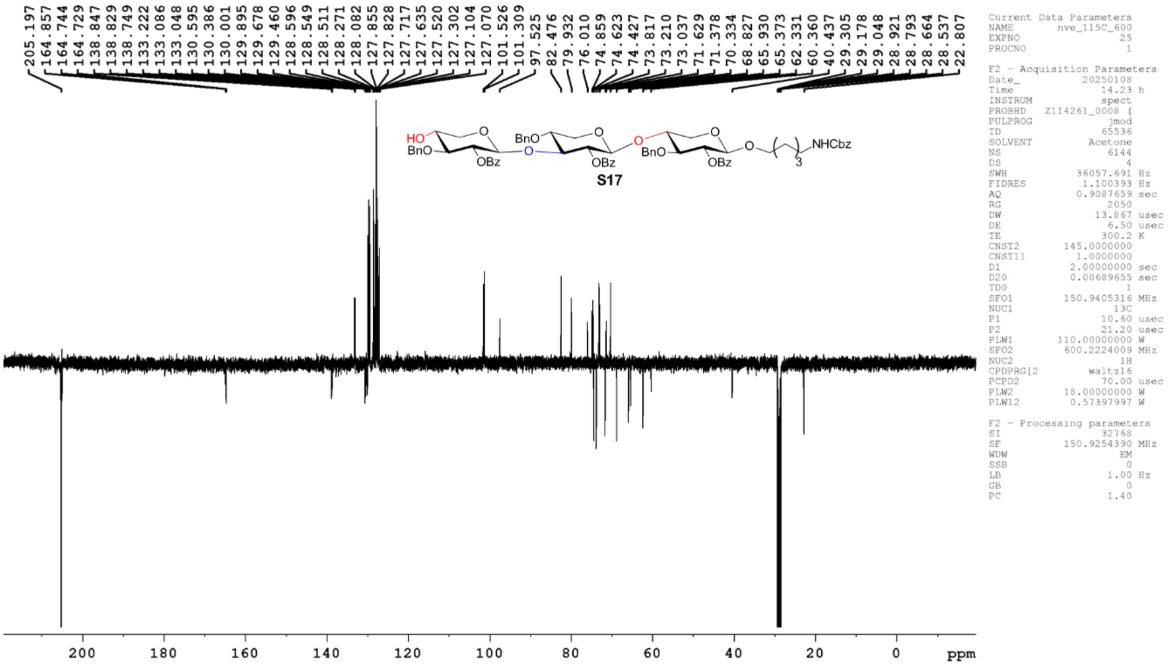

^{13}C APT NMR spectrum of compound **S17** (151 MHz, $(CD_3)_2CO$)

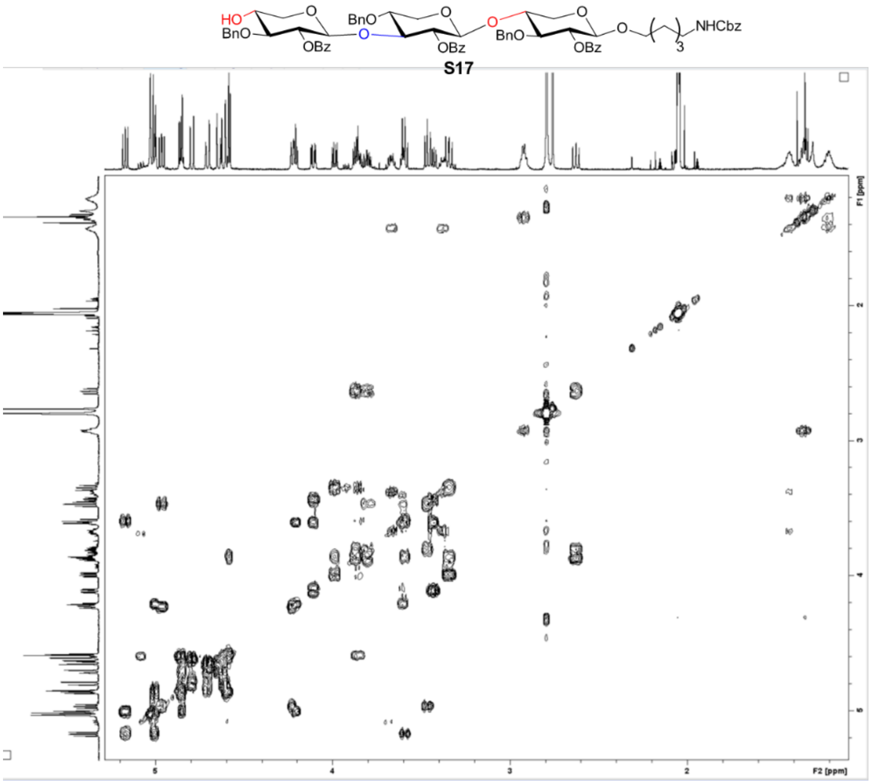

^{1}H-^{1}H COSY NMR spectrum of compound **S17** (600 MHz, $(CD_3)_2CO$)

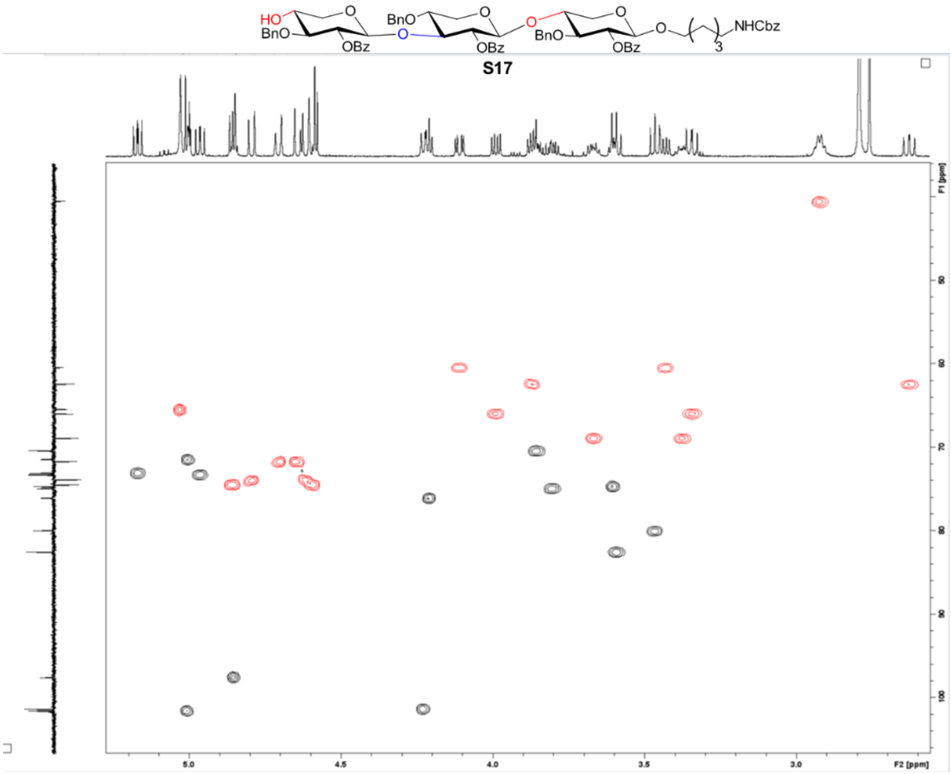

^{1}H-^{13}C HSQC NMR spectrum of compound **S17** (600/151 MHz, $(CD_3)_2CO$)

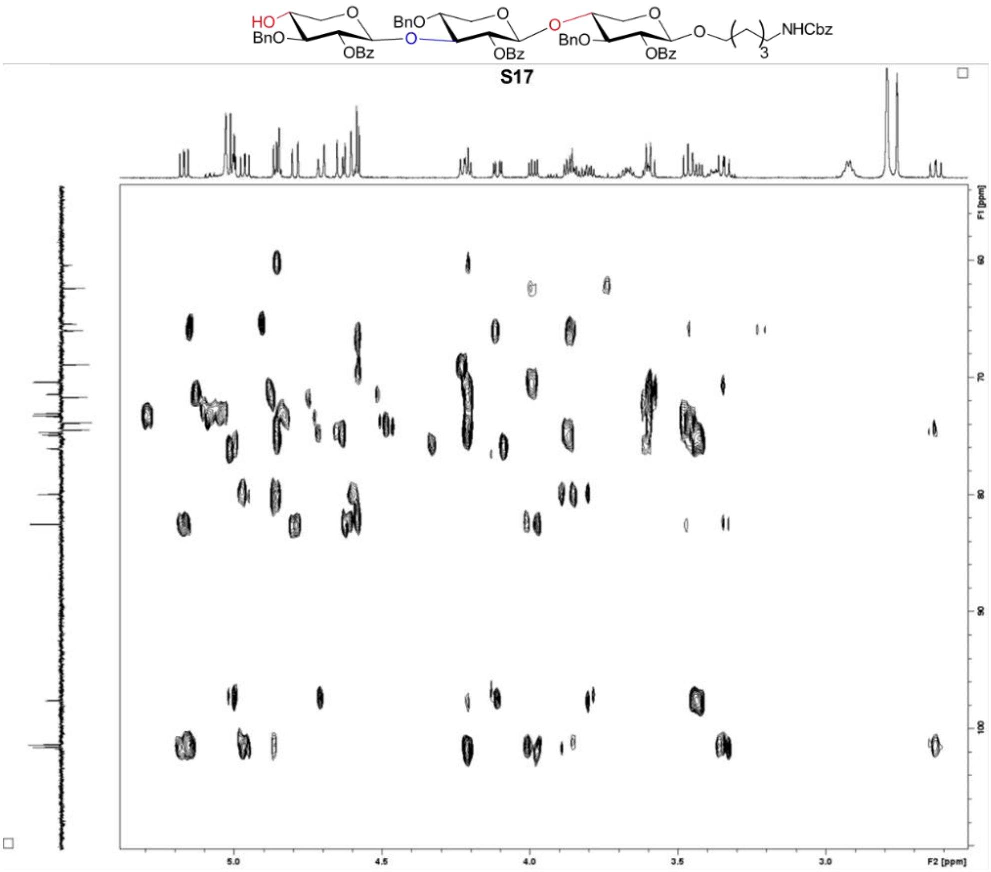

^{1}H-^{13}C HMBC NMR spectrum of compound **S17** (600/151 MHz, $(CD_3)_2CO$)

Aminopentyl β-D-xylopyranosyl-(1→3)-β-D-xylopyranosyl-(1→4)-β-D-xylopyranoside (16)

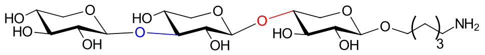

16

Experimental procedure: To a solution of protected MLX trisaccharide **S17** (3.3 mg, 2.71 µmol) in THF (54 µL, 50 mM), a solution of NaOMe in MeOH (0.5 M, 41 µL, 20.3 µmol) was added at rt, and the reaction mixture was allowed to stir overnight. After confirmation of reaction completion (23 h) by TLC (R_f: 0.37 (MeOH/$CHCl_3$ = 1/15, v/v)), the reaction mixture was neutralized by the addition of IR-120 H^+ resin while slowly stirring the mixture. Then, the reaction mixture was filtered, and the filtrate was concentrated under reduced pressure to yield a partially-deprotected glassy crude, which was kept under high vacuum until usage in the next step without any further purification. To a solution of partially deprotected crude (2.71 µmol) in *t*-BuOH (0.4 mL), H_2O (0.2 mL), and AcOH (3 drops), unreduced 10% Pd/C (2.2 mg) was added and the reaction mixture was stirred in the H_2 reactor under a pressure of 8 bar H_2. After 28 h, the reaction mixture was filtered using a PTFE syringe filter (0.45 µm) and concentrated under reduced pressure to yield a crude product, which was purified using HILIC-HPLC column chromatography (13 mM NH_4OAc/ACN = 1/4 to 1/1, v/v). The purified product was lyophilized to give MLX trisaccharide **16** (1.36 mg, 55% yield over 2 steps) as a white amorphous solid.

^{1}H NMR (600 MHz, D_2O): δ 4.65 (d, J = 7.8 Hz, 1H, H-1C), 4.47 (d, J = 7.8 Hz, 1H, H-1B), 4.40 (d, J = 7.9 Hz, 1H, H-1A), 4.06 (dd, J = 5.3, 11.8 Hz, 1H, H-5A), 4.015-3.93 (m, 2H, H-5B, H-5C), 3.89-3.83 (m, 1H, OCH_2), 3.78-3.72 (m, 1H, H-4A), 3.71-3.65 (m, 2H, H-4B, OCH_2), 3.65-3.59 (m, 2H, H-3B, H-4C), 3.53 (t, J = 9.2 Hz, 1H, H-3A), 3.47-3.41 (m, 2H, H-2B, H-3C), 3.36 (t, J = 11.1 Hz, 1H, H-5A), 3.33-3.28 (m, 3H, H-2C, H-5B, H-5C), 3.27-3.23 (m, 1H, H-2A), 2.98 (t, J = 7.5 Hz, 2H, CH_2-NH_2), 1.71-1.60 (m, 4H, CH_2), 1.47-1.40 (m, 2H, CH_2) ppm.

^{13}C NMR (151 MHz, D_2O): δ 104.0, 103.4, 102.3, 84.1, 77.0, 76.2, 74.5, 73.95, 73.56, 73.07, 70.8, 69.8, 68.2, 65.76, 65.47, 63.5, 40.0, 28.8, 27.0, 22.7 ppm.

ESI-HRMS: m/z $[M + H]^+$ calcd. for $C_{20}H_{38}NO_{13}$: 500.2338; found 500.2343.

NMR chemical shifts of selected ^{1}H and ^{13}C atoms in compound **16**:

xylose ring	proton	δ (ppm)	multiplicity	J (Hz)	carbon	δ (ppm)
A (reducing end)	H-1A	4.40	d	7.9	C-1A	103.4
	H-2A	3.27-3.23	m		C-2A	73.56
	H-3A	3.53	t	9.2	C-3A	74.5
	H-4A	3.78-3.72	m		C-4A	77.0
	H-5Aa	3.36	t	11.1	C-5A	63.5
	H-5Ab	4.06	dd	5.3, 11.8		
B	H-1B	4.47	d	7.8	C-1B	102.3
	H-2B	3.44				
	H-3B	3.61				
	H-4B	3.68			C-4B	68.2
	H-5Ba	3.31			C-5B	65.47
	H-5Bb	3.99				
C (non-reducing end)	H-1C	4.65	d	7.8	C-1C	104.0
	H-2C	3.32				
	H-3C	3.44				
	H-4C	3.61				
	H-5Ca	3.29			C-5C	65.76
	H-5Cb	3.95				

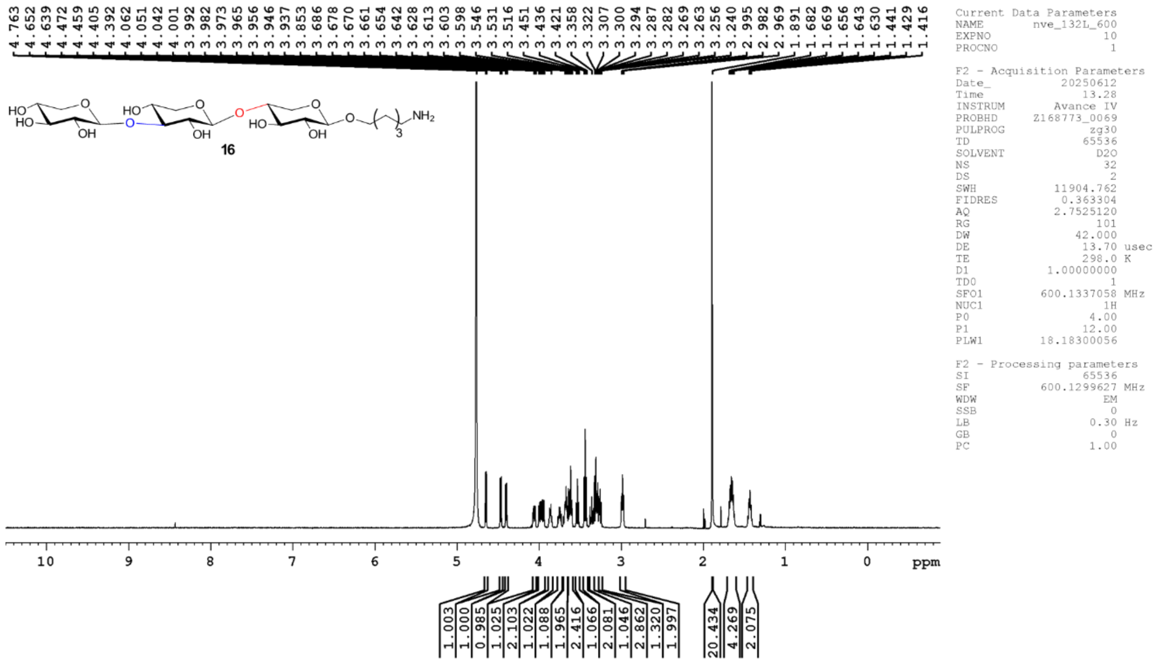

^{1}H NMR spectrum of compound **16** (600 MHz, D_2O)

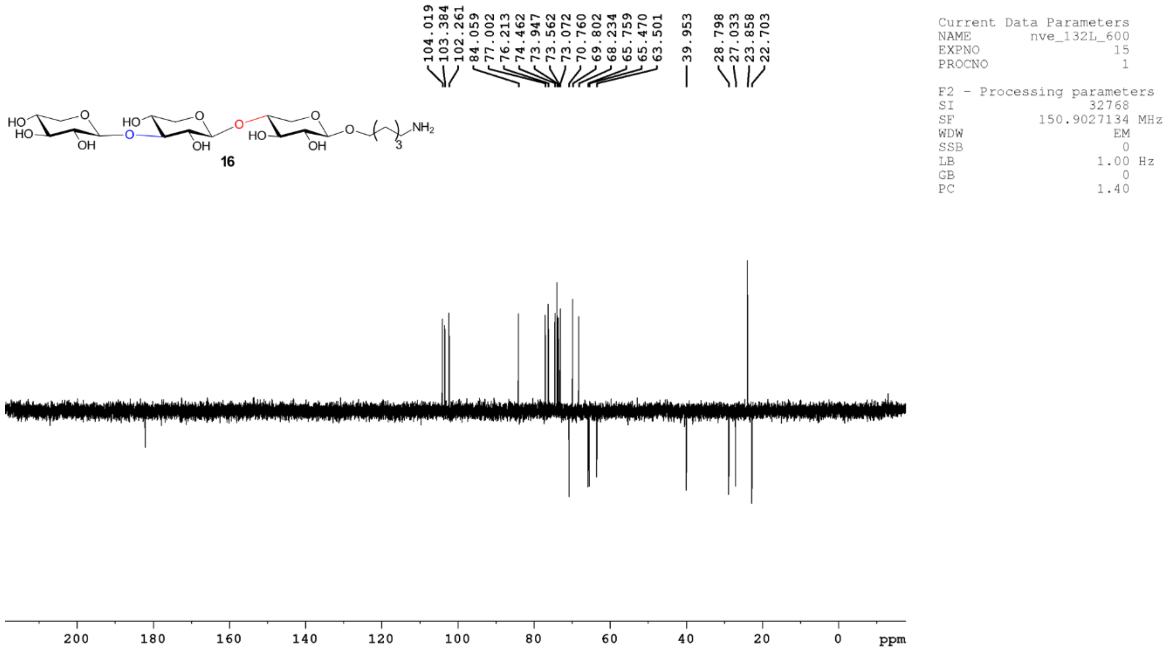

^{13}C APT NMR spectrum of compound **16** (151 MHz, D_2O)

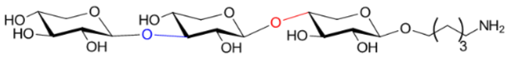

16

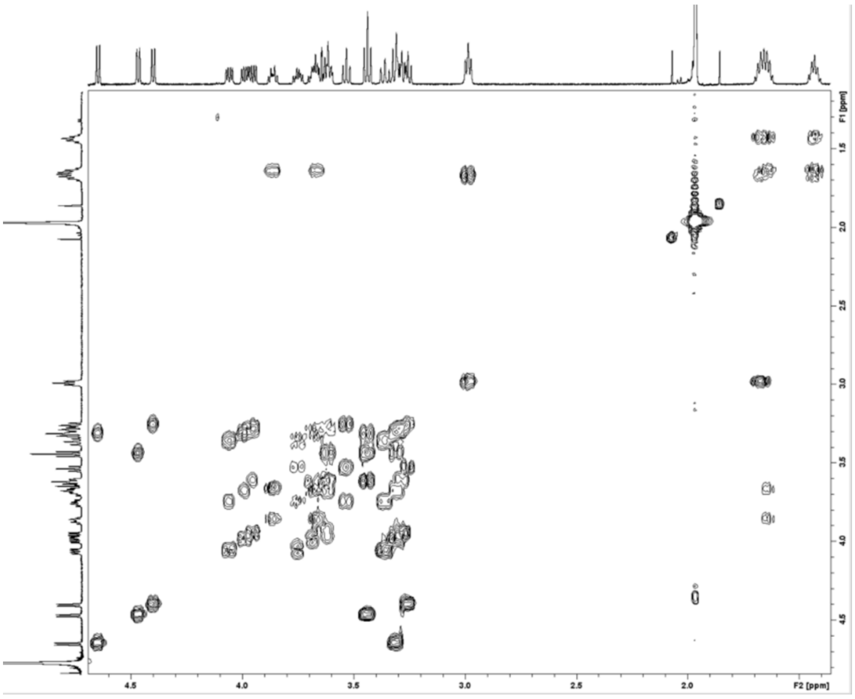

^{1}H-^{1}H COSY NMR spectrum of compound **16** (600 MHz, D_2O)

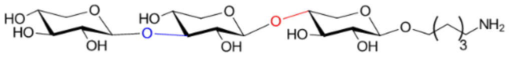

16

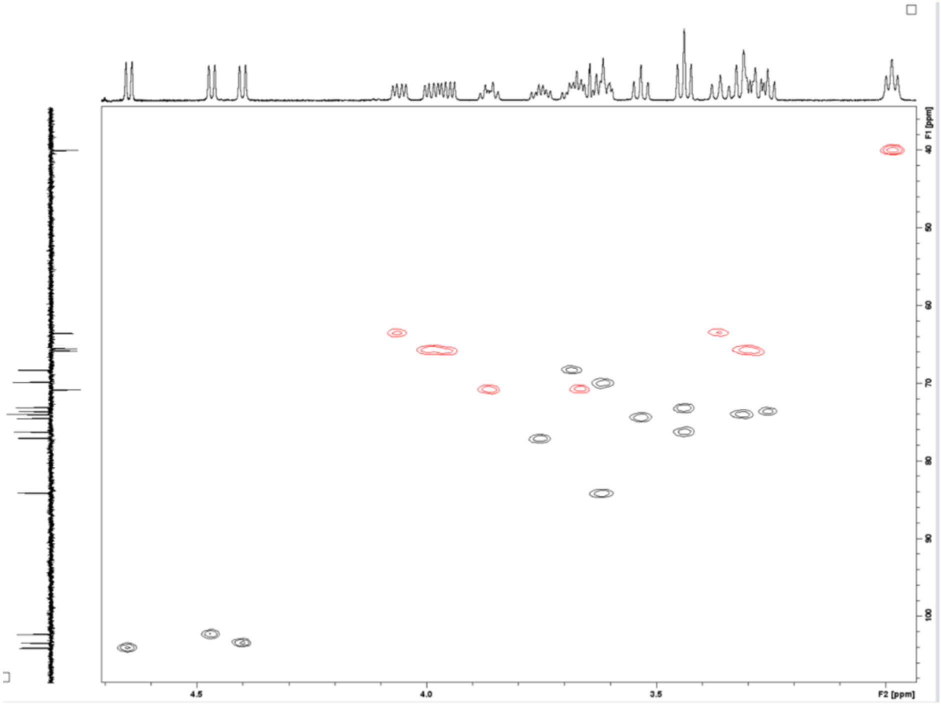

^{1}H-^{13}C HSQC NMR spectrum of compound **16** (600/151 MHz, D_2O)

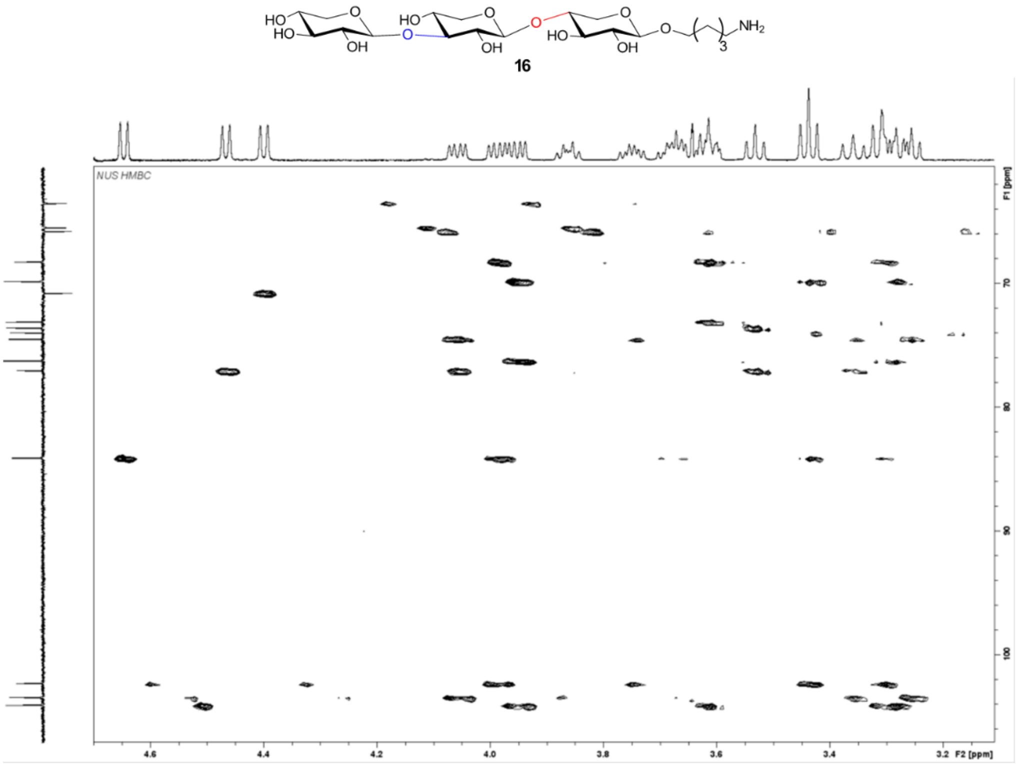

^{1}H-^{13}C HMBC NMR spectrum of compound **16** (600/151 MHz, D_2O)

AGA of MLX trisaccharide S18

Benzyloxycarbonylaminopentyl 2-*O*-benzoyl-4-*O*-benzyl-β-D-xylopyranosyl-(1→4)-2-*O*-benzoyl-3-*O*-benzyl-β-D-xylopyranosyl-(1→3)-2-*O*-benzoyl-4-*O*-benzyl-β-D-xylopyranoside (**S18**)

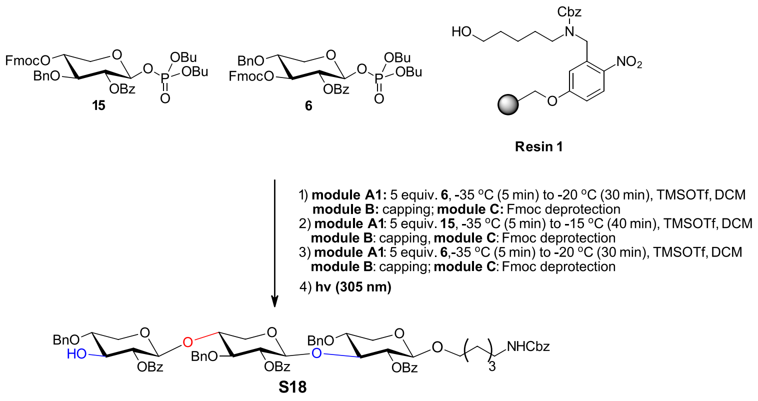

Experimental procedure: Linker-functionalized resin **1** (38 mg, 12.5 μmol) was placed in the synthesizer and synthesizer modules were applied as follows:

1) module A1 (BB **6**, 47 mg, 0.0625 mmol, 5 equiv.) at –35 °C (5 min) to –20 °C (30 min), module B, and module C.
2) module A1 (BB **15**, 47 mg, 0.0625 mmol, 5 equiv.) at –35 °C (5 min) to –15 °C (40 min), module B, and module C.
3) module A1 (BB **6**, 47 mg, 0.0625 mmol, 5 equiv.) at –35 °C (5 min) to –20 °C (30 min), module B, and module C.

Cleavage from the resin using UV irradiation at 305 nm in a continuous flow photoreactor afforded the crude product. Purification of the crude by normal phase HPLC using a preparative YMC-Small column (EtOAc/hexanes = 1/2.3 to 1/1, v/v) gave protected MLX trisaccharide **S18** (6.9 mg, 45% yield over 7 steps) as a glassy solid.

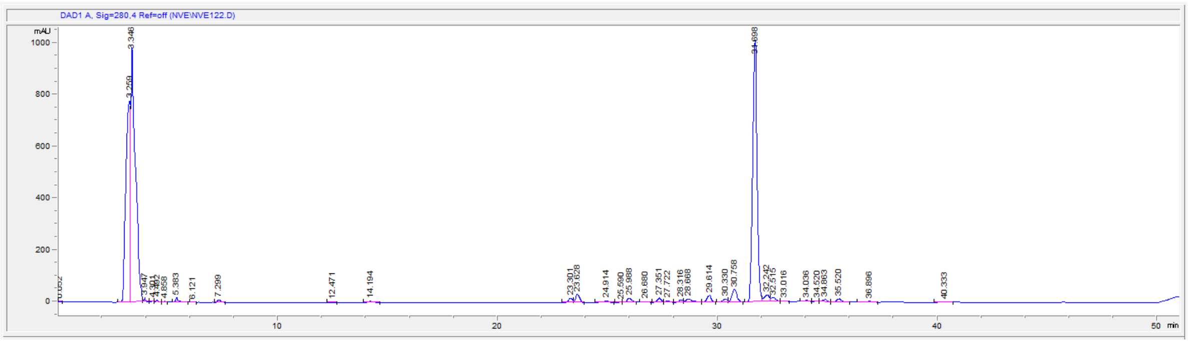

Analytical HPLC was performed on a YMC-Small NP column using a gradient of EtOAc in hexanes (50 min, flow rate 1 mL/min).

^{1}H NMR (600 MHz, $(CD_3)_2CO$): δ 8.13-8.09 (m, 2H, Ar-H), 7.86-7.82 (m, 2H, Ar-H), 7.75-7.71 (m, 2H, Ar-H), 7.69-7.65 (m, 1H, Ar-H), 7.60-7.52 (m, 4H, Ar-H), 7.41-7.25 (m, 19H, Ar-H), 7.13-7.06 (m, 5H, Ar-H), 6.11 (br, 1H, N-H), 5.07-5.0 (m, 4H, H-2B, H-2C, CH_2-Cbz), 4.93 (dd, *J* = 7.0, 8.5 Hz, 1H, H-2A), 4.86 (d, *J* = 5.1 Hz, 1H, O-H), 4.83-4.69 (m, 6H, H-1C, H-1B, CH_2-Ph), 4.63 (d, *J* = 11.8 Hz, 1H, CH_2-Ph), 4.52 (d, *J* = 11.3 Hz, 1H, CH_2-Ph), 4.48 (d, *J* = 7.0 Hz, 1H, H-1A), 4.09-3.99 (m, 3H, H-5B, H-3A, H-5C), 3.96-3.87 (m, 3H, H-5A, H-4B, H-3C), 3.63-3.54 (m, 3H, OCH_2, H-4C, H-4A), 3.52 (t, *J* = 8.1 Hz, 1H, H-3B), 3.36-3.25 (m, 3H, H-5A, H-5C, OCH_2), 3.09 (dd, *J* = 8.4,

12.0 Hz, 1H, H-5B), 2.89-2.84 (m, 2H, CH_2-NHCbz, merged with H_2O peak in acetone-d_6), 1.35-1.25 (m, 4H, CH_2), 1.13-1.05 (m, 2H, CH_2) ppm.

^{13}C NMR (151 MHz, $(CD_3)_2CO$): δ 165.81, 165.50, 165.25, 139.94, 139.85, 139.41, 133.98, 133.85, 133.54, 131.41, 131.03, 130.90, 130.53, 130.45, 130.36, 129.45, 129.24, 129.17, 129.04, 129.01, 128.80, 128.71, 128.69, 128.61, 128.53, 128.51, 128.28, 128.26, 128.02, 101.60, 101.46, 101.35, 79.6, 78.88, 78.61, 77.5, 76.7, 75.37, 75.05, 74.1, 73.96, 73.53, 73.42, 72.95, 69.4, 66.2, 64.6, 63.71, 63.15, 41.3, 30.2, 23.7 ppm.

ESI-HRMS: m/z $[M + K]^+$ calcd. for $C_{70}H_{73}NO_{18}K$: 1254.4459; found 1254.4480.

NMR chemical shifts of selected ^{1}H and ^{13}C atoms in compound **S18**:

**xylose ring**	**proton**	**δ (ppm)**	**multiplicity**	***J* (Hz)**	**carbon**	**δ (ppm)**
A (reducing end)	H-1A	4.48	d	7.0	C-1A	101.60
	H-2A	4.93	dd	7.0, 8.5	C-2A	73.96
	H-3A	4.03			C-3A	78.88
	H-4A	3.55				
	H-5Aa	3.32				
	H-5Ab	3.92				
B	H-1B	4.81			C-1B	101.35
	H-2B	5.03			C-2B	72.95
	H-3B	3.52	t	8.1		
	H-4B	3.92				
	H-5Ba	3.09	dd	8.4, 12.0		
	H-5Bb	4.06				
C (non-reducing end)	H-1C	4.77			C-1C	101.46
	H-2C	5.01			C-2C	75.37
	H-3C	3.88			C-3C	75.05
	H-4C	3.56				
	H-5Ca	3.31				
	H-5Cb	3.99				

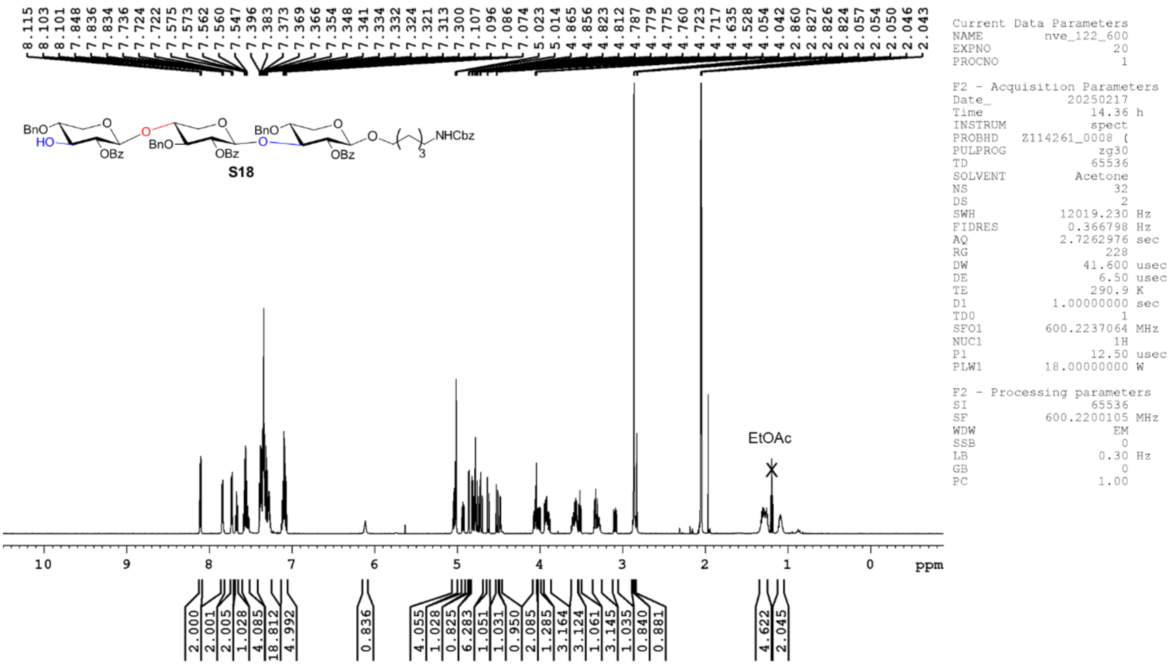

^{1}H NMR spectrum of compound **S18** (600 MHz, $(CD_3)_2CO$)

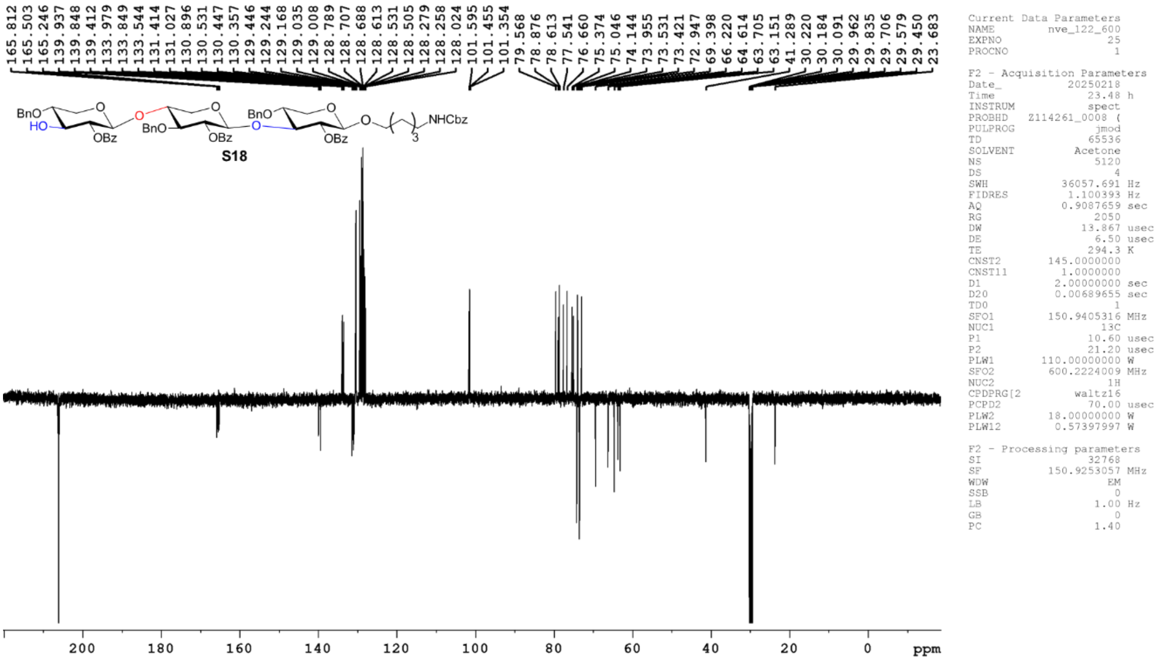

^{13}C APT NMR spectrum of compound **S18** (151 MHz, $(CD_3)_2CO$)

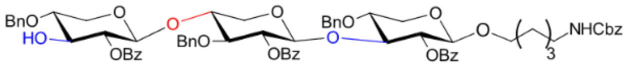

S18

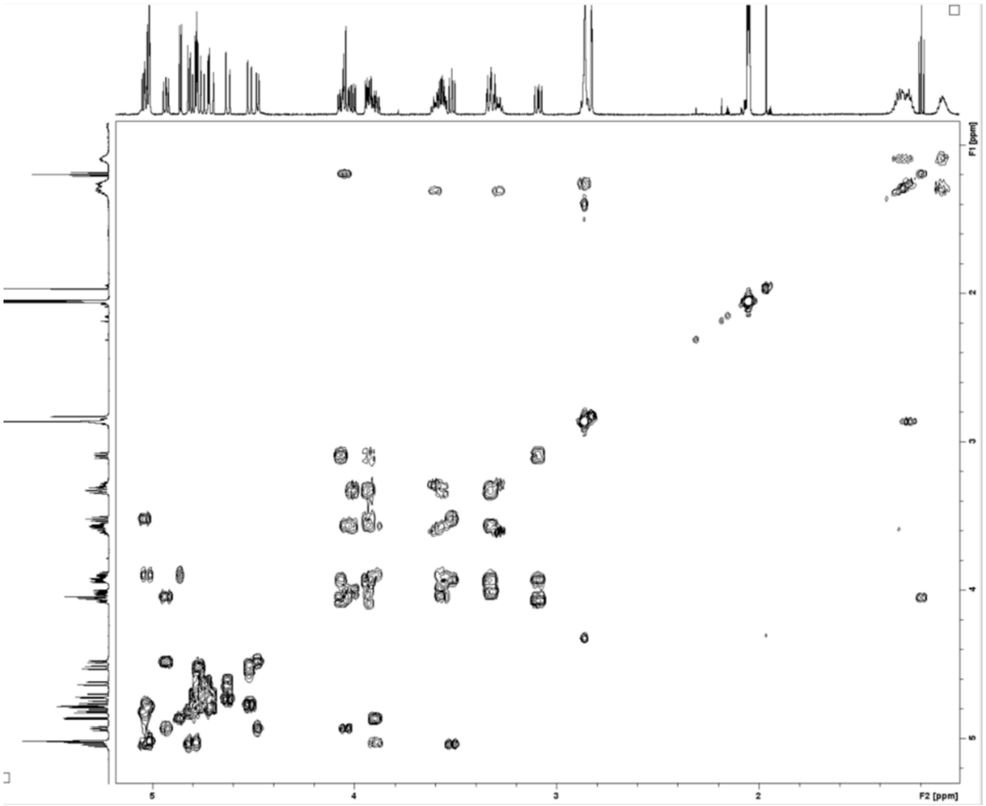

^{1}H-^{1}H COSY NMR spectrum of compound **S18** (600 MHz, $(CD_3)_2CO$)

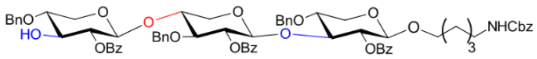

S18

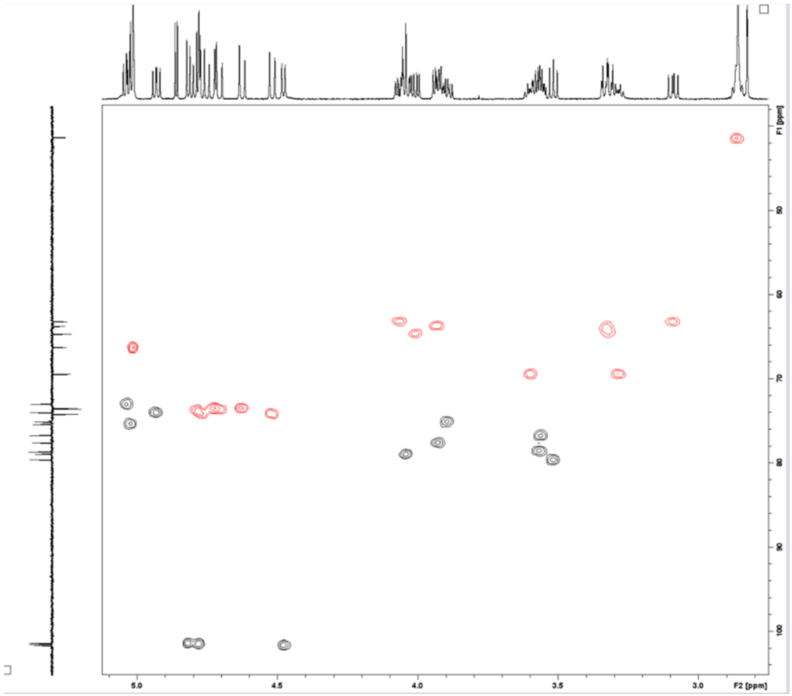

^{1}H-^{13}C HSQC NMR spectrum of compound **S18** (600/151 MHz, $(CD_3)_2CO$)

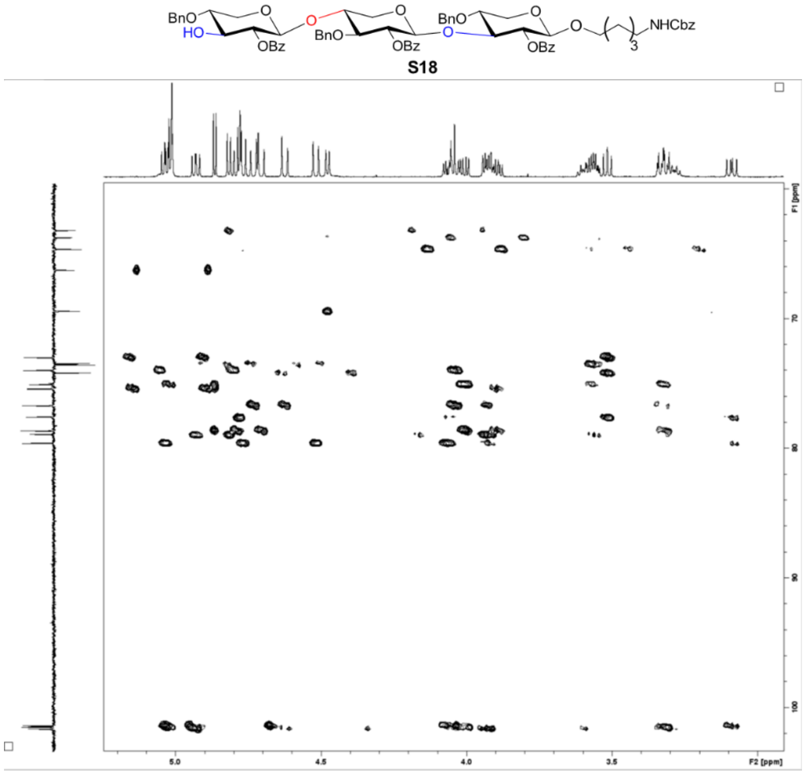

^{1}H-^{13}C HMBC NMR spectrum of compound **S18** (600/151 MHz, $(CD_3)_2CO$)

Aminopentyl β-D-xylopyranosyl-(1→4)-β-D-xylopyranosyl-(1→3)-β-D-xylopyranoside (17)

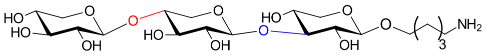

17

Experimental procedure: To a solution of protected MLX trisaccharide **S18** (6.9 mg, 5.67 µmol) in THF (114 µL, 50 mM), a solution of NaOMe in MeOH (0.5 M, 85 µL, 43 µmol) was added at rt, and the reaction mixture was allowed to stir overnight. After confirmation of reaction completion (22 h) by TLC (R_f: 0.32 (MeOH/$CHCl_3$ = 1/15, v/v)), the reaction mixture was neutralized by the addition of IR-120 H^+ resin while slowly stirring the mixture. Then, the reaction mixture was filtered, and the filtrate was concentrated under reduced pressure to yield a partially-deprotected glassy crude, which was kept under high vacuum until usage in the next step without any further purification. To a solution of partially deprotected crude (5.67 µmol) in *t*-BuOH (0.83 mL), H_2O (0.42 mL), and AcOH (4 drops), unreduced 10% Pd/C (7.0 mg) was added and the reaction mixture was stirred in the H_2 reactor under a pressure of 8 bar H_2. After 24 h, the reaction mixture was filtered using a PTFE syringe filter (0.45 µm) and concentrated under reduced pressure to yield a crude product, which was purified using HILIC-HPLC column chromatography (13 mM NH_4OAc/ACN = 1/4 to 1/1, v/v). The purified product was lyophilized to give MLX trisaccharide **17** (2.83 mg, 69% yield over 2 steps) as a white amorphous solid.

^{1}H NMR (600 MHz, D_2O): δ 4.67 (d, J = 7.7 Hz, 1H, H-1B), 4.44 (d, J = 7.8 Hz, 1H, H-1C), 4.41 (d, J = 7.9 Hz, 1H, H-1A), 4.09 (dd, J = 5.3, 11.8 Hz, 1H, H-5B), 3.99-3.93 (m, 2H, H-5A, H-5C), 3.90-3.83 (m, 1H, OCH_2), 3.81-3.75 (m, 1H, H-4B), 3.70-3.54 (m, 5H, H-4A, OCH_2, H-3A, H-4C, H-3B), 3.45-3.26 (m, 6H, H-2A, H-3C, H-5B, H-2B, H-5A, H-5C), 3.26-3.22 (m, 1H, H-2C), 2.98 (t, J = 7.5 Hz, 2H, CH_2-NH_2), 1.71-1.61 (m, 4H, CH_2), 1.48-1.39 (m, 2H, CH_2) ppm.

^{13}C NMR (151 MHz, D_2O): δ 103.89, 103.28, 102.4, 84.4, 77.0, 76.2, 74.3, 73.87, 73.36, 73.28, 70.8, 69.8, 68.3, 65.81, 65.37, 63.5, 40.0, 28.8, 27.0, 22.7 ppm.

ESI-HRMS: m/z $[M + H]^+$ calcd. for $C_{20}H_{38}NO_{13}$: 500.2338; found 500.2345.

NMR chemical shifts of selected ^{1}H and ^{13}C atoms in compound **17**:

**xylose ring**	**proton**	***δ* (ppm)**	**multiplicity**	***J* (Hz)**	**carbon**	***δ* (ppm)**
A (reducing end)	H-1A	4.41	d	7.9	C-1A	103.28
	H-2A	3.42				
	H-3A	3.63			C-3A	84.4
	H-4A	3.66			C-4A	68.3
	H-5Aa	3.31				
	H-5Ab	3.95				
B	H-1B	4.67	d	7.7	C-1B	103.89
	H-2B	3.36				
	H-3B	3.56			C-3B	74.3
	H-4B	3.77			C-4B	77.0
	H-5Ba	3.36			C-5B	63.5
	H-5Bb	4.09	dd	5.3, 11.8		
C (non-reducing end)	H-1C	4.44	d	7.8	C-1C	102.4
	H-2C	3.24				
	H-3C	3.41				
	H-4C	3.61				
	H-5Ca	3.29				
	H-5Cb	3.95				

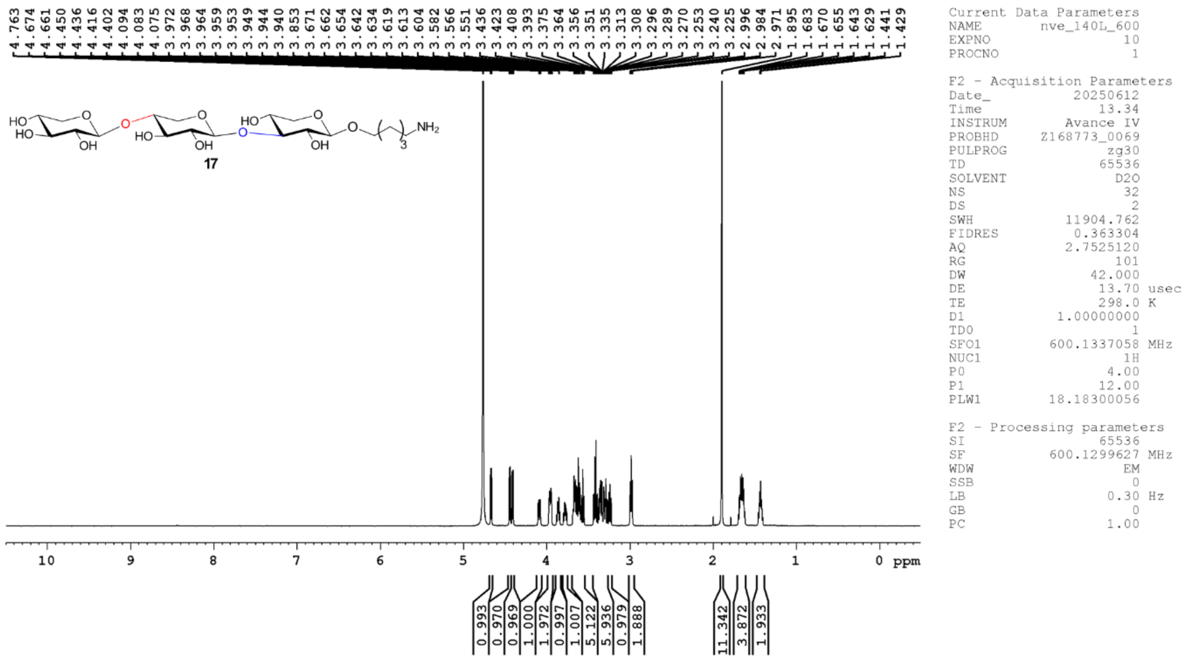

^{1}H NMR spectrum of compound **17** (600 MHz, D_2O)

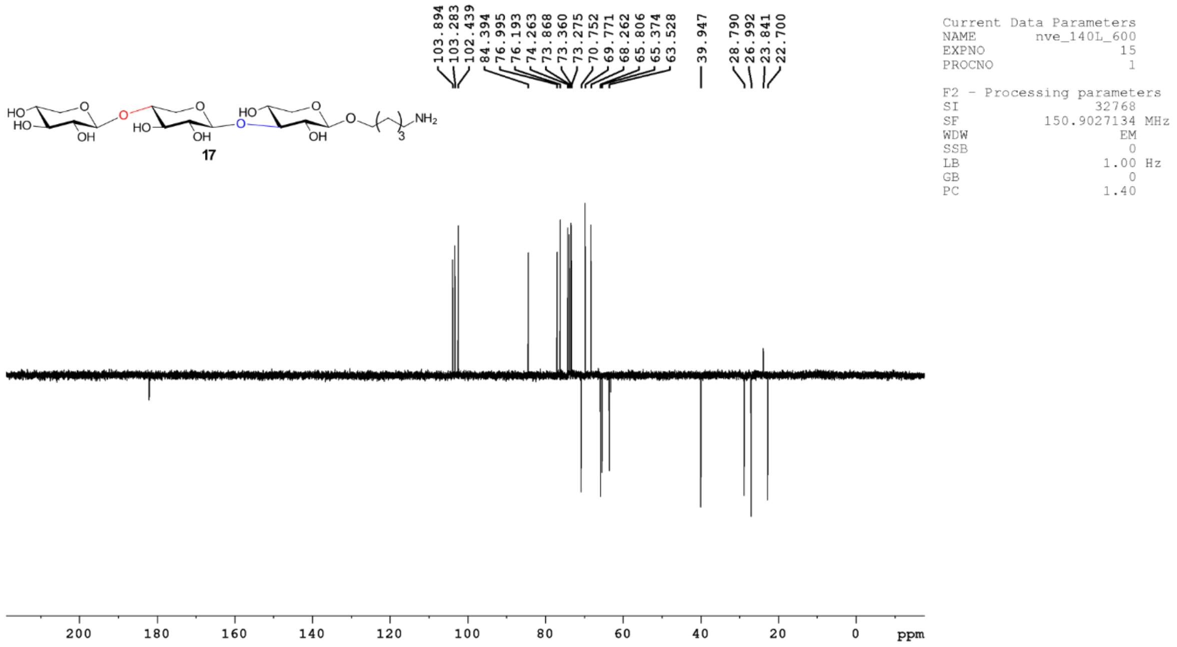

^{13}C APT NMR spectrum of compound **17** (151 MHz, D_2O)

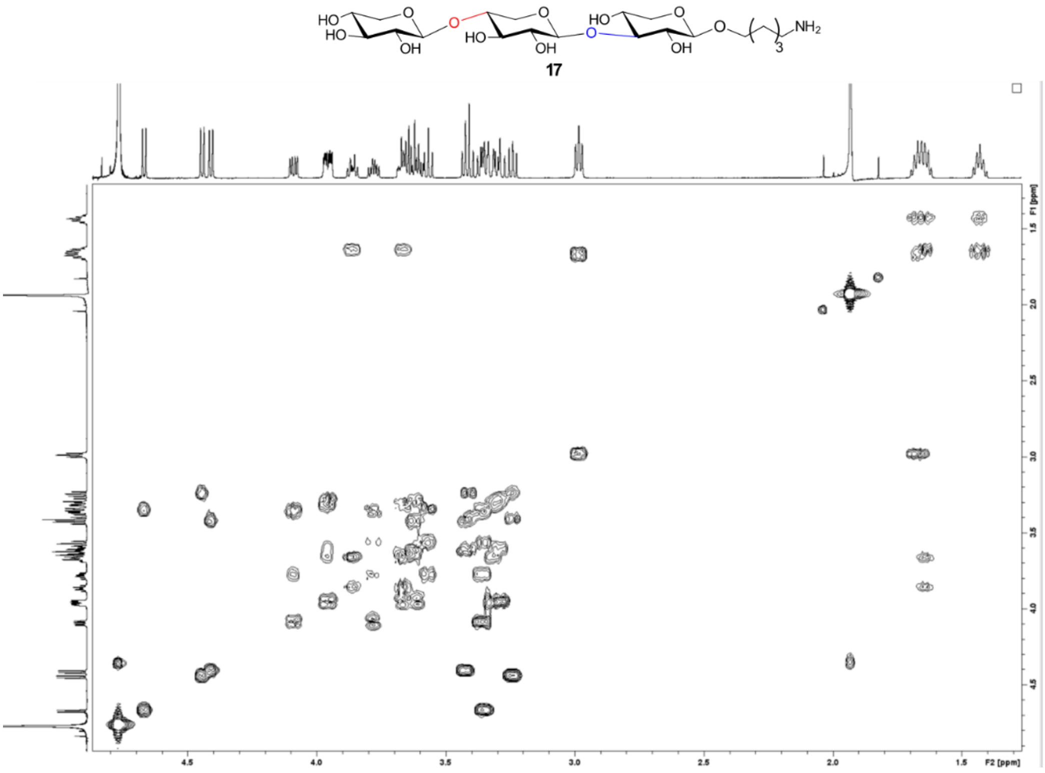

^1H-^1H COSY NMR spectrum of compound **17** (600 MHz, D_2O)

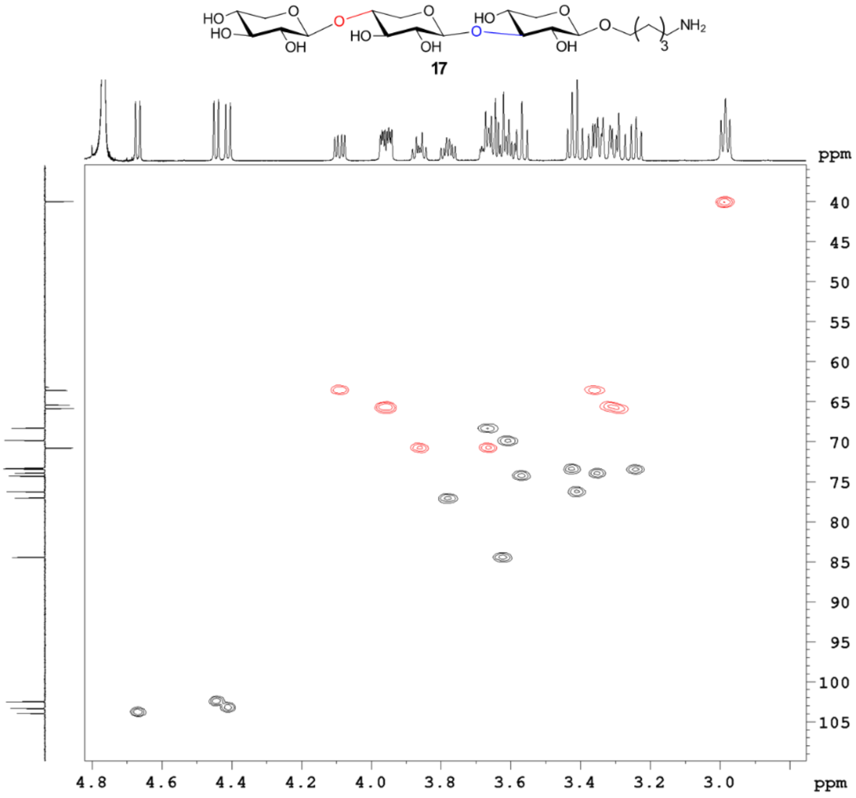

^1H-^{13}C HSQC NMR spectrum of compound **17** (600/151 MHz, D_2O)

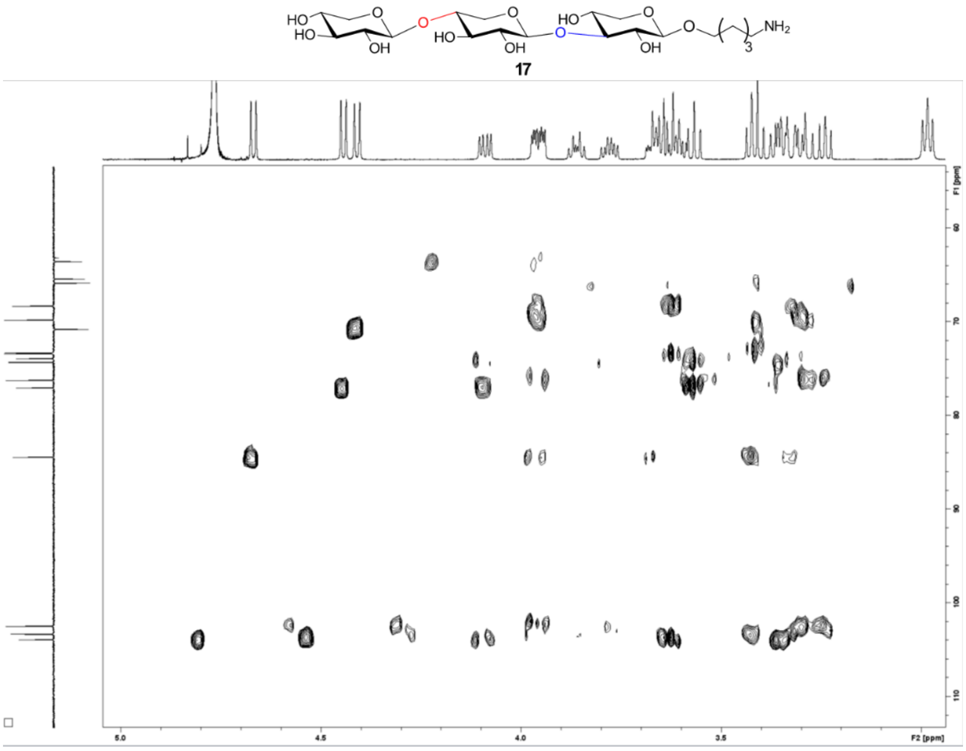

^{1}H-^{13}C HMBC NMR spectrum of compound **17** (600/151 MHz, D_2O)

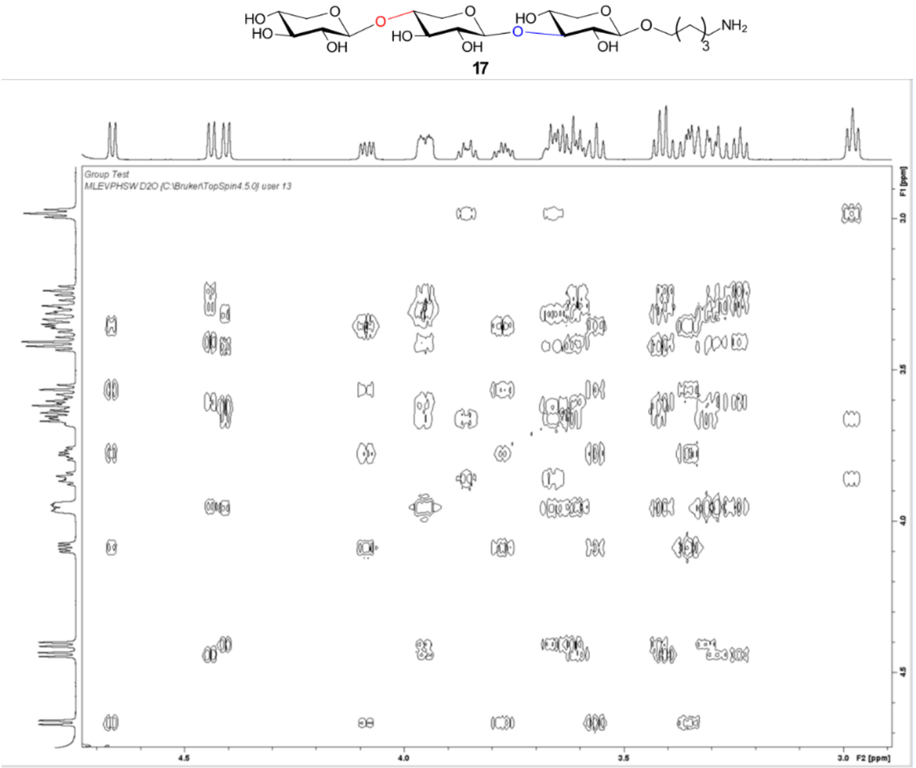

^{1}H-^{1}H TOCSY NMR spectrum of compound **17** (600 MHz, D_2O)

AGA of MLX tetrasaccharide S19

Benzyloxycarbonylaminopentyl 2-*O*-benzoyl-3-*O*-benzyl-β-D-xylopyranosyl-(1→4)-2-*O*-benzoyl-3-*O*-benzyl-β-D-xylopyranosyl-(1→3)-2-*O*-benzoyl-4-*O*-benzyl-β-D-xylopyranosyl-(1→4)-2-*O*-benzoyl-3-*O*-benzyl-β-D-xylopyranoside (**S19**)

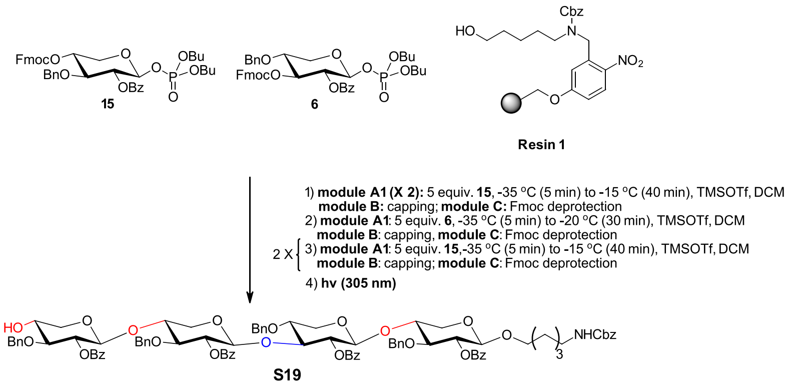

Experimental procedure: Linker-functionalized resin **1** (38 mg, 12.5 µmol) was placed in the synthesizer and synthesizer modules were applied as follows:

1) two cycles of module A1 (**15**, 47 mg, 0.0625 mmol, 5 equiv.) at –35 °C (5 min) to –15 °C (40 min), module B, and module C.
2) module A1 (**6**, 47 mg, 0.0625 mmol, 5 equiv.) at –35 °C (5 min) to –20 °C (30 min), module B, and module C.
3) 2 × [module A1 (**15**, 47 mg, 0.0625 mmol, 5 equiv.) at –35 °C (5 min) to –15 °C (40 min), module B, and module C].

Cleavage from the resin using UV irradiation at 305 nm in a continuous flow photoreactor afforded the crude product. Purification of the crude by normal phase HPLC using a preparative YMC-Small column (EtOAc/hexanes = 1/9 to 1/1.5 and then toluene/ACN = 1/4, v/v) gave protected MLX tetrasaccharide **S19** (3.9 mg, 20% yield over 9 steps) as a glassy solid.

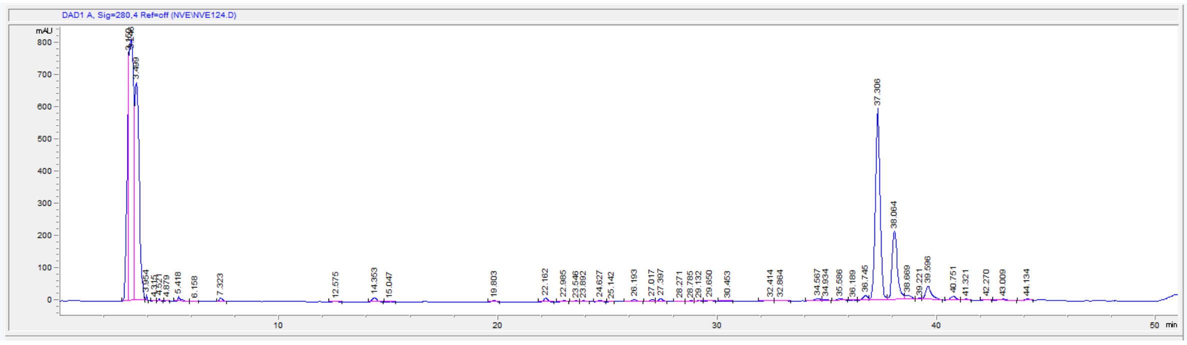

Analytical HPLC of the crude reaction mixture was performed on a YMC-Small NP column using a gradient of EtOAc in hexanes (50 min, flow rate 1 mL/min). MLX tetrasaccharide **S19** was eluted at 37.3 min.

^{1}H NMR (600 MHz, $(CD_3)_2CO$): *δ* 8.12-8.08 (m, 2H, Ar-H), 8.06-8.02 (m, 2H, Ar-H), 7.99-7.96 (m, 2H, Ar-H), 7.92-7.88 (m, 2H, Ar-H), 7.71-7.65 (m, 2H, Ar-H), 7.65-7.59 (m, 2H, Ar-H), 7.58-7.50 (m, 6H, Ar-H), 7.42-7.37 (m, 2H, Ar-H), 7.35-7.33 (m, 3H, Ar-H), 7.33-7.30 (m, 2H, Ar-H), 7.30-7.27 (m, 2H, Ar-H), 7.27-7.26 (m, 1H, Ar-H), 7.25-7.22 (m, 2H, Ar-H), 7.20-7.16 (m, 5H, Ar-H), 7.15-7.12 (m, 6H, Ar-H), 7.11-7.10 (m, 2H, Ar-H), 7.10-7.09 (m, 1H, Ar-H), 7.08-7.06 (m, 1H, Ar-H), 6.14 (br, 1H, NH), 5.15-5.08 (m, 2H, H-2D, H-2C), 5.02 (s, 2H, CH_2Cbz), 4.96-4.92 (m, 2H, H-2A, H-2B), 4.89-4.83 (m, 4H, H-1C, H-1D, CH_2-Ph), 4.82 (d, *J* = 4.7 Hz, 1H, CH_2Ph), 4.79 (d, *J* = 4.7 Hz, 1H, H-1B), 4.72-4.68 (m, 2H, CH_2-Ph, O-H), 4.63-4.55 (m, 4H, CH_2-Ph), 4.22 (d, *J* = 7.8 Hz, 1H, H-1A), 4.11-4.02 (m, 3H, H-5C, H-3B, H-5B), 4.01-3.97 (m, 1H, H-4C), 3.93 (dd, *J* = 5.4, 11.5 Hz, 1H, H-5D), 3.89-3.81 (m, 2H, H-4D, H-5A), 3.81-3.75 (m, 1H, H-4A), 3.70 (t, *J* = 8.9 Hz, 1H, H-3D), 3.67-3.62 (m, 1H, OCH_2), 3.59 (t, *J* = 8.5 Hz, 1H, H-3C), 3.56-3.52 (m, 1H, H-4B), 3.45 (t, *J* = 9.0 Hz, 1H, H-3A), 3.40-3.33 (m, 3H, H-5B, H-5D, OCH_2), 3.13 (dd, *J* = 9.1, 11.8 Hz, 1H, H-5C), 2.93-2.88 (m, 2H, CH_2-NHCbz), 2.59 (dd, *J* = 10.2, 11.3 Hz, 1H, H-5A), 1.44-1.37 (m, 2H, CH_2), 1.35-1.30 (m, 2H, CH_2), 1.22-1.14 (m, 2H, CH_2) ppm.

^{13}C NMR (151 MHz, $(CD_3)_2CO$): *δ* 165.71, 165.61, 165.58, 165.48, 139.73, 139.70, 139.65, 139.46, 134.10, 134.06, 133.96, 133.92, 131.32, 131.22, 130.80, 130.74, 130.48, 130.43, 130.35, 129.76, 129.53, 129.44, 129.40, 129.33, 129.16, 129.04, 128.99, 128.86, 128.8, 128.71, 128.62, 128.53, 128.43, 128.24, 128.05, 128.02, 127.96, 102.2, 101.90, 101.44, 98.6, 83.1, 80.69, 80.07, 77.63, 76.97, 75.89, 75.55, 75.21, 74.74, 74.37, 74.11, 74.0, 73.2, 72.56, 72.33, 71.2, 69.7, 66.80, 66.24, 63.54, 63.18, 61.5, 41.3, 29.8, 23.7 ppm.

ESI-HRMS: m/z $[M + HCOO]^-$ calcd. for $C_{90}H_{92}NO_{25}$: 1586.5964; found 1586.5926.

NMR chemical shifts of selected ^{1}H and ^{13}C atoms in compound **S19**:

xylose ring	proton	*δ* (ppm)	multiplicity	*J* (Hz)	carbon	*δ* (ppm)
A (reducing end)	H-1A	4.22	d	7.8	C-1A	102.2
	H-2A	4.93			C-2A	72.33
	H-3A	3.45	t	9.0	C-3A	80.69
	H-4A	3.81-3.75	m		C-4A	75.89
	H-5Aa	2.59	dd	10.2, 11.3	C-5A	63.18
	H-5Ab	3.82				
B	H-1B	4.79	d	4.7	C-1B	98.6
	H-2B	4.93			C-2B	74.0
	H-3B	4.07			C-3B	76.97
	H-4B	3.56-3.52	m		C-4B	75.55
	H-5Ba	3.36			C-5B	61.5
	H-5Bb	4.04				
C	H-1C	4.87			C-1C	101.90
	H-2C	5.08			C-2C	73.2
	H-3C	3.59	t	8.5	C-3C	80.07
	H-4C	4.01-3.97	m		C-4C	77.63
	H-5Ca	3.13	dd	9.1, 11.8	C-5C	63.54
	H-5Cb	4.08				
D (non-reducing end)	H-1D	4.83			C-1D	101.44
	H-2D	5.12				
	H-3D	3.70	t	8.9		
	H-4D	3.85				
	H-5Da	3.35				
	H-5Db	3.93	dd	5.4, 11.5		

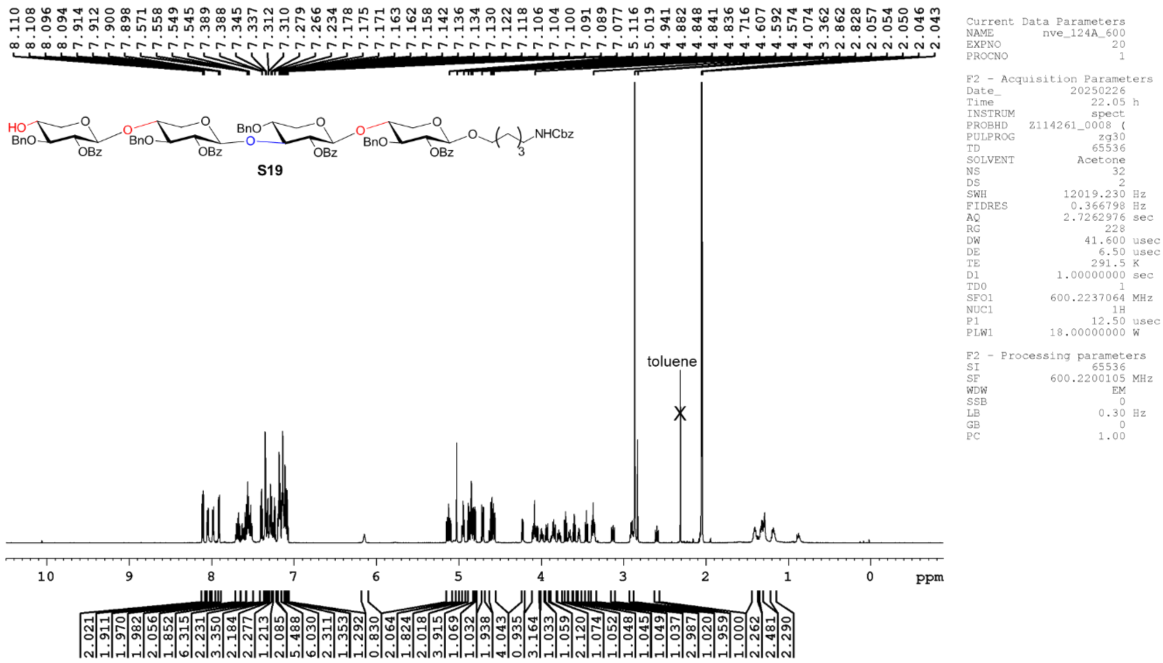

^{1}H NMR spectrum of compound **S19** (600 MHz, $(CD_3)_2CO$)

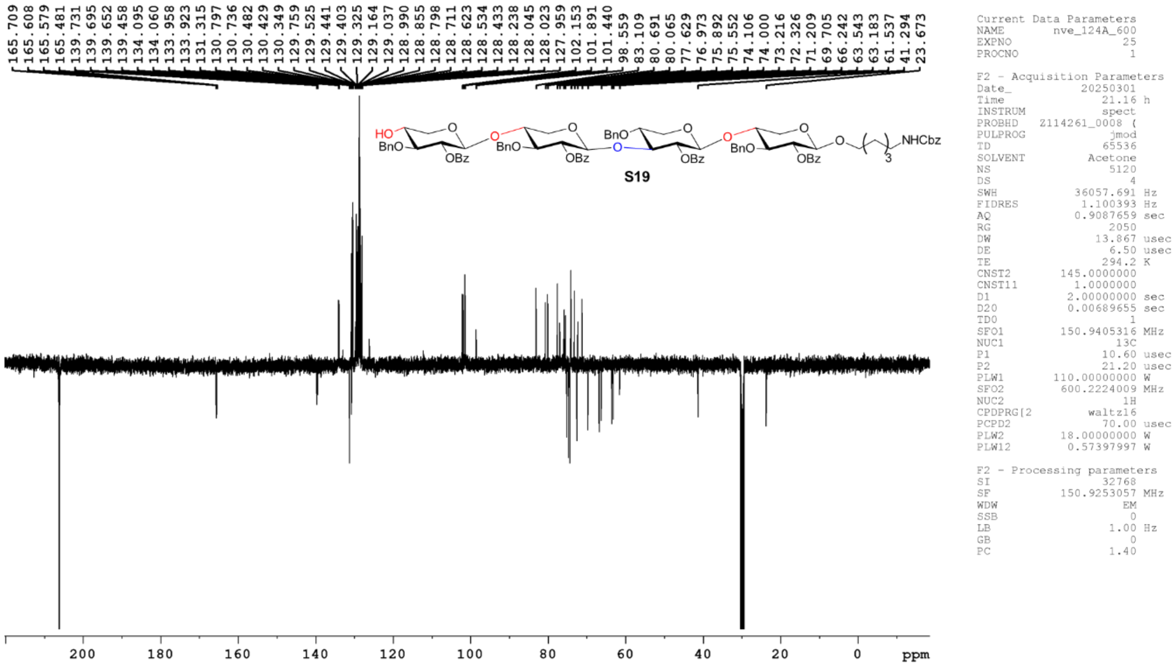

^{13}C APT NMR spectrum of compound **S19** (151 MHz, $(CD_3)_2CO$)

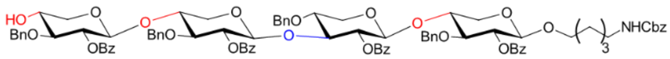

S19

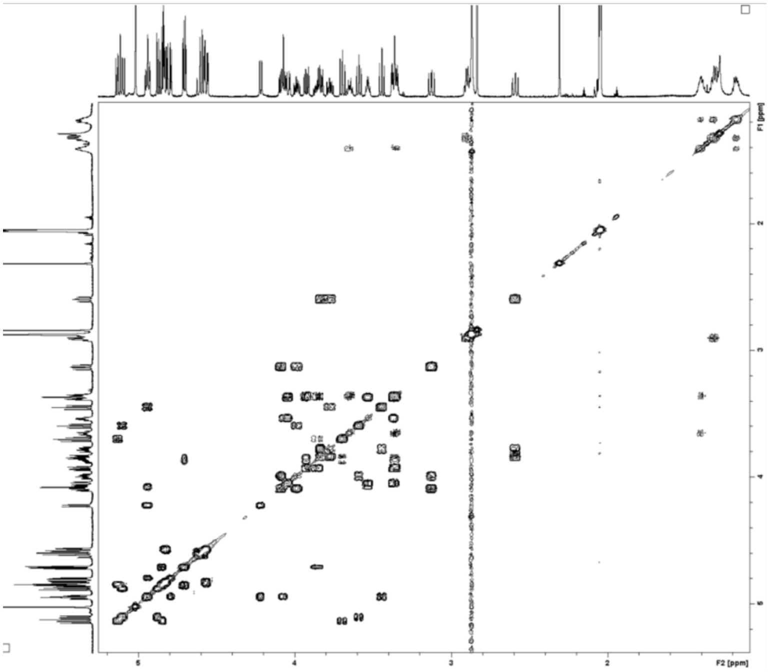

^{1}H-^{1}H COSY NMR spectrum of compound **S19** (600 MHz, $(CD_3)_2CO$)

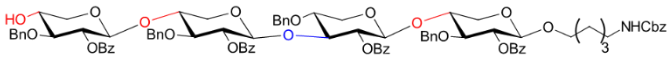

S19

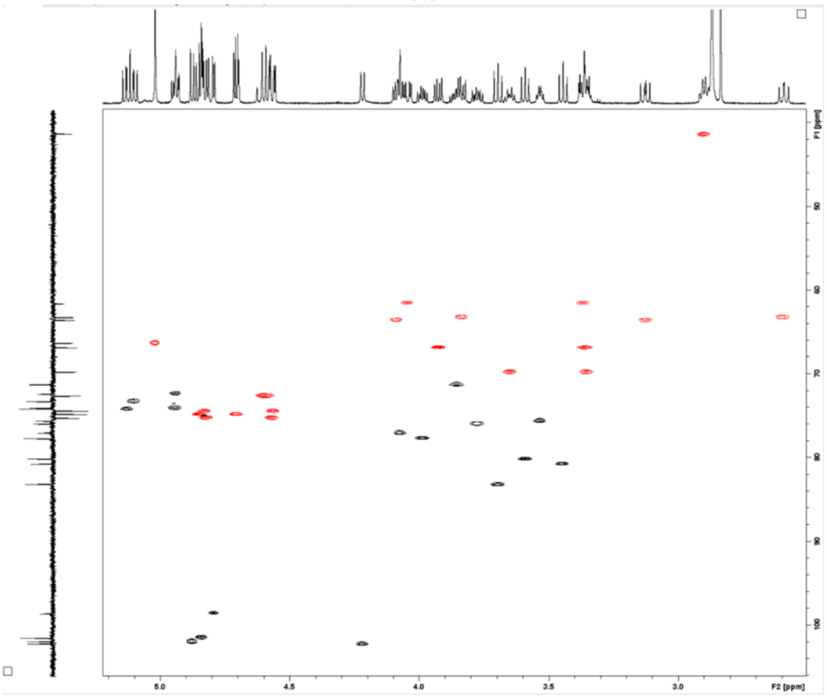

^{1}H-^{13}C HSQC NMR spectrum of compound **S19** (600/151 MHz, $(CD_3)_2CO$)

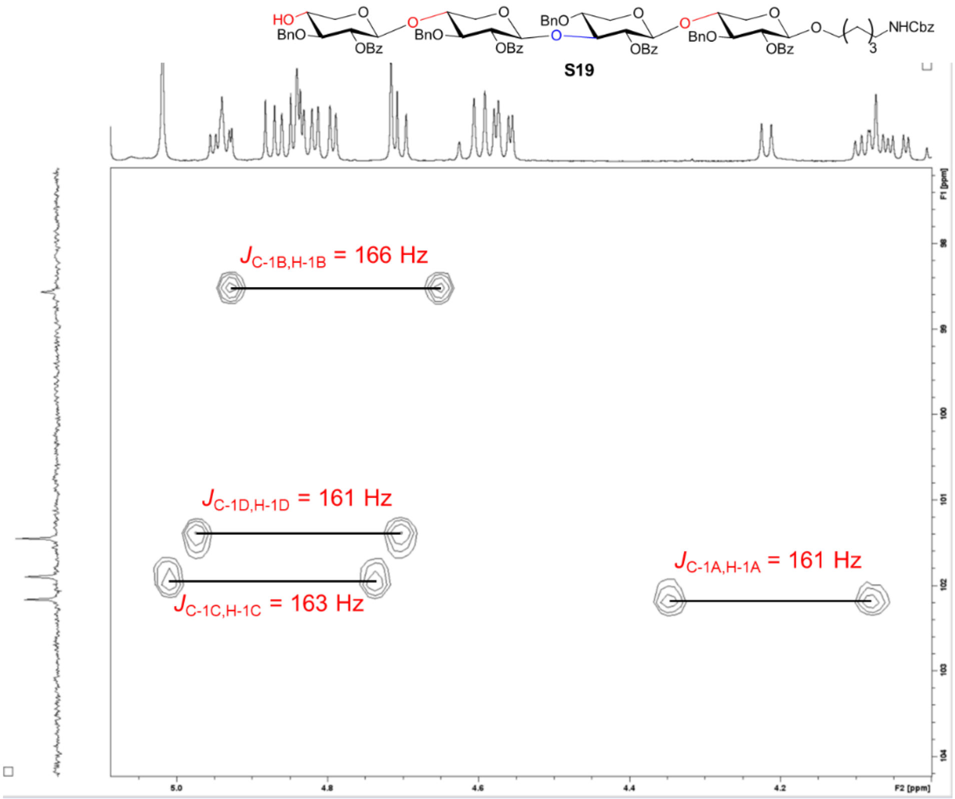

^{1}H-^{13}C CLIP HSQC NMR spectrum of compound **S19** (600/151 MHz, $(CD_3)_2CO$)

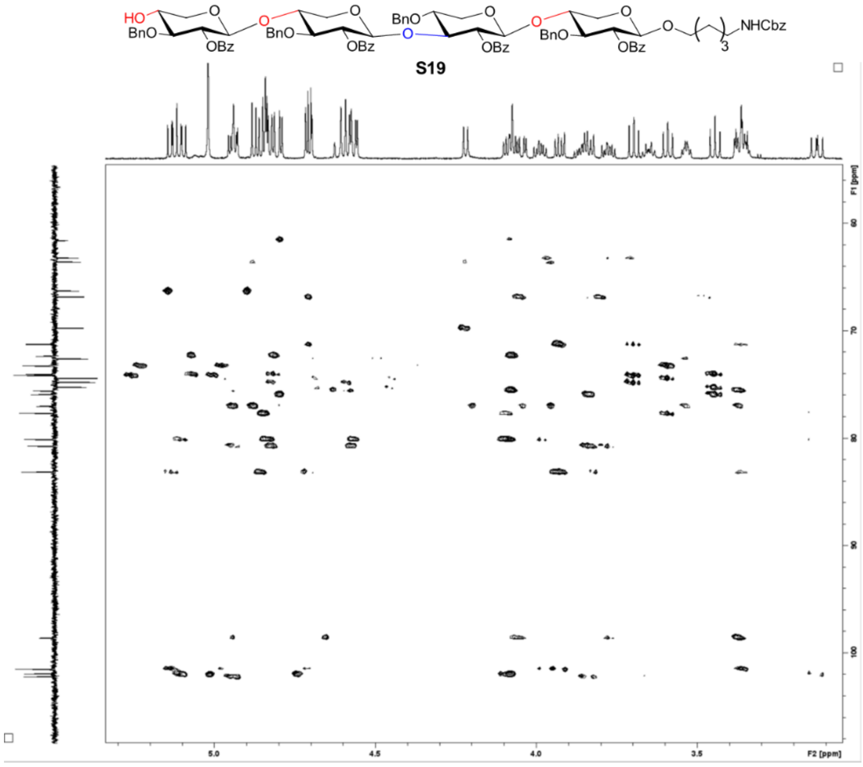

^{1}H-^{13}C HMBC NMR spectrum of compound **S19** (600/151 MHz, $(CD_3)_2CO$)

Aminopentyl β-D-xylopyranosyl-(1→4)-β-D-xylopyranosyl-(1→3)-β-D-xylopyranosyl-(1→4)-β-D-xylopyranoside (18)

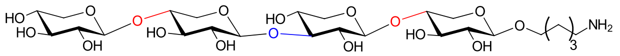

18

Experimental procedure: To a solution of protected MLX tetrasaccharide **S19** (3.9 mg, 2.53 μmol) in THF (51 μL, 50 mM), a solution of NaOMe in MeOH (0.5 M, 51 μL, 25 μmol) was added at rt, and the reaction mixture was allowed to stir overnight. After confirmation of reaction completion (20 h) by TLC (R_f: 0.41 (MeOH/$CHCl_3$ = 1/15, v/v)), the reaction mixture was neutralized by the addition of IR-120 H^+ resin while slowly stirring the mixture. Then, the reaction mixture was filtered, and the filtrate was concentrated under reduced pressure to yield a partially-deprotected glassy crude, which was kept under high vacuum until usage in the next step without any further purification. To a solution of partially deprotected crude (2.53 μmol) in *t*-BuOH (0.4 mL), H_2O (0.2 mL), and AcOH (3 drops), unreduced 10% Pd/C (3.8 mg) was added and the reaction mixture was stirred in the H_2 reactor under a pressure of 8 bar H_2. After 42 h, the reaction mixture was filtered using a PTFE syringe filter (0.45 μm) and concentrated under reduced pressure to yield a crude product, which was purified using HILIC-HPLC column chromatography (13 mM NH_4OAc/ACN = 1/4 to 1/1, v/v). The purified product was lyophilized to give MLX tetrasaccharide **18** (1.54 mg, 79% yield over 2 steps) as a white amorphous solid.

^{1}H NMR (600 MHz, D_2O): δ 4.67 (d, J = 7.7 Hz, 1H, H-1C), 4.48-4.43 (m, 2H, H-1B, H-1D), 4.40 (d, J = 7.9 Hz, 1H, H-1A), 4.12-4.03 (m, 2H, H-5C, H-5A), 4.02-3.93 (m, 2H, H-5B, H-5D), 3.89-3.83 (m, 1H, OCH_2), 3.81-3.72 (m, 2H, H-4C, H-4A), 3.71-3.64 (m, 2H, H-4B, OCH_2), 3.64-3.51 (m, 4H, H-3B, H-4D, H-3C, H-3A), 3.46-3.39 (m, 2H, H-2B, H-3D), 3.38-3.32 (m, 3H, H-5A, H-5C, H-2C), 3.32-3.22 (m, 4H, H-5B, H-5D, H-2A, H-2D), 2.97 (t, J = 7.5 Hz, 2H, CH_2-NH_2), 1.71-1.61 (m, 4H, CH_2), 1.47-1.39 (m, 2H, CH_2) ppm.

^{13}C NMR (151 MHz, D_2O): δ 103.82, 103.38, 102.44, 102.27, 84.0, 77.0, 76.2, 74.46, 74.25, 73.85, 73.56, 73.36, 73.10, 70.75, 69.77, 68.2, 65.81, 65.48, 63.5, 40.0, 28.8, 27.0, 22.7 ppm.

ESI-HRMS: m/z $[M + H]^+$ calcd. for $C_{25}H_{46}NO_{17}$: 632.2760; found 632.2758.

NMR chemical shifts of selected ^{1}H and ^{13}C atoms in compound **18**:

**xylose ring**	**proton**	***δ* (ppm)**	**multiplicity**	***J* (Hz)**	**carbon**	***δ* (ppm)**
A (reducing end)	H-1A	4.40	d	7.9	C-1A	103.38
	H-2A	3.24				
	H-3A	3.53				
	H-4A	3.75				
	H-5Aa	3.36				
	H-5Ab	4.05				
B	H-1B	4.46			C-1B	102.27
	H-2B	3.43				
	H-3B	3.61				
	H-4B	3.68				
	H-5Ba	3.30				
	H-5Bb	3.99				
C	H-1C	4.67	d	7.7	C-1C	103.82
	H-2C	3.33				
	H-3C	3.56				
	H-4C	3.77				
	H-5Ca	3.35				
	H-5Cb	4.09				
D (non-reducing end)	H-1D	4.44			C-1D	102.44
	H-2D	3.23				
	H-3D	3.41				
	H-4D	3.60				
	H-5Da	3.29				
	H-5Db	3.95				

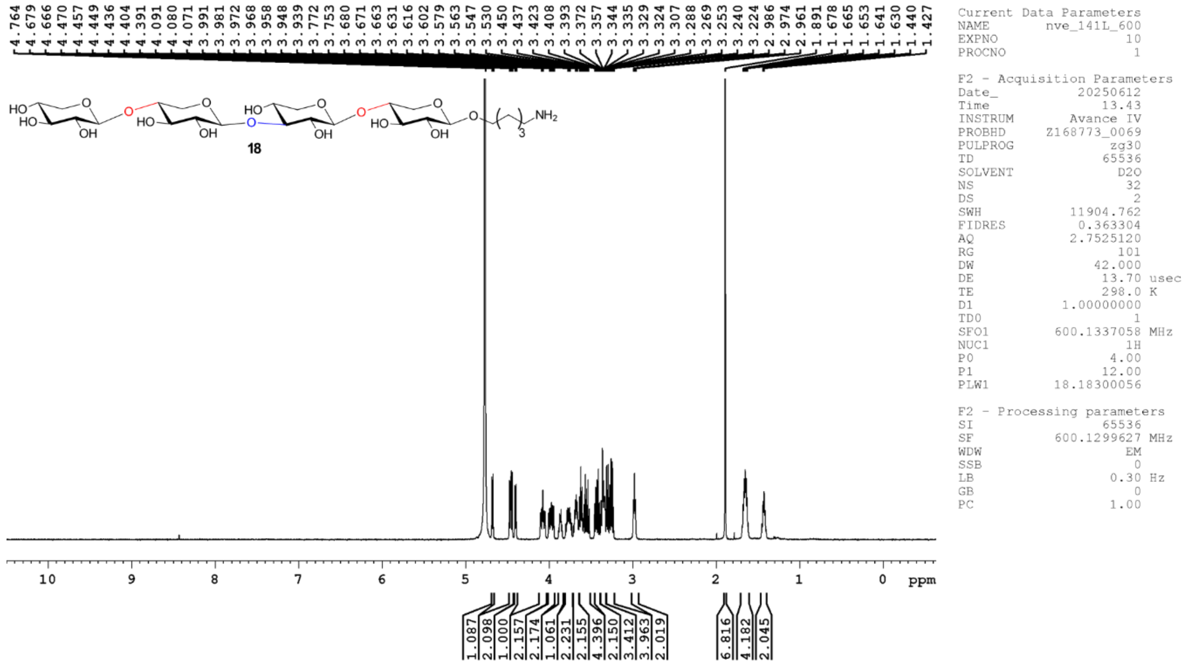

^{1}H NMR spectrum of compound **18** (600 MHz, D_2O)

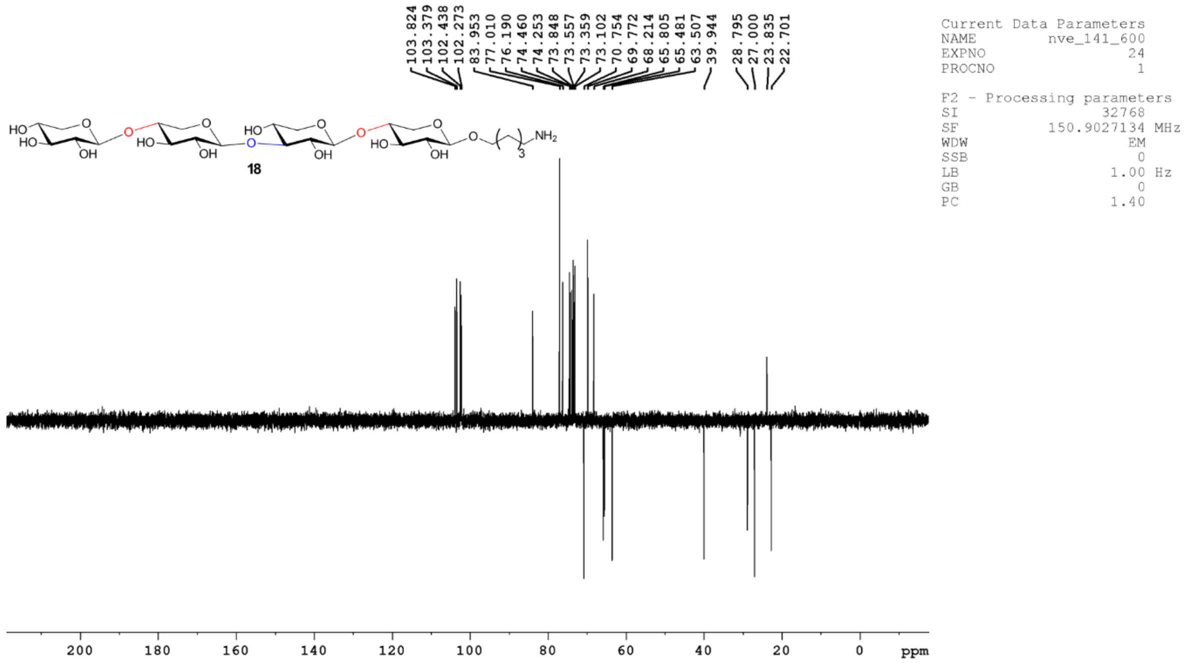

^{13}C APT NMR spectrum of compound **18** (151 MHz, D_2O)

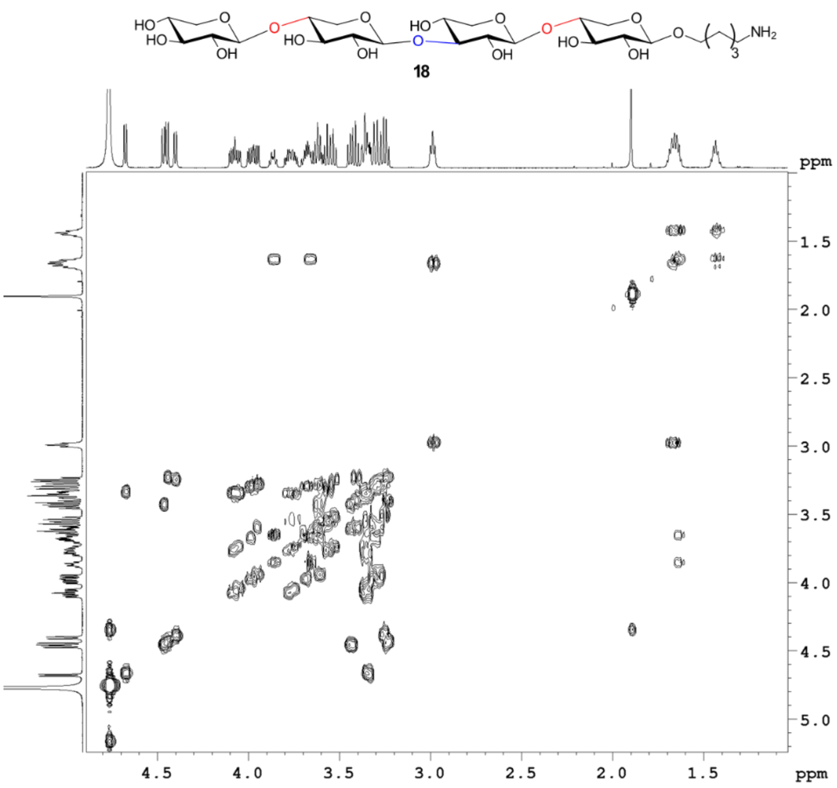

^{1}H-^{1}H COSY NMR spectrum of compound **18** (600 MHz, D_2O)

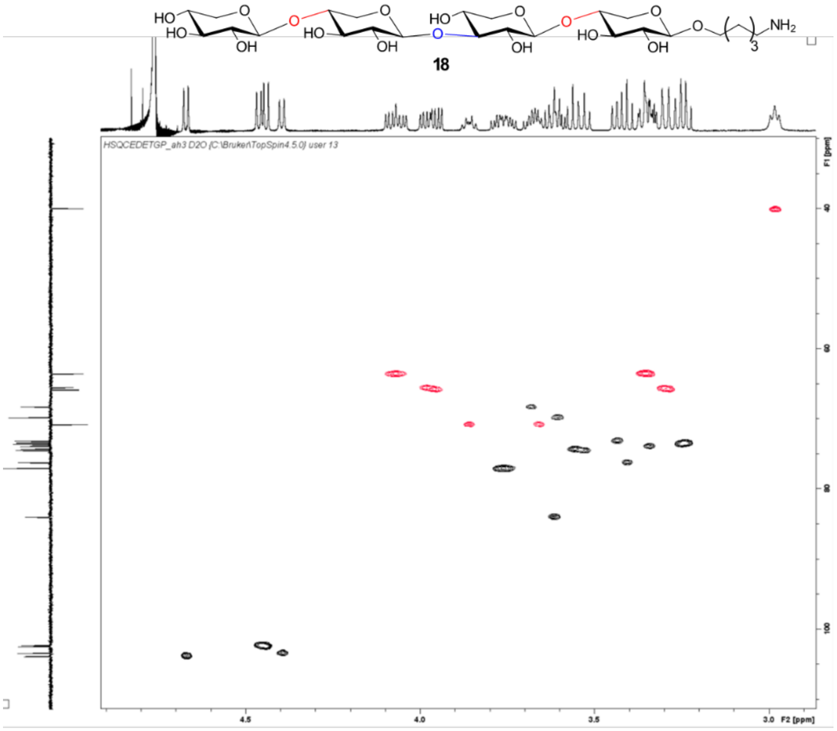

^{1}H-^{13}C HSQC NMR spectrum of compound **18** (600/151 MHz, D_2O)

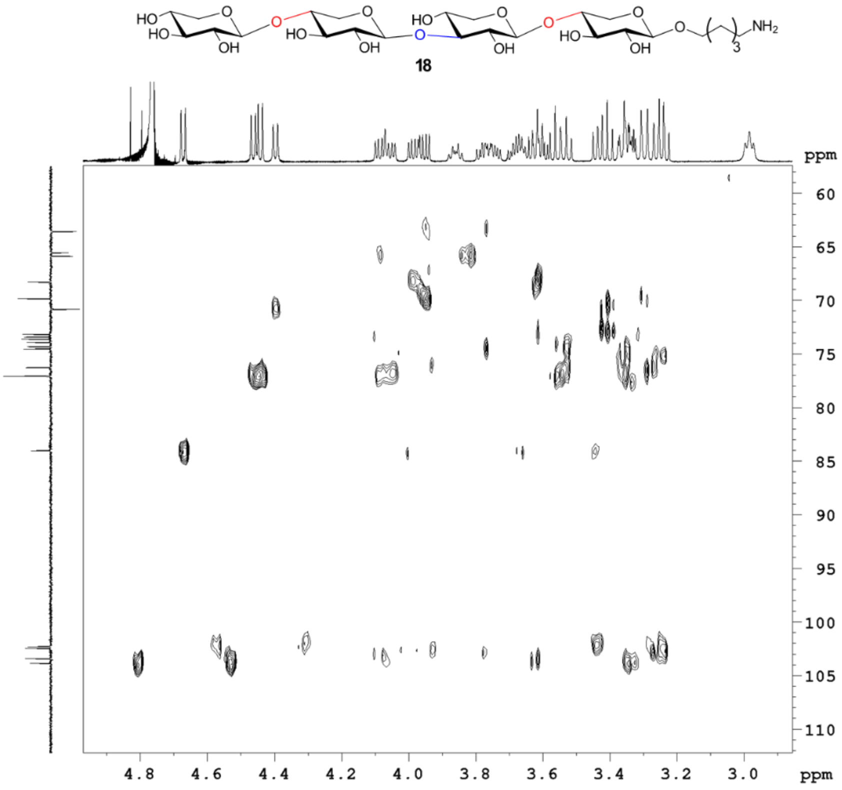

^{1}H-^{13}C HMBC NMR spectrum of compound **18** (600/151 MHz, D_2O)

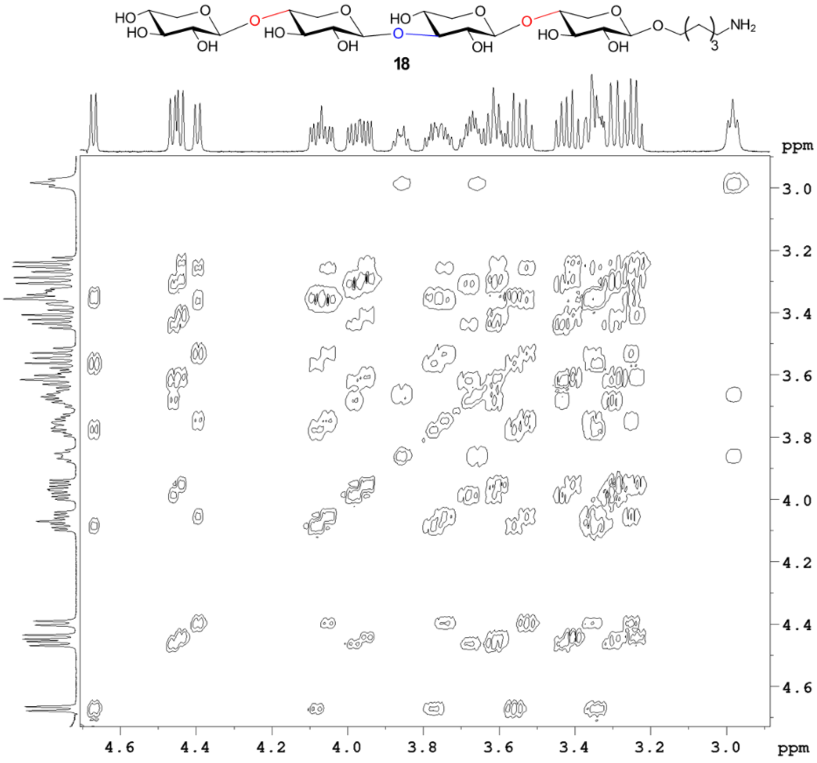

^{1}H-^{1}H TOCSY NMR spectrum of compound **18** (600 MHz, D_2O)

AGA of MLX hexasaccharide S20

Benzyloxycarbonylaminopentyl 2-*O*-benzoyl-4-*O*-benzyl-β-D-xylopyranosyl-(1→4)-2-*O*-benzoyl-3-*O*-benzyl-β-D-xylopyranosyl-(1→3)-2-*O*-benzoyl-4-*O*-benzyl-β-D-xylopyranosyl-(1→4)-2-*O*-benzoyl-3-*O*-benzyl-β-D-xylopyranosyl-(1→3)-2-*O*-benzoyl-4-*O*-benzyl-β-D-xylopyranosyl-(1→4)-2-*O*-benzoyl-3-*O*-benzyl-β-D-xylopyranoside (**S20**)

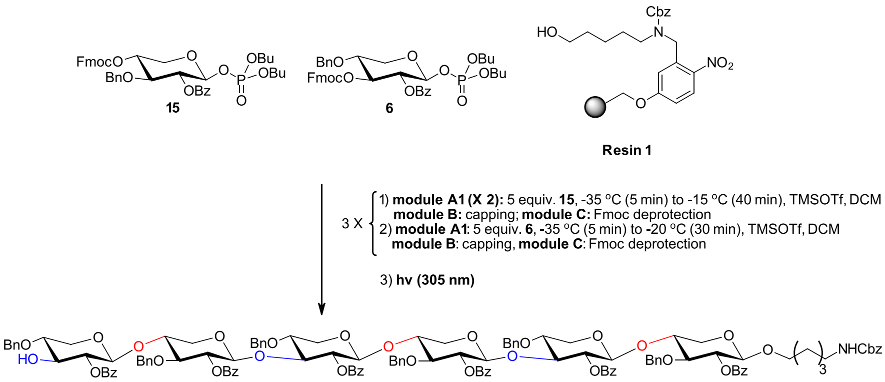

S20

Experimental procedure: Linker-functionalized resin **1** (38 mg, 12.5 µmol) was placed in the synthesizer and synthesizer modules were applied as follows:

3 × [

1) two cycles of module A1 (BB **15**, 47 mg, 0.0625 mmol, 5 equiv.) at −35 °C (5 min) to −15 °C (40 min), module B, and module C.
2) module A1 (BB **6**, 47 mg, 0.0625 mmol, 5 equiv.) at −35 °C (5 min) to −20 °C (30 min), module B, and module C
   ]

Cleavage from the resin using UV irradiation at 305 nm in a continuous flow photoreactor afforded the crude product. Purification of the crude by normal phase HPLC using a preparative YMC-Small column (EtOAc/hexanes = 1/9 to 1/1.5) and then using YMC-Diol column (EtOAc/hexanes = 1/9 to 1/1.5, v/v) gave protected MLX hexasaccharide **S20** (3.8 mg, 14% yield over 13 steps) as a glassy solid.

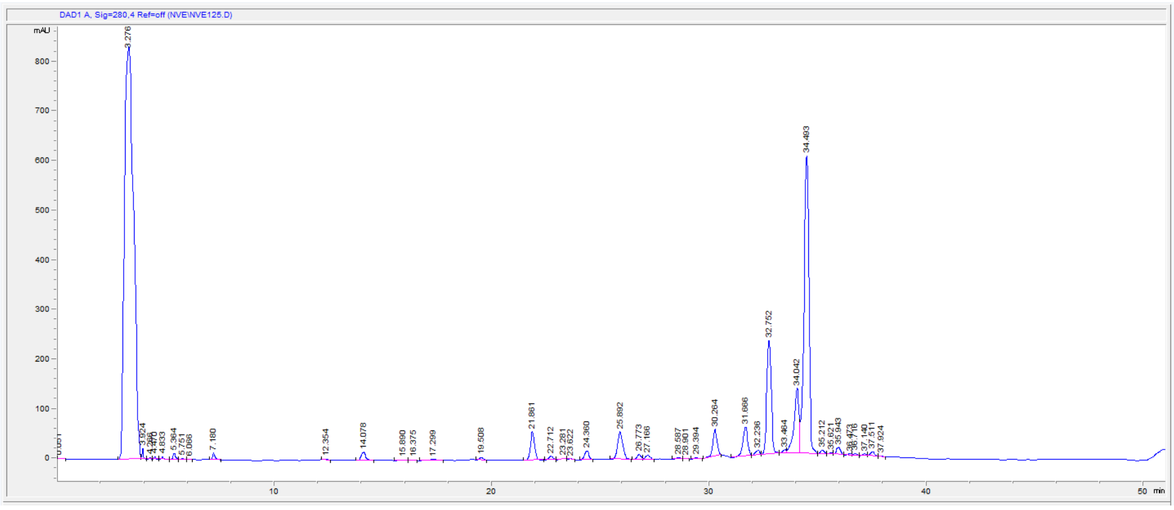

Analytical HPLC of the crude reaction mixture was performed on a YMC-Small NP column using a gradient of EtOAc in hexanes (50 min, flow rate 1 mL/min). MLX hexasaccharide **S20** was eluted at 34.5 min.

^{1}H NMR (600 MHz, $(CD_3)_2CO$): δ 8.13-8.09 (m, 2H, Ar-H), 8.08-8.03 (m, 4H, Ar-H), 7.99-7.95 (m, 2H, Ar-H), 7.95-7.89 (m, 4H, Ar-H), 7.72-7.68 (m, 1H, Ar-H), 7.68-7.60 (m, 4H, Ar-H), 7.60-7.50 (m, 9H, Ar-H), 7.45-7.41 (m, 2H, Ar-H), 7.40-7.36 (m, 4H, Ar-H), 7.36-7.32 (m, 6H, Ar-H), 7.31-7.22 (m, 12H, Ar-H), 7.18-7.15 (m, 2H, Ar-H), 7.15-7.11 (m, 5H, Ar-H), 7.10-7.05 (m, 8H, Ar-H), 6.12 (br, 1H, N-H), 5.10-5.01 (m, 5H, H-2E, H-2C, H-2F, CH_2-Ph), 4.98-4.91 (m, 3H, H-2A, H-2B, H-2D), 4.86 (d, J = 7.14 Hz, 1H, H-1E), 4.84-4.78 (m, 6H, O-H, H-1B, H-1F, CH_2-Ph), 4.77-4.74 (m, 2H, CH_2-Ph, H-1D), 4.73-4.68 (m, 2H, CH_2-Ph, H-1C), 4.62-4.54 (m, 6H, CH_2-Ph), 4.48 (d, J = 11.3 Hz, 1H, CH_2-Ph), 4.21 (d, J = 7.8 Hz, 1H, H-1A), 4.10-4.01 (m, 5H, H-5E, H-3D, H-3B, H-5B, H-5F), 4.0-3.94 (m, 2H, H-5D, H-4E), 3.93-3.84 (m, 3H, H-3F, H-5C, H-5A), 3.81-3.75 (m, 2H, H-4C, H-4A), 3.69-3.64 (m, 1H, OCH_2), 3.62-3.56 (m, 2H, H-4F, H-3E), 3.53-3.48 (m, 2H, H-4D, H-4B), 3.44 (t, J = 9.1 Hz, 1H, H-3A), 3.41-3.31 (m, 5H, H-3C, H-5B, H-5F, H-5D, OCH_2), 3.12 (dd, J = 9.0, 11.9 Hz, 1H, H-5E), 2.95-2.89 (m, 2H, CH_2-NHCbz), 2.70 (dd, J = 9.6, 11.7 Hz, 1H, H-5C), 2.58 (dd, J = 10.4, 11.4 Hz, 1H, H-5A), 1.47-1.38 (m, 2H, CH_2), 1.37-1.31 (m, 2H, CH_2), 1.24-1.15 (m, 2H, CH_2) ppm.

^{13}C NMR (151 MHz, $(CD_3)_2CO$): δ 165.84, 165.70, 165.64, 165.62, 165.57, 165.46, 139.95, 139.69, 139.65, 139.62, 139.52, 139.46, 134.14, 134.07, 134.04, 133.98, 131.41, 131.37, 131.24, 130.80, 130.75, 130.55, 130.49, 130.45, 130.35, 129.55, 129.47, 129.45, 129.40, 129.34, 129.18, 129.05, 128.99, 128.86, 128.74, 128.71, 128.63, 128.61, 128.55, 128.52, 128.29, 128.24, 128.22, 128.04, 127.96, 102.21, 102.11, 101.86, 101.32, 98.69, 98.19, 80.84, 80.56, 79.98, 78.64, 78.61, 77.52, 77.14, 76.68, 75.83, 75.60, 75.58, 75.42, 75.41, 75.38, 75.36, 75.13, 75.02, 74.91, 74.33, 74.15, 73.76, 73.56, 73.15, 72.64, 72.46, 72.41, 71.9, 69.8, 66.3, 64.7, 63.52, 63.2, 61.71, 60.95, 41.3, 23.7 ppm.

ESI-HRMS: m/z $[M + HCOO]^-$ calcd. for $C_{128}H_{128}NO_{35}$: 2238.8272; found 2238.8222.

NMR chemical shifts of selected ^{1}H and ^{13}C atoms in compound **S20**:

**xylose ring**	**proton**	***δ* (ppm)**	**multiplicity**	***J* (Hz)**	**carbon**	***δ* (ppm)**
A (reducing end)	H-1A	4.21	d	7.8	C-1A	102.21
	H-2A	4.95				
	H-3A	3.44	t	9.1	C-3A	80.84
	H-4A	3.77				
	H-5Aa	2.58	dd	10.4, 11.4		
	H-5Ab	3.85				
B	H-1B	4.83			C-1B	98.19
	H-2B	4.95				
	H-3B	4.04			C-3B	76.68
	H-4B	3.50			C-4B	75.41
	H-5Ba	3.37			C-5B	61.71
	H-5Bb	4.03				
C	H-1C	4.69			C-1C	102.11
	H-2C	5.05			C-2C	73.76
	H-3C	3.39			C-3C	80.56
	H-4C	3.79				
	H-5Ca	2.70	dd	9.6, 11.7	C-5C	63.2
	H-5Cb	3.89				
D	H-1D	4.74			C-1D	98.69
	H-2D	4.92			C-2D	72.46
	H-3D	4.06				
	H-4D	3.50				
	H-5Da	3.33			C-5D	61.71
	H-5Db	3.98				
E	H-1E	4.86	d	7.14	C-1E	101.86
	H-2E	5.07			C-2E	73.15
	H-3E	3.58			C-3E	79.98
	H-4E	3.96			C-4E	77.52
	H-5Ea	3.12	dd	9.0, 11.9	C-5E	63.52
	H-5Eb	4.08				
F (non-reducing end)	H-1F	4.80			C-1F	101.32
	H-2F	5.04				
	H-3F	3.91			C-3F	74.91
	H-4F	3.60			C-4F	78.61
	H-5Fa	3.34			C-5F	64.7
	H-5Fb	4.03				

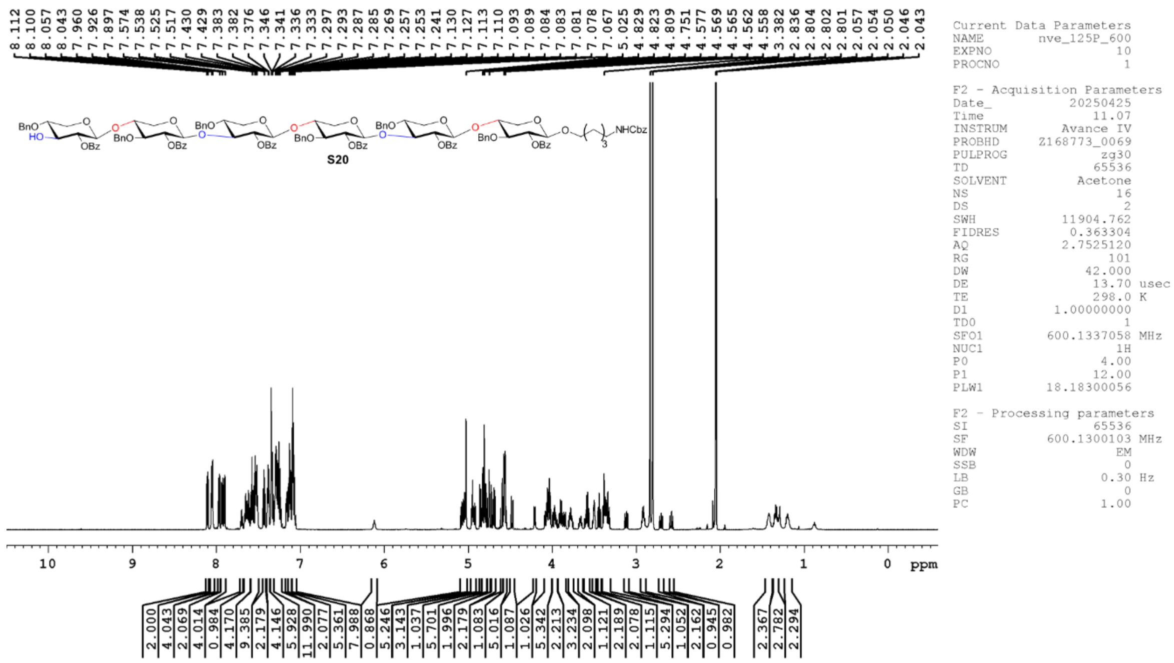

^{1}H NMR spectrum of compound **S20** (600 MHz, $(CD_3)_2CO$)

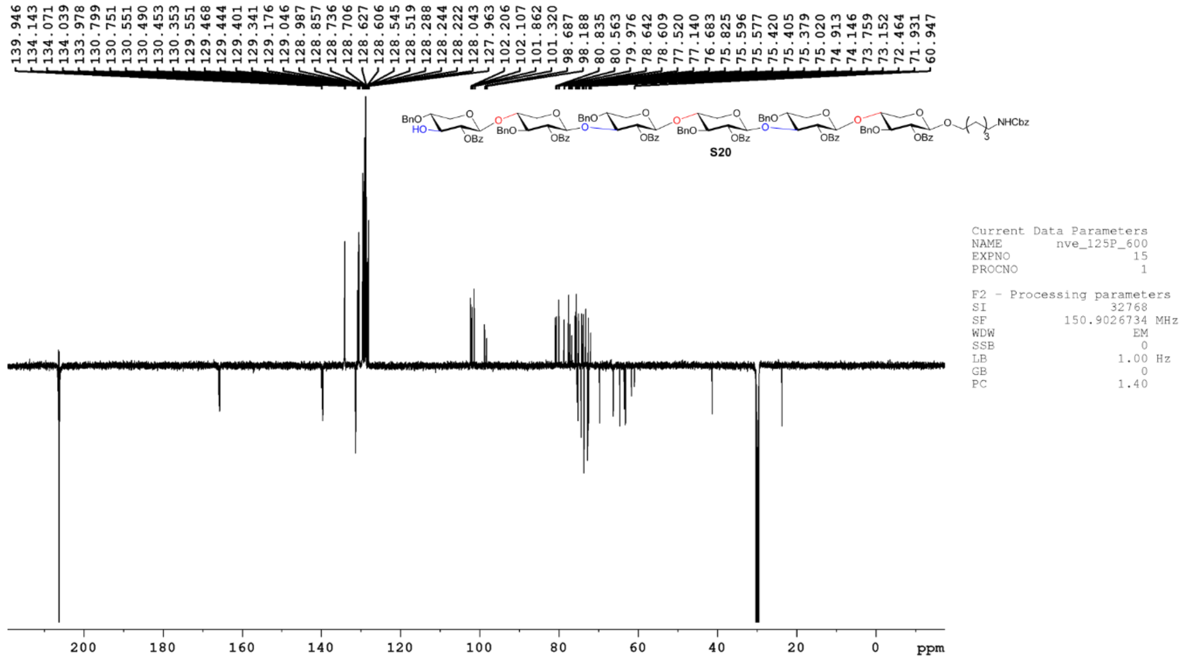

^{13}C APT NMR spectrum of compound **S20** (151 MHz, $(CD_3)_2CO$)

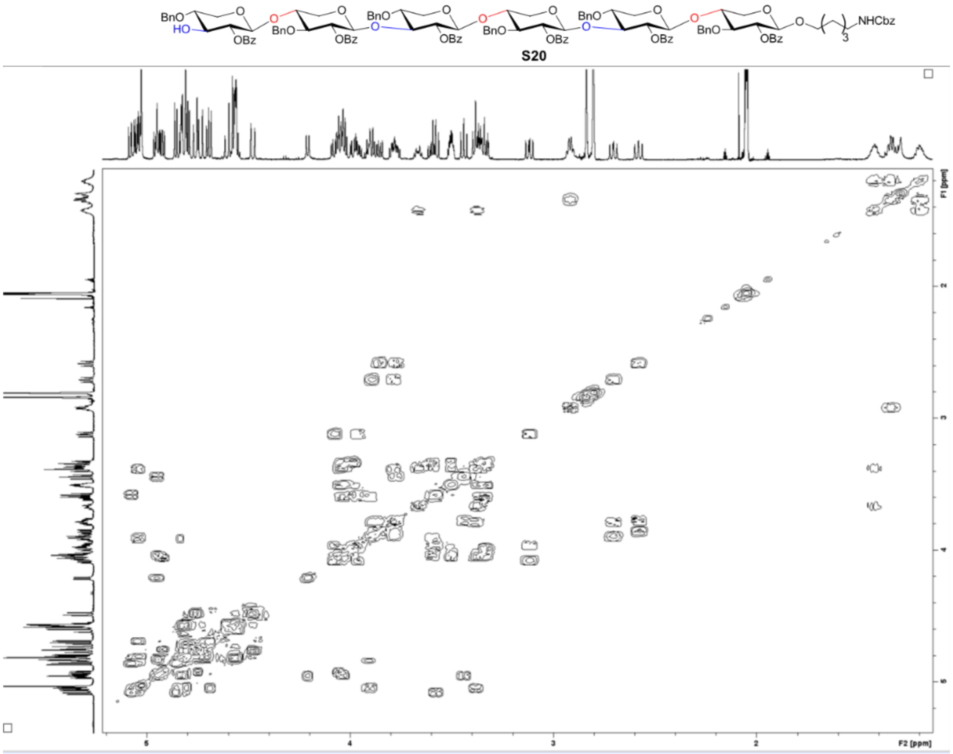

^{1}H-^{1}H COSY NMR spectrum of compound **S20** (600 MHz, $(CD_3)_2CO$)

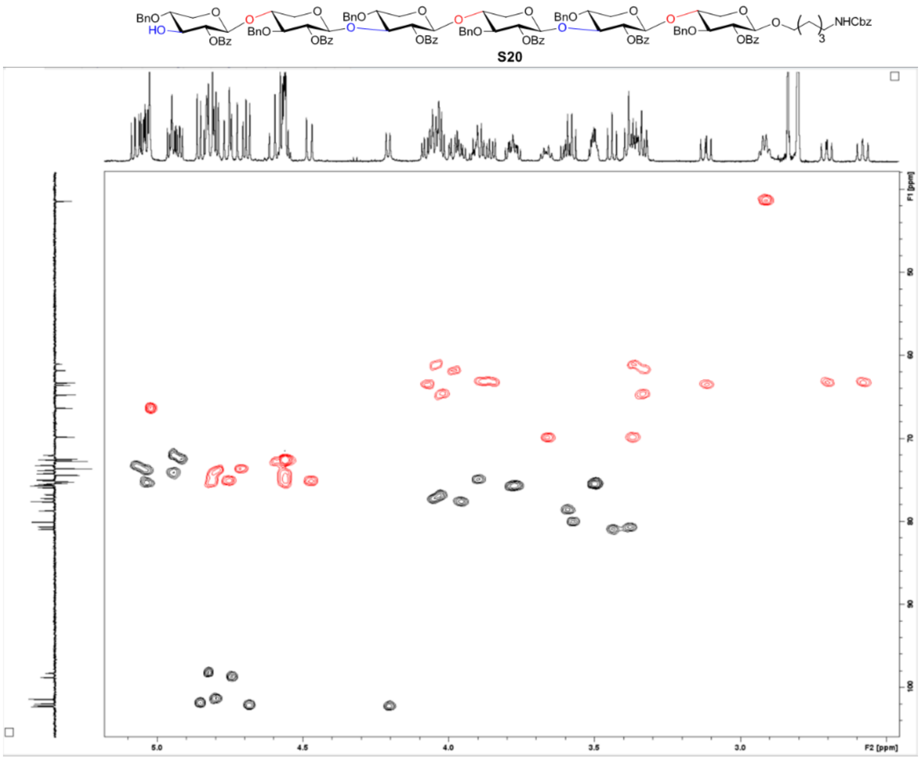

^{1}H-^{13}C HSQC NMR spectrum of compound **S20** (600/151 MHz, $(CD_3)_2CO$)

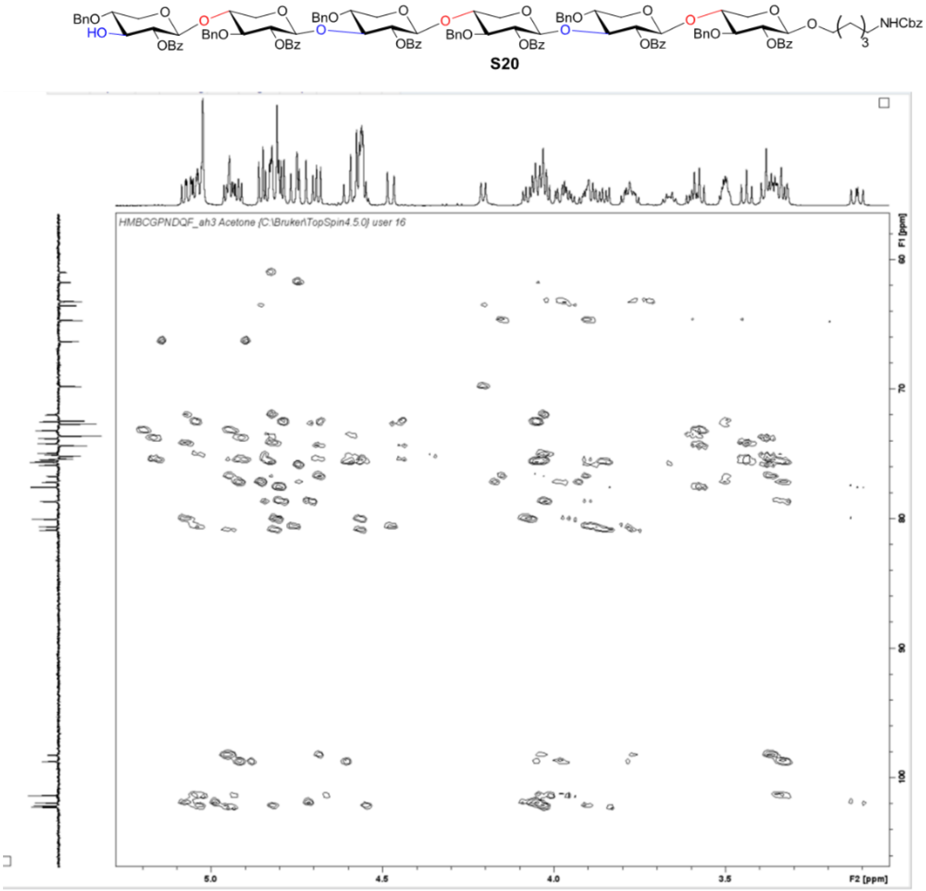

^{1}H-^{13}C HMBC NMR spectrum of compound **S20** (600/151 MHz, $(CD_3)_2CO$)

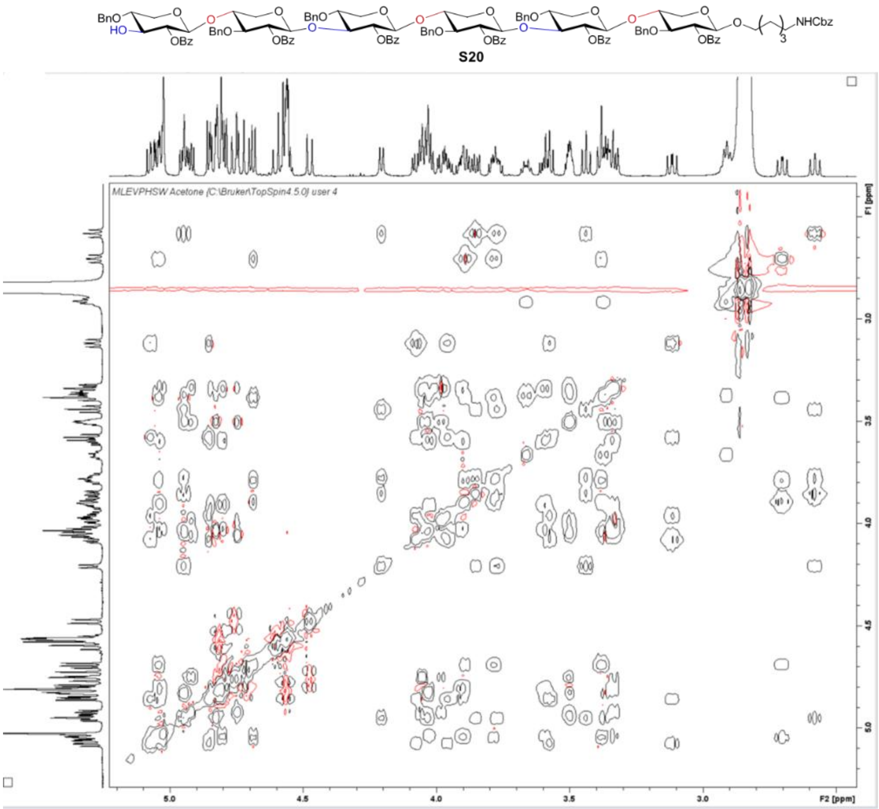

^{1}H-^{1}H TOCSY NMR spectrum of compound **S20** (600 MHz, $(CD_3)_2CO$)

Aminopentyl β-D-xylopyranosyl-(1→4)-β-D-xylopyranosyl-(1→3)-β-D-xylopyranosyl-(1→4)-β-D-xylopyranosyl-(1→3)-β-D-xylopyranosyl-(1→4)-β-D-xylopyranoside (19)

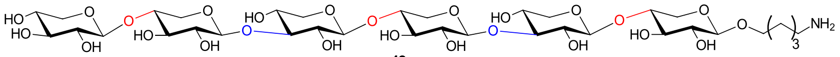

19

Experimental procedure: To a solution of protected MLX alternating hexasaccharide **S20** (3.8 mg, 1.73 µmol) in THF (100 µL, 17 mM), a solution of NaOMe in MeOH (0.5 M, 52 µL, 26 µmol) was added at rt, and the reaction mixture was allowed to stir overnight. After confirmation of reaction completion (26 h) by TLC (R_f: 0.28 (MeOH/$CHCl_3$ = 1/15, v/v)), the reaction mixture was neutralized by the addition of IR-120 H^+ resin while slowly stirring the mixture. Then, the reaction mixture was filtered, and the filtrate was concentrated under reduced pressure to yield a partially-deprotected glassy crude, which was kept under high vacuum until usage in the next step without any further purification. To a solution of partially deprotected crude (1.73 µmol) in *t*-BuOH (0.35 mL), H_2O (0.17 mL), and AcOH (60 µL), unreduced 10% Pd/C (3.5 mg) was added and the reaction mixture was stirred in the H_2 reactor under a pressure of 8 bar H_2. After 43 h, the reaction progress was checked by MALDI/TOF-MS analysis of the reaction mixture. Then, the reaction mixture was filtered using a PTFE syringe filter (0.45 µm) and concentrated under reduced pressure to yield a crude product, which was purified using pre-packed C18 (500 mg, 3 mL) column chromatography with H_2O/ACN (100% to 80%, v/v, + 0.1% AcOH). The purified product was lyophilized to give MLX alternating hexasaccharide **19** (1.76 mg, quantitative yield over 2 steps) as a white amorphous foam.

^{1}H NMR (600 MHz, D_2O): δ 4.69-4.65 (m, 2H, H-1C, H-1E), 4.49-4.43 (m, 3H, H-1B, H-1D, H-1F), 4.40 (d, J = 7.9 Hz, 1H, H-1A), 4.11-4.03 (m, 3H, H-5C, H-5E, H-5A), 4.01-3.93 (m, 3H, H-5B, H-5D, H-5F), 3.89-3.84 (m, 1H, OCH_2), 3.81-3.72 (m, 3H, H-4C, H-4E, H-4A), 3.71-3.65 (m, 3H, H-4B, H-4D, OCH_2), 3.65-3.59 (m, 3H, H-3B, H-3D, H-4F), 3.59-3.51 (m, 3H, H-3C, H-3E, H-3A), 3.46-3.39 (m, 3H, H-2B, H-2D, H-3F), 3.39-3.28 (m, 8H, H-5A, H-5C, H-5E, H-2C, H-2E, H-5B, H-5D, H-5F), 3.28-3.22 (m, 2H, H-2A, H-2F), 2.98 (t, J = 7.5 Hz, 2H, CH_2-NH_2), 1.71-1.61 (m, 4H, CH_2), 1.47-1.40 (m, 2H, CH_2) ppm.

^{13}C NMR (151 MHz, D_2O): δ 103.83, 103.38, 102.44, 102.27, 83.96, 83.95, 77.04, 77.02, 76.2, 74.46, 74.26, 73.86, 73.56, 73.36, 73.13, 73.11, 70.8, 69.8, 68.2, 65.81, 65.49, 63.5, 40.0, 28.8, 27.0, 22.7 ppm.

ESI-HRMS: m/z $[M + H]^+$ calcd. for $C_{35}H_{62}NO_{25}$: 896.3605; found 896.3610.

NMR chemical shifts of selected ^{1}H and ^{13}C atoms in compound **19**:

**xylose ring**	**proton**	***δ* (ppm)**	**multiplicity**	***J* (Hz)**	**carbon**	***δ* (ppm)**
A (reducing end)	H-1A	4.40	d	7.9	C-1A	103.38
	H-2A	3.26				
	H-3A	3.54				
	H-4A	3.75				
	H-5Aa	3.36				
	H-5Ab	4.06				
B	H-1B	4.49-4.43	m			102.27
	H-2B	3.46-3.39	m			
	H-3B	3.65-3.59	m			
	H-4B	3.71-3.65	m			
	H-5Ba	3.39-3.28	m			
	H-5Bb	4.01-3.93	m			
C	H-1C	4.69-4.65	m		C-1C	103.83
	H-2C	3.39-3.28	m			
	H-3C	3.59-3.31	m			
	H-4C	3.81-3.72	m			
	H-5Ca	3.39-3.28	m			
	H-5Cb	4.11-4.03	m			
D	H-1D	4.49-4.43	m		C-1D	102.27
	H-2D	3.46-3.39	m			
	H-3D	3.65-3.59	m			
	H-4D	3.71-3.65	m			
	H-5Da	3.39-3.28	m			
	H-5Db	4.01-3.93	m			
E	H-1E	4.69-4.65	m		C-1E	103.83
	H-2E	3.39-3.28	m			
	H-3E	3.59-3.31	m			
	H-4E	3.81-3.72	m			
	H-5Ea	3.39-3.28	m			
	H-5Eb	4.11-4.03	m			
F (non-reducing end)	H-1F	4.44			C-1F	102.44
	H-2F	3.24				
	H-3F	3.41				
	H-4F	3.61				
	H-5Fa	3.30				
	H-5Fb	3.96				

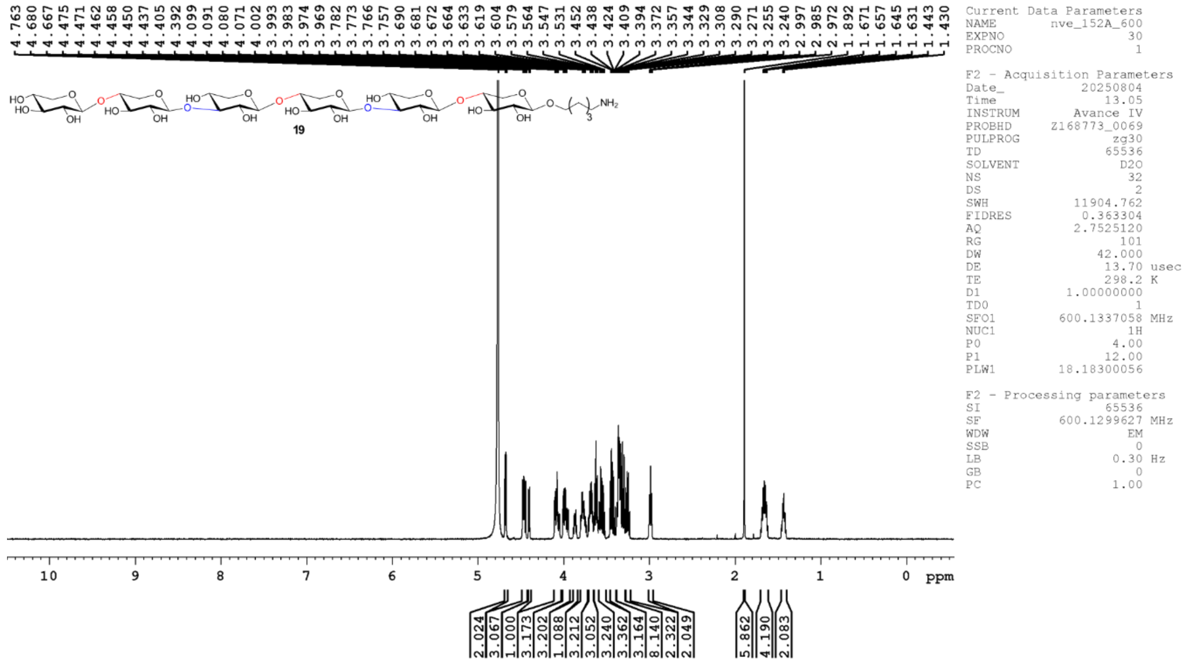

^{1}H NMR spectrum of compound **19** (600 MHz, D_2O)

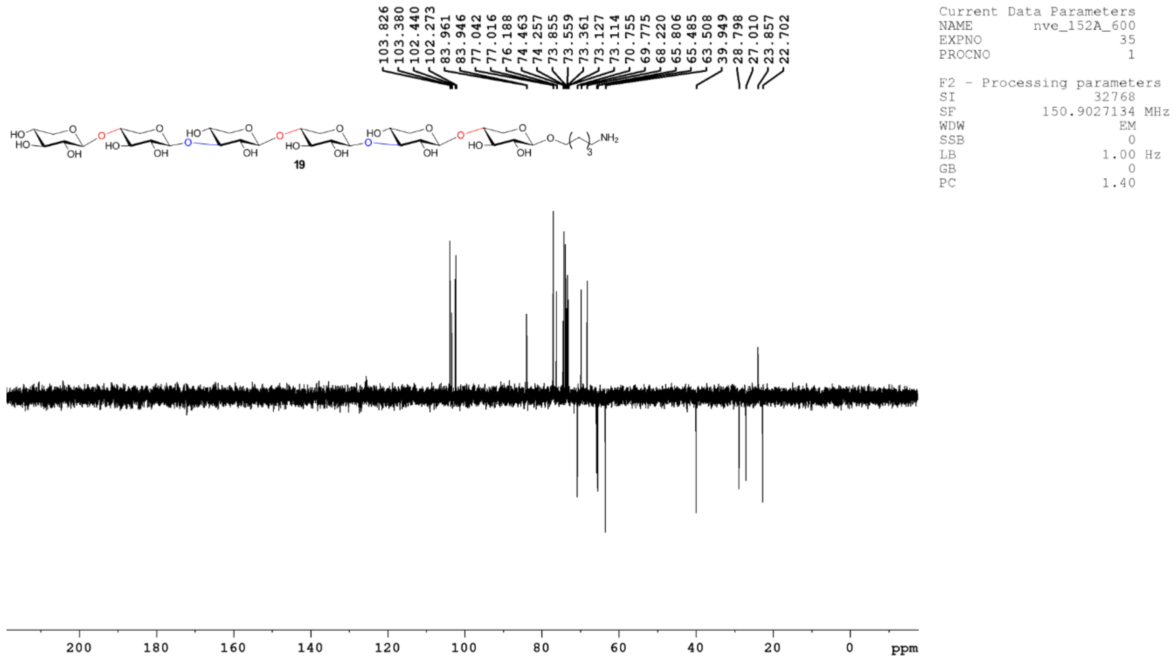

^{13}C APT NMR spectrum of compound **19** (151 MHz, D_2O)

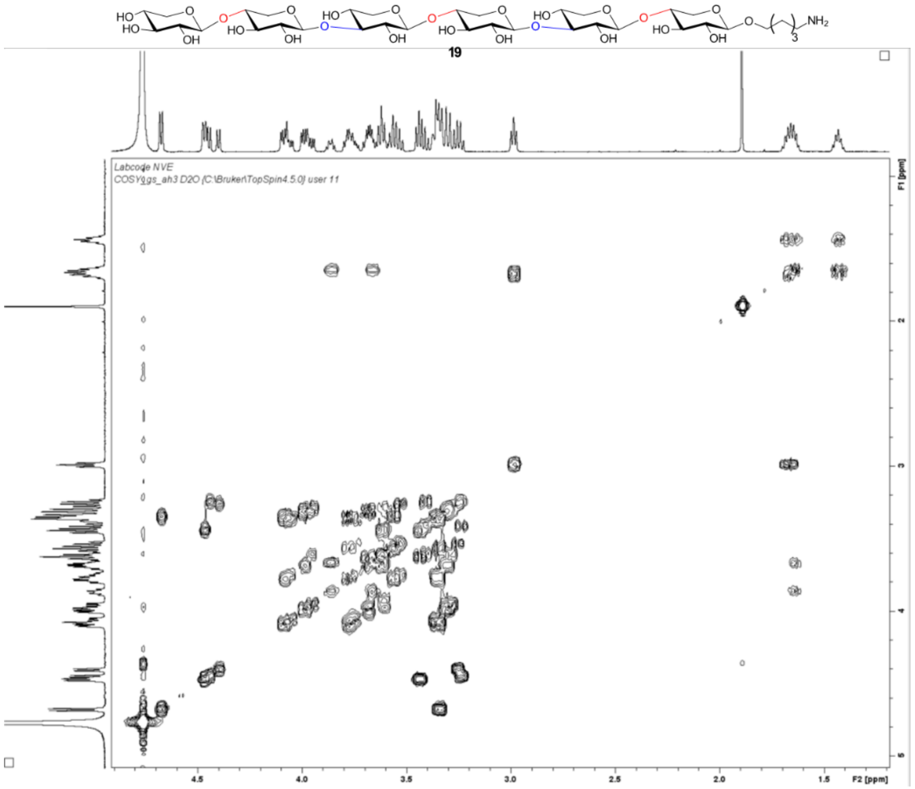

^{1}H-^{1}H COSY NMR spectrum of compound **19** (600 MHz, D_2O)

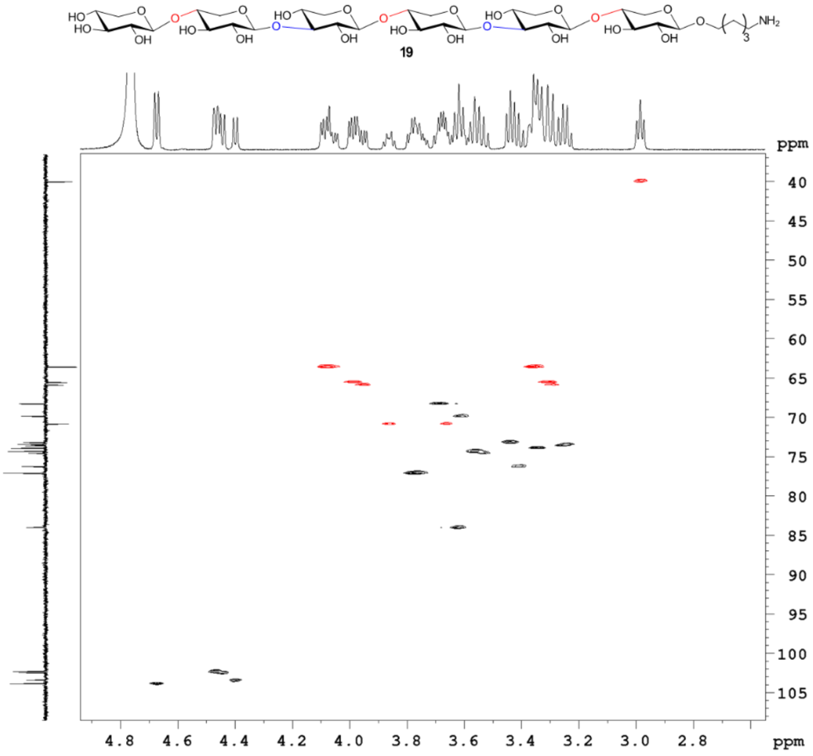

^{1}H-^{13}C HSQC NMR spectrum of compound **19** (600/151 MHz, D_2O)

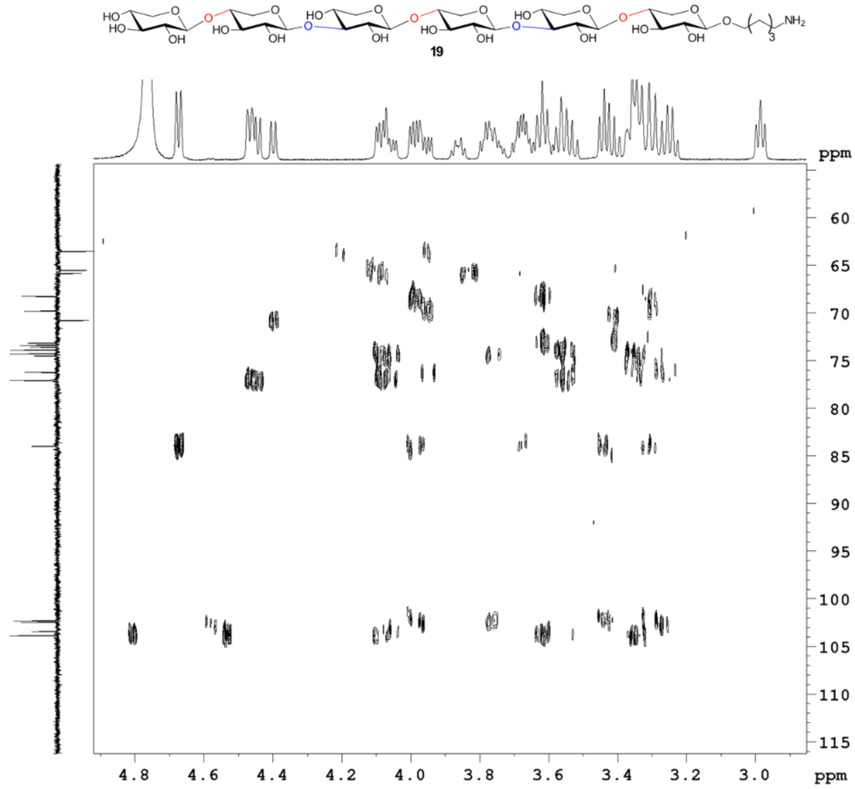

^{1}H-^{13}C HMBC NMR spectrum of compound **19** (600/151 MHz, D_2O)

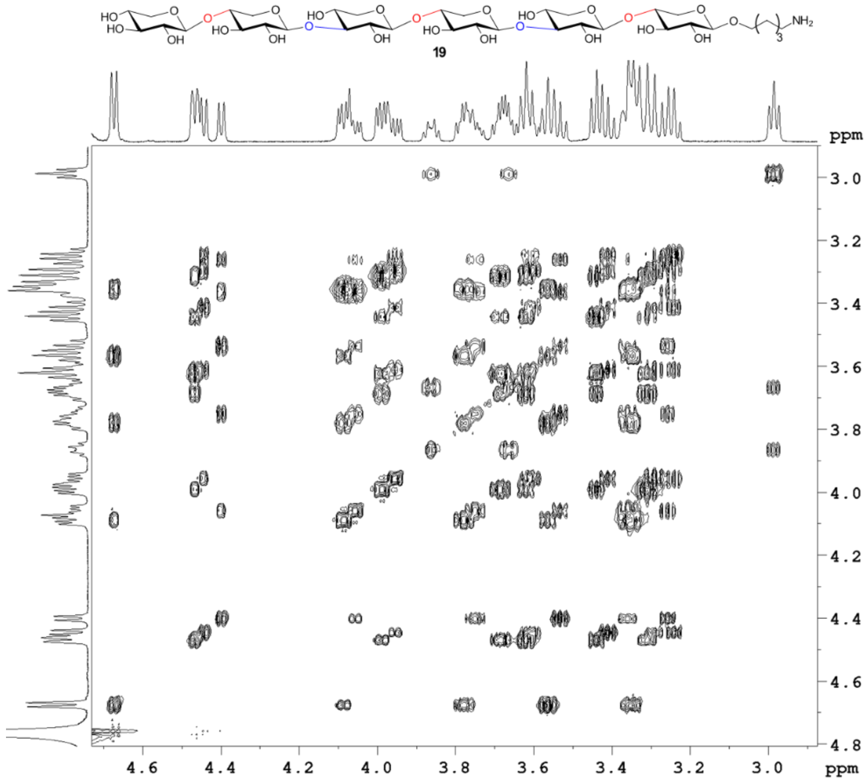

^{1}H-^{1}H TOCSY NMR spectrum of compound **19** (600 MHz, D_2O)

AGA of MLX hexasaccharide S21

Benzyloxycarbonylaminopentyl 2-*O*-benzoyl-3-*O*-benzyl-β-D-xylopyranosyl-(1→3)-2-*O*-benzoyl-4-*O*-benzyl-β-D-xylopyranosyl-(1→4)-2-*O*-benzoyl-3-*O*-benzyl-β-D-xylopyranosyl-(1→4)-2-*O*-benzoyl-3-*O*-benzyl-β-D-xylopyranosyl-(1→4)-2-*O*-benzoyl-3-*O*-benzyl-β-D-xylopyranosyl-(1→3)-2-*O*-benzoyl-4-*O*-benzyl-β-D-xylopyranoside (**S21**)

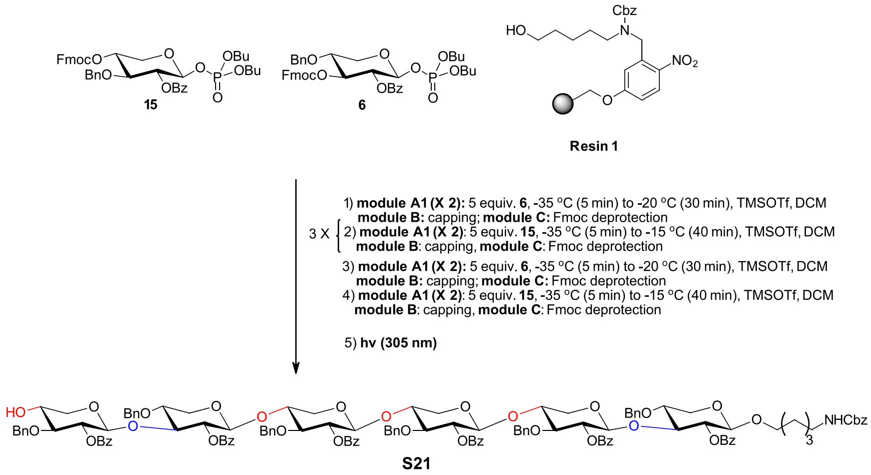

Experimental procedure: Linker-functionalized resin **1** (38 mg, 12.5 μmol) was placed in the synthesizer and synthesizer modules were applied as follows:

1) two cycles of module A1 (BB **6**, 47 mg, 0.0625 mmol, 5 equiv.) at –35 °C (5 min) to –20 °C (30 min), module B, and module C

3 × [

2) two cycles of module A1 (BB **15**, 47 mg, 0.0625 mmol, 5 equiv.) at –35 °C (5 min) to –15 °C (40 min), module B, and module C
   ]
3) two cycles of module A1 (BB **6**, 47 mg, 0.0625 mmol, 5 equiv.) at –35 °C (5 min) to –20 °C (30 min), module B, and module C
4) two cycles of module A1 (BB **15**, 47 mg, 0.0625 mmol, 5 equiv.) at –35 °C (5 min) to –15 °C (40 min), module B, and module C.

Cleavage from the resin using UV irradiation at 305 nm in a continuous flow photoreactor afforded the crude product. Purification of the crude by normal phase HPLC using a preparative YMC-Diol column (EtOAc/hexanes = 1/9 to 1/1.5, v/v) gave protected MLX hexasaccharide **S21** (8.8 mg, 32% yield over 13 steps) as a glassy solid.

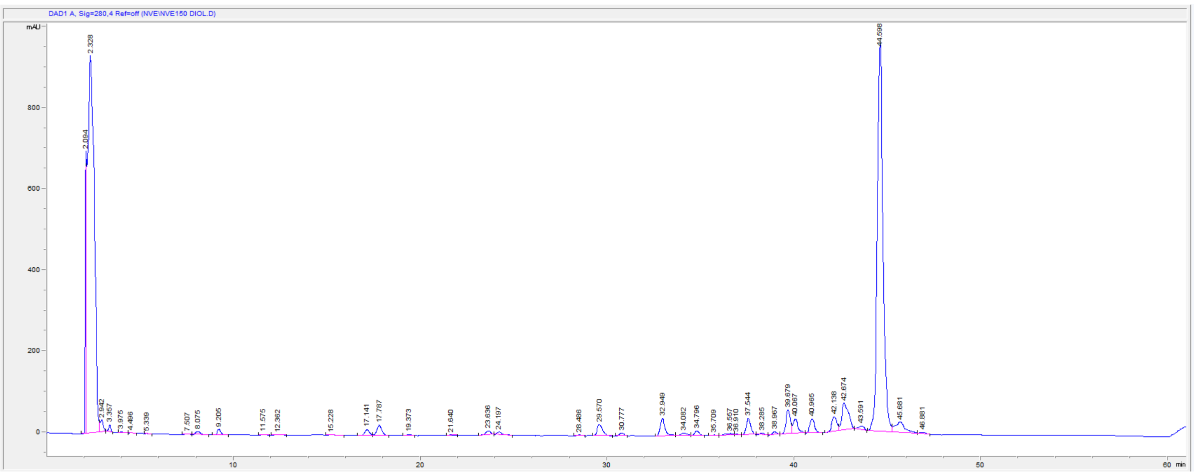

Analytical HPLC of the crude reaction mixture was performed on a YMC-Diol column using a gradient of EtOAc in hexanes (60 min, flow rate 1 mL/min).

^{1}H NMR (600 MHz, $(CD_3)_2CO$): δ 8.12-8.08 (m, 2H, Ar-H), 8.07-8.03 (m, 2H, Ar-H), 8.03-7.99 (m, 4H, Ar-H), 7.86-7.81 (m, 2H, Ar-H), 7.74-7.71 (m, 2H, Ar-H), 7.69-7.61 (m, 4H, Ar-H), 7.58-7.50 (m, 8H, Ar-H), 7.49-7.44 (m, 2H, Ar-H), 7.39-7.36 (m, 4H, Ar-H), 7.35-7.23 (m, 14H, Ar-H), 7.23-7.16 (m, 3H, Ar-H), 7.15-7.024 (m, 18H, Ar-H), 6.07 (br, 1H, N-H), 5.15 (dd, J = 7.6, 9.1 Hz, 1H, H-2F), 5.07-4.98 (m, 7H, H-2D, H-2C, H-2E, H-2B, H-1F, CH_2-Cbz), 4.92 (dd, J = 7.0, 8.5 Hz, 1H, H-2A), 4.87-4.82 (m, 2H, H-1E, CH_2-Ph), 4.81-4.76 (m, 3H, H-1B, CH_2-Ph), 4.74-4.57 (m, 10H, H-1C, O-H, CH_2-Ph), 4.49-4.43 (m, 3H, H-1A, H-1D, CH_2-Ph), 4.21 (t, J = 6.0 Hz, 1H, H-3E), 4.09 (dd, J = 3.9, 12.3 Hz, 1H, H-5E), 4.04-3.91 (m, 6H, H-3A, H-5B, H-5F, H-5C, H-5D, H-5A), 3.87-3.79 (m, 4H, H-4D, H-4B, H-4C, H-4F), 3.64-3.52 (m, 5H, H-3C, H-4E, OCH_2, H-3F, H-4A), 3.50-3.40 (m, 3H, H-3B, H-3D, H-5E), 3.36-3.26 (m, 3H, H-5F, H-5A, OCH_2), 3.10-3.0 (m, 2H, H-5C, H-5B), 2.9-2.83 (m, 2H, CH_2-NHCbz), 2.72 (dd, J = 10.0, 11.6 Hz, 1H, H-5D), 1.37-1.28 (m, 4H, CH_2), 1.15-1.05 (m, 2H, CH_2) ppm.

^{13}C NMR (151 MHz, $(CD_3)_2CO$): δ 165.76, 165.63, 165.58, 165.51, 165.25, 157.0, 139.80, 139.73, 139.61, 139.47, 139.40, 138.6, 134.15, 134.08, 133.97, 133.85, 133.57, 131.42, 131.13, 131.06, 131.02, 130.90, 130.85, 130.77, 130.56, 130.52, 130.49, 130.43, 130.36, 129.58, 129.48, 129.46, 129.44, 129.25, 129.18, 129.04, 128.99, 128.89, 128.77, 128.75, 128.71, 128.65, 128.63, 128.54, 128.40, 128.23, 128.22, 128.07, 128.03, 128.01, 102.3, 101.61, 101.34, 101.06, 100.9, 98.7, 83.34, 83.30, 80.5, 79.77, 79.54, 78.8, 77.41, 77.24, 77.15, 76.7, 75.74, 75.63, 75.22, 74.72, 74.32, 74.14, 73.98, 73.93, 73.39, 73.32, 73.01, 72.66, 72.48, 71.21, 71.11, 69.4, 66.79, 66.77, 66.24, 63.71, 63.46, 63.36, 63.05, 61.7, 41.3, 30.2, 23.7 ppm.

ESI-HRMS: m/z $[M + Na]^+$ calcd. for $C_{127}H_{127}NO_{33}Na$: 2216.8183; found 2216.8058.

NMR chemical shifts of selected ^{1}H and ^{13}C atoms in compound **S21**:

**xylose ring**	**proton**	***δ* (ppm)**	**multiplicity**	***J* (Hz)**	**carbon**	***δ* (ppm)**
A (reducing end)	H-1A	4.47			C-1A	101.61
	H-2A	4.92	dd	7.0, 8.5		
	H-3A	4.02			C-3A	78.8
	H-4A	3.55			C-4A	76.7
	H-5Aa	3.32			C-5A	63.71
	H-5Ab	3.91				
B	H-1B	4.79			C-1B	101.34
	H-2B	5.00				
	H-3B	3.48			C-3B	79.54
	H-4B	3.83				
	H-5Ba	3.03			C-5B	63.05
	H-5Bb	4.01				
C	H-1C	4.64			C-1C	100.9
	H-2C	5.02				
	H-3C	3.62			C-3C	79.77
	H-4C	3.83				
	H-5Ca	3.08			C-5C	63.46
	H-5Cb	3.96				
D	H-1D	4.45			C-1D	101.06
	H-2D	5.04				
	H-3D	3.47				
	H-4D	3.85				
	H-5Da	2.72	dd	10.0, 11.6	C-5D	63.36
	H-5Db	3.93				
E	H-1E	4.85			C-1E	98.7
	H-2E	5.02				
	H-3E	4.21	t	6.0	C-3E	77.15
	H-4E	3.61			C-4E	79.77
	H-5Ea	3.44			C-5E	61.7
	H-5Eb	4.09	dd	3.9, 12.3		
F (non-reducing end)	H-1F	4.99			C-1F	102.3
	H-2F	5.15	dd	7.6, 9.1	C-2F	73.93
	H-3F	3.58			C-3F	83.34
	H-4F	3.83				
	H-5Fa	3.33			C-5F	66.77
	H-5Fb	3.98				

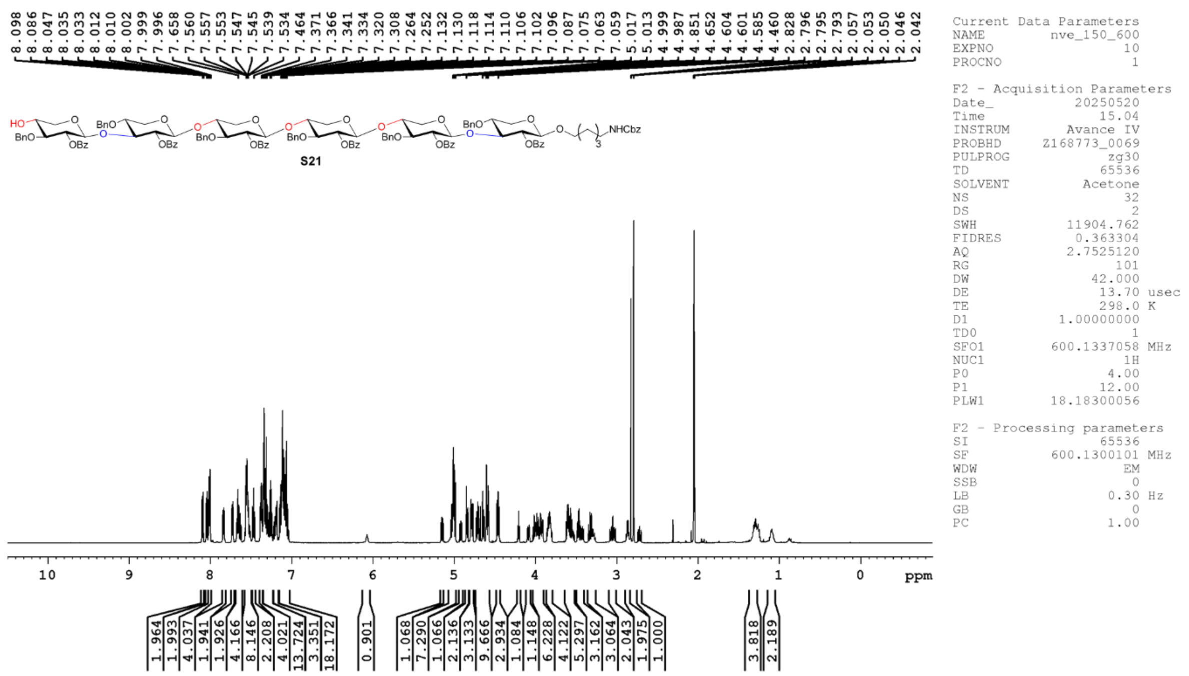

^{1}H NMR spectrum of compound **S21** (600 MHz, $(CD_3)_2CO$)

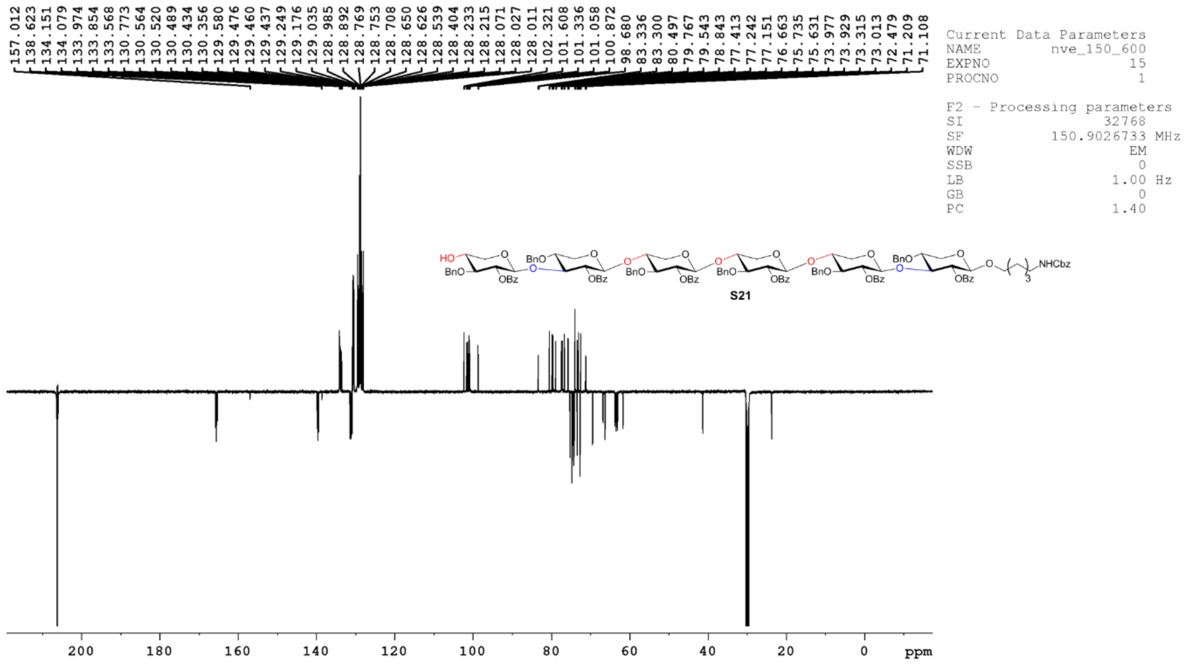

^{13}C APT NMR spectrum of compound **S21** (151 MHz, $(CD_3)_2CO$)

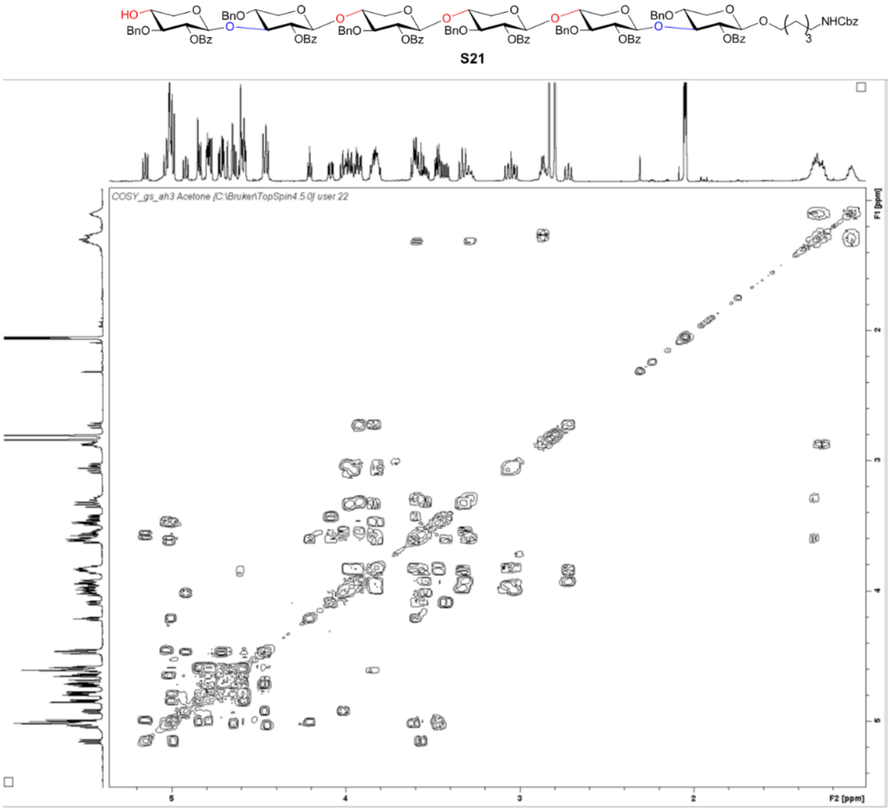

^{1}H-^{1}H COSY NMR spectrum of compound **S21** (600 MHz, $(CD_3)_2CO$)

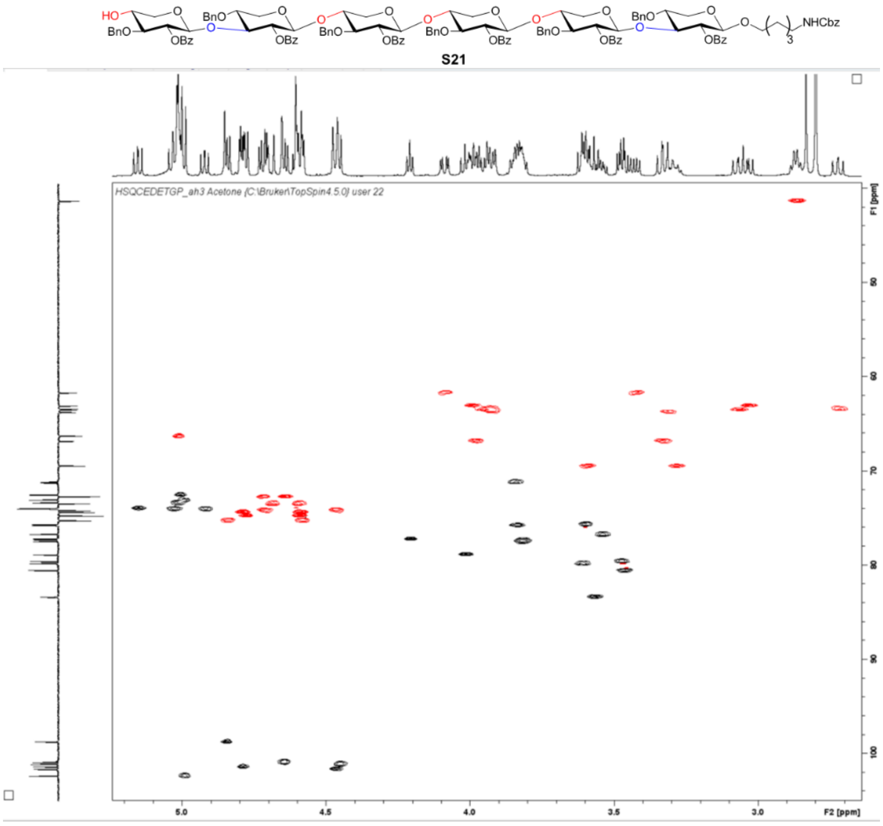

^{1}H-^{13}C HSQC NMR spectrum of compound **S21** (600/151 MHz, $(CD_3)_2CO$)

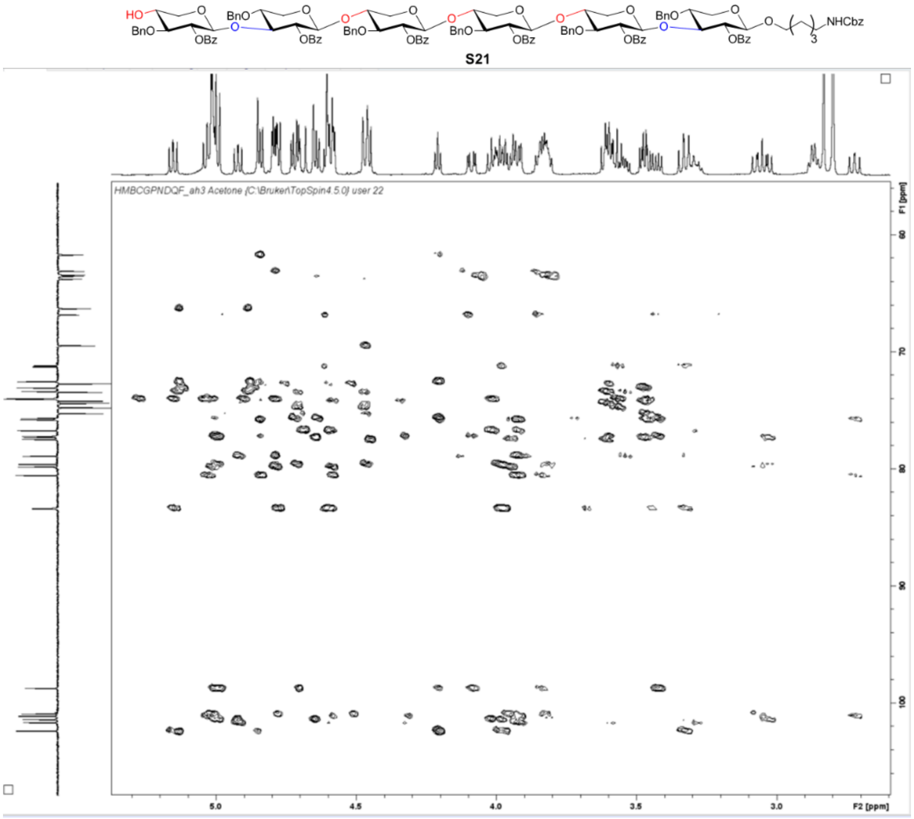

^{1}H-^{13}C HMBC NMR spectrum of compound **S21** (600/151 MHz, $(CD_3)_2CO$)

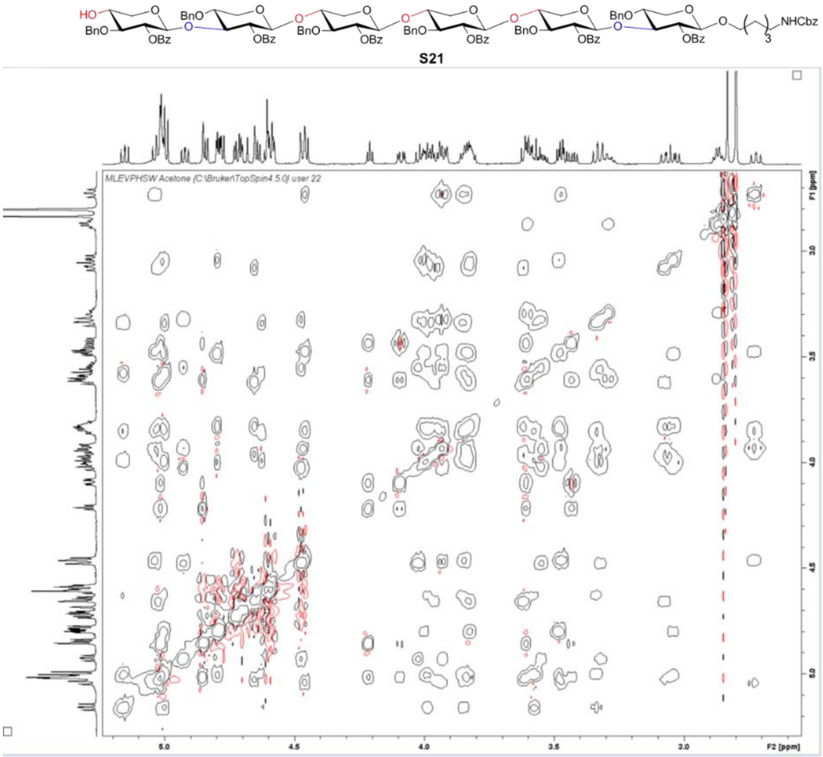

^{1}H-^{1}H TOCSY NMR spectrum of compound **S21** (600 MHz, $(CD_3)_2CO$)

Aminopentyl β-D-xylopyranosyl-(1→3)-β-D-xylopyranosyl-(1→4)-β-D-xylopyranosyl-(1→4)-β-D-xylopyranosyl-(1→4)-β-D-xylopyranosyl-(1→3)-β-D-xylopyranoside (20)

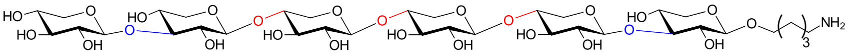

20

Experimental procedure: To a solution of protected MLX hexasaccharide **S21** (7.9 mg, 3.6 µmol) in THF (216 µL, 17 mM), a solution of NaOMe in MeOH (0.5 M, 108 µL, 54 µmol) was added at rt, and the reaction mixture was allowed to stir overnight. After confirmation of reaction completion (24 h) by TLC (R_f: 0.33 (MeOH/$CHCl_3$ = 1/15, v/v)), the reaction mixture was neutralized by the addition of IR-120 H^+ resin while slowly stirring the mixture. Then, the reaction mixture was filtered, and the filtrate was concentrated under reduced pressure to yield a partially-deprotected glassy crude, which was kept under high vacuum until usage in the next step without any further purification. To a solution of partially deprotected crude (3.6 µmol) in *t*-BuOH (0.72 mL), H_2O (0.36 mL), and AcOH (0.18 mL), unreduced 10% Pd/C (7.9 mg) was added and the reaction mixture was stirred in the H_2 reactor under a pressure of 8 bar H_2. After 47 h, the reaction progress was checked by MALDI/TOF-MS analysis of the reaction mixture. Then, the reaction mixture was filtered using a PTFE syringe filter (0.45 µm) and concentrated under reduced pressure to yield a crude product, which was purified using pre-packed C18 (500 mg, 3 mL) column chromatography with H_2O/ACN (100% to 80%, v/v, + 0.1% AcOH). The purified product was lyophilized to give MLX hexasaccharide **20** (3.3 mg, 95% yield over 2 steps) as a white amorphous foam.

^{1}H NMR (600 MHz, D_2O): δ 4.70-4.63 (m, 2H, H-1C, H-1B), 4.50-4.44 (m, 3H, H-1D, H-1E, H-1F), 4.41 (d, J = 7.9 Hz, 1H, H-1A), 4.13-4.05 (m, 3H, H-5C), 4.02-3.92 (m, 3H, H-5A, H-5B), 3.89-3.83 (m, 1H, OCH_2), 3.82-3.74 (m, 3H, H-4C), 3.71-3.64 (m, 3H, H-4A, OCH_2), 3.64-3.59 (m, 3H, H-3A, H-4B), 3.59-3.51 (m, 3H, H-3C), 3.46-3.40 (m, 3H, H-2A, H-3B), 3.39-3.25 (m, 10H, H-2C, H-5C, H-2B, H-5A, H-5B), 2.98 (t, J = 7.5 Hz, 2H, CH_2-NH_2), 1.70-1.61 (m, 4H, CH_2), 1.47-1.40 (m, 2H, CH_2) ppm.

^{13}C NMR (151 MHz, D_2O): δ 104.0, 103.90, 103.28, 102.3, 84.39, 84.04, 76.97, 76.96, 76.92, 76.21, 74.25, 73.95, 73.87, 73.28, 73.08, 70.8, 69.8, 68.26, 68.24, 65.76, 65.47, 65.37, 63.56, 63.51, 40.0, 28.8, 27.0, 22.7 ppm.

ESI-HRMS: m/z [M + Na]$^+$ calcd. for $C_{35}H_{61}NO_{25}Na$: 918.3425; found 918.3439.

NMR chemical shifts of selected ^{1}H and ^{13}C atoms in compound **20**:

**xylose ring**	**proton**	***δ* (ppm)**	**multiplicity**	***J* (Hz)**	**carbon**	***δ* (ppm)**
A (reducing end)	H-1A	4.41	d	7.9	C-1A	103.28
	H-2A	3.43				
	H-3A	3.62			C-3A	84.39
	H-4A	3.66			C-4A	68.26
	H-5Aa	3.31			C-5A	65.47
	H-5Ab	3.96				
B	H-1B	4.70-4.63	m			
	H-2B	3.39-3.25	m			
	H-3B	3.46-3.40	m			
	H-4B	3.64-3.59	m			
	H-5Ba	3.39-3.25	m			
	H-5Bb	4.02-3.92	m			
C	H-1C	4.70-4.63	m			
	H-2C	3.39-3.25	m			
	H-3C	3.59-3.51	m			
	H-4C	3.82-3.74	m			
	H-5Ca	4.13-4.05	m			
	H-5Cb	3.39-3.25	m			

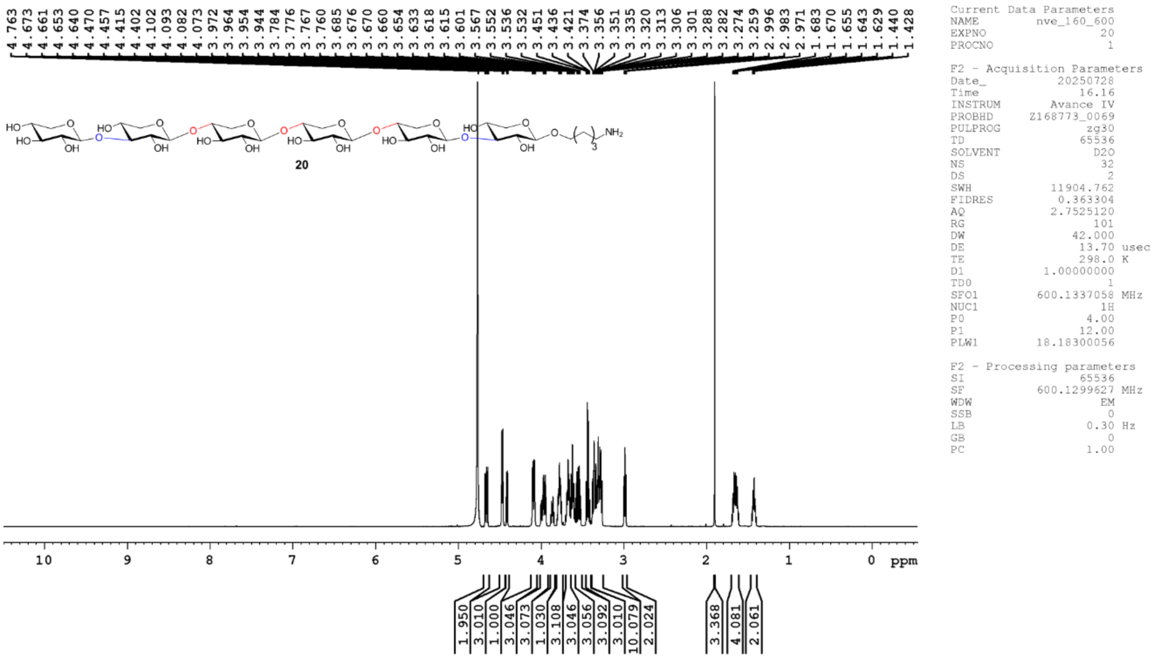

^{1}H NMR spectrum of compound **20** (600 MHz, D_2O)

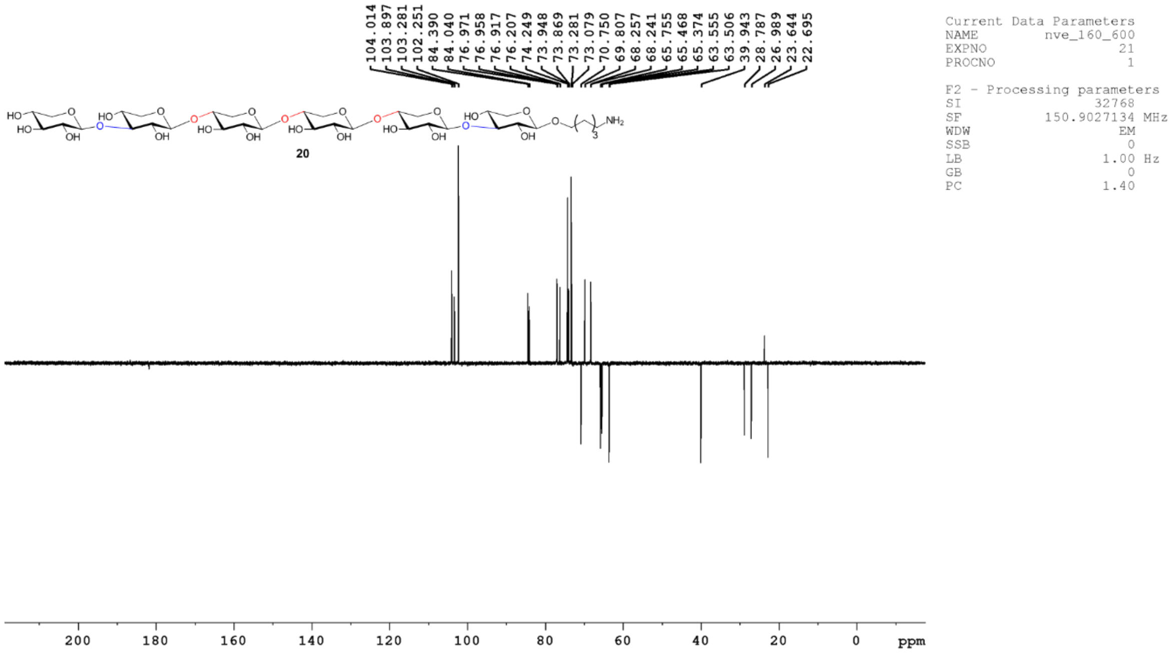

^{13}C DEPTQ135 NMR spectrum of compound **20** (151 MHz, D_2O)

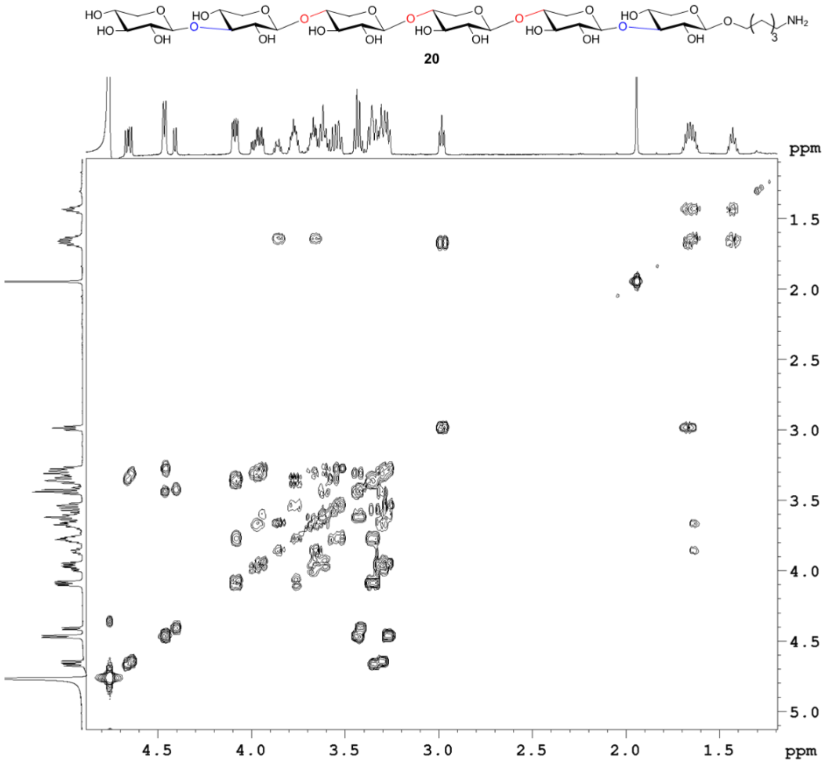

^1H-^1H COSY NMR spectrum of compound **20** (600 MHz, D_2O)

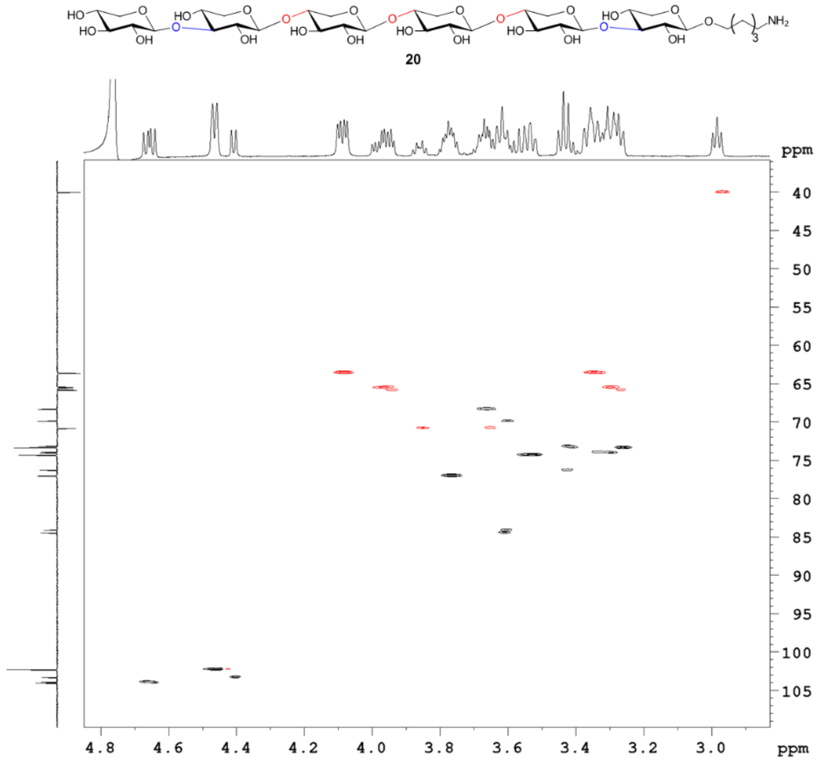

^1H-^{13}C HSQC NMR spectrum of compound **20** (600/151 MHz, D_2O)

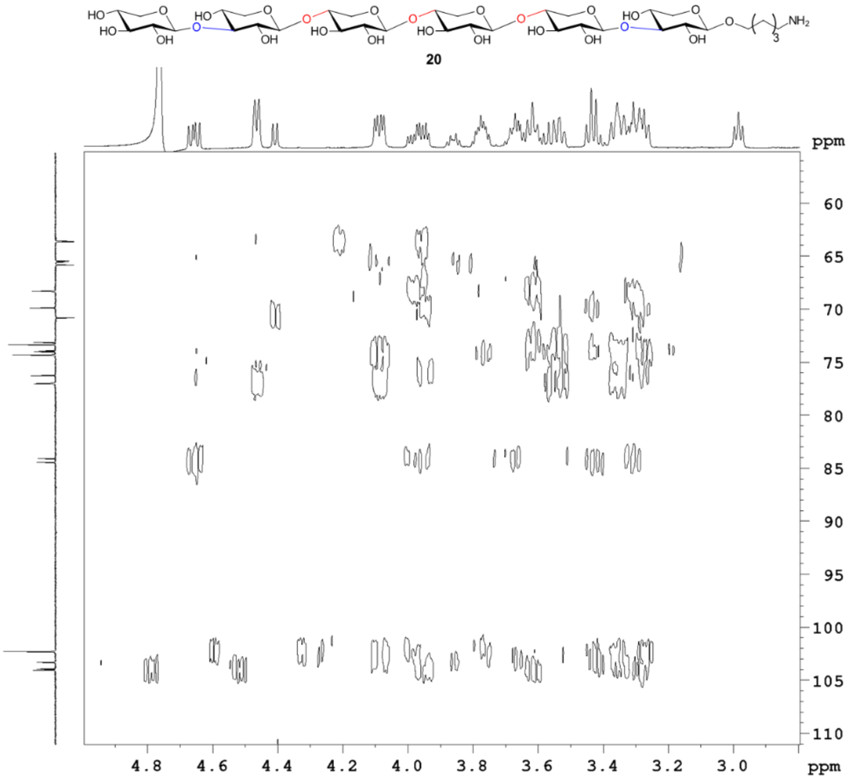

^{1}H-^{13}C HMBC NMR spectrum of compound **20** (600/151 MHz, D_2O)

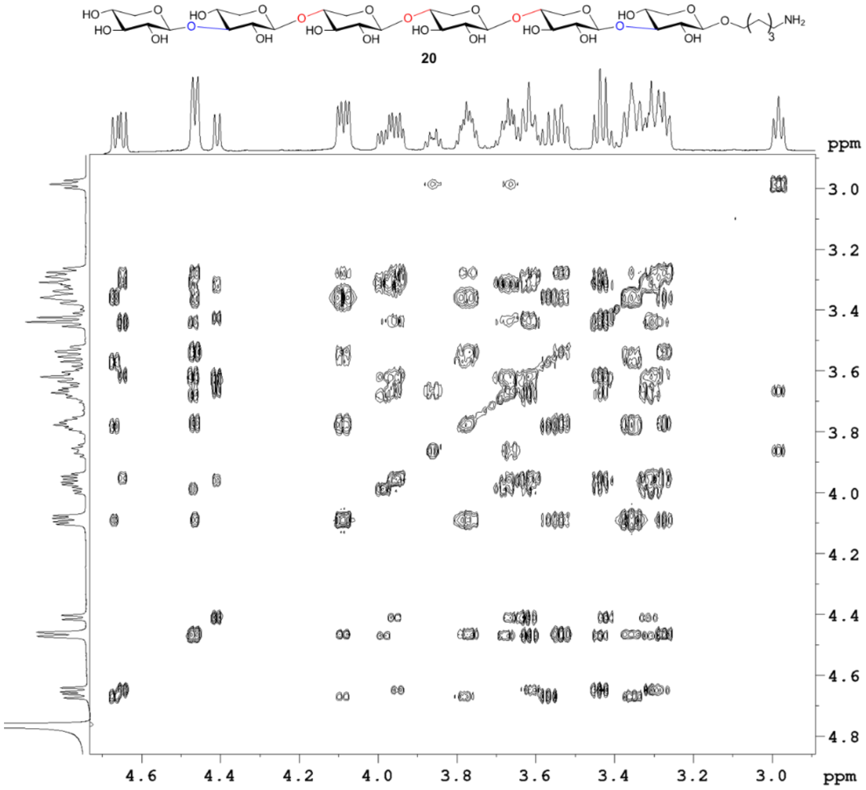

^{1}H-^{1}H TOCSY NMR spectrum of compound **20** (600 MHz, D_2O)

AGA of MLX heptasaccharide S22

Benzyloxycarbonylaminopentyl 2-*O*-benzoyl-4-*O*-benzyl-β-D-xylopyranosyl-(1→4)-2-*O*-benzoyl-3-*O*-benzyl-β-D-xylopyranosyl-(1→4)-2-*O*-benzoyl-3-*O*-benzyl-β-D-xylopyranosyl-(1→4)-2-*O*-benzoyl-3-*O*-benzyl-β-D-xylopyranosyl-(1→4)-2-*O*-benzoyl-3-*O*-benzyl-β-D-xylopyranosyl-(1→3)-2-*O*-benzoyl-4-*O*-benzyl-β-D-xylopyranosyl-(1→4)-2-*O*-benzoyl-3-*O*-benzyl-β-D-xylopyranoside (**S22**)

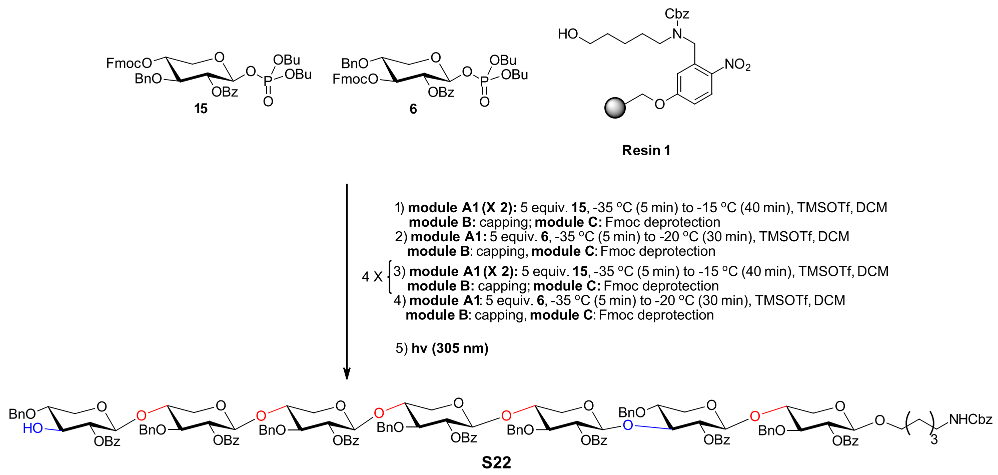

Experimental procedure: Linker-functionalized resin **1** (38 mg, 12.5 µmol) was placed in the synthesizer and synthesizer modules were applied as follows:

1) two cycles of module A1 (BB **15**, 47 mg, 0.0625 mmol, 5 equiv.) at –35 °C (5 min) to –15 °C (40 min), module B, and module C
2) module A1 (BB **6**, 47 mg, 0.0625 mmol, 5 equiv.) at –35 °C (5 min) to –20 °C (30 min), module B, and module C

4 × [

3) two cycles of module A1 (BB **15**, 47 mg, 0.0625 mmol, 5 equiv.) at –35 °C (5 min) to –15 °C (40 min), module B, and module C
]
4) module A1 (BB **6**, 47 mg, 0.0625 mmol, 5 equiv.) at –35 °C (5 min) to –20 °C (30 min), module B, and module C

Cleavage from the resin using UV irradiation at 305 nm in a continuous flow photoreactor afforded the crude product. Purification of the crude by normal phase HPLC using a preparative YMC-Small column (EtOAc/hexanes = 1/9 to 1/1.5, v/v) gave protected MLX heptasaccharide **S22** (3.3 mg, 11% yield over 15 steps) as a glassy solid.

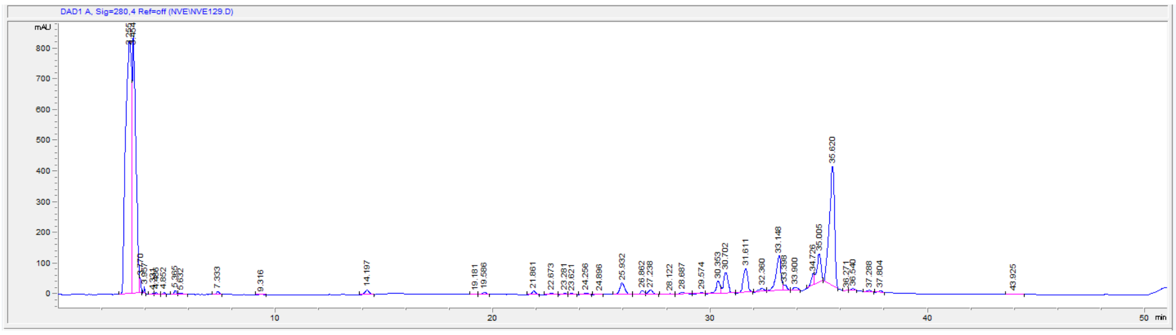

Analytical HPLC of the crude reaction mixture was performed on a YMC-Small NP column using a gradient of EtOAc in hexanes (50 min, flow rate 1 mL/min). MLX heptasaccharide **S22** was eluted at 35.6 min.

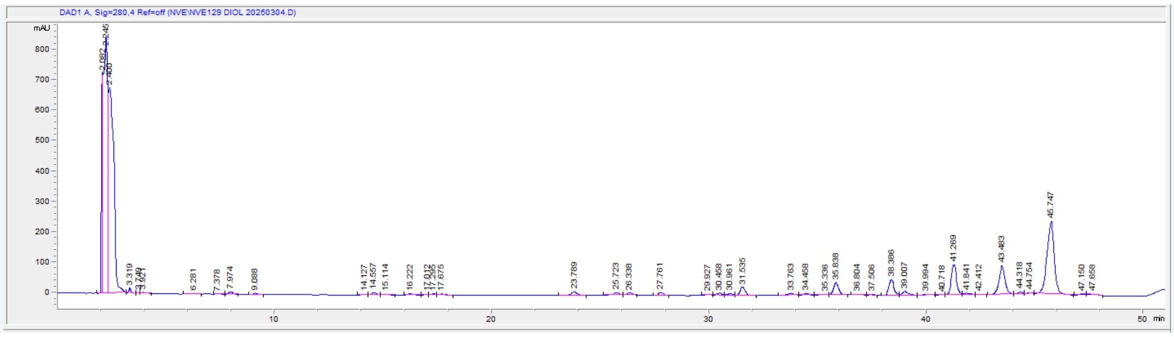

Analytical HPLC of the crude reaction mixture (diluted in 4 mL toluene, injection volume = 70 µL) was performed on a YMC-Diol column using a gradient of EtOAc in hexanes (50 min, flow rate 1 mL/min). MLX heptasaccharide **S22** was eluted at 45.75 min.

^{1}H NMR (600 MHz, $(CD_3)_2CO$): δ 8.12-8.09 (m, 2H, Ar-H), 8.06-8.01 (m, 8H, Ar-H), 7.95-7.92 (m, 2H, Ar-H), 7.90-7.87 (m, 2H, Ar-H), 7.68-7.60 (m, 6H, Ar-H), 7.58-7.49 (m, 13H, Ar-H), 7.41-7.31 (m, 10H, Ar-H), 7.30-7.25 (m, 4H, Ar-H), 7.24-7.18 (m, 5H, Ar-H), 7.17-7.03 (m, 21H, Ar-H), 7.03-6.99 (m, 2H, Ar-H), 6.1 (br, 1H, N-H), 5.11-4.98 (m, 7H, H-2F, H-2G, H-2C, H-2D, H-2E, CH_2-Cbz), 4.95-4.90 (m, 2H, H-2A, H-2B), 4.90-4.80 (m, 6H, CH_2-Ph, O-H, H-1G, H-1C), 4.80-4.65 (m, 9H, H-1B, H-1F, H-1D, H-1E, CH_2-Ph), 4.62 (d, J = 11.6 Hz, 1H, CH_2-Ph), 4.60-4.53 (m, 4H, CH_2-Ph), 4.48 (d, J = 11.5 Hz, 1H, CH_2-Ph), 4.22 (d, J = 7.7 Hz, 1H, H-1A), 4.11-4.05 (m, 2H, H-5F, H-5G), 4.05-3.88 (m, 8H, H-3B, H-5B, H-4F, H-5C, H-5D, H-5E, H-3G), 3.87-3.81 (m, 3H, H-4C, H-5A), 3.79-3.74 (m, 1H, H-4A), 3.69 (t, J = 8.6 Hz, 1H, H-3F), 3.66-3.59 (m, 4H, OCH_2, H-4G, H-3D, H-3E), 3.54-3.48 (m, 2H, H-3C, H-4B), 3.44 (t, J = 9.0 Hz, 1H, H-3A), 3.41-3.32 (m, 3H, H-5G, OCH_2, H-5B), 3.15 (dd, J = 9.5, 11.7 Hz, 1H, H-5F), 3.11-3.02 (m, 3H, H-5D, H-5E, H-5C), 2.94-2.87 (m, 2H, CH_2-NHCbz), 2.61 (dd, J = 10.2, 11.3 Hz, 1H, H-5A), 1.45-1.37 (m, 2H, CH_2), 1.35-1.31 (m, 2H, CH_2, merged with EtOAc peak), 1.22-1.15 (m, 2H, CH_2) ppm.

^{13}C NMR (151 MHz, $(CD_3)_2CO$): δ 165.88, 165.61, 165.55, 165.48, 139.97, 139.68, 139.61, 139.56, 139.52, 139.46, 139.38, 134.08, 134.05, 133.99, 133.96, 133.91, 131.97, 131.37, 131.29, 131.23, 131.1, 131.08, 130.76, 130.73, 130.47, 130.45, 130.43, 130.35, 129.47, 129.46, 129.38, 129.32, 129.17, 129.05, 128.96, 128.92, 128.86, 128.83, 128.79, 128.78, 128.70, 128.68, 128.62, 128.60, 128.53, 128.29, 128.21, 128.09, 128.07, 127.99, 127.95, 102.16, 101.82, 101.25, 100.74, 100.7, 98.6, 80.7, 79.93, 79.89, 79.86, 79.68, 78.70, 78.66, 77.44, 77.24, 77.13, 76.97, 75.91, 75.54, 75.43, 75.40, 75.19, 75.08, 74.97, 74.39, 74.29, 74.00, 73.58, 73.47, 73.35, 73.20, 72.56, 72.38, 69.7, 66.3, 64.7, 63.61, 63.49, 63.38, 63.19, 61.6, 41.3, 23.7 ppm.

ESI-HRMS: m/z $[M + HCOO]^-$ calcd. for $C_{147}H_{146}NO_{40}$: 2564.9427; found 2564.9397.

NMR chemical shifts of selected ^{1}H and ^{13}C atoms in compound **S22**:

**xylose ring**	**proton**	***δ*** **(ppm)**	**multiplicity**	***J*** **(Hz)**	**carbon**	***δ*** **(ppm)**
A (reducing end)	H-1A	4.22	d	7.7	C-1A	102.16
	H-2A	4.93			C-2A	74.00
	H-3A	3.44	t	9.0	C-3A	80.70
	H-4A	3.79-3.74	m		C-4A	75.91
	H-5Aa	2.61	dd	10.2, 11.3	C-5A	63.19
	H-5Ab	3.82				
B	H-1B	4.77			C-1B	98.63
	H-2B	4.92			C-2B	72.38
	H-3B	4.03			C-3B	76.97
	H-4B	3.51			C-4B	75.54
	H-5Ba	3.34			C-5B	61.60
	H-5Bb	4.02				
C	H-1C	4.83			C-1C	101.82
	H-2C	5.03				
	H-3C	3.52			C-3C	79.86
	H-4C	3.83				
	H-5Ca	3.05				
	H-5Cb	3.98				
D	H-1D	4.80-4.65	m			
	H-2D	5.11-4.98	m			
	H-3D	3.66-3.59	m			
	H-5Da	3.11-3.02	m			
	H-5Db	4.05-3.88				
E	H-1E	4.80-4.65	m			
	H-2E	5.11-4.98	m			
	H-3E	3.66-3.59	m			
	H-5Ea	3.11-3.02	m			
	H-5Eb	4.05-3.88	m			
F	H-1F	4.76			C-1F	100.74
	H-2F	5.09			C-2F	73.47
	H-3F	3.69	t	8.6	C-3F	79.86
	H-4F	4.01			C-4F	77.44
	H-5Fa	3.15	dd	9.5, 11.7	C-5F	63.61
	H-5Fb	4.08				
G (non-reducing end)	H-1G	4.86			C-1G	101.25
	H-2G	5.08			C-2G	75.40
	H-3G	3.92			C-3G	75.08
	H-4G	3.63				
	H-5Ga	3.38			C-5G	64.72
	H-5Gb	4.07				

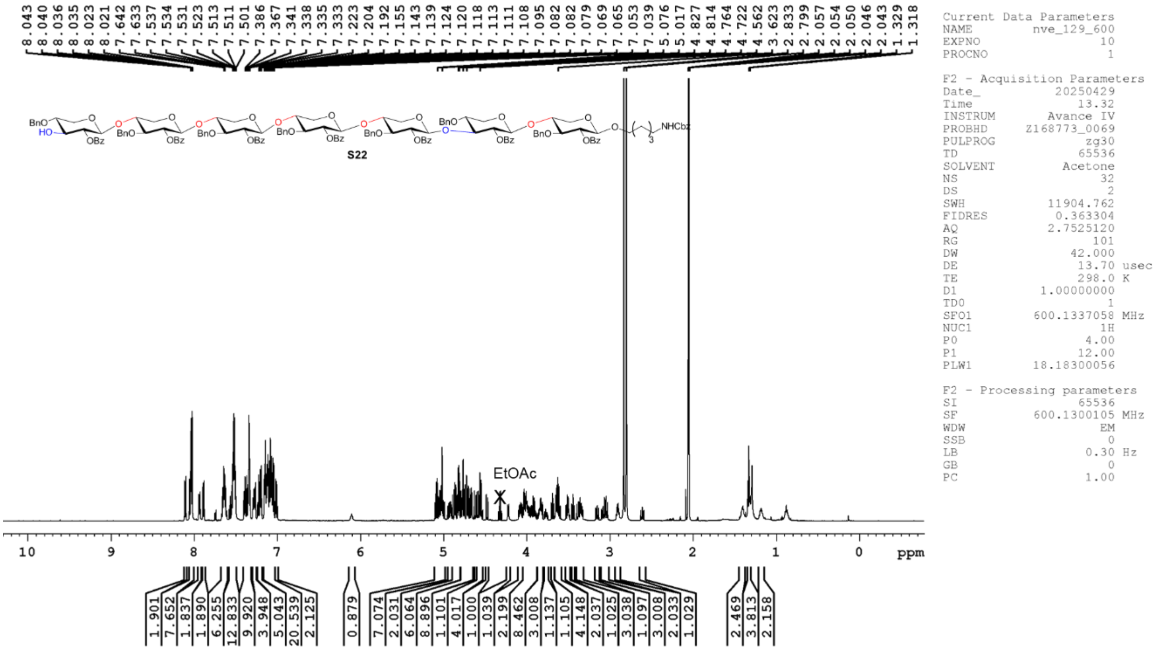

^{1}H NMR spectrum of compound **S22** (600 MHz, $(CD_3)_2CO$)

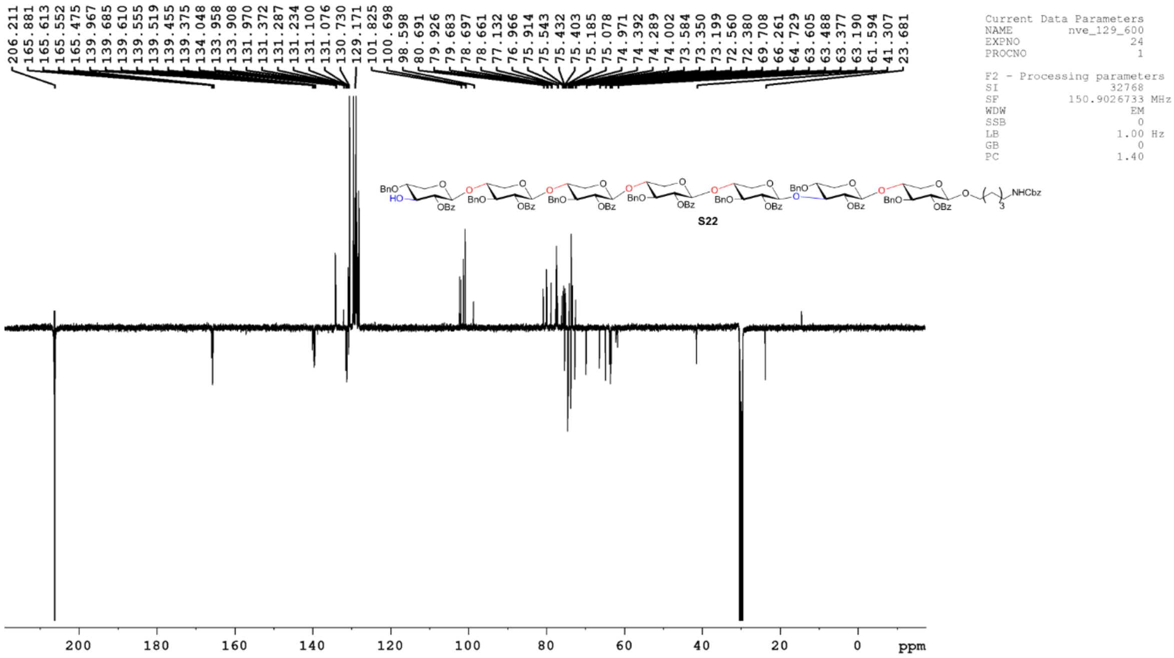

^{13}C APT NMR spectrum of compound **S22** (151 MHz, $(CD_3)_2CO$)

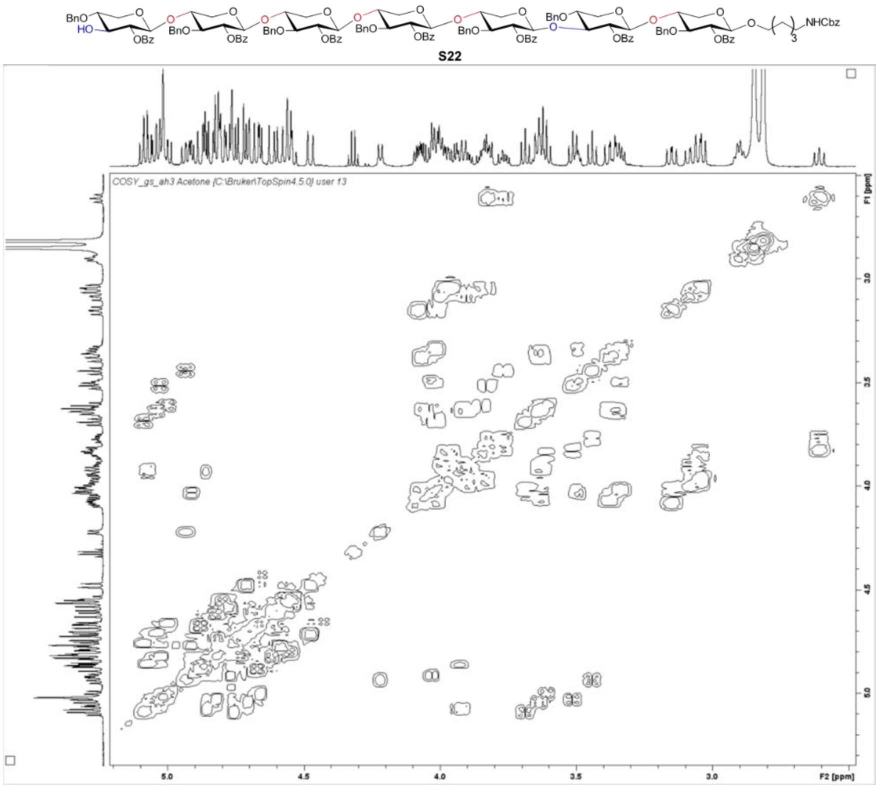

^{1}H-^{1}H COSY NMR spectrum of compound **S22** (600 MHz, $(CD_3)_2CO$)

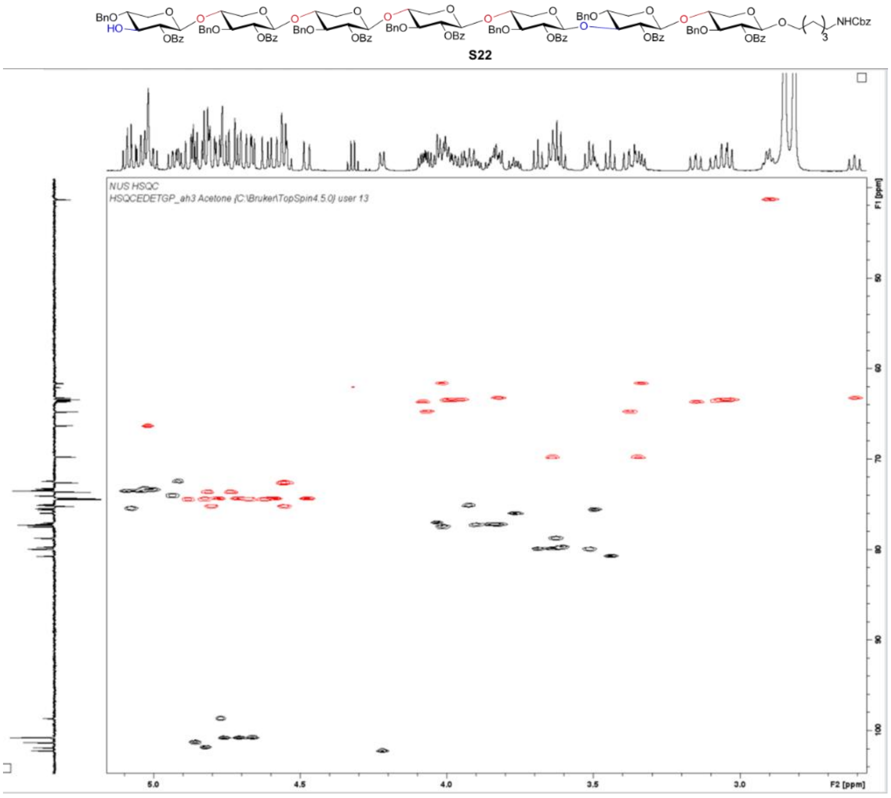

^{1}H-^{13}C HSQC NMR spectrum of compound **S22** (600/151 MHz, $(CD_3)_2CO$)

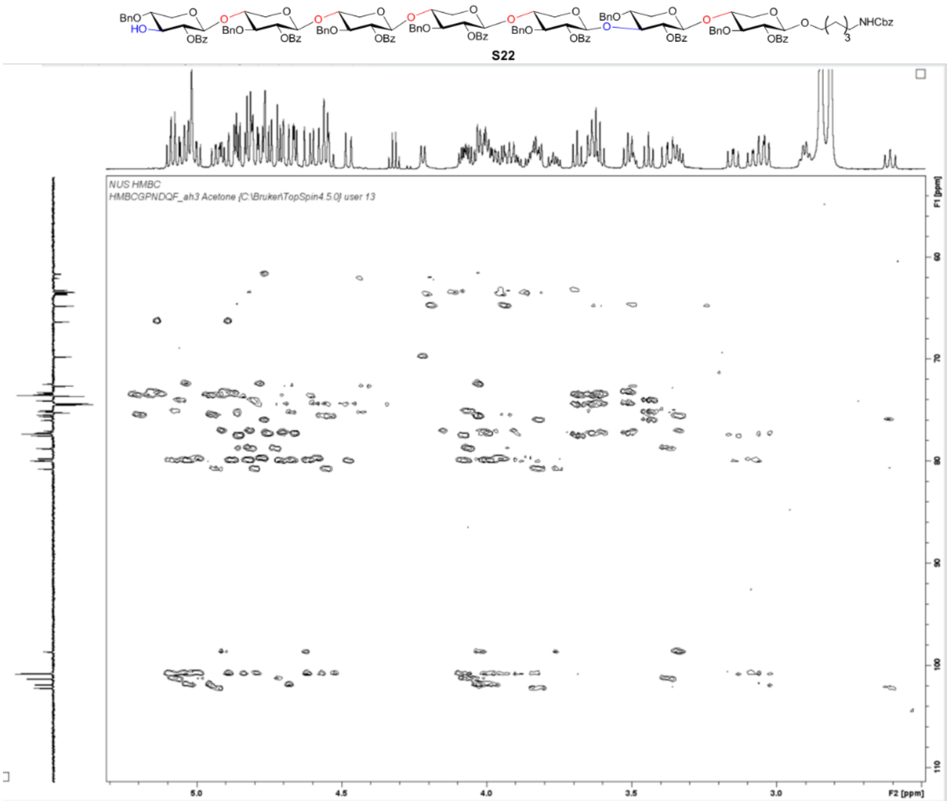

^1H-^{13}C HMBC NMR spectrum of compound **S22** (600/151 MHz, $(CD_3)_2CO$)

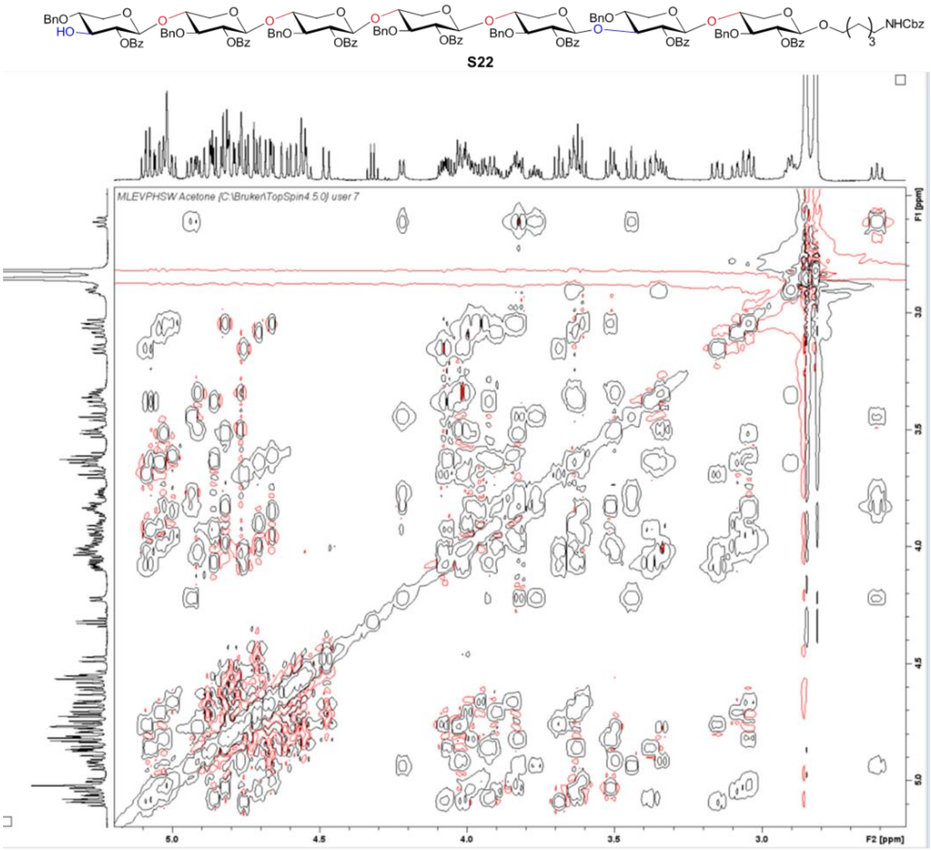

^1H-^1H TOCSY NMR spectrum of compound **S22** (600 MHz, $(CD_3)_2CO$)

Aminopentyl β-D-xylopyranosyl-(1→4)-β-D-xylopyranosyl-(1→4)-β-D-xylopyranosyl-(1→4)-β-D-xylopyranosyl-(1→4)-β-D-xylopyranosyl-(1→3)-β-D-xylopyranosyl-(1→4)-β-D-xylopyranoside (21)

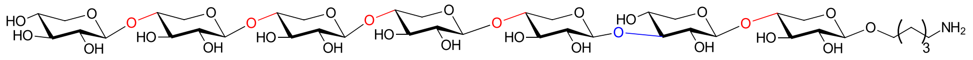

21

Experimental procedure: To a solution of protected MLX heptasaccharide **S22** (3.3 mg, 1.31 µmol) in THF (79 µL, 17 mM), a solution of NaOMe in MeOH (0.5 M, 46 µL, 23 µmol) was added at rt, and the reaction mixture was allowed to stir overnight. After confirmation of reaction completion (19 h) by TLC (R_f: 0.5 (MeOH/$CHCl_3$ = 1/15, v/v)), the reaction mixture was neutralized by the addition of IR-120 H^+ resin while slowly stirring the mixture. Then, the reaction mixture was filtered, and the filtrate was concentrated under reduced pressure to yield a partially-deprotected glassy crude, which was kept under high vacuum until usage in the next step without any further purification. To a solution of partially deprotected crude (1.31 µmol) in *t*-BuOH (0.26 mL), H_2O (0.13 mL), and AcOH (0.07 mL), unreduced 10% Pd/C (3.9 mg) was added and the reaction mixture was stirred in the H_2 reactor under a pressure of 8 bar H_2. After 72 h, the reaction progress was checked by MALDI/TOF-MS analysis of the reaction mixture. Then, the reaction mixture was filtered using a PTFE syringe filter (0.45 µm) and concentrated under reduced pressure to yield a crude product, which was purified using pre-packed C18 (500 mg, 3 mL) column chromatography with H_2O/ACN (100% to 80%, v/v, + 0.1% AcOH). The purified product was lyophilized to give MLX heptasaccharide **21** (1.09 mg, 75% yield over 2 steps) as a white amorphous foam.

^{1}H NMR (600 MHz, D_2O): δ 4.67 (d, J = 7.8 Hz, 1H, H-1F), 4.49-4.43 (m, 5H, H-1B, H-1C, H-1D, H-1E, H-1G), 4.40 (d, J = 7.8 Hz, 1H, H-1A), 4.12-4.03 (m, 5H, H-5F, H-5A), 4.01-3.93 (m, 2H, H-5G), 3.89-3.83 (m, 1H, OCH_2), 3.81-3.72 (m, 5H, H-4F, H-4A), 3.71-3.58 (m, 5H, OCH_2, H-4G), 3.57-3.51 (m, 5H, H-3F, H-3A), 3.46-3.39 (m, 2H, H-3G), 3.38-3.32 (m, 6H, H-5A, H-2F), 3.31-3.27 (m, 4H, H-5G), 3.26-3.22 (m, 2H, H-2A, H-2G), 2.98 (t, J = 7.5 Hz, 2H, CH_2-NH_2), 1.71-1.61 (m, 4H, CH_2), 1.47-1.40 (m, 2H, CH_2) ppm.

^{13}C NMR (151 MHz, D_2O): δ 103.83, 103.38, 102.43, 102.28, 102.25, 84.0, 77.01, 76.98, 76.96, 76.92, 76.19, 74.46, 74.24, 73.85, 73.56, 73.36, 73.28, 73.11, 70.8, 69.8, 68.2, 65.81, 65.48, 63.55, 63.50, 40.0, 28.8, 27.0, 22.7 ppm.

ESI-HRMS: m/z [M + Na]$^+$ calcd. for $C_{40}H_{69}NO_{29}Na$: 1050.3847; found 1050.3855.

NMR chemical shifts of selected ^{1}H and ^{13}C atoms in compound **21**:

**xylose ring**	**proton**	***δ* (ppm)**	**multiplicity**	***J* (Hz)**	**carbon**	***δ* (ppm)**
A (reducing end)	H-1A	4.40	d	7.8	C-1A	103.38
	H-2A	3.26				
	H-3A	3.54				
	H-4A	3.75				
	H-5Aa	3.37				
	H-5Ab	4.06				
F	H-1F	4.67	d	7.8	C-1F	103.83
	H-2F	3.36				
	H-3F	3.57				
	H-4F	3.79				
	H-5Fa	-			C-5F	63.5
	H-5Fb	4.09				
G	H-1G	4.44			C-1G	102.43
	H-2G	3.24				
	H-3G	3.41				
	H-4G	3.62				
	H-5Ga	3.30			C-5G	65.81
	H-5Gb	3.96				

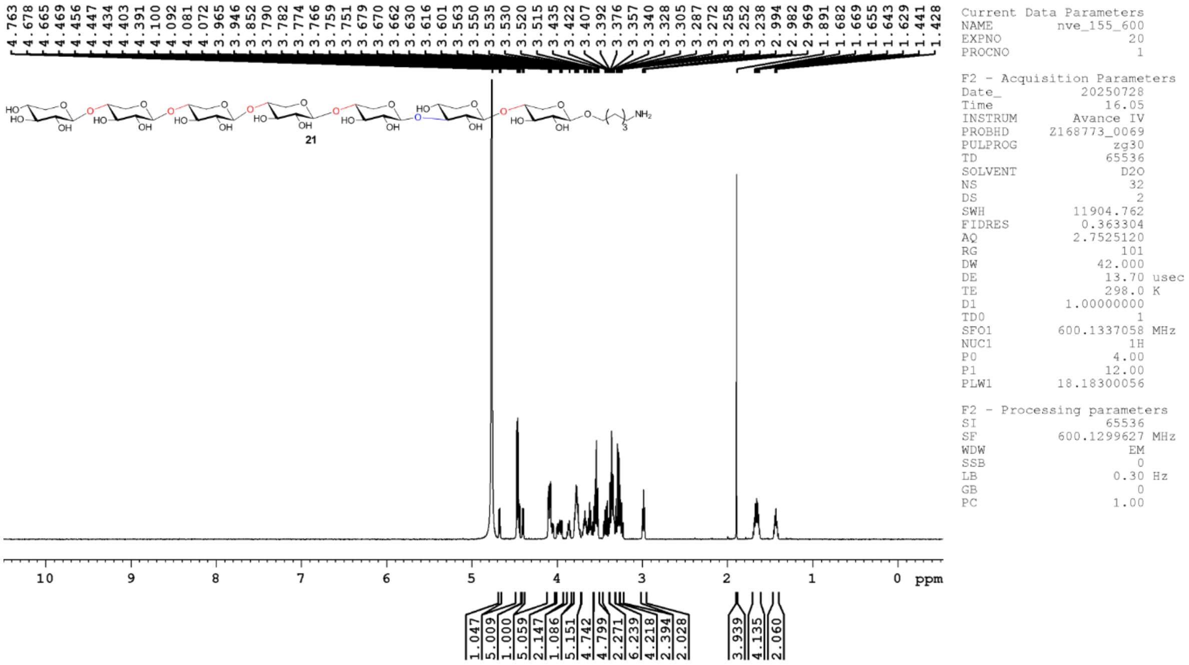

^{1}H NMR spectrum of compound **21** (600 MHz, D_2O)

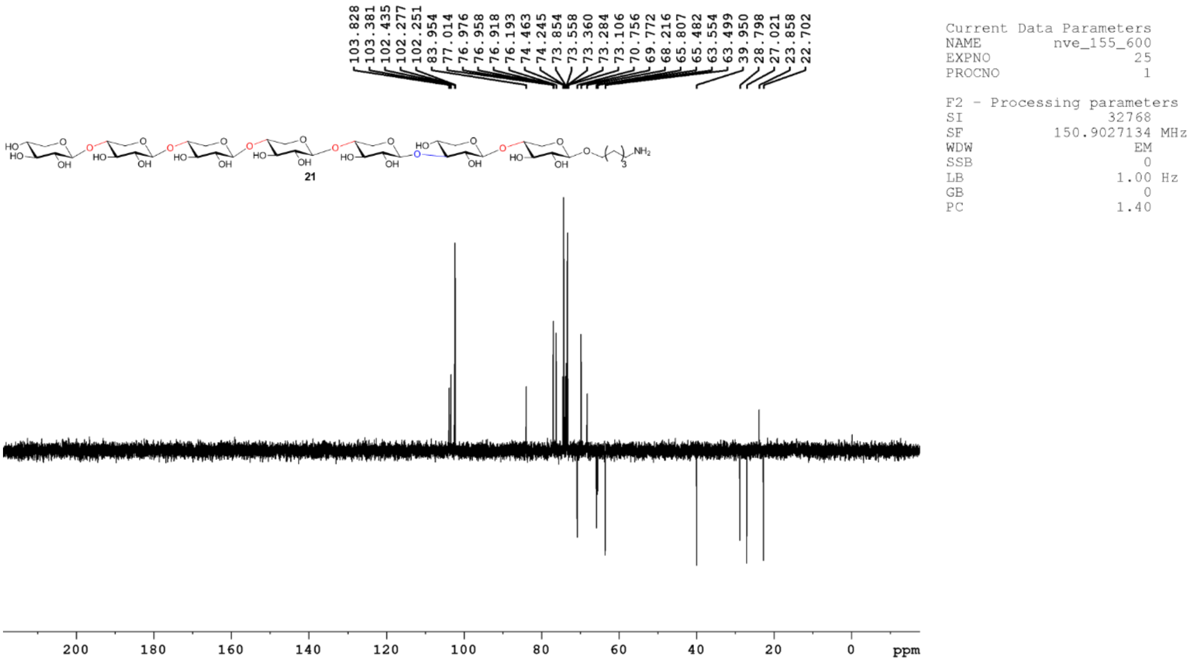

^{13}C APT NMR spectrum of compound **21** (151 MHz, D_2O)

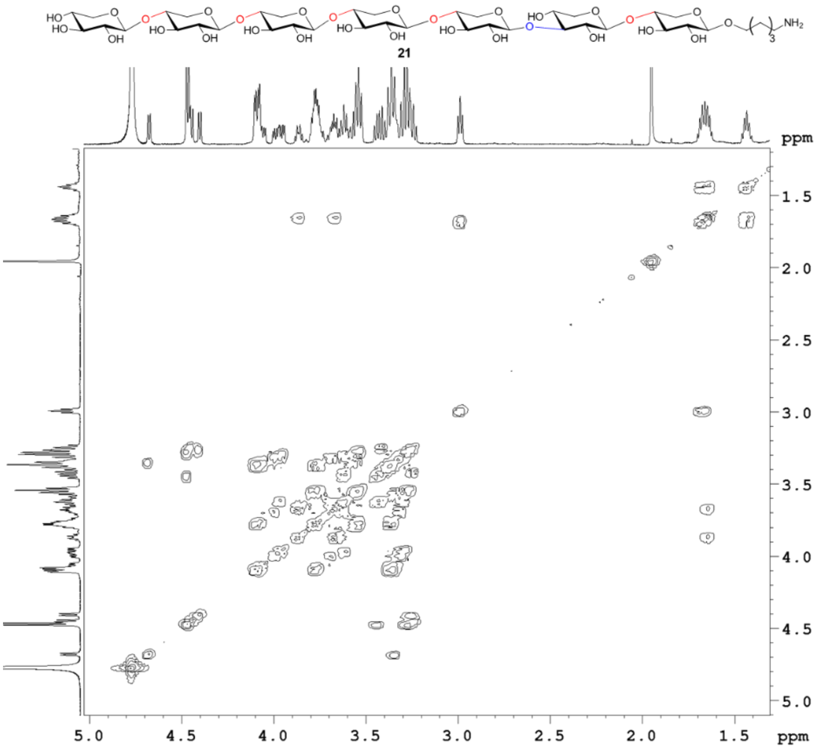

^{1}H-^{1}H COSY NMR spectrum of compound **21** (600 MHz, D_2O)

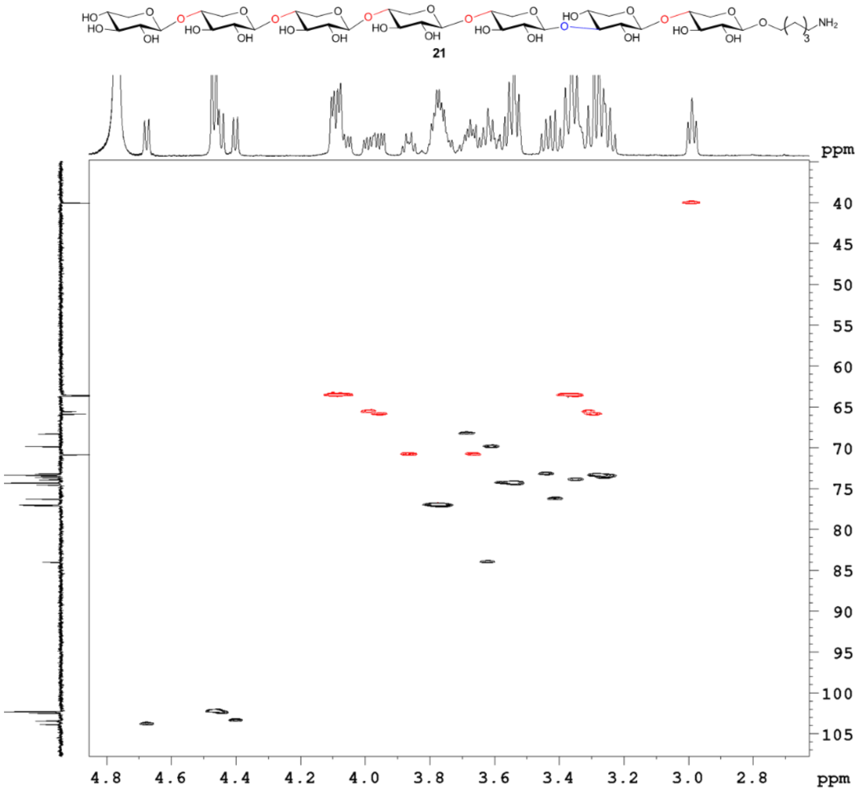

^{1}H-^{13}C HSQC NMR spectrum of compound **21** (600/151 MHz, D_2O)

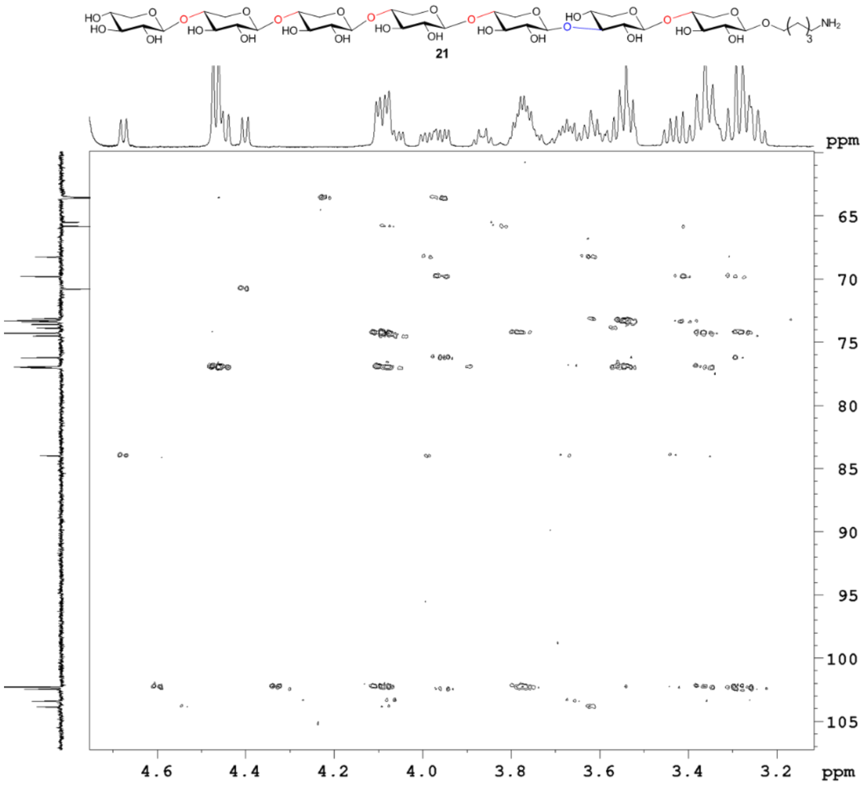

^{1}H-^{13}C HMBC NMR spectrum of compound **21** (600/151 MHz, D_2O)

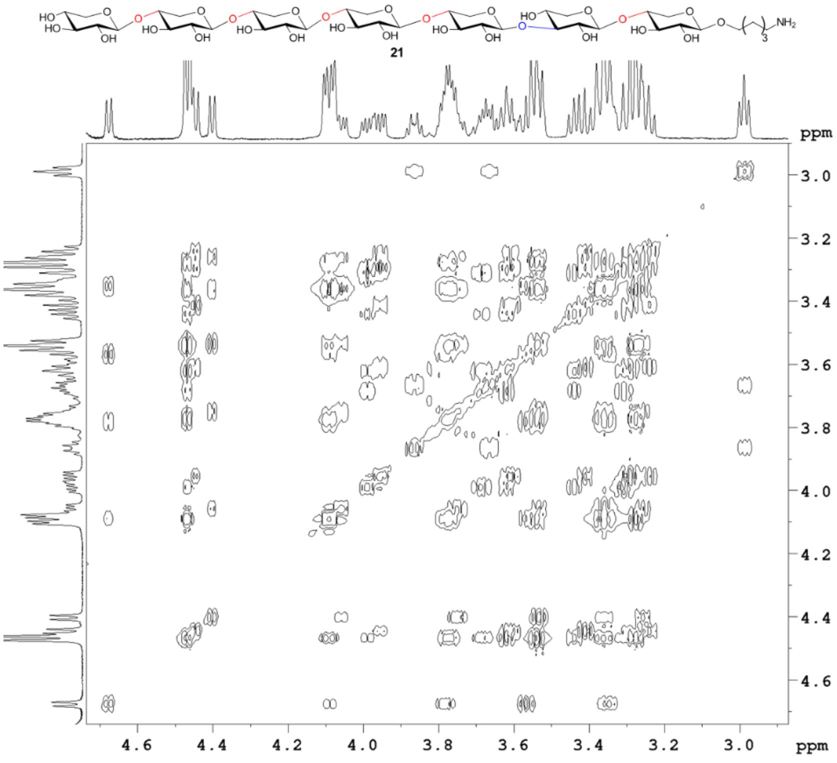

^{1}H-^{1}H TOCSY NMR spectrum of compound **21** (600 MHz, D_2O)

AGA of MLX octasaccharide S23

Benzyloxycarbonylaminopentyl 2-*O*-benzoyl-4-*O*-benzyl-β-D-xylopyranosyl-(1→4)-2-*O*-benzoyl-3-*O*-benzyl-β-D-xylopyranosyl-(1→4)-2-*O*-benzoyl-3-*O*-benzyl-β-D-xylopyranosyl-(1→3)-2-*O*-benzoyl-4-*O*-benzyl-β-D-xylopyranosyl-(1→4)-2-*O*-benzoyl-3-*O*-benzyl-β-D-xylopyranosyl-(1→4)-2-*O*-benzoyl-3-*O*-benzyl-β-D-xylopyranosyl-(1→3)-2-*O*-benzoyl-4-*O*-benzyl-β-D-xylopyranosyl-(1→4)-2-*O*-benzoyl-3-*O*-benzyl-β-D-xylopyranoside (**S23**)

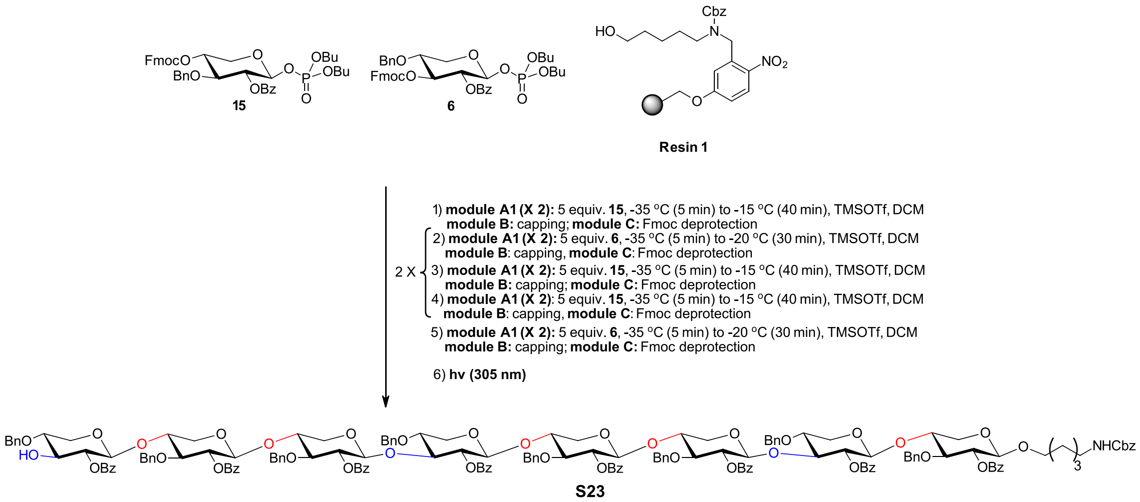

Experimental procedure: Linker-functionalized resin **1** (38 mg, 12.5 μmol) was placed in the synthesizer and synthesizer modules were applied as follows:

1) two cycles of module A1 (BB **15**, 47 mg, 0.0625 mmol, 5 equiv.) at –35 °C (5 min) to –15 °C (40 min), module B, and module C

2 × [

2) two cycles of module A1 (BB **6**, 47 mg, 0.0625 mmol, 5 equiv.) at –35 °C (5 min) to –20 °C (30 min), module B, and module C
3) two cycles of module A1 (BB **15**, 47 mg, 0.0625 mmol, 5 equiv.) at –35 °C (5 min) to –15 °C (40 min), module B, and module C
4) two cycles of module A1 (BB **15**, 47 mg, 0.0625 mmol, 5 equiv.) at –35 °C (5 min) to –15 °C (40 min), module B, and module C
]
5) two cycles of module A1 (BB **6**, 47 mg, 0.0625 mmol, 5 equiv.) at –35 °C (5 min) to –20 °C (30 min), module B, and module C.

Cleavage from the resin using UV irradiation at 305 nm in a continuous flow photoreactor afforded the crude product. Purification of the crude by normal phase HPLC using a preparative YMC-Diol column (EtOAc/hexanes = 1/9 to 1/1.5, v/v) gave protected MLX octasaccharide **S23** (5.3 mg, 15% yield over 17 steps) as a glassy solid and heptasaccharide as a deletion sequence (4.2 mg, 13% yield).

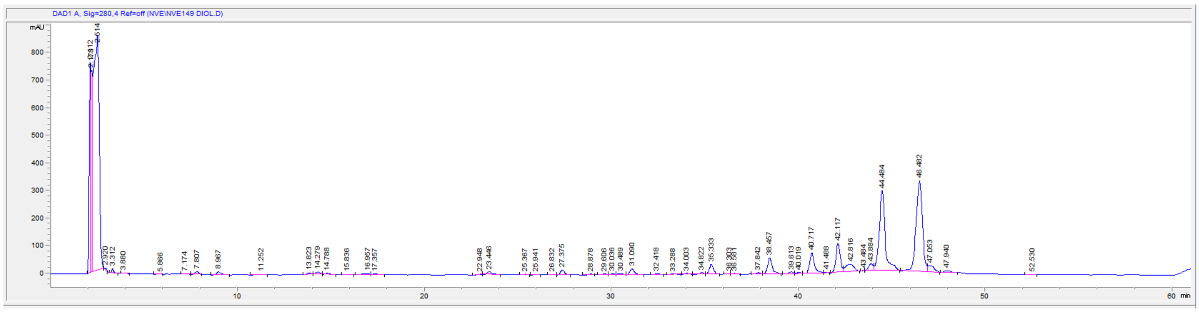

Analytical HPLC of the crude reaction mixture (diluted in 4 mL toluene, injection volume = 70 µL) was performed on a YMC-Diol column using a gradient of EtOAc in hexanes (60 min, flow rate 1 mL/min). MLX octasaccharide **S23** was eluted at 46.5 min.

^{1}H NMR (600 MHz, $(CD_3)_2CO$): *δ* 8.13-8.02 (m, 8H, Ar-H), 7.96-7.93 (m, 2H, Ar-H), 7.92-7.87 (m, 6H, Ar-H), 7.69-7.60 (m, 6H, Ar-H), 7.60-7.47 (m, 15H, Ar-H), 7.41-7.36 (m, 6H, Ar-H), 7.35-7.32 (m, 6H, Ar-H), 7.31-7.26 (m, 6H, Ar-H), 7.26-7.18 (m, 8H, Ar-H), 7.18-7.15 (m, 3H, Ar-H), 7.15-7.13 (m, 2H, Ar-H), 7.13-7.08 (m, 9H, Ar-H), 7.08-7.07 (m, 3H, Ar-H), 7.06-7.05 (m, 2H, Ar-H), 7.05-7.01 (m, 3H, Ar-H), 6.1 (br, 1H, N-H), 5.09-5.01 (m, 6H, H-2H, H-2C, H-2F, H-2G, CH_2-Cbz), 4.99 (dd, *J* = 7.5, 9.0 Hz, 1H, H-2D), 4.96-4.92 (m, 3H, H-2A, H-2E, H-2B), 4.88-4.71 (m, 13H, O-H, H-1H, H-1F, H-1G, H-1E, H-1B, CH_2-Ph), 4.71-4.65 (m, 2H, H-1C, CH_2-Ph), 4.63-4.54 (m, 6H, CH_2-Ph), 4.52-4.45 (m, 3H, H-1D, CH_2-Ph), 4.23 (d, *J* = 7.7 Hz, 1H, H-1A), 4.09-3.95 (m, 9H, H-3E, H-5H, H-3B, H-5E, H-5C, H-5F, H-5G, H-5B, H-4C), 3.91 (t, *J* = 8.9 Hz, 1H, H-3H), 3.88-3.76 (m, 6H, H-5D, H-4F, H-4G, H-5A, H-4D, H-4A), 3.70-3.60 (m, 3H, H-3C, OCH_2, H-4H), 3.55-3.49 (m, 4H, H-4E, H-3F, H-3G, H-4B), 3.48-3.43 (m, 2H, H-3A, H-3D), 3.39-3.31 (m, 4H, H-5E, H-5H, OCH_2, H-5B), 3.15-3.02 (m, 3H, H-5C, H-5F, H-5G), 2.94-2.88 (m, 2H, C*H_2*-NHCbz), 2.71 (dd, *J* = 9.8, 11.6 Hz, 1H, H-5D), 2.62 (dd, *J* = 10.1, 11.4 Hz, 1H, H-5A), 1.45-1.38 (m, 2H, CH_2), 1.36-1.31 (m, 2H, CH_2), 1.22-1.15 (m, 2H, CH_2) ppm.

^{13}C NMR (151 MHz, $(CD_3)_2CO$): *δ* 165.87, 165.62, 165.57, 165.49, 165.43, 139.96, 139.70, 139.66, 139.65, 139.55, 139.53, 139.41, 139.39, 134.11, 134.08, 134.06, 133.99, 133.96, 133.92, 133.88, 131.37, 131.29, 131.24, 131.14, 131.09, 130.77, 130.74, 130.71, 130.50, 130.48, 130.45, 130.43, 130.36, 129.53, 129.47, 129.46, 129.39, 129.33, 129.17, 129.05, 128.99, 128.97, 128.94, 128.79, 128.77, 128.72, 128.60, 128.54, 128.53, 128.29, 128.24, 128.22, 128.1, 128.01, 127.97, 102.2, 101.84, 101.75, 101.25, 101.03, 100.8, 98.98, 98.63, 80.70, 80.26, 79.99, 79.90, 79.76, 78.69, 78.65, 77.54, 77.37, 77.28, 77.04, 75.92, 75.74, 75.58, 75.43, 75.4, 75.19, 75.09, 74.99, 74.98, 74.33, 74.32, 74.01, 73.78, 73.58, 73.34, 73.21, 73.18, 72.75, 72.72, 72.58, 72.40, 69.7, 66.3, 64.7, 63.51, 63.48, 63.35, 63.25, 63.20, 62.1, 61.6, 41.31, 41.18, 23.7 ppm.

ESI-HRMS: m/z $[M + (NH_4)_2]^{2+}$ calcd. for $C_{165}H_{171}N_3O_{43}$: 1441.5654; found 1441.5673.

NMR chemical shifts of selected ^{1}H and ^{13}C atoms in compound **S23**:

**xylose ring**	**proton**	***δ* (ppm)**	**multiplicity**	***J* (Hz)**	**carbon**	***δ* (ppm)**
A (reducing end)	H-1A	4.23	d	7.7	C-1A	102.2
	H-2A	4.95			C-2A	74.01
	H-3A	3.46			C-3A	80.26
	H-4A	3.79				
	H-5Aa	2.62	dd	10.1, 11.4	C-5A	63.20
	H-5Ab	3.83				
B	H-1B	4.75			C-1B	98.98
	H-2B	4.93				
	H-3B	4.04			C-3B	77.37
	H-4B	3.50			C-4B	75.74
	H-5Ba	3.33			C-5B	62.1
	H-5Bb	3.99				
C	H-1C	4.70			C-1C	100.8
	H-2C	5.05				
	H-3C	3.67			C-3C	79.76
	H-4C	3.98			C-4C	77.37
	H-5Ca	3.12				
	H-5Cb	4.04				
D	H-1D	4.46			C-1D	101.03
	H-2D	4.99	dd	7.5, 9.0	C-2D	73.78
	H-3D	3.46			C-3D	80.26
	H-4D	3.79			C-4D	75.92
	H-5Da	2.71	dd	9.8, 11.6	C-5D	63.25
	H-5Db	3.86				
E	H-1E	4.79			C-1E	98.63
	H-2E	4.94				
	H-3E	4.06			C-3E	77.04
	H-4E	3.53				
	H-5Ea	3.37			C-5E	61.6
	H-5Eb	4.04				
F	H-1F	4.88-4.71	m			
	H-2F	5.09-5.01	m			
	H-3F	3.55-3.49	m			
	H-4F	3.88-3.76	m			
	H-5Fa	3.15-3.02	m			
	H-5Fb	4.09-3.95	m			
G	H-1G	4.88-4.71	m			
	H-2G	5.09-5.01	m			
	H-3G	3.55-3.49	m			
	H-4G	3.88-3.76	m			
	H-5Ga	3.15-3.02	m			
	H-5Gb	4.09-3.95	m			
H (non-reducing end)	H-1H	4.84			C-1H	101.25
	H-2H	5.07				
	H-3H	3.91	t	8.9	C-3H	74.98
	H-4H	3.62				
	H-5Ha	3.37			C-5H	64.7
	H-5Hb	4.06				

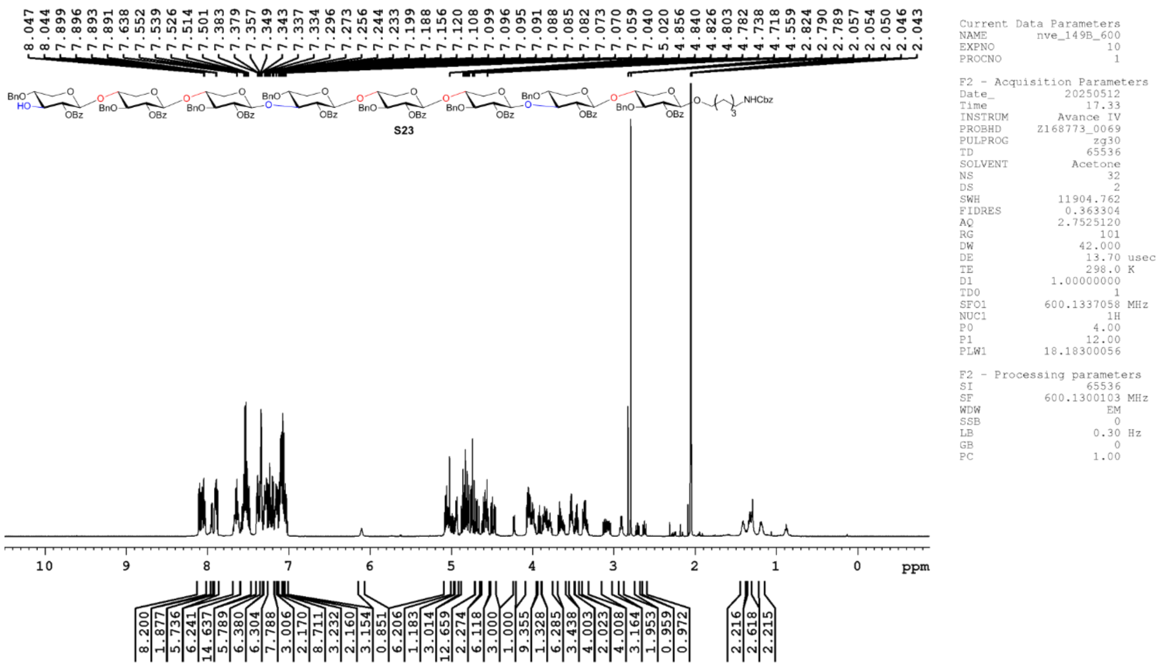

^{1}H NMR spectrum of compound **S23** (600 MHz, $(CD_3)_2CO$)

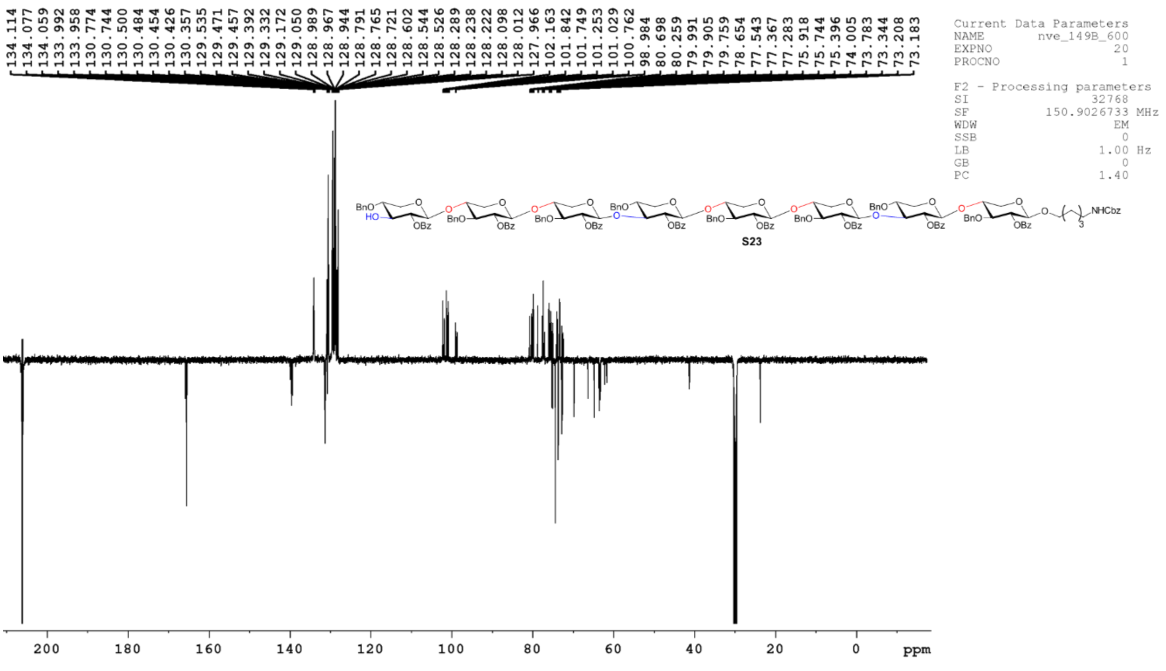

^{13}C APT NMR spectrum of compound **S23** (151 MHz, $(CD_3)_2CO$)

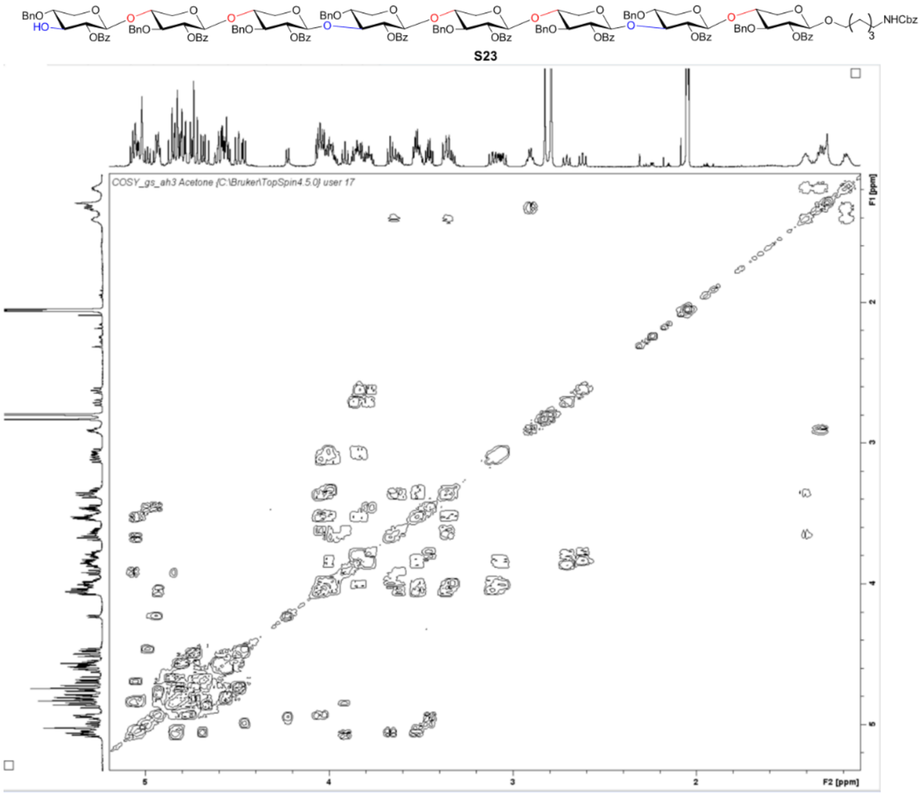

^{1}H-^{1}H COSY NMR spectrum of compound **S23** (600 MHz, $(CD_3)_2CO$)

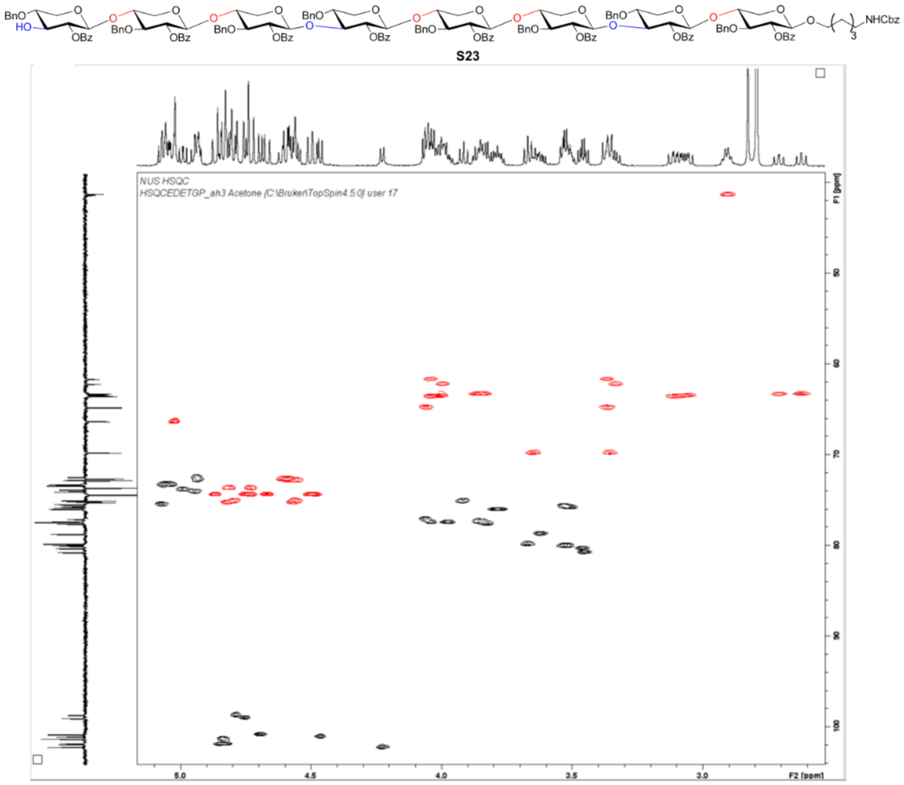

^{1}H-^{13}C HSQC NMR spectrum of compound **S23** (600/151 MHz, $(CD_3)_2CO$)

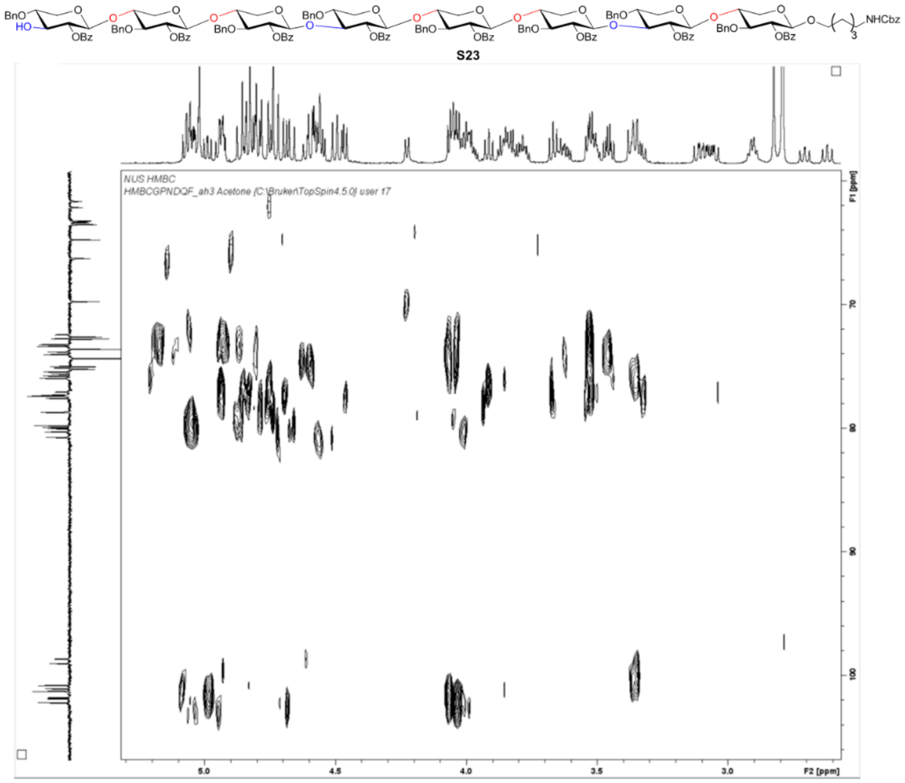

^1H-^{13}C HMBC NMR spectrum of compound **S23** (600/151 MHz, $(CD_3)_2CO$)

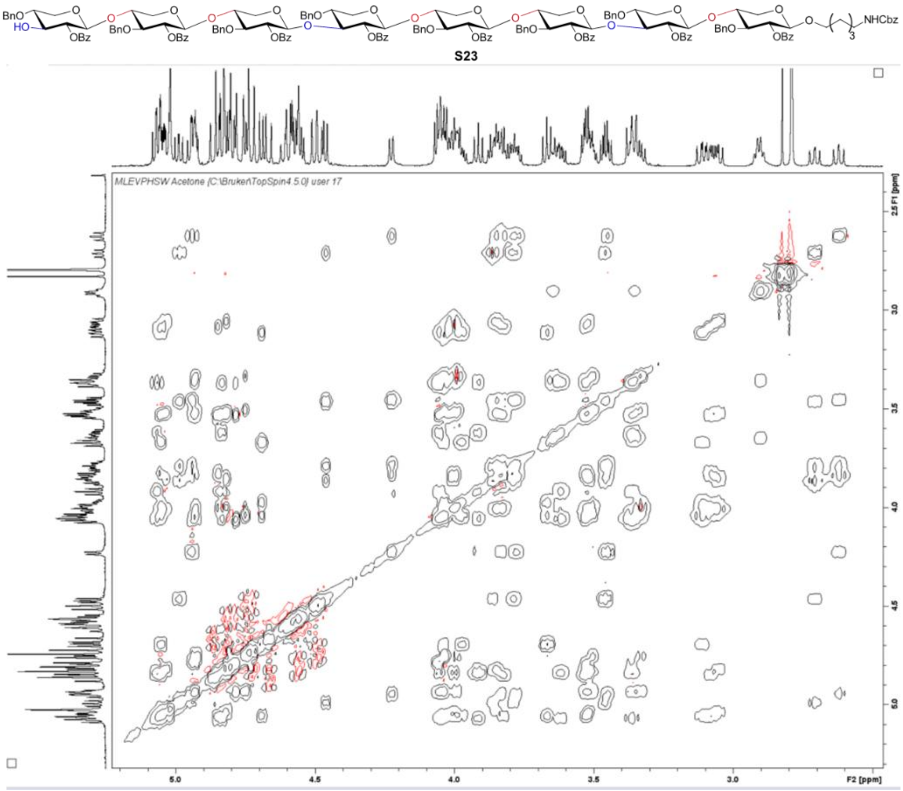

^1H-^1H TOCSY NMR spectrum of compound **S23** (600 MHz, $(CD_3)_2CO$)

Aminopentyl β-D-xylopyranosyl-(1→4)-β-D-xylopyranosyl-(1→4)-β-D-xylopyranosyl-(1→3)-β-D-xylopyranosyl-(1→4)-β-D-xylopyranosyl-(1→4)-β-D-xylopyranosyl-(1→3)-β-D-xylopyranosyl-(1→4)-β-D-xylopyranoside (22)

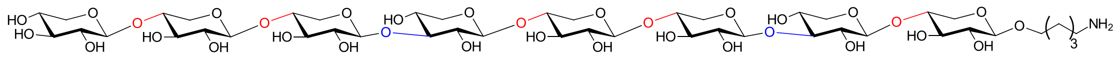

22

Experimental procedure: To a solution of protected MLX octasaccharide **S23** (5.1 mg, 1.8 µmol) in THF (107 µL, 17 mM), a solution of NaOMe in MeOH (0.5 M, 72 µL, 36 µmol) was added at rt, and the reaction mixture was allowed to stir overnight. After confirmation of reaction completion (24 h) by TLC (R_f: 0.34 (MeOH/$CHCl_3$ = 1/15, v/v)), the reaction mixture was neutralized by the addition of IR-120 H^+ resin while slowly stirring the mixture. Then, the reaction mixture was filtered, and the filtrate was concentrated under reduced pressure to yield a partially-deprotected glassy crude, which was kept under high vacuum until usage in the next step without any further purification. To a solution of partially deprotected crude starting material (1.8 µmol) in *t*-BuOH (0.36 mL), H_2O (0.18 mL), and AcOH (0.18 mL), unreduced 10% Pd/C (5.9 mg) was added and the reaction mixture was stirred in the H_2 reactor under a pressure of 8 bar H_2. After 68 h, the reaction progress was checked by MALDI/TOF-MS analysis of the reaction mixture. Then, the reaction mixture was filtered using a PTFE syringe filter (0.45 µm) and concentrated under reduced pressure to yield a crude product, which was purified using pre-packed C18 (500 mg, 3 mL) column chromatography with H_2O/ACN (100% to 80%, v/v, + 0.1% AcOH). The purified product was lyophilized to give MLX octasaccharide **22** (1.9 mg, 86% yield over 2 steps) as a white amorphous foam.

^{1}H NMR (600 MHz, D_2O): δ 4.69-4.66 (m, 2H), 4.49-4.43 (m, 5H), 4.40 (d, J = 7.8 Hz, 1H, H-1A), 4.12-4.03 (m, 5H, H-5A), 4.02-3.93 (m, 3H), 3.89-3.83 (m, 1H, OCH_2), 3.81-3.72 (m, 5H, H-4A), 3.72-3.65 (m, 3H, OCH_2), 3.65-3.59 (m, 3H), 3.59-3.54 (m, 3H), 3.54-3.51 (m, 2H, H-3A), 3.46-3.39 (m, 3H), 3.38-3.35 (m, 4H, H-5A), 3.35-3.32 (m, 3H), 3.32-3.28 (m, 3H), 3.28-3.22 (m, 4H, H-2A), 2.98 (t, J = 7.5 Hz, 2H, CH_2-NH_2), 1.71-1.61 (m, 4H, CH_2), 1.48-1.39 (m, 2H, CH_2) ppm.

^{13}C NMR (151 MHz, D_2O): δ 103.83, 103.38, 102.43, 102.26, 102.25, 83.95, 83.93, 77.01, 76.98, 76.96, 76.19, 74.46, 74.24, 73.85, 73.56, 73.36, 73.28, 73.11, 70.8, 69.8, 68.2, 65.80, 65.48, 63.56, 63.49, 40.0, 28.8, 27.0, 22.7 ppm.

ESI-HRMS: m/z $[M + H]^+$ calcd. for $C_{45}H_{78}NO_{33}$: 1160.4451; found 1160.4445.

NMR chemical shifts of selected ^{1}H and ^{13}C atoms in compound **22**:

**xylose ring**	**proton**	***δ* (ppm)**	**multiplicity**	***J* (Hz)**	**carbon**	***δ* (ppm)**
A (reducing end)	H-1A	4.40	d	7.8	C-1A	103.39
	H-2A	3.26				
	H-3A	3.53				
	H-4A	3.75				
	H-5Aa	3.36				
	H-5Ab	4.05				

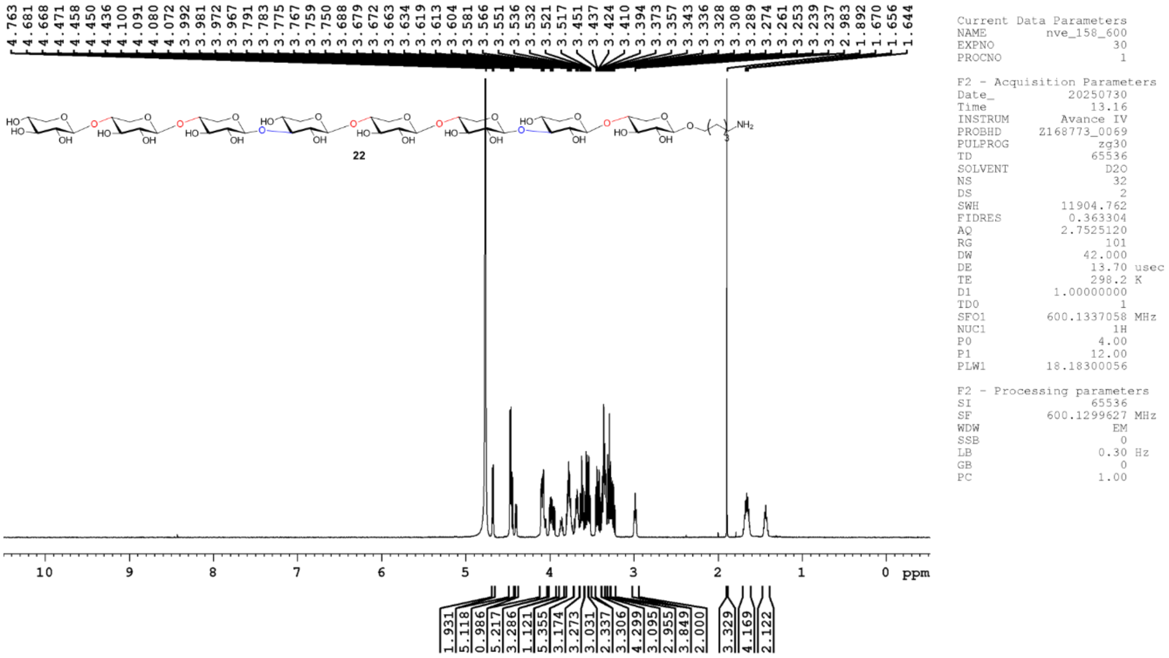

^{1}H NMR spectrum of compound **22** (600 MHz, D_2O)

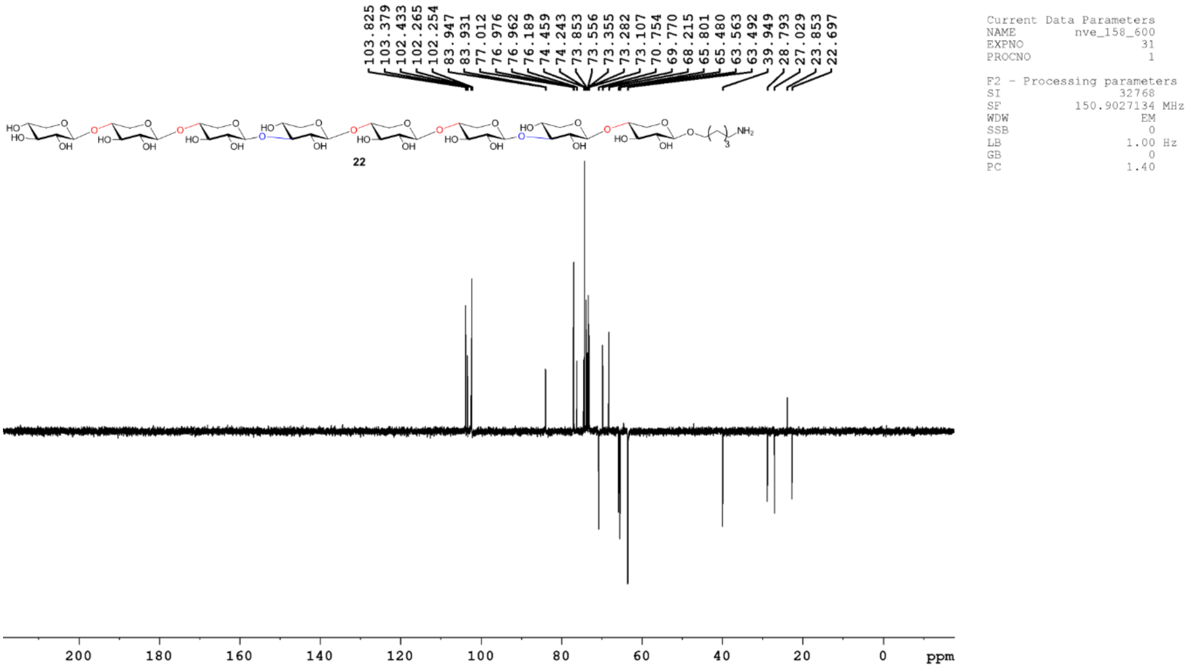

^{13}C DEPTQ135 NMR spectrum of compound **22** (151 MHz, D_2O)

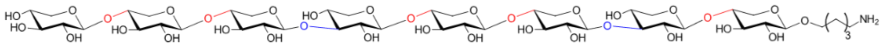

22

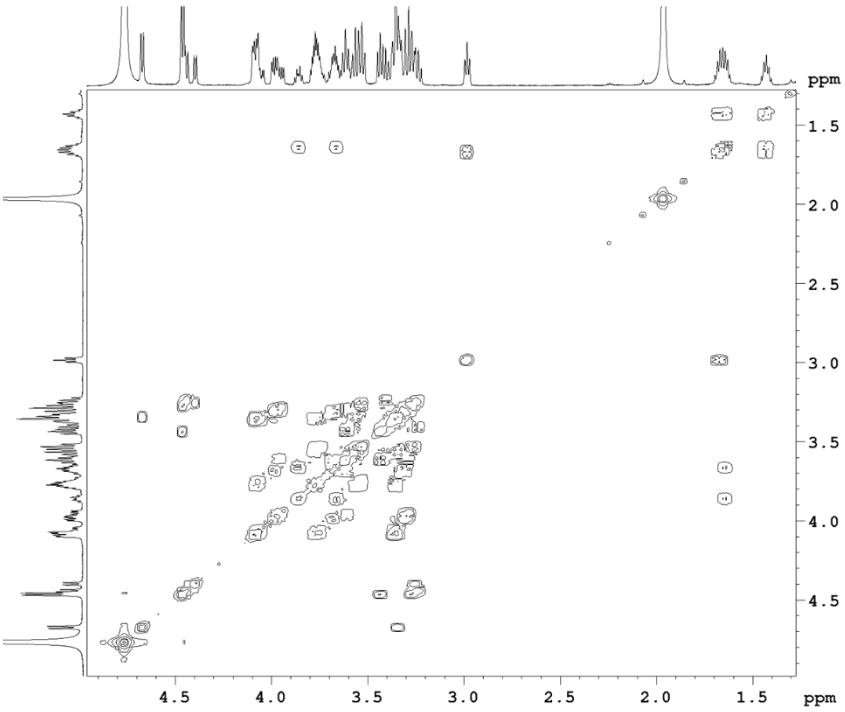

^{1}H-^{1}H COSY NMR spectrum of compound **22** (600 MHz, D_2O)

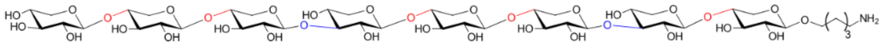

22

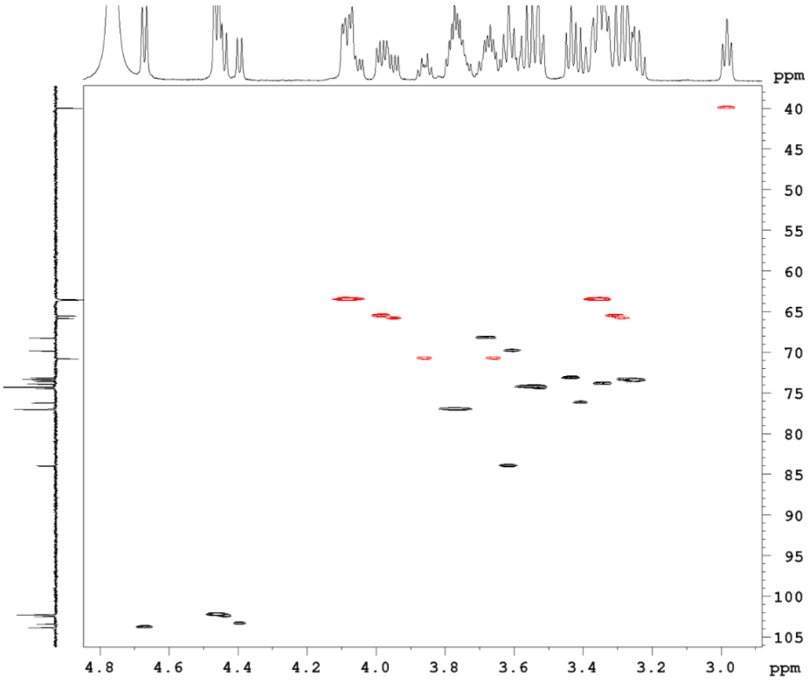

^{1}H-^{13}C HSQC NMR spectrum of compound **22** (600/151 MHz, D_2O)

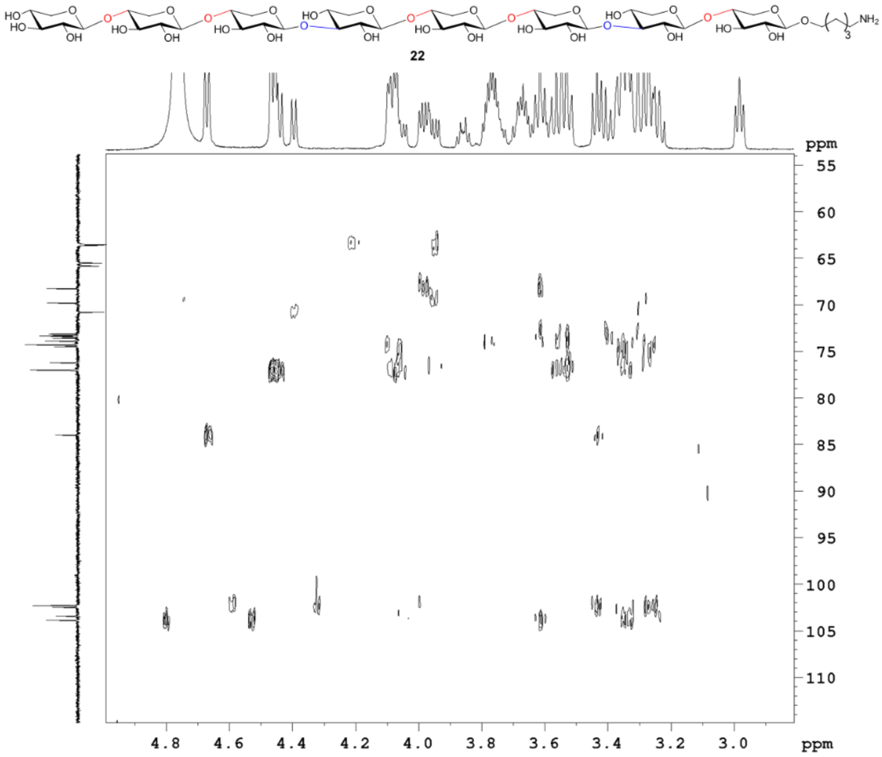

^{1}H-^{13}C HMBC NMR spectrum of compound **22** (600/151 MHz, D_2O)

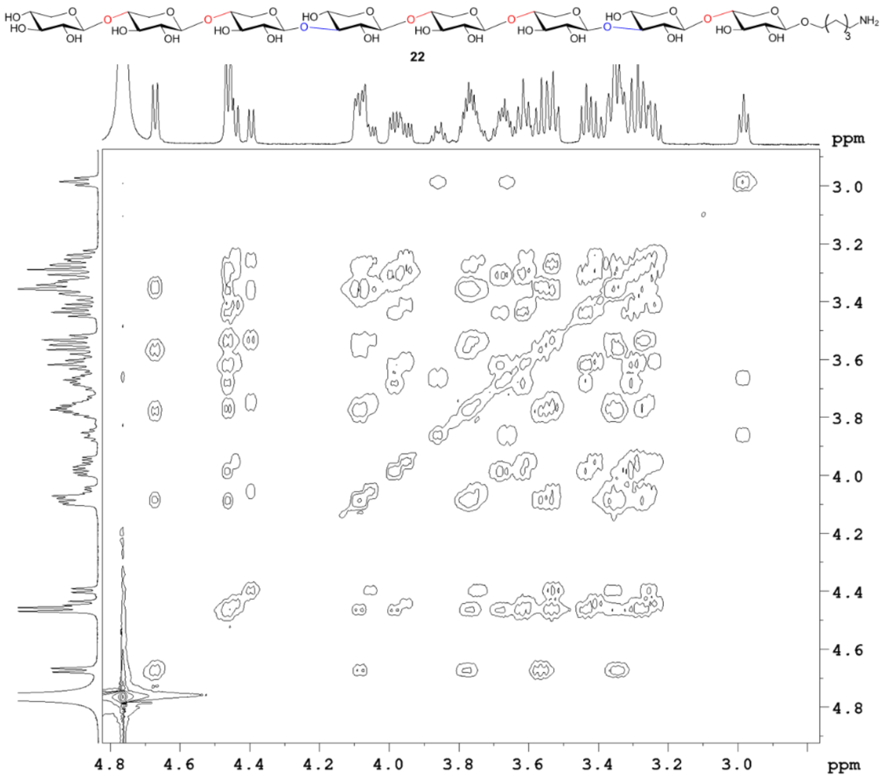

^{1}H-^{1}H TOCSY NMR spectrum of compound **22** (600 MHz, D_2O)

AGA of MLX nonasaccharide S24

Benzyloxycarbonylaminopentyl 2-*O*-benzoyl-3-*O*-benzyl-β-D-xylopyranosyl-(1→3)-2-*O*-benzoyl-4-*O*-benzyl-β-D-xylopyranosyl-(1→4)-2-*O*-benzoyl-3-*O*-benzyl-β-D-xylopyranosyl-(1→4)-2-*O*-benzoyl-3-*O*-benzyl-β-D-xylopyranosyl-(1→4)-2-*O*-benzoyl-3-*O*-benzyl-β-D-xylopyranosyl-(1→4)-2-*O*-benzoyl-3-*O*-benzyl-β-D-xylopyranosyl-(1→3)-2-*O*-benzoyl-4-*O*-benzyl-β-D-xylopyranosyl-(1→4)-2-*O*-benzoyl-3-*O*-benzyl-β-D-xylopyranosyl-(1→3)-2-*O*-benzoyl-4-*O*-benzyl-β-D-xylopyranoside (**S24**)

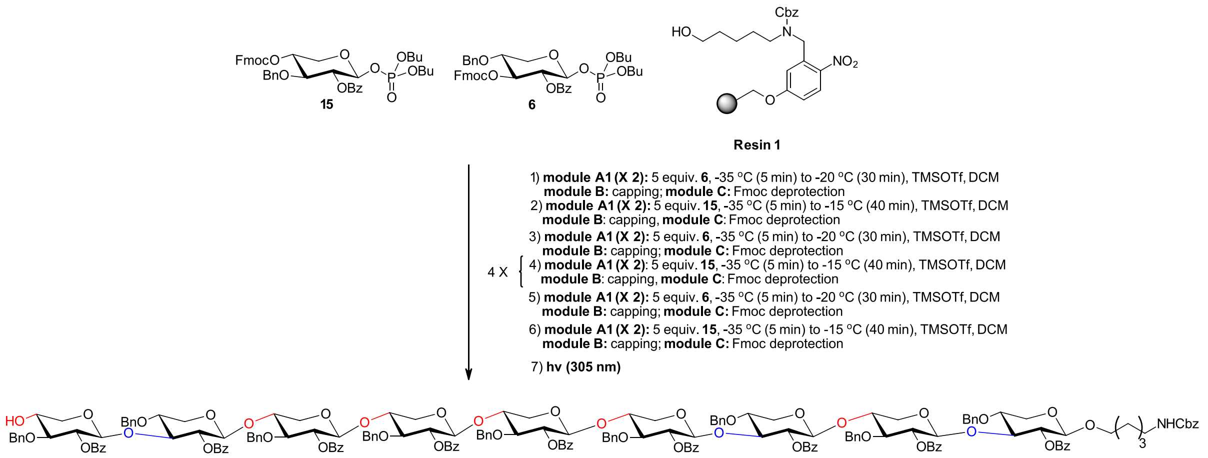

S24

Experimental procedure: Linker-functionalized resin **1** (38 mg, 12.5 μmol) was placed in the synthesizer and synthesizer modules were applied as follows:

1) two cycles of module A1 (BB **6**, 47 mg, 0.0625 mmol, 5 equiv.) at –35 °C (5 min) to –20 °C (30 min), module B, and module C
2) two cycles of module A1 (BB **15**, 47 mg, 0.0625 mmol, 5 equiv.) at –35 °C (5 min) to –15 °C (40 min), module B, and module C
3) two cycles of module A1 (BB **6**, 47 mg, 0.0625 mmol, 5 equiv.) at –35 °C (5 min) to –20 °C (30 min), module B, and module C

4 × [

4) two cycles of module A1 (BB **15**, 47 mg, 0.0625 mmol, 5 equiv.) at –35 °C (5 min) to –15 °C (40 min), module B, and module C
   ]
5) two cycles of module A1 (BB **6**, 47 mg, 0.0625 mmol, 5 equiv.) at –35 °C (5 min) to –20 °C (30 min), module B, and module C
6) two cycles of module A1 (BB **15**, 47 mg, 0.0625 mmol, 5 equiv.) at –35 °C (5 min) to –15 °C (40 min), module B, and module C.

Cleavage from the resin using UV irradiation at 305 nm in a continuous flow photoreactor afforded the crude product. Purification of the crude by normal phase HPLC using a preparative YMC-Diol column (EtOAc/hexanes = 1/9 to 1/1.5, v/v) gave protected MLX nonasaccharide **S24** (2.2 mg, 6% yield over 19 steps) as a glassy solid.

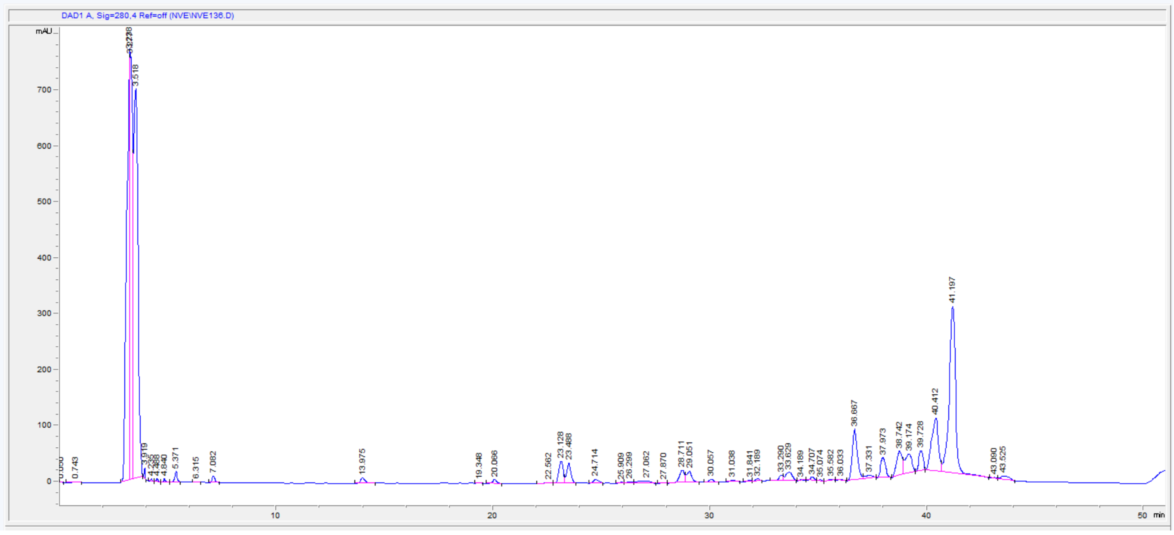

Analytical HPLC of the crude reaction mixture (diluted in 3 mL toluene, injection volume = 70 µL) was performed on a YMC-Small NP column using a gradient of EtOAc in hexanes (50 min, flow rate 1 mL/min). MLX nonasaccharide **S24** was eluted at 41.2 min.

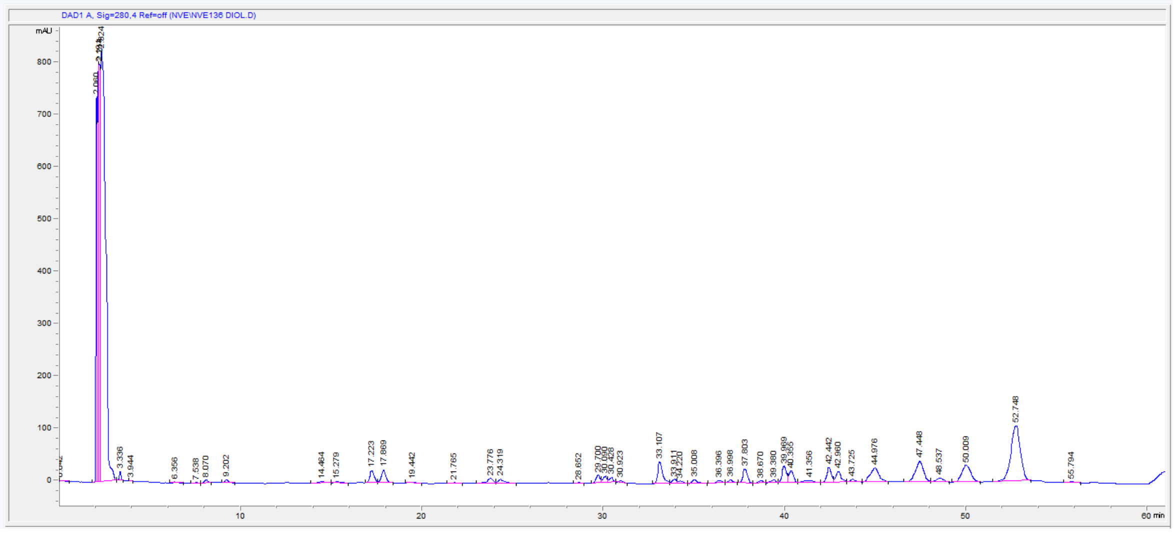

Analytical HPLC of the crude reaction mixture (diluted in 4 mL toluene, injection volume = 70 µL) was performed on a YMC-Diol column using a gradient of EtOAc in hexanes (60 min, flow rate 1 mL/min). MLX nonasaccharide **S24** was eluted at 52.75 min.

^{1}H NMR (600 MHz, $(CD_3)_2CO$): δ 8.11-8.08 (m, 2H, Ar-H), 8.06-7.99 (m, 8H, Ar-H), 7.90-7.83 (m, 6H, Ar-H), 7.78-7.75 (m, 2H, Ar-H), 7.70-7.61 (m, 5H, Ar-H), 7.61-7.49 (m, 12H, Ar-H), 7.49-7.42 (m, 4H, Ar-H), 7.40-7.36 (m, 8H, Ar-H), 7.35-7.25 (m, 14H, Ar-H), 7.25-7.17 (m, 6H, Ar-H), 7.17-7.00 (m, 28H, Ar-H), 6.09 (br, 1H, N-H), 5.15 (dd, J = 7.6, 9.1 Hz, 1H, H-2I), 5.09-4.95 (m, 9H, H-2E, H-2F, H-2H, H-2G, H-2D, H-1I, H-2B, CH_2-Cbz), 4.92 (dd, J = 6.7, 8.2 Hz, 1H, H-2A), 4.89 (dd, J = 5.4, 6.5 Hz, 1H, H-2C), 4.86-4.77 (m, 6H, H-1H, H-1D, CH_2-Ph), 4.74-4.68 (m, 6H, H-1E, H-1C, CH_2-Ph), 4.67-4.45 (m, 14H, H-1G, H-1B, O-H, H-1A, H-1F, CH_2-Ph), 4.42 (d, J = 11.2 Hz, 1H, CH_2Ph), 4.21 (t, J = 6.0 Hz, 1H, H-3H), 4.09 (dd, J = 3.9, 12.3 Hz, 1H, H-5H), 4.03-3.90 (m, 9H, H-3C, H-5E, H-3A, H-5I, H-5D, H-5G, H-5A, H-5C, H-5F), 3.89-3.79 (m, 6H, H-4E, H-4G, H-5B, H-4F, H-4I, H-4D), 3.77-3.72 (m, 1H, H-4B), 3.66-3.52 (m, 6H, H-3E, H-3G, H-4H, OCH_2, H-3I, H-4A), 3.51-3.41 (m, 4H, H-3D, H-3F, H-4C, H-5H), 3.37-3.25 (m, 5H, H-5A, H-5I, H-3B, H-5C, OCH_2), 3.11 (dd, J = 9.7,

11.6 Hz, 1H, H-5E), 3.08-3.0 (m, 2H, H-5G, H-5D), 2.91-2.85 (m, 2H, CH_2-NHCbz), 2.77-2.68 (m, 2H, H-5F, H-5B), 1.35-1.27 (m, 4H, CH_2), 1.13-1.07 (m, 2H, CH_2) ppm.

^{13}C NMR (151 MHz, $(CD_3)_2CO$): δ 165.75, 165.61, 165.58, 165.57, 165.50, 165.38, 139.84, 139.73, 139.62, 139.61, 139.51, 139.50, 139.46, 139.37, 134.14, 134.08, 134.05, 133.98, 133.91, 133.86, 133.58, 131.43, 131.16, 131.13, 131.07, 130.97, 130.86, 130.78, 130.76, 130.65, 130.57, 130.48, 130.46, 130.44, 130.4, 129.56, 129.47, 129.44, 129.34, 129.30, 129.27, 129.18, 129.10, 128.99, 128.95, 128.86, 128.78, 128.77, 128.75, 128.7, 128.67, 128.65, 128.62, 128.60, 128.54, 128.40, 128.23, 128.21, 128.09, 128.06, 128.03, 128.01, 127.93, 102.3, 101.76, 101.53, 101.40, 101.06, 100.78, 100.72, 99.1, 98.7, 83.34, 83.3, 80.49, 80.06, 79.90, 79.79, 79.76, 78.81, 77.50, 77.22, 77.16, 77.12, 76.67, 76.04, 75.74, 75.71, 75.63, 75.21, 74.8, 74.71, 74.41, 74.33, 74.24, 73.97, 73.93, 73.66, 73.53, 73.44, 73.37, 73.35, 73.09, 72.80, 72.75, 72.66, 72.49, 71.21, 71.11, 69.3, 66.77, 66.24, 63.57, 63.36, 63.31, 62.85, 62.12, 61.6, 41.3, 23.7 ppm.

ESI-HRMS: m/z $[M + (NH_4)_2]^{2+}$ calcd. for $C_{184}H_{189}N_3O_{48}$: 1604.1215; found 1604.1208.

NMR chemical shifts of selected ^{1}H and ^{13}C atoms in compound **S24**:

**xylose ring**	**proton**	***δ* (ppm)**	**multiplicity**	***J* (Hz)**	**carbon**	***δ* (ppm)**
A (reducing end)	H-1A	4.50			C-1A	101.40
	H-2A	4.92	dd	6.7, 8.2	C-2A	73.66
	H-3A	3.99				
	H-4A	3.54			C-4A	76.67
	H-5Aa	3.34			C-5A	63.36
	H-5Ab	3.92				
B	H-1B	4.65			C-1B	101.53
	H-2B	4.97			C-2B	73.53
	H-3B	3.33			C-3B	80.06
	H-4B	3.77-3.72	m		C-4B	76.04
	H-5Ba	2.71			C-5B	62.85
	H-5Bb	3.86				
C	H-1C	4.69			C-1C	99.1
	H-2C	4.89	dd	5.4, 6.5	C-2C	72.80
	H-3C	4.01				
	H-4C	3.45				
	H-5Ca	3.29			C-5C	62.12
	H-5Cb	3.92				
D	H-1D	4.79			C-1D	101.76
	H-2D	4.99			C-2D	73.09
	H-3D	3.49			C-3D	79.76
	H-4D	3.81			C-4D	77.12
	H-5Da	3.03				
	H-5Db	3.98				
E	H-1E	4.73			C-1E	100.72
	H-2E	5.06				
	H-3E	3.63			C-3E	79.79
	H-4E	3.88			C-4E	77.50
	H-5Ea	3.11	dd	9.7, 11.6	C-5E	63.57
	H-5Eb	4.00				
F	H-1F	4.49			C-1F	101.06
	H-2F	5.04				
	H-3F	3.47				
	H-4F	3.85				
	H-5Fa	2.74				
	H-5Fb					
G	H-1G	4.66			C-1G	100.78
	H-2G	5.00				
	H-3G	3.63				
	H-4G	3.87				
	H-5Ga	3.05				
	H-5Gb	3.96				
H	H-1H	4.86			C-1H	98.7
	H-2H	5.01				
	H-3H	4.21	t	6.0		
	H-4H	3.60			C-4H	75.63
	H-5Ha	3.43			C-5H	61.6
	H-5Hb	4.09	dd	3.9, 12.3		
I (non-reducing end)	H-1I	4.99			C-1I	102.3
	H-2I	5.15	dd	7.6, 9.1	C-2I	73.93

	H-3I	3.57			C-3I	83.3
	H-4I	3.85				
	H-5Ia	3.33			C-5I	66.77
	H-5Ib	3.98				

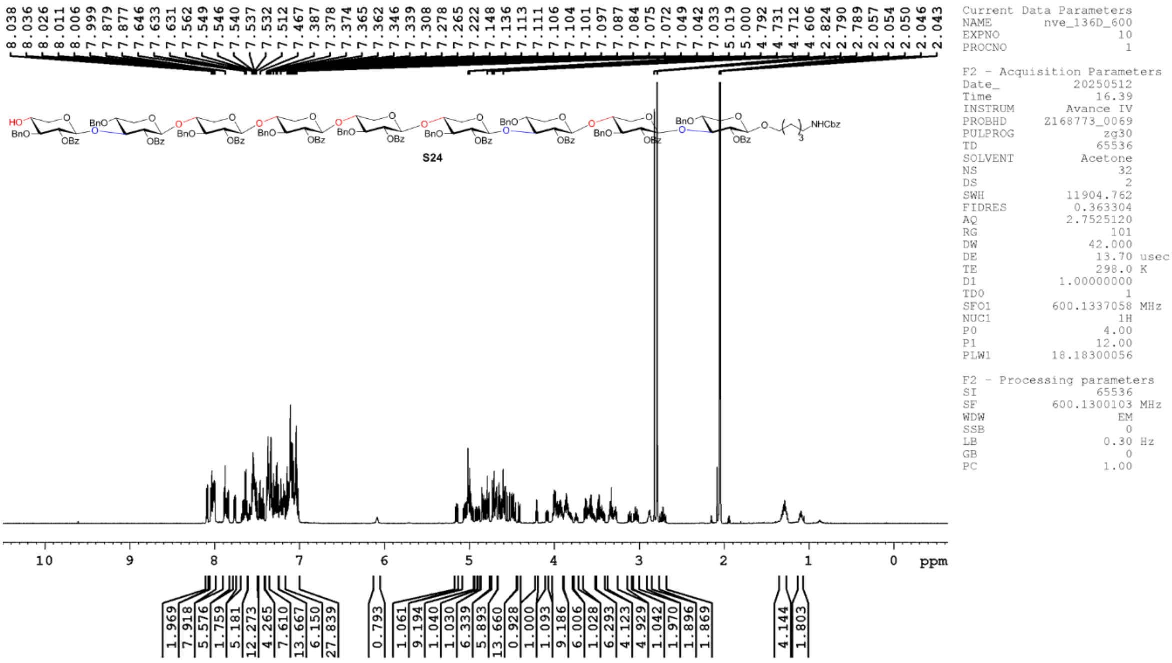

^{1}H NMR spectrum of compound **S24** (600 MHz, $(CD_3)_2CO$)

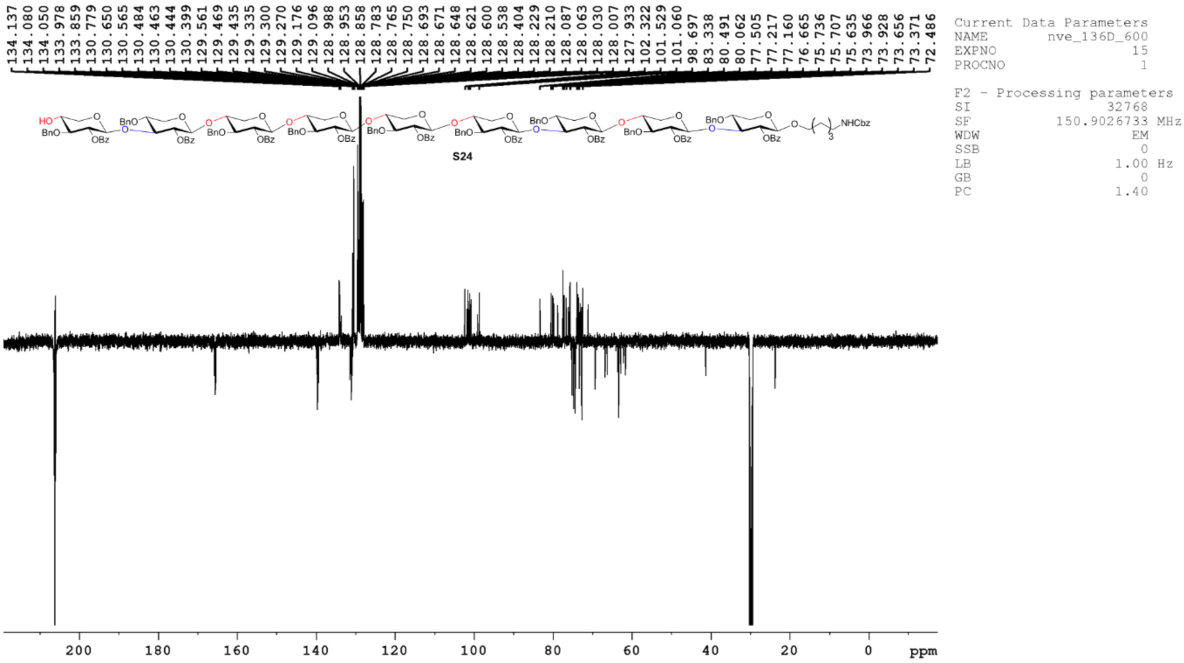

^{13}C APT NMR spectrum of compound **S24** (151 MHz, $(CD_3)_2CO$)

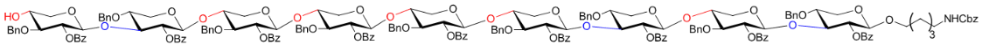

S24

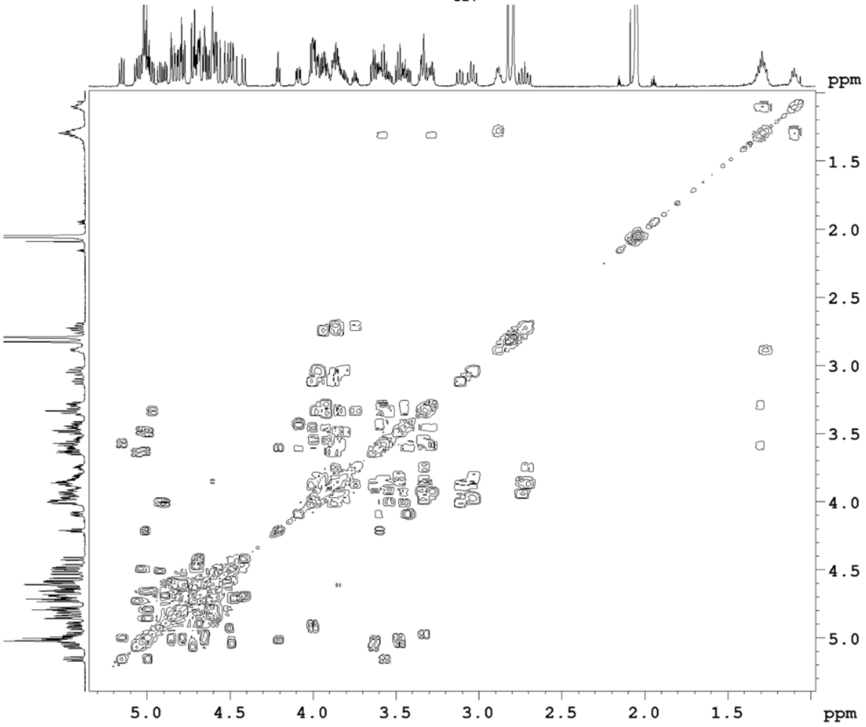

^{1}H-^{1}H COSY NMR spectrum of compound **S24** (600 MHz, $(CD_3)_2CO$)

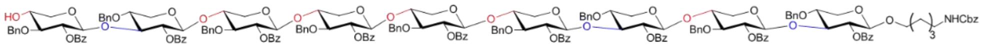

S24

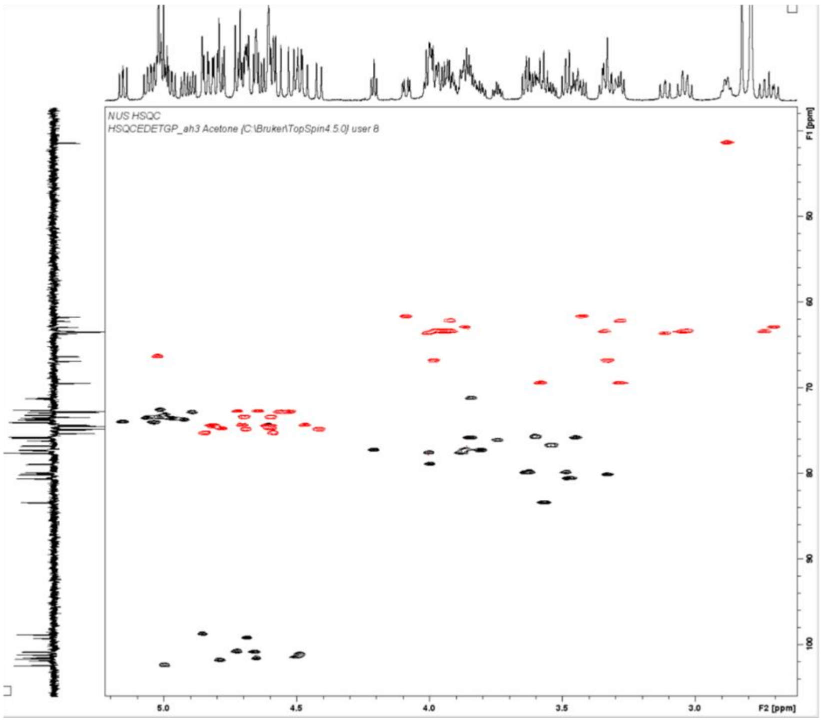

^{1}H-^{13}C HSQC NMR spectrum of compound **S24** (600/151 MHz, $(CD_3)_2CO$)

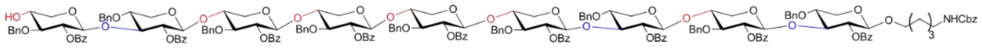

S24

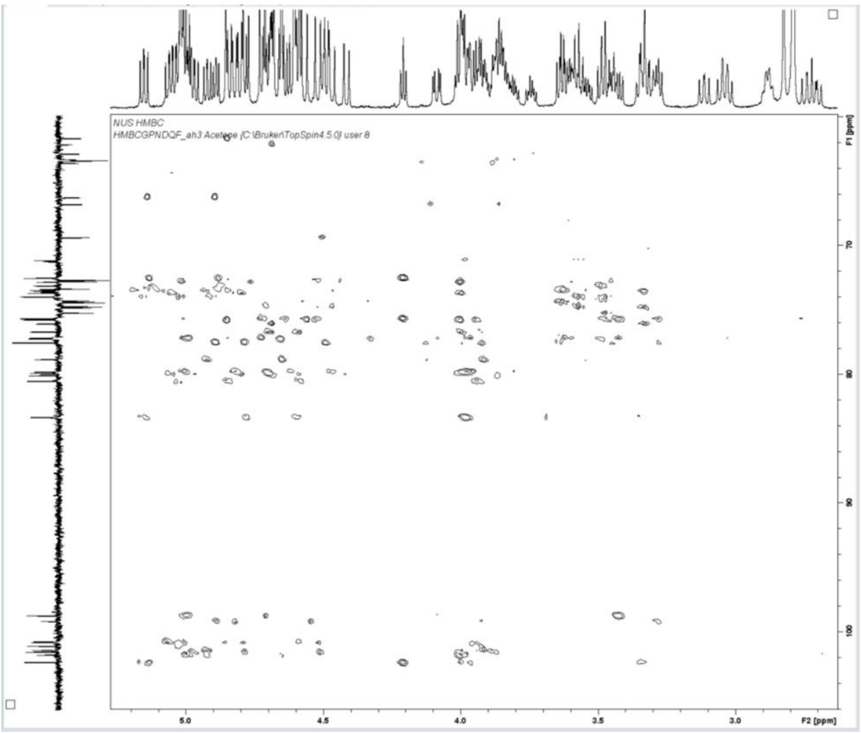

^{1}H-^{13}C HMBC NMR spectrum of compound **S24** (600/151 MHz, $(CD_3)_2CO$)

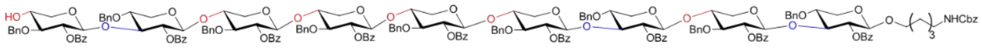

S24

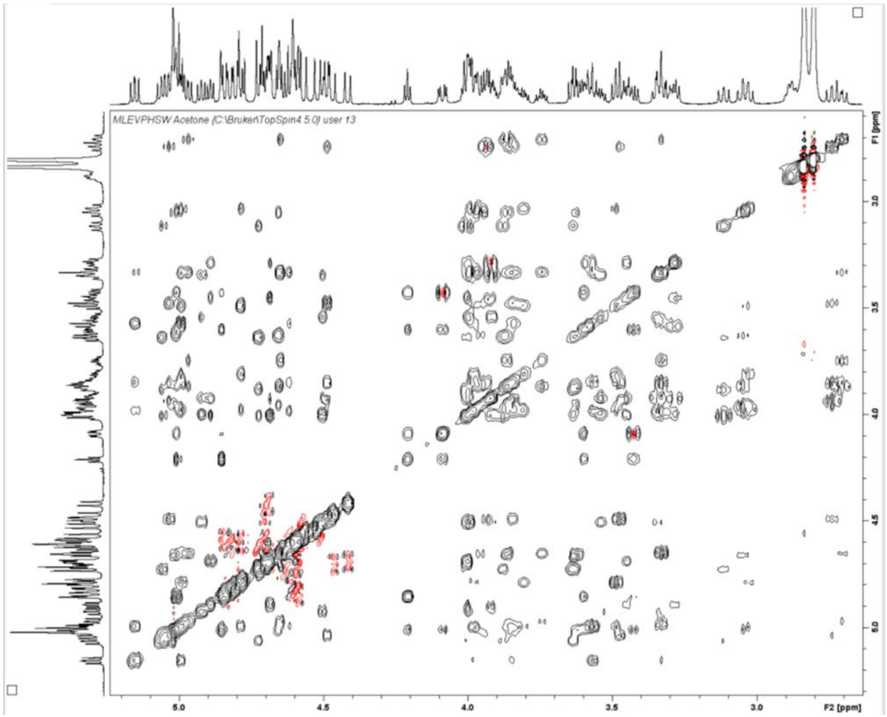

^{1}H-^{1}H TOCSY NMR spectrum of compound **S24** (600 MHz, $(CD_3)_2CO$)

Aminopentyl β-D-xylopyranosyl-(1→3)-β-D-xylopyranosyl-(1→4)-β-D-xylopyranosyl-(1→4)-β-D-xylopyranosyl-(1→4)-β-D-xylopyranosyl-(1→4)-β-D-xylopyranosyl-(1→3)-β-D-xylopyranosyl-(1→4)-β-D-xylopyranosyl-(1→3)-β-D-xylopyranoside (23)

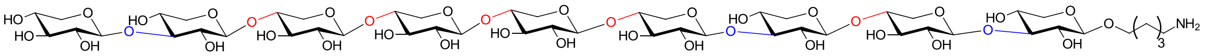

23

Experimental procedure: To a solution of protected MLX nonasaccharide **S24** (2.2 mg, 0.7 μmol) in THF (42 μL, 17 mM), a solution of NaOMe in MeOH (0.5 M, 31.2 μL, 16 μmol) was added at rt, and the reaction mixture was allowed to stir overnight. After confirmation of reaction completion (26 h) by TLC (R_f: 0.42 (MeOH/$CHCl_3$ = 1/15, v/v)), the reaction mixture was neutralized by the addition of IR-120 H^+ resin while slowly stirring the mixture. Then, the reaction mixture was filtered, and the filtrate was concentrated under reduced pressure to yield a partially-deprotected glassy crude, which was kept under high vacuum until usage in the next step without any further purification. To a solution of partially deprotected crude (0.7 μmol) in *t*-BuOH (0.14 mL), H_2O (70 μL), and AcOH (70 μL), unreduced 10% Pd/C (3.6 mg) was added and the reaction mixture was stirred in the H_2 reactor under a pressure of 8 bar H_2. After 48 h, the reaction progress was checked by the MALDI/TOF-MS analysis of the reaction mixture. Then, the reaction mixture was filtered using a PTFE syringe filter (0.45 μm) and concentrated under reduced pressure to yield a crude product, which was purified using pre-packed C18 (500 mg, 3 mL) column chromatography with H_2O/ACN (100% to 80%, v/v, + 0.1% AcOH). The purified product was lyophilized to give MLX nonasaccharide **23** (0.83 mg, 84% yield over 2 steps) as a white amorphous foam.

^{1}H NMR (600 MHz, D_2O): δ 4.69-4.63 (m, 3H), 4.49-4.44 (m, 5H), 4.41 (d, J = 7.8 Hz, 1H, H-1A), 4.12-4.06 (m, 5H), 4.02-3.93 (m, 4H, H-5A), 3.89-3.84 (m, 1H, OCH_2), 3.81-3.74 (m, 5H), 3.72-3.64 (m, 4H, H-4A, OCH_2), 3.64-3.59 (m, 4H, H-3A), 3.58-3.51 (m, 5H), 3.46-3.40 (m, 4H, H-2A), 3.39-3.25 (m, 15H, H-5A, H-5B, H-5C, H-5D, H-5E, H-5F, H-5G, H-5H, H-5I), 2.98 (t, J = 7.4 Hz, 2H, CH_2-NH_2), 1.71-1.61 (m, 4H, CH_2), 1.47-1.39 (m, 2H, CH_2) ppm.

^{13}C NMR (151 MHz, D_2O): δ 104.0, 103.89, 103.83, 103.28, 102.3, 84.40, 84.04, 83.94, 77.03, 76.98, 76.92, 76.21, 74.24, 73.95, 73.88, 73.86, 73.28, 73.12, 73.08, 70.75, 69.81, 68.26, 68.24, 68.22, 65.75, 65.47, 65.37, 63.55, 63.52, 63.49, 40.0, 28.8, 27.0, 22.7 ppm.

ESI-HRMS: m/z [M + H]$^+$ calcd. for $C_{50}H_{86}NO_{37}$: 1292.4873; found 1292.4889.

NMR chemical shifts of selected ^{1}H and ^{13}C atoms in compound **23**:

xylose ring	proton	δ (ppm)	multiplicity	J (Hz)	carbon	δ (ppm)
A (reducing end)	H-1A	4.41	d	7.8	C-1A	103.28
	H-2A	3.42				
	H-3A	3.63			C-3A	84.38
	H-4A	3.67				
	H-5Aa	3.32				
	H-5Ab	3.96				

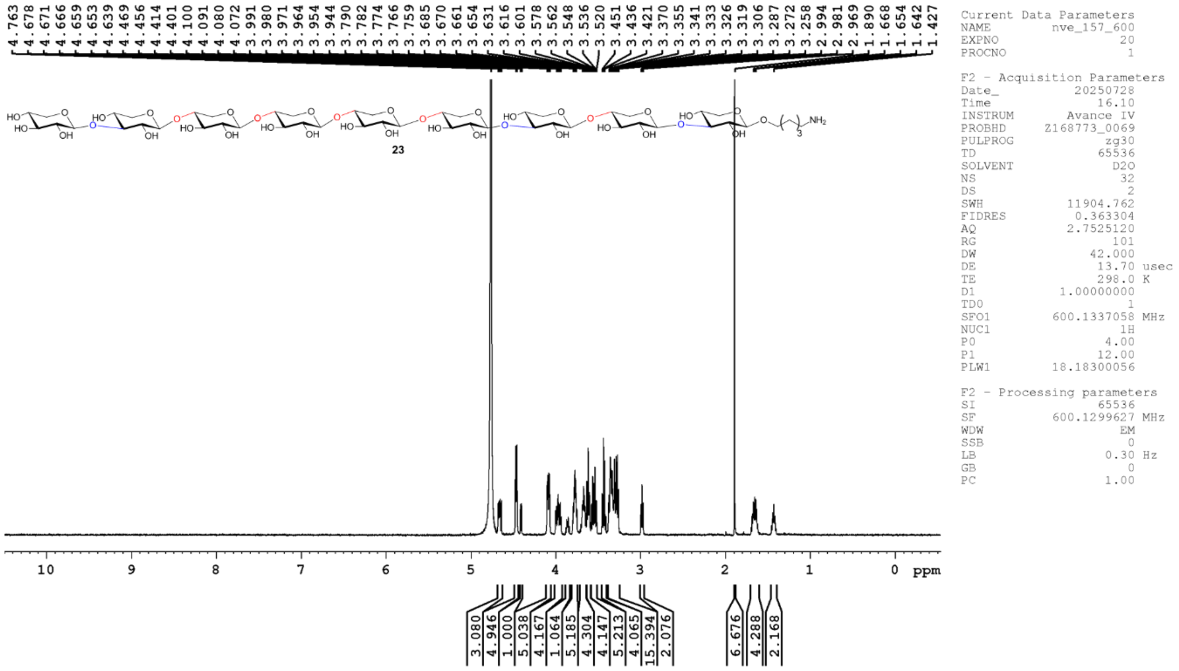

^{1}H NMR spectrum of compound **23** (600 MHz, D_2O)

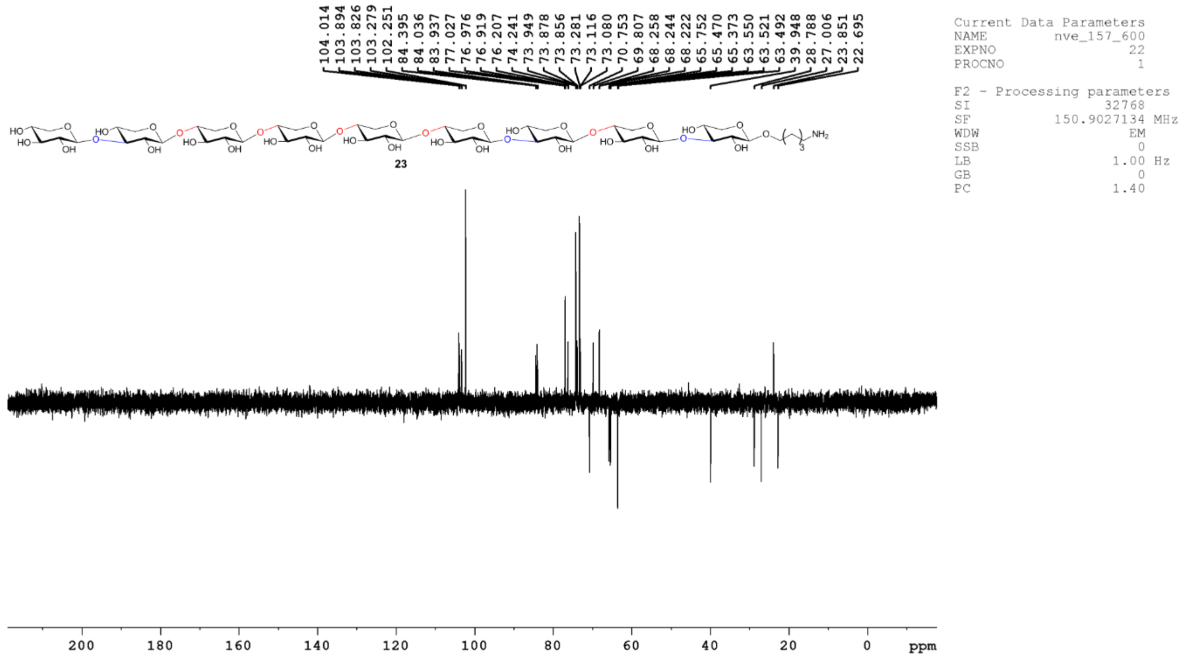

^{13}C DEPTQ135 NMR spectrum of compound **23** (151 MHz, D_2O)

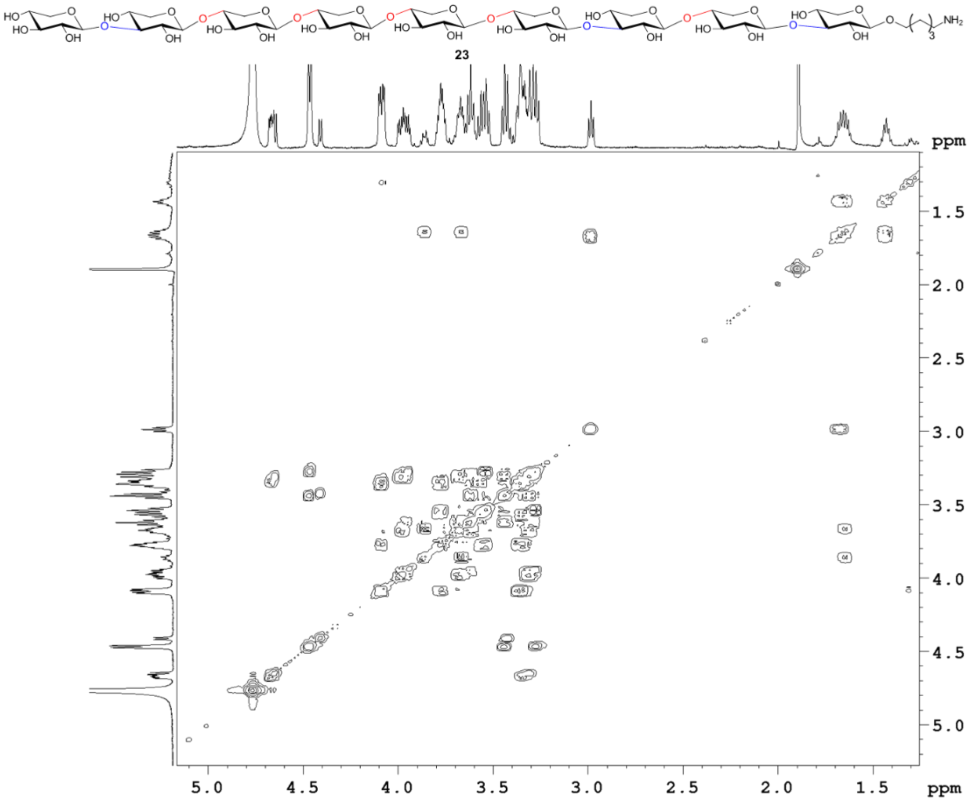

^{1}H-^{1}H COSY NMR spectrum of compound **23** (600 MHz, D_2O)

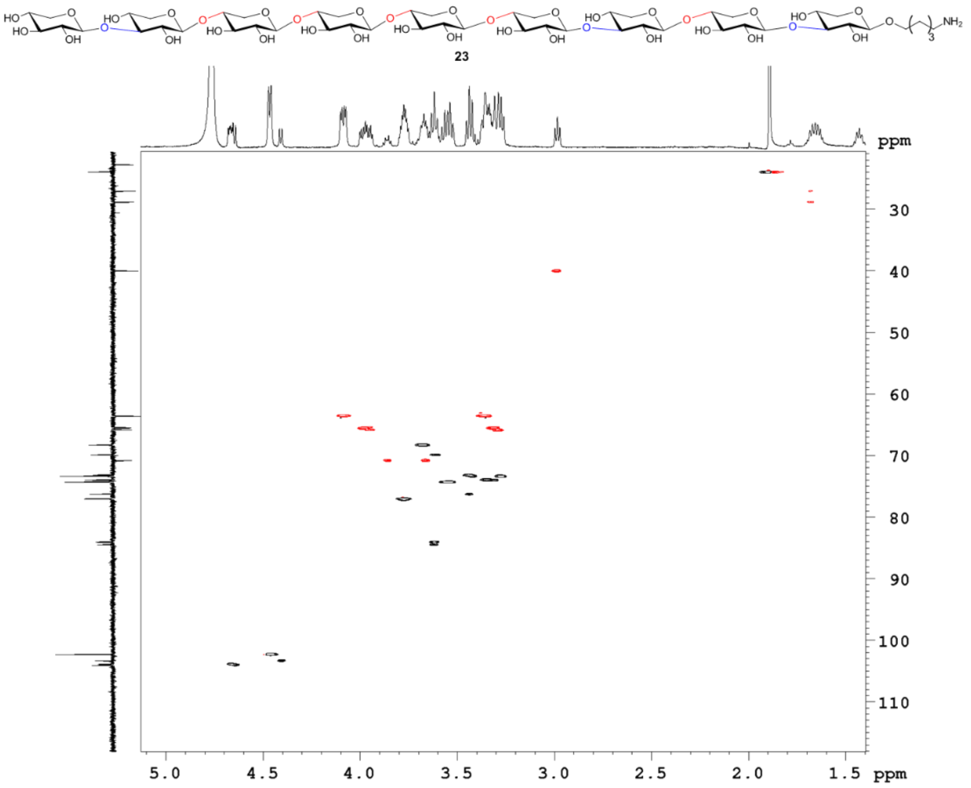

^{1}H-^{13}C HSQC NMR spectrum of compound **23** (600/151 MHz, D_2O)

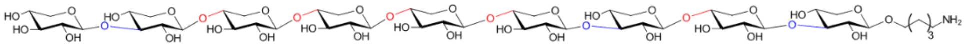

23

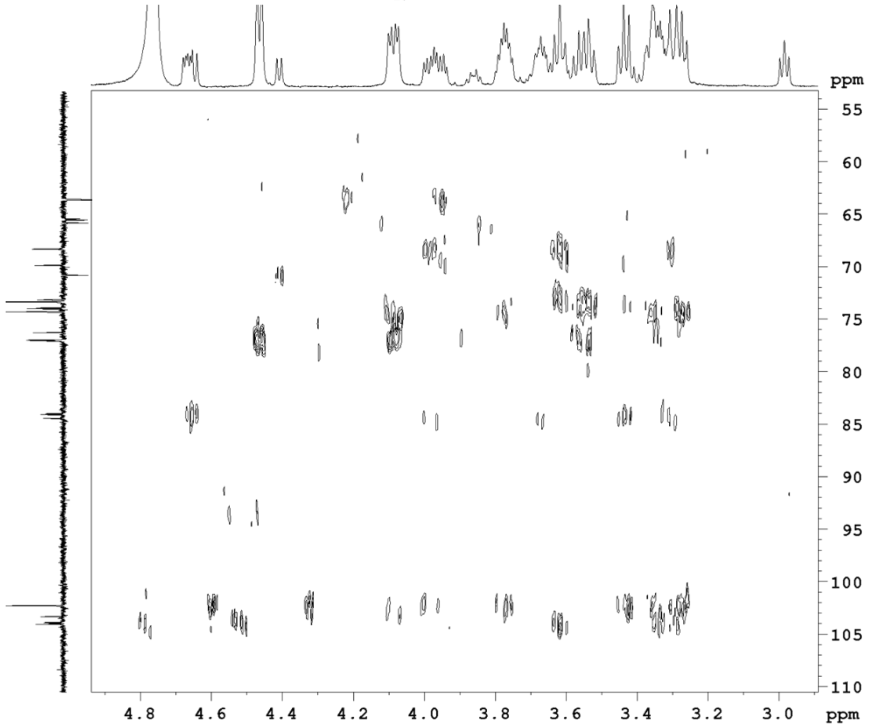

^{1}H-^{13}C HMBC NMR spectrum of compound **23** (600/151 MHz, D_2O)

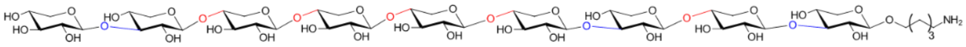

23

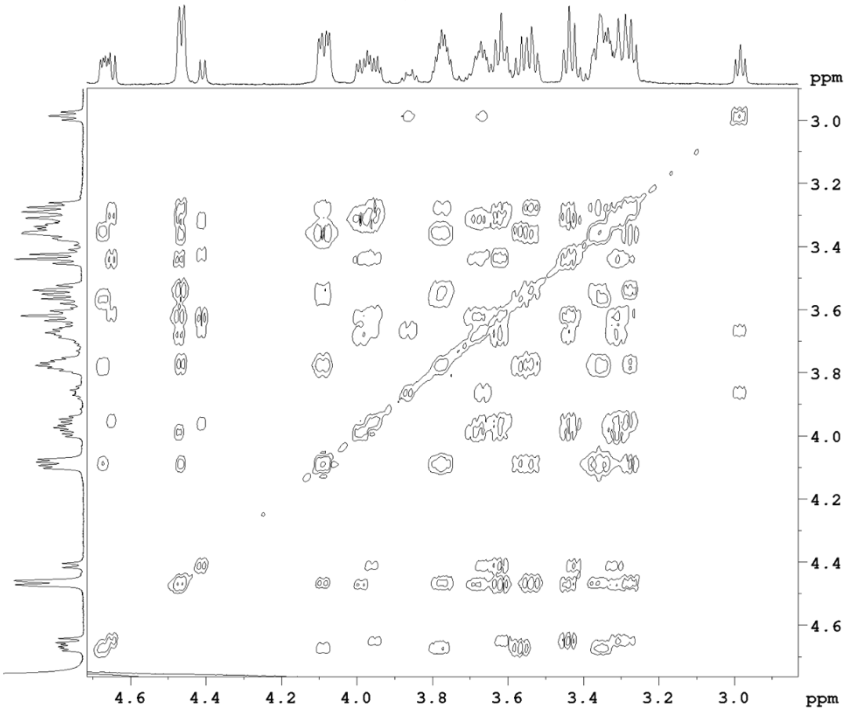

^{1}H-^{1}H TOCSY NMR spectrum of compound **23** (600 MHz, D_2O)

5. Extraction and purification of recombinant carbohydrate hydrolases

Recombinant MLXases, AlXyn62A and MfXyn26A, and the β3-xylanase, Xyl4, were produced as follows.[4, 5] 10 mL aliquots of LB medium supplemented with 50 µg/mL Carbenicillin were inoculated with single colonies of transformed *E. coli* BL21 (DE3), and grown overnight at 37 °C in an orbital shaker at 180 rpm. After 18 hours, overnight cultures were diluted 1:100 (v/v) in 400 mL of fresh antibiotic medium and further incubated at 37 °C and 200 rpm. At a 600 nm-optical density of ~0.6, the culturing temperature was reduced to 16 °C for 25 minutes. Recombinant expression was then induced by the addition of 200 µM isopropyl β-D-thiogalactopyranoside (IPTG).

After overnight expression, bacterial cells were pelleted by centrifugation (8,000 RCF for 20 min) and then frozen at –20 °C. 40 mL lysis buffer (50 mM Tris-HCl pH 7.5, 500 mM NaCl, 1 mM $MgCl_2$, 20 mM imidazole, 1% Triton X-100, and 0.5% sodium deoxycholate) supplemented with 10% (w/v) sucrose, 2 mg/mL lysozyme and 0.3 µL Pierce™ Universal Nuclease (Thermo Scientific) were used to extract crude protein for 1 hour at 37 °C and 180 rpm. The soluble protein fraction was isolated by centrifugation in a precooled fixed-angle rotor at 4 °C and 35,000 RCF for 40 min.

The recombinant hydrolases carry a C-terminal His6-tag, enabling purification by IMAC. After loading, 5 mL HisTrap HP columns (Cytiva) were washed with binding buffer (50 mM Tris-HCl pH 7.5, 500 mM NaCl, and 20 mM imidazole) until the UV signal baselined. For elution, a 20–300 mM imidazole gradient was applied while monitoring A280 to pool target enzyme containing fractions. All recombinant enzymes were dialyzed (3,500 Da MWCO membrane) against a 20 mM Tris-HCl pH 7 and 200 mM NaCl buffer at 4 °C overnight.

6. Xylanase assays and analysis of enzymatic degradation products using HPLC-MS

To solutions of 0.1% (w/v) synthetic xylan oligosaccharides in xylanase assay buffer (20 mM Tris-HCl pH 7.4, 200 mM NaCl), were added recombinant xylanases at 1 µM concentration with 75 µL as total reaction mixture volume per sample. Reaction mixtures were incubated at 20 °C (1-hour samples) or 37 °C (24-hour samples). Products were purified using 25 mg bed weight HyperSep™ Hypercarb™ SPE (Thermo Scientific) cartridges according to the manufacturer's protocol using 450 µL of 80 mM $NH_4(HCOO)$ pH 3 for washing and 450 µL of 65% acetonitrile in 80 mM $NH_4(HCOO)$ pH 3 for elution. Purified samples were dried in a rotating evacuator before reconstitution in 20 µL water and LC-MS analysis. After injection of 15 µL, separation was performed on a Hypercarb™ column (Thermo Scientific) applying a gradient of 2.5–40% acetonitrile in water (see Table S5).

Table S5. LC/MS method using Hypercarb column.

Time (min)	ACN (%)
0.01	2.5
8	2.5
33	40
37	100

Flow rate = 0.7 mL/min.

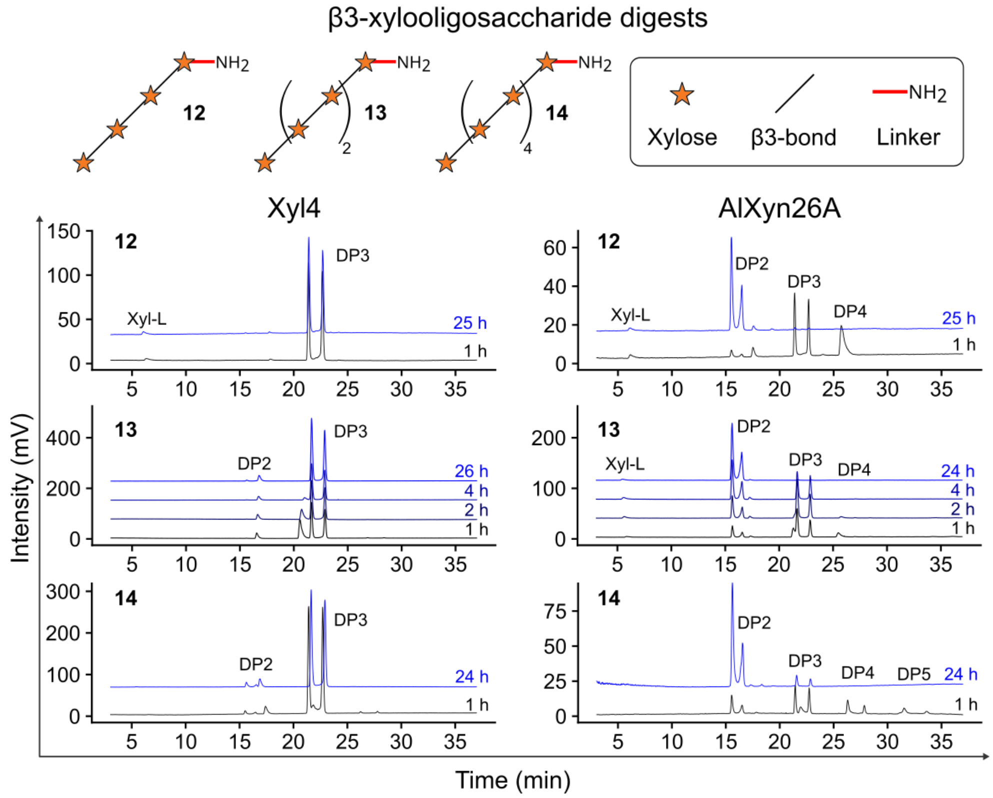

Figure S7. Digests of synthesized β3-xylooligosaccharides by recombinant xylanases. Symbol structures of the starting materials are shown on top. Reactions using Xyl4 and AlXyl26A are below in the left and right column, respectively. All chromatograms are displayed with ELS-intensities in millivolts (mV) against the retention time in minutes. Traces after 1 hour of incubation are drawn in black, and after one day (24–26 hours as indicated), in blue. Time-course of hexasaccharide **13** digests by Xyl4 and AlXyn26A monitored between 1 hour and 26 or 24 hours. Peaks are labeled by the degree of polymerization (DP) of the respective product β3-XOS or as linker-functionalized xylose-monosaccharide (Xyl-L).

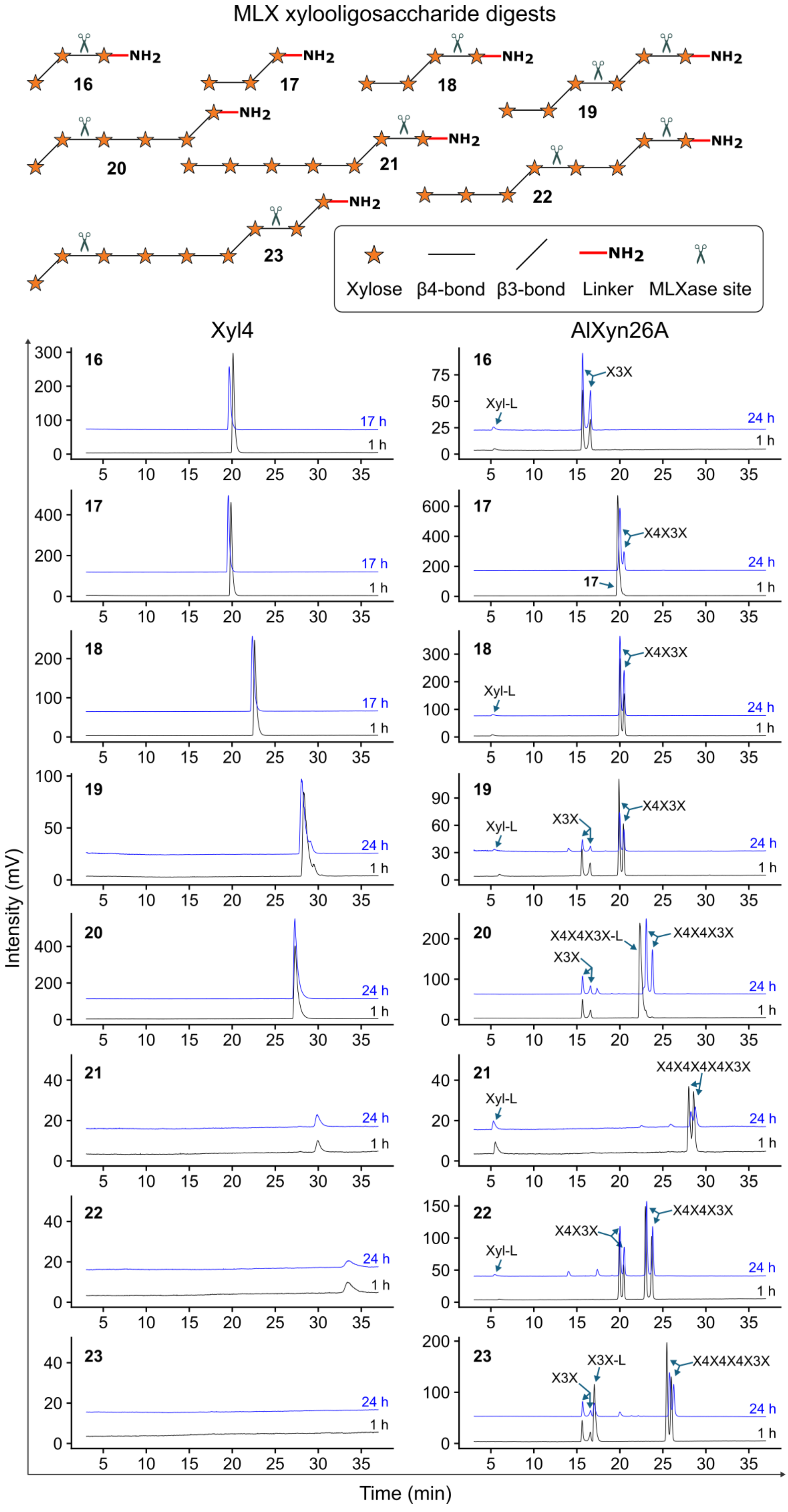
MLX xylooligosaccharide digests
16
17
18
19
20
21
22
23
NH2
Xylose
β4-bond
β3-bond
Linker
MLXase site
Xyl4
AlXyn26A
Intensity (mV)
Time (min)
17 h
24 h
1 h
Xyl-L
X3X
X4X3X
X4X4X3X-L
X4X4X3X
X4X4X4X4X3X
X3X-L
X4X4X4X3X

Figure S8. Digests of synthesized MLX oligosaccharides by recombinant xylanases. Symbol structures of the starting materials are shown on top. Reactions using Xyl4 and AlXyl26A are below in the left and right column, respectively. All chromatograms are displayed with ELS-intensities in millivolts (mV) against the retention time in minutes. Traces after 1 hour of incubation are drawn in black, and after overnight (17 or 24 hours as indicated), in blue. Except for the unaltered starting materials in the Xyl4-column, peaks are labeled by the sequence of the respective MLX-OS (e.g., X4X3X for Xyl1β-4Xyl1β-3Xyl trisaccharide). Linker-functionalization is denoted with an "-L" addendum as in the linker-functionalized xylose-monosaccharide (Xyl-L).

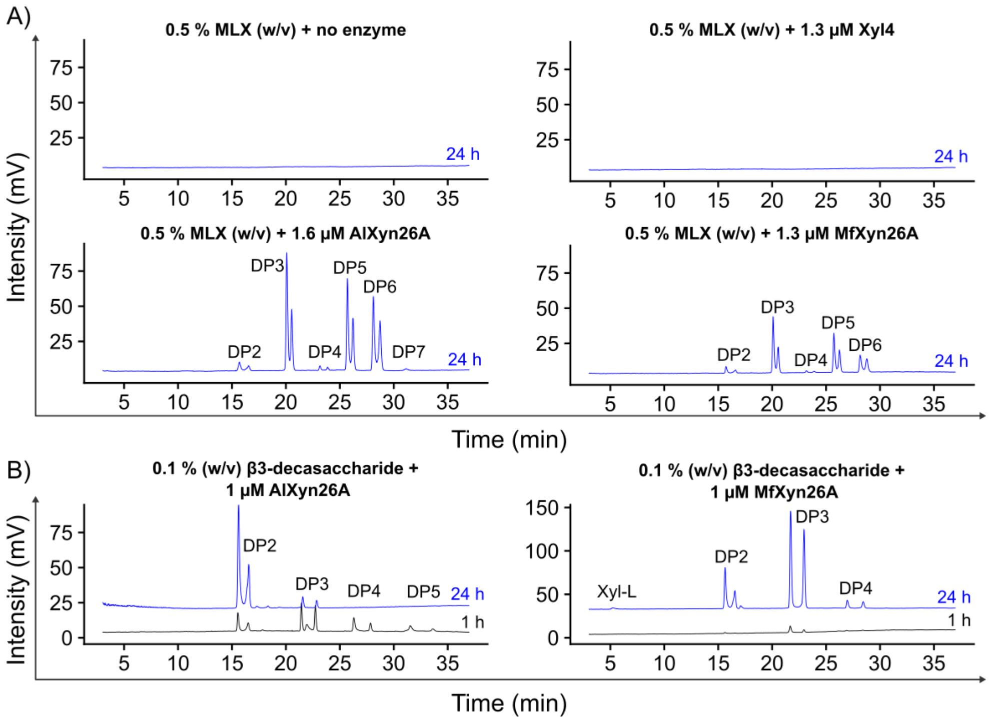

Figure S9. Digests of natural MLX and synthesized β3-xylan decasaccharide (**14**) using recombinant xylanases. In (A), from top left to bottom right: Reactions using 0.5% (w/v) solutions of natural MLX from *Palmaria palmata* in xylanase assay buffer and no enzyme, 1.3 µM Xyl4, 1.6 µM AlXyn26A, and 1.3 µM MfXyn26A. In (B): Reactions using 0.1% (w/v) solutions of β3-decasaccharide in xylanase assay buffer and 1 µM of either AlXyn26A or MfXyn26A are shown on the left and right, respectively. All chromatograms are displayed with ELS-intensities in millivolts (mV) against the retention time in minutes. Traces after 1 hour of incubation are drawn in black, and after 24 hours, in blue. Throughout the figure, peaks are labeled by the degree of polymerization (DP) of the respective product β3-XOS or as linker-functionalized xylose-monosaccharide (Xyl-L).

7. References

1. D. Schmidt, F. Schuhmacher, A. Geissner, P. H. Seeberger, F. Pfrengle, Automated Synthesis of Arabinoxylan-Oligosaccharides Enables Characterization of Antibodies that Recognize Plant Cell Wall Glycans. *Chem. Eur. J.* **2015**, *21*, 5709–5713.

2. D. Cai, Y. Bian, S. Wu, K. Ding, Conformation-Controlled Hydrogen-Bond-Mediated Aglycone Delivery Method for α-Xylosylation. *J. Org. Chem.* **2021**, *86*, 9945–9960.

3. N. E. Underlin, M. Böhm, R. Madsen, Synthesis of Arabinoxylan Oligosaccharides by Preactivation-Based Iterative Glycosylations. *J. Org. Chem.* **2019**, *84*, 16036–16054.

4. M. Kiyohara, K. Sakaguchi, K. Yamaguchi, T. Araki, T. Nakamura, M. Ito, Molecular Cloning and Characterization of a Novel β-1,3-Xylanase Possessing Two Putative Carbohydrate-Binding Modules from a Marine Bacterium Vibrio sp. Strain AX-4. *Biochem. J.* **2005**, *388*, 949–957.

5. F. Zhao, C.-M. Yu, H.-N. Sun, L.-S. Zhao, H.-T. Ding, H.-Y. Cao, Y. Chen, Q.-L. Qin, Y.-Z. Zhang, P.-Y. Li, X.-L. Chen, A Novel Class of Xylanases Specifically Degrade Marine Red Algal β1,3/1,4-Mixed-Linkage Xylan. *J. Biol. Chem.* **2023**, *299*, 105116.
